# Development of a multi-epitope chimeric vaccine *in silico* against *Babesia bovis*, *Theileria annulata*, *and Anaplasma marginale* using computational biology tools and reverse vaccinology approach

**DOI:** 10.1371/journal.pone.0312262

**Published:** 2025-01-24

**Authors:** Amam Zonaed Siddiki, Sabreena Alam, Farhan Fuad Bin Hossen, Md. Abdul Alim

**Affiliations:** 1 Department of Pathology and Parasitology, Chittagong Veterinary and Animal Sciences University (CVASU), Chittagong, Bangladesh; 2 Department of Biochemistry and Biotechnology, University of Science and Technology Chittagong (USTC), Chittagong, Bangladesh; The University of Texas Medical Branch at Galveston, UNITED STATES OF AMERICA

## Abstract

The three rickettsial parasites- *Babesia bovis*, *Theileria annulata* and *Anaplasma Marginale* are responsible for causing Babesiosis, Theileriosis and Anaplasmosis among cattle. These diseases exist due to spreading of infected ticks. A large number of cattle were found to suffer from mixed infections caused by the three parasites at the same time. Due to these reasons cattle have been devoid of milk production with reduced meat availability. Hence, it is a matter of urgency for the immunity of cattle to exhibit resilience against all three rickettsial parasites. It could be possible if trials are carried out after producing a subunit chimeric vaccine against the rickettsial protozoan parasites and introducing it into the bloodstream of the cattle species. In this paper, we have used the process of reverse vaccinology to conduct a study in which we have developed a multi-epitope subunit chimeric vaccine against three protozoan parasites. We constructed three chimeric vaccine sequences from which only one chimeric vaccine construct was found to be an effective and efficient vaccine which is stable with high solubility and negative allergenicity. Following that, we performed molecular docking of the refined chimeric vaccine construct with Rp-105 and TLR-9. It was observed that the chimeric vaccines interacted with the receptors with high binding energy. Immune simulation was also performed to determine the potentiality of the chimeric vaccine for eliciting an immune response. The best-designed chimeric vaccine construct was then reverse transcribed and adapted for the host *E*. *coli* K12 strain which was later inserted into the pET28a (+) vector for the cloning and expression of the vaccine. The study could be a good initiative for the development of an effective chimeric vaccine against bovine parasites.

## 1. Introduction

Babesiosis, Theileriosis and Anaplasmosis are three tick-borne diseases spreading in many parts of the world, including Bangladesh. Babesiosis and Theileriosis are caused by the apicomplexa parasites *Babesia bovis* and *Theileria annulata*, respectively. Anaplasmosis is caused by the rickettsial gram-negative parasite, *Anaplasma marginale*. These are pathogenic parasites that require vectors such as ticks to spread among dairy cows, buffaloes, bulls and ox. Approximately twenty types of ticks help in the transmission of *Anaplasma marginale* and mechanical transmission occurs through biting flies and fomites. As a result, substantial numbers of cattle become infected and die every year. In Bangladesh, much of the economy has suffered due to the experience of loss in the dairy and beef industries. Babesiosis, Theileriosis and Anaplasmosis have become a major reason for the reduction in supply of milk, meat and other dairy products. It has also been observed that it is entirely possible for individual cattle to acquire mixed infections with Babesia, Theileria and Anaplasma. In 2015, a recent study held in Kenya observed that about more than half the samples of cattle were infected by at least two hemoparasites of different genera, and 29 various types of mixed infections were identified in single cattle caused by five different pathogens [1]. In the year 2019, research was carried out in Bangladesh which demonstrated that tick borne diseases caused by both *Anaplasma spp*. and *Babesia spp* was 33% whereas all the three rickettsial parasites *Babesia bovis*, *Theileria annulata* and *Anaplasma marginale* responsible for multiple infections in cattle was 1%. Even though the multiple infections caused by all the three rickettsial parasites in Bangladesh were 1%, these infected cattle were strong carriers and were the main reason for the transmission of the diseases to the healthy cattle [2]. Another study held in Turkey revealed that about 22% of animals were found to be infected by two or three parasites [3]. Therefore, it has become very demanding to design an in-silico based chimeric peptide vaccine that possesses all the antigenic and functional epitopes for cattle to develop their immunity against all the three protozoan parasites, Babesiosis, Theileriosis and Anaplasmosis. This paper involves all the steps involved in the process of constructing the chimeric vaccine against the three protozoan parasites—Babesia, Theileria and Anaplasma. We have described each protozoan parasite in detail and identified its target proteins for vaccine development as follows:

### Babesia bovis

*Babesia bovis*, is a protozoan, apicomplexa parasite responsible for spreading bovine babesiosis in cattle [4]. Bovine babesiosis has spread in most parts of the world including, North America [5], South America, Asia [6], Africa [7], Europe [8] and Australia [9]. In Bangladesh, Bovine Babesiosis caused by *Babesia bovis* has been observed in various districts such as Chittagong [10], Mymensingh [11], Dhaka, Sirajganj and Nikhangsori [2]. Bovine babesiosis is characterized by fever within the first few days of infection followed by anorexia, high respiration rate is observed during the movement of cattle, destruction of large numbers of erythrocytes leading to anemia, pipe-stem diarrhea, muscle tremors and weight loss. Intermediate stages of infections involve hemoglobinemia and hemoglobinuria (red to port wine-colored urine), jaundice, constipation, abortion and infertility in bulls. *Babesia bovis* is classified under the family Babesiidae, with class Sporozoasida and order Eucoccidiorida belonging to the phylum Apicomplexa. In 2003, Criado- Fornelio [8] categorized *Babesia bovis* into a group along with other Babesia species that had the ability to parasitize only ungulates and hence, termed the group Ungulibabesids. Of this group, *Babesia bigemina*, *Babesia bovis*, *Babesia major* and *Babesia ovata* were reported to infect only cattle. Another group was termed babesids which involved *Babesia divergens* that had the capability to infect cattle too [4]. Among all the Babesia species that infect cattle, *Babesia bovis*, is believed to be more pathogenic than the rest of the species.

*Babesia bovis* is transmitted through the invasion of infected ticks, especially *Boophilus microplus*, *Boophilus annulatus* and *Boophilus geigyi*. (Now Boophilus is termed Rhipicephalus). The protozoan parasite, *Babesia bovis* infects the host by parasitizing only erythrocytes. The two outer membrane proteins of *Babesia bovis* recruited for vaccine construction include MSA-2c (Merozoite surface antigen-2c) and AMA-1 (Apical Membrane antigen-1). The functions of MSA-2c and AMA-1 are listed below.

### 1.1 MSA-2c

Msa-2c is an immunostimulatory *B*. *bovis* surface protein encoded by four copies of variable merozoite surface antigen (VMSA) genes. The four MSA-2c genes named msa-2a1, a2, b and c [12] reside on chromosome 1, tandemly organized in a head-to-tail arrangement. Merozoite surface antigens help in identifying parasites, are responsible for pathogenicity, attachment to erythrocytes and cause invasion [13]. These merozoite surface antigens are located on the surface of both sporozoites and merozoites. They have been revealed to contain neutralization-sensitive B-cell epitopes that are surface exposed [14,15]. MSA-2c was discovered to be the most conserved gene among the rest of the MSA antigens of the VMSA family. Due to this reason, MSA-2c has been observed as potential vaccine candidates.

### 1.2 AMA-1

AMA-1 is an outer membrane surface protein of *Babesia bovis*, which collects in micronemes at first before being transported to the surface of the parasite in the period of invading host cells. The function of AMA-1 is known to play a role in ‘moving junctions’ that force the transportation of parasites into the parasitophorous vacuole [16–18]. It has also been confirmed that antibodies produced against AMA-1 neutralize the invasion of the parasite in infected cells [19,20]. In 2019, a study was also held that confirmed that the AMA-1 surface protein also consists of conserved and exposed B-cell epitopes that trigger the production of antibodies in cattle [21]. For example, in 2021, a research study was also held to predict a B-cell epitope from the *B*. *bovis* AMA-1 surface protein in domain I, PAN motif by the utilization of B-cell prediction software. The results displayed the location of the predicted BbAMA-1 epitope to be between amino acid residues 181 and 230, belonging to domain I. This localized epitope was proposed to be neutralization-sensitive and could be used as an important target for vaccine development against *Babesia bovis* [22]. MSA2-c and AMA-1 are also predicted to activate Th1 cells and cause the expression of CD45RO and CD62L that produce IFN-c which in turn, elevates the expression of IgG2 by B-cells and nitric acid by macrophages [23].

#### Theileria annulata

*Theileria annulata* is classified under Phylum-apicomplexa, Order- Piroplasmida, Suborder—Leucosporidea, Family- Theileriidae by Du Toit in 1918 [24]. Tropical Theileriosis is a disease observed in cattle infected with *Theileria annulata*. It is estimated that there are two types of Theileria parasites that have infected more than 250 million cattle worldwide. This includes *Theileria parva* and *Theileria annulata*. *Theileria parva* infections are widespread in Eastern, Central and Southern Africa. In contrast, *Theileria annulata* infections are very prevalent among cattle in the Middle East, Asia, China, India and Southern Asia. Therefore, being aware of all the conditions, an in silico based chimeric peptide vaccine design is carried out for *T*. *annulata* and not for *T*. *parva*. Theileriosis is characterized by destruction of a large number of erythrocytes leading to severe anemia along with swelling of the lymph nodes. The symptoms also include fever, increased pulse and respiration rate, protruding of eyeballs from the socket, continuous flowing of tears, loose feces (yellow colored), red urine, ulcers within the tongue, and induced coma until death [25]. They die within 10–15 days (about 2 weeks) of infection due to pulmonary edema and severe gastroenteritis. The apicomplexa protozoan parasite increases its distribution among cattle through the transmission of Hyalomma [26] ticks. *T*. *annulata* like *B*. *bovis* infects cells in the form of sporozoites which later infect B-lymphocytes and macrophages. The two outer membrane proteins of *Theileria annulata* that have been used for vaccine construction are SPAG-1 and TASP. TASP and SPAG-1 have the following functions and features listed below.

### 1.3 SPAG-1

SPAG-1 (Sporozoite antigen-1) is an immunodominant surface antigen that is considered to be the best candidate for vaccine design due to the presence of neutralizing epitopes near the C-terminal of the protein. It has also been hypothesized that the C-terminus SR1 region consists of ligands that are used to recognize host cells. SPAG-1 is coded by a single gene that consists of many RFLPs (Restriction fragment length polymorphism) [27]. The protein SPAG-1 has been fully sequenced earlier and demonstrated a high level of polymorphism. It was found that both N-terminus and C-terminus variants shared approximately 92%-97% similarity and 60% identity in the central region. The C-terminal half of SPAG-1 is also 56% identical to the P67 of *Theileria parva* which suggests that the vaccine constructed against *T*. *annulata* could also be helpful against *T*. *parva* [28]. The N-terminus and the C-terminus exhibit conservation due to their role in host cell invasion [29].

### 1.4 TASP

TASP is an immunodominant protein [30] belonging to *Theileria annulata* which has been discovered to consist of an N-terminal region composed of a signal peptide and a C-terminal region coding for the membrane-spanning region which confirms that TASP is located at the membrane. Both the N-terminal regions and the C-terminal regions were conserved, while the C-terminal regions showed polymorphism within the amino acid sequence between different allelic variants of each parasite isolate. TASP is expressed as a surface membrane protein in both the sporozoite and schizont stages of the parasite’s life cycle. It has also been analyzed that the protein, TASP has 93% similarity to the polymorphic immunodominant molecule (PIM) of *Theileria parva* which is believed to be a homologue of PIM [28]. Recently, a study emerged confirming that cytotoxic t-cells are effective agents in the fight against *Theileria annulata* [31,32]. The TASP molecule consists of 22 high-binding peptides. Therefore, TASP can function as a strong immunogenic MHC I antigen to attract and stimulate cytotoxic t-cells. TASP also triggers neutralizing antibodies against sporozoites [33,34].

#### Anaplasma marginale

Bovine anaplasmosis is an infectious disease of cattle caused by *Anaplasma marginale* [35]. It is responsible for causing the death of a large number of cattle due to the destruction of numerous amounts of red blood cells. Bovine anaplasmosis is spread through the transmission of ticks such as *Dermacentor spp* [36,37]. *Anaplasma marginale* is an obligate, intraerythrocytic rickettsial belonging to the family Anaplasmataceae of the order Rickettsiales. The disease is more endemic to cattle in Europe, Africa, Asia, Australia, and most parts of America [38,39]. In Bangladesh, it has spread to many districts such as Mymensingh [11], Sirajganj [40] and Chittagong [41].

### 1.5 Vir B10

Vir-B10 is a highly conserved outer membrane protein of *Anaplasma marginale* belonging to the type IV secretion system (T4SS) consisting of many other T4SS proteins [42]. In 2007, research was conducted to determine the potentiality of the T4SS proteins to induce IgG and helper T cells following the vaccination of cattle. It was found that Vir B-10 together with Vir B9-1 and VirB9-2 triggered the strongest immunity by the secretion of IgG as well as stimulation of T-cell responses [43]. A major function of the T4SS is to transport proteins, DNA or protein-DNA complexes between bacterial and host cells. Vir B10 along with Vir B7 together make up the mid-core of the T4SS and is believed to be important for their survival and invasion in bovine erythrocytes and tick cells [44]. Vir B10 is a highly immunogenic protein because it is lacking in surface lipopolysaccharide (LPS) which enhances its surface exposure forming the outer cap of the T4SS core complex [43].

### 1.6 OMP1

OMP1 is present as a single discrete gene along with OMP14 on the same chromosome. OMP1 is expressed in large amounts in infected erythrocytes by *A*. *marginale*. They are highly conserved proteins during their infection in mammalian hosts and tick cells [45]. The fundamental function of the OMPs is to keep the native conformation of *A*. *marginale’s* outer membrane intact and secure by forming extensive inter- and intermolecular covalent and non-covalent bonds [46].

## 2. Methods and materials used

### 2.1 The UniProtKB server was used to retrieve outer membrane protein sequences

**Fig 1:**
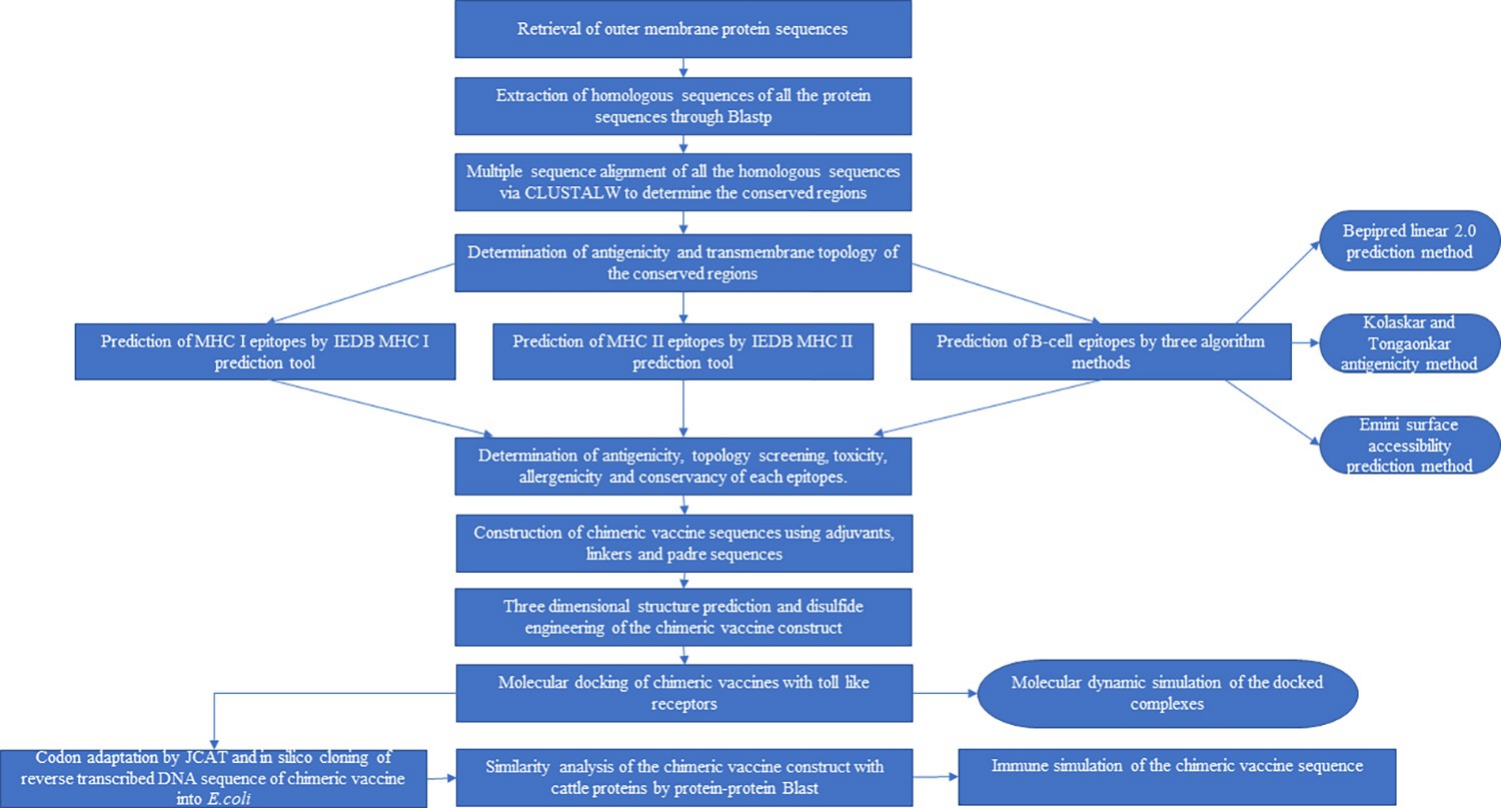
Flowchart representation of the methodology process for the current study.

The complete proteome of each of the apicomplexan, *Babesia bovis*, *Theileria annulata* and rickettsial parasite, *Anaplasma marginale* in the form of fasta sequences was retrieved by the utilization of the UniProtKB server (https://www.uniprot.org/uniprotkb?query=*) [47]. *Babesia bovis* str. T2Bo consisted of 3,216 proteins in total, *Theileria annulata* str. Ankara comprised of 3,790 proteins in total, and *Anaplasma marginale* consisted of 939 proteins in total. Among the whole set of proteins belonging to each parasite, only the outer membrane proteins were analyzed for their antigenicity using the server VaxiJen v2.0 (http://www.ddg-pharmfac.net/vaxijen/VaxiJen/VaxiJen.html) [48]. VaxiJen v2.0 server is a de novo approach whose principle is based on auto-cross covariance alteration to predict the antigenicity of the peptides and proteins. It provides an accuracy of 70%-89% depending upon the target organisms. All the proteins and epitopes which have acquired the antigenicity score greater than threshold value 0.5 were assumed to be highly immunogenic and thus were selected for further work. This involved a list of numerous forms of MSA-2c, AMA-1, SPAG-1 and TASP pertaining to the three parasites. Only proteins having the highest antigenic scores were chosen from each of the lists of proteins. The individual protein sequences of Vir B10 and OMP1 belong to *Anaplasma marginale* str. Florida only appeared in the UniProtKB server. These proteins were also examined for their antigenicity scores using the VaxiJen v2.0 server (http://www.ddg-pharmfac.net/vaxijen/VaxiJen/VaxiJen.html) [48]. DeepTMHMM v. 1.0 and TMHMM v. 2.0 servers (https://dtu.biolib.com/DeepTMHMM) [49] were used to determine the transmembrane topologies of all six proteins. Furthermore, the Protparam (https://web.expasy.org/protparam/#top) tool on the ExPasy server [50] was used to predict the physicochemical properties of all the six outer membrane proteins of the three parasites. The flowchart that illustrates the methodology for designing the in silico multi-epitope chimeric vaccine is (Fig 1).

### 2.2 Both MHC I and MHC II epitopes were predicted individually for the six outer membrane proteins

Each of the six outer membrane proteins possessing the highest antigenic scores was individually inserted into the BLAST (https://blast.ncbi.nlm.nih.gov/Blast.cgi?PAGE=Proteins) [51] tool of the BLAST server to extract all their homologous sequences from the NCBI (https://www.ncbi.nlm.nih.gov/) [52] database. Individually, all the proteins were inserted as a query, and the searches were restricted to *Babesia bovis* (taxid: 5865) for MSA-2c and AMA-1, *Theileria annulata* (taxid: 5874) for SPAG-1 and TASP-1 and *Anaplasma marginale* (taxid: 770). Following that, the homologous sequences of the different proteins were submitted to the server, CLUSTALW (https://www.ebi.ac.uk/Tools/msa/clustalo/) [53] to perform multiple sequence alignment (MSA). Then, the selected conserved regions were used to predict both MHC-I and MHC-II binding peptides by introducing them into the search box of the MHC I prediction tool v2.24 (http://tools.iedb.org/mhci/) [54] and MHC II prediction tool v1.0 (http://tools.iedb.org/mhcii/) [55] of the Immune Epitope Database (IEDB) server by keeping the parameters under default settings. The bovine MHC I alleles present in the IEDB server were all selected. According to the IEDB MHC I and MHC II prediction tool, the lower the percentile rank is achieved for each individual epitope, the higher binding affinity of the predicted epitopes. Following the research that was conducted in the year 2018 [56], we also chose our threshold of the percentile value to be less than 50 for all predicted MHC I and MHC II epitopes. We chose the threshold value of the percentile rank to be less than 50 to find all the promiscuous epitopes among identified T-cell epitopes. Promiscuous epitopes are those epitopes which bind to all the BoLA allele members of BoLA supertypes. This study includes 105 BoLA alleles of six BoLA class I supertypes viz. **BoLA-1** (15 BoLA alleles), **BoLA-2** (33 BoLA alleles), **BoLA-3** (30 BoLA alleles) **BoLA-4** (3 BoLA alleles), **BoLA-5** (4 BoLA alleles) **BoLA-6** (9 BoLA alleles) and other alleles such as BoLA-amani.1, BoLA-AW10, BoLA-D18.4, BoLA-gb1.7, BoLA-HD6, BoLA-JSP.1, BoLA-T2a, BoLA-T2b, BoLA-T2c, BoLA-T5, and BoLA-T7 to cover maximum population. Therefore, the MHC I epitopes and MHC II epitopes that had a percentile value less than 50 (threshold value) were selected for further analysis. All the selected BoLA class I supertypes with their related BoLA allele members can be observed in S1 File.

### 2.3 Building models of bovine MHC-II alleles, their refinement and stereochemical analysis

The sequences of bovine MHC-II alleles namely BoLA-DRB3*016:01, BoLA-DRB3*4101, BoLA-DRB3*3301 and BoLA-DRB3*4301 were downloaded from the Immuno Polymorphism Database (IPD). Since the PDB structures of these bovine alleles were not available in the Protein data bank, their crystal structures were modelled using homology modelling. The server SWISS-MODEL (https://swissmodel.expasy.org/) was integrated into the software for homology modelling. The server was initially used to identify templates with identical sequences homologous to bovine MHC-II alleles. This was accomplished through protein BLAST against the Protein Data Bank. The bovine MHC-II PDB model generated by SWISS-MODEL was then refined by the GalaxyRefine tool of the GalaxyWEB server. The stereochemical analysis was later carried out by the PROCHECK program of the SAVES v6.1 server.

### 2.4 Interaction studies between selected bovine MHC II alleles and selected MHC II epitopes

The PDB structures of the four bovine MHC II alleles such as BoLA-DRB3*016:01, BoLA-DRB3*4101, BoLA-DRB3*3301 and BoLA-DRB3*4301 were used to examine the binding affinity of the binding interactions with the predicted MHC II epitopes. Two predicted MHC II epitopes involving “TDGTTTGPGGNGEGG” and “PTKASSSGDGAAPCH” belonging to *Theileria annulata* were considered for molecular docking. Furthermore, two predicted epitopes such as “GPSEDGGGQGTDSRF” and “AQAAGGKLPGLLYPQ” belonging to *Anaplasma marginale* were also selected. The molecular docking between the bovine MHC II alleles and the selected epitopes was conducted using the server CABS-dock. The binding affinities were measured in kcal/mol.

### 2.5 Both the conserved regions and T-cell epitopes of the outer membrane proteins are screened for their antigenicity and transmembrane topology

The conserved regions were individually checked for their antigenicity by examining their antigenic scores using VaxiJen v2.0 server (http://www.ddg-pharmfac.net/vaxijen/VaxiJen/VaxiJen.html) and later screened for their transmembrane topology using DeepTMHMM v. 1.0 server (https://dtu.biolib.com/DeepTMHMM). All the conserved regions expected to be most antigenic and located in their outer membrane were chosen for MHC I and MHC II epitope prediction. Following the process carried out by IEDB (Immune epitope database server), all the predicted cytotoxic t-cell (MHC I) and helper t-cell (MHC II) epitopes were also analyzed for their level of antigenicity and transmembrane location using the same server. As a result, epitopes possessing potential antigenic scores and located in the outer membrane were separated from the rest of the epitopes for further analysis.

### 2.6 Assessment of allergenicity, toxicity and conservancy of each epitope

Three servers were utilized to assess the allergenicity of each epitope, involving AllerTOP v. 2.0 [57] (https://www.ddg-pharmfac.net/AllerTOP/), AllergenFP v.1.0. (https://ddg-pharmfac.net/AllergenFP/) [58] Allermatch v.1.0. (https://www.allermatch.org/allermatchsearch/form) [59]. The epitopes which were deduced to be non-allergenic were chosen and used to predict their toxicity by using the server, ToxinPred v 1.0 (https://webs.iiitd.edu.in/raghava/toxinpred/design.php) [60]. The ToxinPred v 1.0 server predicts the toxic peptides in a hybrid method by detecting motifs in the peptides and then query sequences are hit with a list of toxic peptide motifs. The peptides are confirmed as toxic when hits found against a peptide increase SVM score by 5 [61]. Finally, the conservancies of each of the non-toxic and non-allergenic epitopes were examined by the conservancy analysis tool (http://tools.iedb.org/conservancy/) [62] belonging to the Immune epitope Database server (IEDB) to determine whether all the selected epitopes are conserved within the result of the BLASTp (https://blast.ncbi.nlm.nih.gov/Blast.cgi?PAGE=Proteins) of each of the six proteins received from the NCBI database (https://www.ncbi.nlm.nih.gov/).

### 2.7 Three algorithm methods were used to predict B-cell epitopes

The three algorithm methods were Bepipred linear epitope prediction method 2.0 [63], Kolaskar and Tongaonkar antigenicity scale [64], and Emini surface accessibility prediction from IEDB b-cell prediction tool (http://tools.iedb.org/bcell/) which were applied to determine the b-cell epitopes for each of the six proteins- MSA-2c, AMA-1, SPAG-1, TASP, Vir B10 and OMP1. The B-cell epitopes are essential requirements to construct a vaccine. The B-cell epitopes present in the vaccine can interact and stimulate the attraction of b-lymphocyte and trigger them to produce antibodies. The BepiPred-2.0 server predicts B-cell epitopes from a protein sequence using a Random Forest algorithm trained on epitopes and non-epitope amino acids based on their three-dimensional structures. Residues with scores above the threshold value of 0.5 are expected to be part of an epitope. Kolaskar and Tongaonkar antigenicity scale uses the physicochemical properties of amino acids and their frequency of occurrence on proteins to predict epitopes. This provides 75% accuracy. Emini surface accessibility prediction method predicts epitopes based on a formula Sn = (n+4+i) (0.37)-6 where Sn = surface probability, dn = fractional surface probability value, and i = varies from 1 to 6. The peptide sequences with scores above a threshold value of 1.0 are predicted to be epitopes.

### 2.8 Three chimeric vaccine sequences were constructed

The sequences required to construct the three vaccines involved three different types of adjuvants. First vaccine construct consisted of bovine beta-defensin as the adjuvant, the second vaccine construct comprised of L7/L12 ribosomal protein and the third vaccine design possessed HBHA as an adjuvant. All the vaccine sequences were developed by organizing the adjuvants initially, followed by the best cytotoxic T-lymphocyte epitopes from each of the six outer membrane proteins of the three microorganisms (MSA- 2c, AMA-1, SPAG-1, TASP, Vir B10 and OMP1), top helper T-lymphocyte epitopes and then B- lymphocyte epitopes. PADRE sequences were arranged after adjuvant sequences and linkers were added between each epitope. In this way, an effective chimeric vaccine molecule was constructed to fight three microorganisms.

### 2.9 Assessment of allergenicity, antigenicity and solubility of individual chimeric vaccine constructs

The allergenicity of each chimeric vaccine construct was determined using two servers, AlgPred v.2.0 (https://webs.iiitd.edu.in/raghava/algpred/submission.html) [65] and AllerTop v.2.0 (https://www.ddg-pharmfac.net/AllerTOP/). The allergenicity of each epitope was tested to ensure that none of the epitopes in the chimeric vaccine are allergenic since most vaccines induce an allergic reaction after being introduced to the immune system of cattle [66]. The World Health Organization also states that a peptide sequence having a minimum of six amino acids over a window of 80 amino acids (0.35% sequence identity) is a potential allergen. The antigenicity of the chimeric vaccine sequences was predicted by the server, VaxiJen v2.0 and the solubility of the chimeric vaccine constructs was assessed through Protein-sol software (https://protein-sol.manchester.ac.uk/). The protein-sol server was used to determine the solubility of chimeric vaccines. The server processes amino acids and predicts solubility depending on 35 features of proteins such as weight, charge, PI, etc. And provides an initial fit to the solubility data. For the determination of the solubility of a query sequence, the values of each protein feature are averaged with each of the lower and higher subsets. They are then multiplied by feature weight, with feature weight normalized to sum to 1 [67].

### 2.10 Determination of the physicochemical properties and the prediction of the secondary structure of the chimeric vaccine construct

The physicochemical properties of the three chimeric vaccine constructs were predicted by the Protparam tool (https://web.expasy.org/protparam/#top) of the ExPasy server. All characteristics such as the number of amino acids, theoretical PI, molecular weight, amino acid composition, atomic composition, extinction coefficients, total number of atoms, hydropathicity (GRAVY values), instability index, aliphatic index, and estimated half-life related to the chimeric vaccine constructs were determined by the server. Two servers were utilized for the prediction of the secondary structures of the chimeric vaccine constructs-PSIPRED (http://bioinf.cs.ucl.ac.uk/psipred/) [68] and CFSSP (http://www.biogem.org/tool/chou-fasman/) [69]. These servers helped to display all the locations of alpha-helices, beta-sheets and coils present in the chimeric vaccine construct.

### 2.11 Three-dimensional structure prediction, refinement and disulfide engineering of the chimeric vaccine construct

The server, RaptorX (http://raptorx.uchicago.edu/StructurePropertyPred/predict/) [70] was used to predict the three-dimensional structure of the chimeric vaccine molecule, V3. It works by displaying 10 PDB model structures of the chimeric vaccine construct. This is based on the similarity between the target protein and PDB templates. The top-quality PDB structure of the chimeric vaccine construct possessing the highest Ramachandran plot value was selected and sent for refinement. The GalaxyRefine tool belonging to the GalaxyWEB server (https://galaxy.seoklab.org/cgi-bin/submit.cgi?type=REFINE) [71] was used to refine the model. The refinement is carried out to make the predicted 3-D structures of the vaccine more reliable and accurate for further docking purposes. SAVES v6.1 server (https://saves.mbi.ucla.edu/) was then employed for validating the refined vaccine structures via different programs such as ERRAT [72], PROCHECK [73], and VERIFY 3D [74]. The geometry of each amino acid residue is examined to predict the overall geometry and stereochemical quality of the structure by the program PROCHECK. It also uses the Ramachandran plot to determine all the amino acids present in favored or disallowed regions, providing the total value in percentage. If the predicted value is above 90%, then the refined model of the vaccine construct is expected to be an accurate model. The ERRAT program provides a quality factor value. The greatest value of the quality factor indicates an idea of a better protein structure because it gives the value of non-bonded interactions after comparing them with that of highly defined proteins. Depending on the location and surroundings of the protein, the VERIFY 3D program seeks the compatibility of the protein’s tertiary structure with its primary structure. Subsequently, to increase the stability of the vaccine construct, the server DbD2 v2.13 (http://cptweb.cpt.wayne.edu/DbD2/) [75] was used to install disulfide bonds within the chimeric vaccine construct. Disulfide bonds were formed between pairs of amino acids located in the highly mobile region. They possessed proper geometry because these amino acid residues could be easily mutated to cysteine.

### 2.12 Conformational B-cell epitopes were predicted using IEDB

The conformational B-cell epitopes were determined from the vaccine construct by operating the ellipro tool [76] of the IEDB server. This discontinuous B-cell epitopes will further stimulate B-cell production and induce antibody secretion. The PDB format of our vaccine construct was uploaded to the server and all parameter settings were kept default. The Ellipro tool predicts discontinuous antibody epitopes depending on the three-dimensional structure of the vaccine design, the PI value (protrusion index) and neighboring cluster residues. The epitopes that receive higher scores demonstrate greater solvent accessibility. For predicting discontinuous B-cell epitopes, the default minimum score was 0.5 and the maximum distance was set at 6 Angstroms.

### 2.13 Molecular docking of the vaccine construct with toll-like receptor

ClusPro v2.0 (https://cluspro.bu.edu/login.php) [77], HDOCK v4.0 (http://hdock.phys.hust.edu.cn/) [78], and PatchDock v1.3 servers (https://bioinfo3d.cs.tau.ac.il/PatchDock/php.php) [79] were used for the molecular docking of the chimeric vaccine construct with the bovine cell surface molecules, RP-105 (https://www.rcsb.org/structure/3RG1) and TLR-9 (https://www.rcsb.org/structure/5Y3M). The TLR-4 receptor PDB format is not available in RCSB Protein Data Bank. In this case, RP-105 was used in molecular docking instead of TLR-4. RP-105 is 30% similar in its amino acid sequence to the bovine TLR-4 receptor (https://www.rcsb.org/structure/3RG1). Moreover, RP-105 can accelerate the interaction of toll-like receptor 2 with macrophages present in cattle blood and also stimulate the appearance of immunoglobulins such as IgM and IgG3 after their increased production [80]. It was easily retrieved from RCSB Protein Data Bank in its PDB format. Docking displayed the best vaccine-toll receptor complexes according to their global binding energy and electrostatic interaction. These docked complexes were then refined by the FireDock server (https://bioinfo3d.cs.tau.ac.il/FireDock/php.php) [81] to represent the top 10 docked complexes with the highest global binding energy. Following that, the software Discovery Studio Visualizer (https://discover.3ds.com/discovery-studio-visualizer-download) was used to understand which amino acids of the receptors had participated in docking with the chimeric vaccine V3.

### 2.14 Peptide docking with toll-like receptors

The peptides used to construct the chimeric vaccine were individually docked with the toll-like receptors- TLR9 and Rp-105. The server used to conduct peptide-receptor docking is HPEPDOCK (http://huanglab.phys.hust.edu.cn/hpepdock/) [82]. About 25 peptides including six MHC I epitopes, six MHC II epitopes and thirteen B-cell epitopes were docked with toll-like receptors- TLR9 and Rp-105.

### 2.15 Molecular dynamic simulation of the vaccine-receptor complex

Following the molecular docking of the vaccine construct with the toll-like receptors, molecular dynamic simulation of the most suitable vaccine-receptor complex was carried out by utilizing the server, iMODS [83](https://imods.iqf.csic.es/). The parameters were all set to their default settings. The iMODS server makes predictions regarding the vaccine-receptor complex in terms of eigenvalue, atomic B-factor, elastic network, deformability and covariance map. It depends on the deformability of the entire protein in order for individual residues to be deformed. The eigenvalue is related to the energy required to deform the protein structure. The protein-receptor complex becomes easier to distort as the eigenvalue decreases. The eigenvalue represents motion stiffness. The variance is associated with each normal mode and is inversely proportional to the eigenvalue. Co-variance represents the coupling between residue pairs. Red color indicates correlated motion, white color shows uncorrelated motion and blue color illustrates anti-correlated motion. The elastic network determines which pairs of atoms are connected by springs. Dark colored dots indicate the spring stiffness.

### 2.16 Immunological analysis of the vaccine construct through immune simulation

The immune server, C-immSim [84] was utilized to analyze the immunological characteristics of the newly designed chimeric vaccine construct. The web-server is free online and works with Position-Specific Scoring Framework (PSSM). All the parameters were set as default settings except that the MHC-II alleles were selected as DRB3_0216 and DRB3_0223 including three vaccine injections with no polysaccharides. The time step of injections and adjuvants was kept at default settings.

### 2.17 Codon adaptations, in silico cloning and similarity analysis of the chimeric vaccine construct with cattle proteins

In order to clone chimeric vaccine construct V3, *E*. *coli* strain k12 was used. At first, the amino acid sequence of the chimeric vaccine design, V3 was converted to the DNA sequence codon by using the codon adaptation tool (JCAT) (http://www.jcat.de/) [85]. A restriction site, BglII was added to the N-terminal and BglI was added to the C-terminal. Then a reverse codon adaptation sequence of the chimeric vaccine molecule, V3 was produced. The adapted DNA sequence of the chimeric vaccine construct, V3 was then inserted into the plasmid pET28a (+) vector with Snapgene v6.2.0 software (https://www.snapgene.com/). The similarity analysis of the chimeric vaccine construct with cattle proteins was performed by protein-protein BLAST (https://blast.ncbi.nlm.nih.gov/Blast.cgi?PAGE=Proteins) against *Bos sp*. (taxid: 29061).

## 3. Results

### 3.1 The UniProtKB server was used to retrieve outer membrane protein sequences

The whole proteome of the two apicomplexans and rickettsial parasites *Babesia bovis* str. T2Bo, *Theileria annulata* str. Ankara and *Anaplasma marginale* Florida str. were derived from the server, UniProtKB (https://www.uniprot.org/uniprotkb?query=*) which displayed total proteins of 3,216, 3,790 and 939, respectively. Among the proteome of each parasite, only the outer membrane proteins from each of the parasites were chosen for the modeling of an effective chimeric vaccine. The outer membrane proteins were individually measured for their antigenic scores by the server, VaxiJen v2.0. From each parasite, two outer membrane proteins were selected with the highest antigenicity score. These included MSA-2c (Accession ID: A0A0D6A0Y2) and AMA-1 (Accession ID: T2HG14) from *Babesia bovis*, SPAG-1 (Accession ID: Q26675) and TASP (Accession ID: A7UAD3) from *Theileria annulata* and, Vir- B10 (Accession ID: B9KHA8) and OMP-1 (Accession ID: Q2V9Q7) from *Anaplasma marginale* with the highest probable antigenic scores of **0.5202, 0.5724, 0.8560, 0.9452, 0.5802** and**, 0.5624,** respectively. This is represented in Table 1. The physicochemical properties of each of the outer membrane proteins belonging to the three bovine parasites can be observed in Table 2.

**Table 1:** The antigenic scores of the six proteins- MSA-2c, AMA-1, TASP, SPAG-1, Vir B10, and OMP1 with their location.

Name of Microorganism	Name of Protein	Accession ID	Vaxijen score (Antigenic score)	Location
***Babesia bovis***	**MSA-2c**	A0A0D6A0Y2	0.5202	Outer membrane
	**AMA-1**	T2HG14	0.5724	Outer membrane
***Theileria annulata***	**SPAG-1**	Q26675	0.8560	Outer membrane
	**TASP**	A7UAD3	0.9452	Outer membrane
***Anaplasma marginale***	**Vir- B10**	B9KHA8	0.5802	Outer membrane
	**OMP1**	Q2V9Q7	0.5624	Outer membrane

**Table 2:** Protparam analysis of the six outer membrane proteins- MSA-2c, AMA-1 (*Babesia bovis*) TASP, SPAG-1 (*Theileria annulata*) and Vir-B10, OMP-1 (*Anaplasma marginale*).

*Name of Microorganisms*	*Proteins*	*Accession ID*	*Molecular weight*	*Instability* *Index*	*Estimated Half-life*	*Theoretical* *pI*	*Amino acids*	*Total No*. *of Atoms*	*Extinction Co-efficient*
***Babesia bovis***	MSA-2c	A0A0D6A0Y2	29467.12	40.25	30 hours	4.59	265	4117	22015
	AMA-1	T2HG14	67230.57	48.20	30 hours	6.30	605	9291	101160
***Theileria annulata***	SPAG-1	Q26675	91885.12	41.68	30 hours	5.04	907	12903	18450
	TASP	A7UAD3	14661.40	84.55	30 hours	3.92	132	1970	125
***Anaplasma marginale***	Vir-B10	B9KHA8	48013.74	51.38	30 hours	5.16	445	6775	25900
	OMP-1	Q2V9Q7	34811.37	44.76	30 hours	9.05	330	4870	31080

### 3.2 Both MHC I and MHC II epitopes were predicted individually for the six outer membrane proteins

The server BLASTp (https://blast.ncbi.nlm.nih.gov/Blast.cgi?PAGE=Proteins) tool was operated to achieve a total set of six homologous protein sequences from the NCBI database for the six outer membrane proteins- MSA-2c, AMA-1, SPAG-1, TASP, Vir-B10 and OMP-1 of the three parasites. All the acquired homologous protein sequences from BLASTp belong to *Babesia bovis* (taxid: 5865), *Theileria annulata* (taxid: 5874) and *Anaplasma marginale* (taxid: 770). Subsequently, the server CLUSTALW (https://www.ebi.ac.uk/Tools/msa/clustalo/) performed multiple sequence alignments between the homologous protein sequences of each protein. As a result, many different lengths of conserved regions were derived from each protein. The server, IEDB (Immune Epitope Database) was then utilized to predict both the MHC I and MHC II epitopes of each protein following the submission of the conserved region sequences to the server. The prediction of MHC I binding epitopes involved 105 BOLA alleles while the prediction of MHC II binding epitopes involved eight human HLA-DR alleles (HLA-DRB1*0301, HLA-DRB1*0401, HLA-DRB1*0801, HLA-DRB1*1101.HLA-DRB1*1301, HLA-DRB1*1401, HLA-DRB3*0101, and HLA-DRB3*0201) which possess a pseudo-sequence similarity with the BOLA alleles [86]. Numerous amounts of cytotoxic (MHC I) and helper T-cell (MHC II) epitopes were obtained from the IEDB server for each of the six proteins- MSA-2c, AMA-1, SPAG-1, TASP, Vir-B10 and OMP-1. High affinity epitopes regarding the large no. of alleles were chosen and used to measure their antigenicity. All epitopes with a percentile value less than 50 were selected. The tables of all the ten excellent MHC I epitopes of AMA-1, MSA-2c, SPAG-1, TASP, Vir-B10 and OMP-1 with their scores and percentile rankings representing their affinities for different BOLA alleles can be viewed in S2–S7 Files. The tables of all the ten most effective MHC II epitopes of AMA-1, MSA-2c, SPAG-1, TASP, Vir-B10 and OMP-1 with their scores and percentile rankings representing their affinities for different HLA alleles (pseudo-sequence similar to BoLA alleles) can be observed in S8–S13 Files. All the selected MHC I and MHC II epitopes with their highest achieved scores and lowest percentile ranks can be observed in Tables 3 and 4. The tables also exhibit the numbers of BoLA allele bonded to the selected peptides with percentile rank less than threshold value 50. It is clearly evident from Table 2 that the epitopes YLSGQSNEE, VILSSFFAE, GPGGNGEGG, TKASSSGDG, DGGGQGTDS, and ASGGSFEGK have bonded to 79, 48, 17, 11, 10, and 91 MHC I BoLA alleles, respectively with percentile rank less than 50 (threshold value). From these results we are confirmed that the MHC I epitope, YLSGQSNEE of **MSA-2c** (*Babesia bovis*), and ASGGSFEGK of **OMP1** (*Anaplasma marginale*) have bonded to all the allele members of the six BoLA supertypes. These analysis shows that these two epitopes have covered a high population of MHC I BoLA alleles and therefore, are promiscuous. On the other hand, the MHC I epitope, GPGGNGEGG of **SPAG-1** (*Theileria annulata*) has bonded to eight allele members of BoLA-2 supertype, three allele members of both BoLA-3 supertype and BoLA-1 supertype, and two allele members of BoLA-6 supertype. Since the epitope has bonded to some of the allele members of the four BoLA supertypes, this makes the epitope, GPGGNGEGG partially promiscuous. We can therefore, confirm that all the predicted MHC I epitopes represented in Table 3 will bind to BoLA MHC I alleles with high affinities. We can also observe from Table 4 that the MHC II epitopes, LEKNFEAVGMEATSA and PVILSSFFAEDALAS have bonded to five and three MHC II HLA alleles, respectively with percentile rank less than 50 (threshold value). None of the MHC II HLA alleles have bonded to the four MHC II epitopes, TDGTTTGPGGNGEGG, PTKASSSGDGAAPCH, GPSEDGGGQGTDSRF, and AQAAGGKLPGLLYPQ with percentile rank less than 50 (threshold value).

**Table 3:** The lowest percentile ranks and highest scores achieved for selected MHC I epitopes.

Position of MHC I epitope	Peptides selected for chimeric vaccine construction	Highest score achieved for binding to MHC I BoLA alleles	Lowest percentile rank achieved for binding to MHC I BoLA alleles	No. of binding MHC I BoLA alleles possessing percentile rank less than threshold value 50
**MSA-2c** 1–9	YLSGQSNEE	0.012596	10	79
**AMA-1** 3–11	VILSSFFAE	0.033706	5.7	48
**SPAG-1** 7–15	GPGGNGEGG	0.00151	18	17
**TASP** 2–10	TKASSSGDG	0.000782	39	11
**Vir-B10** 5–13	DGGGQGTDS	0.00091	29	10
**OMP-1** 12–20	ASGGSFEGK	0.682319	0.18	91

**Table 4:** The lowest percentile ranks and highest scores achieved for selected MHC II epitopes.

Position of MHC II epitope	Peptides selected for chimeric vaccine construction	Highest score achieved for binding to MHC II HLA alleles (pseudo-sequence similar to BoLA alleles)	Lowest percentile rank achieved for binding to MHC II HLA alleles (pseudo-sequence similar to BoLA alleles)	No. of binding MHC II HLA alleles possessing percentile rank less than threshold value 50
**MSA-2c** 1–15	LEKNFEAVGMEATSA	0.0986	16	5
**AMA-1** 2–16	PVILSSFFAEDALAS	0.0731	19	3
**SPAG-1** 1–15	TDGTTTGPGGNGEGG	0.0004	93	-
**TASP** 1–15	PTKASSSGDGAAPCH	0.0010	71	-
**Vir B-10** 1–15	GPSEDGGGQGTDSRF	0.0003	95	-
**OMP-1** 1–15	AQAAGGKLPGLLYPQ	0.0039	83	-

### 3.3 Building models of bovine MHC-II alleles, their refinement and stereochemical analysis

Eight bovine MHC II alleles were selected to download their protein sequences from the Immuno Polymorphism Database because these bovine alleles had pseudo-sequence similarity with the HLA MHC-II alleles that were selected to predict the MHC-II epitopes in IEDB. The BoLA alleles with their protein sequences and pseudo-sequence similar to HLA-alleles can be observed in S14 File. However, only four bovine MHC-II alleles such as BoLA-DRB3*016:01, BoLA-DRB3*4101, BoLA-DRB3*3301 and BoLA-DRB3*4301 were used for homology modelling due to their differences in amino acid length. The tertiary structure of BoLA-DRB3*016:01 was predicted by SWISS-MODEL [87] with the class II MHC DR beta chain (Accession No: A0A0P0QN76.1.A) of *Capra hircus* (Goat) as a template. BoLA-DRB3*016:01 showed 91.35% identity with the class II MHC DR beta chain of *Capra hircus* (Goat). After refinement, the quality factor of the 3D structure of BoLA-DRB3*016:01 was found to be 91.41 and the Ramachandran plot with favorable regions increased from 90.4% to 98.6%. The tertiary structure of BoLA-DRB3*4101 was predicted by SWISS-MODEL with MHC class II antigen (Accession No: A7XVM2.1.A) of *Ovis aries* (Sheep) as a template. BoLA-DRB3*4101 displayed 93.10% identity with MHC class II antigen of *Ovis aries* (Sheep). After refinement, the quality factor of the 3D structure of BoLA-DRB3*4101 increased from 96.0 to 97.26 and the Ramachandran plot possessing favorable regions increased from 93.7% to 97.5%. The tertiary structure of BoLA-DRB3*3301 was predicted by SWISS-MODEL with MHC class II antigen (Accession No: A0A0P0QN76.1.A) of *Ovis aries* (Sheep) as a template. BoLA-DRB3*3301 represented 91.35% identity with MHC class II antigen of *Ovis aries* (Sheep). After refinement, the quality factor of the 3D structure of BoLA-DRB3*3301 was found to be 97.70 and the Ramachandran plot containing favorable regions increased from 90.6% to 96.9%. The tertiary structure of BoLA-DRB3*4301 was predicted by SWISS-MODEL with MHC class II antigen (Accession No: A0A2N9DYY3.1.A) of *Bison bonasus* (European bison) as a template. BoLA-DRB3*4301 showed 92.13% identity with MHC class II antigen of *Bison bonasus* (European bison). After refinement, the quality factor of the 3D structure of BoLA-DRB3*4301 was found to be 91.54 and the Ramachandran plot with favorable regions was estimated to be 95.1%. The protein models of BoLA-DRB3*4101, BoLA-DRB3*4301, BoLA-DRB3*3301, and BoLA-DRB3*016:01were discovered to be high quality models after their refinement as it was observed that more than 90% of their amino acids were in the favorable core and allowed regions in the Ramachandran plot. The selected BoLA alleles with their templates, identity and coverage can be viewed from Table 5.

**Table 5:** Assessment of the features of templates used to predict the tertiary model of bovine MHC II alleles.

Alleles	Template used	Organism	GMQE (Global Model Quality Estimate)	Identity	Coverage
BoLA-DRB3*4101	MHC class II antigen	*Ovis aries* (Sheep)	0.98	93.10	1.00
BoLA-DRB3*4301	MHC class II antigen	*Bison bonasus* (European bison)	0.98	92.13	1.00
BoLA-DRB3*3301	MHC class II antigen	*Ovis aries* (Sheep)	0.92	89.81	1.00
BoLA-DRB3*016:01	Class II MHC DR beta chain	*Capra hircus* (Goat)	0.87	91.35	1.00

### 3.4 Bovine MHC II alleles interact strongly with MHC II epitopes

The two epitopes “LEKNFEAVGMEATSA” from **MSA-2c** and “PVILSSFFAEDALAS” from **AMA-1**, belonging to *Babesia bovis* possessed the lowest percentile score much below threshold value 50 when they bind to pseudo-sequence similar HLA-MHC II alleles. This gives us assurance that these epitopes will adhere to BoLA MHC II alleles strongly. Only four MHC II epitopes were selected from the total six IEDB predicted MHC II epitopes to conduct interactive studies with bovine MHC II alleles. These epitopes displayed their lowest percentile rank to be above 50 when interacting with pseudo-sequence similar HLA-MHC II alleles. All the MHC II HLA alleles have bonded to the four MHC II epitopes with percentile rank above 50 (threshold value). These epitopes included TDGTTTGPGGNGEGG, PTKASSSGDGAAPCH, GPSEDGGGQGTDSRF and AQAAGGKLPGLLYPQ. In order to stimulate an immune response, MHC II epitopes must bind strongly to bovine MHC II alleles. With the aim to observe the affinity between the bovine MHC II alleles and the four MHC II epitopes, molecular docking studies were performed using the server CABS-dock. The predicted epitope “PTKASSSGDGAAPCH” of *Theileria annulata* exhibited the strongest binding affinity with BoLA- DRB3*01601 of -114.19 kcal/mol while the epitope “GPSEDGGGQGTDSRF” of *Anaplasma marginale* exhibited highest binding affinity with BoLA- DRB3*01601 of -107.8 kcal/mol. The epitope “PTKASSSGDGAAPCH” of *Theileria annulata* displayed a high binding affinity with BoLA-DRB3*4101 of -62.87 kcal/mol whereas the predicted epitope “GPSEDGGGQGTDSRF” of *Anaplasma marginale* displayed the highest binding affinity with BoLA-DRB3*4101 of -74.82 kcal/mol. The predicted epitope “PTKASSSGDGAAPCH” of *Theileria annulata* showed a high binding affinity with BoLA-DRB3*3301 of -77.65 kcal/mol while the epitope “AQAAGGKLPGLLYPQ” of *Anaplasma marginale* showed the best binding affinity with BoLA-DRB3*3301 of -38.06 kcal/mol. The predicted epitope “TDGTTTGPGGNGEGG” of *Theileria annulata* exhibited highest binding affinity with BoLA-DRB3*4301 of -59.53 kcal/mol while the epitope “GPSEDGGGQGTDSRF” of *Anaplasma marginale* exhibited highest binding affinity of -85.38 kcal/mol. The docking of the four epitopes with the four bovine MHC II alleles; BoLA-DRB3*4101, BoLA-DRB3*4301, BoLA-DRB3*3301, and BoLA-DRB3*016:01 with high binding energy confirmed the stability and hence, the credibility of the complex. Thus, the MHC II (HTL) epitopes TDGTTTGPGGNGEGG, PTKASSSGDGAAPCH, GPSEDGGGQGTDSRF, AQAAGGKLPGLLYPQ were discovered to be potential peptides for generating effective HTL responses. Thus, we can conclude that all the IEDB predicted MHC II epitopes bind with high affinities to all the bovine MHC II alleles. The average protein-peptide (BoLA allele-MHC II epitopes) interaction energy and the average RMSD value between the bovine MHC II alleles and MHC II epitopes can be viewed from Table 6. The lower the RMSD value, the better the docking structures.

**Table 6:** Assessment of the protein-peptide interaction energy and average RMSD values for BoLA allele docking to the four MHC II epitopes.

BoLA alleles (Protein)	Name of rickettsial parasite	Position of MHC II epitopes	MHC II epitopes (Peptide)	Average RMSD value	Average protein peptide interaction energy (kcal/mol)
Interactive studies of BoLA-DRB3*01601 allele with MHC II epitopes					
BoLA- DRB3*01601	*Theileria annulata*	**SPAG-1** 1–15	TDGTTTGPGGNGEGG	2.38098	-103.4
		**TASP** 1–15	PTKASSSGDGAAPCH	1.54576	-114.19
	*Anaplasma marginale*	**Vir B-10** 1–15	GPSEDGGGQGTDSRF	2.201	-107.8
		**OMP-1** 1–15	AQAAGGKLPGLLYPQ	1.91503	-75.47
Interactive studies of BoLA-DRB3*4101 allele with MHC II epitopes					
BoLA-DRB3*4101	*Theileria annulata*	**SPAG-1** 1–15	TDGTTTGPGGNGEGG	5.41114	-34.23
		**TASP** 1–15	PTKASSSGDGAAPCH	2.35767	-62.87
	*Anaplasma marginale*	**Vir B-10** 1–15	GPSEDGGGQGTDSRF	5.04788	-74.82
		**OMP-1** 1–15	AQAAGGKLPGLLYPQ	3.87423	-46.13
Interactive studies of BoLA-DRB3*3301 allele with MHC II epitopes					
BoLA-DRB3*3301	*Theileria annulata*	**SPAG-1** 1–15	TDGTTTGPGGNGEGG	1.00893	-34.35
		**TASP** 1–15	PTKASSSGDGAAPCH	4.29596	-77.65
	*Anaplasma marginale*	**Vir B-10** 1–15	GPSEDGGGQGTDSRF	5.53753	-31.34
		**OMP-1** 1–15	AQAAGGKLPGLLYPQ	3.07927	-38.06
Interactive studies of BoLA-DRB3*4301 allele with MHC II epitopes					
BoLA-DRB3*4301	*Theileria annulata*	**SPAG-1** 1–15	TDGTTTGPGGNGEGG	4.79131	-59.53
		**TASP** 1–15	PTKASSSGDGAAPCH	2.88721	-41.51
	*Anaplasma marginale*	**Vir B-10** 1–15	GPSEDGGGQGTDSRF	2.00049	-85.38
		**OMP-1** 1–15	AQAAGGKLPGLLYPQ	4.08312	-45.86

### 3.5 Both the conserved regions and T-cell epitopes of the outer membrane proteins are screened for their antigenicity and transmembrane topology

The conserved regions were subjected to antigenicity and transmembrane topology screening before submission to IEDB. The conserved regions with their antigenicity scores and location can be observed in S15 File. Only the highly immunogenic conserved regions and those predicted to be present on the cell’s outer membrane were selected for submission to IEDB. Consequently, the MHC I binding epitopes and the MHC II binding epitopes with the highest probable antigenic scores and located outside the cell were predicted by servers, VaxiJen v2.0 (http://www.ddg-pharmfac.net/vaxijen/VaxiJen/VaxiJen.html) and DeepTMHMM v. 1.0 (https://dtu.biolib.com/DeepTMHMM) [88] respectively, were selected.

### 3.6 Assessment of allergenicity, toxicity and conservancy of individual epitopes

Three servers were required for the detection of allergenicity of each of the MHC I and MHC II epitopes- ALLERTOP v. 2.0. AllergenFP v.1.0., and Allermatch v.1.0.. Confirmation was needed from at least two servers to predict non-allergenic epitopes. These non-allergenic epitopes were then selected to undergo toxicity analysis. The server, ToxinPred v 1.0 used for the toxicity examination of every non-allergenic epitope, declared the epitopes non-toxic. Finally, all the epitopes of the six outer membrane proteins of the three parasites were individually analyzed for their conservancy. It was found that all the epitopes from MSA-2c and AMA-1 were extremely conserved within all the different strains of *B*. *bovis*, epitopes from SPAG-1 and TASP were highly conserved within all the different strains of *T*. *annulata* and Vir-B10 and OMP-1 were incredibly conserved within all the different strains of *A*. *marginale*. All the epitopes had a conservancy measure of 100%. We then selected the top ten MHC I and MHC II epitopes with high antigenic scores for each of the six proteins. This can be viewed in S16–S27 Files. Following that, the epitope with the highest antigen score of all was selected from each of the ten epitopes of the individual proteins. Lastly, the top six MHC I epitopes with the highest antigenic score, located outside the cell membrane, non-allergenic, non-toxic and possessing 100% conservancy were selected from MSA-2c, AMA-1, SPAG-1, TASP, Vir-B10, and OMP-1. Subsequently, the best six MHC II epitopes with the highest antigenic score, located outside the cell membrane, non-allergenic, non-toxic and possessing 100% conservancy were also selected from MSA-2c, AMA-1, SPAG-1, TASP, Vir-B10, and OMP-1. Tables 7 and 8 respectively represent antigenicity scores, transmembrane topology, allergenicity, conservancy along with toxicity analysis of both MHC I and MHC II epitopes.

**Table 7:** Antigenicity prediction, screening of transmembrane topology, allergenicity, conservancy, along with toxicity assessment of the six best major histocompatibility complex class I epitopes.

Name of protein	MHC I epitopes	Start	End	Length	No. of BOLAs* binding epitopes	Antigenicity score	Allergenicity	Toxicity	Conservancy
AMA-1 (*B*. *bovis*)	VILSSFFAE	3	11	9	98	2.168	Probable non-allergen	Non-toxin	100.00%
MSA-2c (*B*. *bovis*)	YLSGQSNEE	1	9	9	294	1.5966	Probable non-allergen	Non-toxin	100.00%
TASP (*T*. *annulata*)	TKASSSGDG	2	10	9	98	2.6063	Probable non-allergen	Non-toxin	100.00%
SPAG-1 (*T*. *annulata*)	GPGGNGEGG	7	15	9	98	4.0758	Probable non-allergen	Non-toxin	100.00%
Vir-B10 *marginale*)	DGGGQGTDS	5	13	9	98	2.6052	Probable non-allergen	Non-toxin	100.00%
OMP-1 *marginale*)	ASGGSFEGK	12	20	9	98	2.2830	Probable non-allergen	Non-toxin	100.00%

*BOLA- Bovine Leukocyte Antigen.

**Table 8:** Antigenicity prediction, screening of transmembrane topology, allergenicity, conservancy along with toxicity assessment of the six best major histocompatibility complex class II epitopes.

Name of protein	MHC II Epitopes	Start	End	Length	No. of BOLAs* binding epitopes	Antigenicity score	Allergenicity	Toxicity	Conservancy
**AMA-1** (*B*. *bovis*)	PVILSSFFAEDALAS	2	16	15	8	1.2864	Probable non-allergen	Non-toxin	100.00%
**MSA-2c** (*B*. *bovis*)	LEKNFEAVGMEATSA	1	15	15	8	0.5764	Probable non-allergen	Non-toxin	100.00%
**TASP** (*T*. *annulata*)	PTKASSSGDGAAPCH	1	15	15	8	1.8933	Probable non-allergen	Non-toxin	100.00%
**SPAG-1** (*T*. *annulata*)	TDGTTTGPGGNGEGG	1	15	15	8	2.9595	Probable non-allergen	Non-toxin	100.00%
**Vir-B10** *(A*. *marginale*)	GPSEDGGGQGTDSRF	1	15	15	8	2.1276	Probable non-allergen	Non-toxin	100.00%
**OMP-1** *(A*. *marginale*)	AQAAGGKLPGLLYPQ	1	15	15	8	1.2848	Probable non-allergen	Non-toxin	100.00%

*BOLA- Bovine Leukocyte Antigen.

### 3.7 Three algorithm methods were used to predict B-cell epitopes

**Fig 2:**
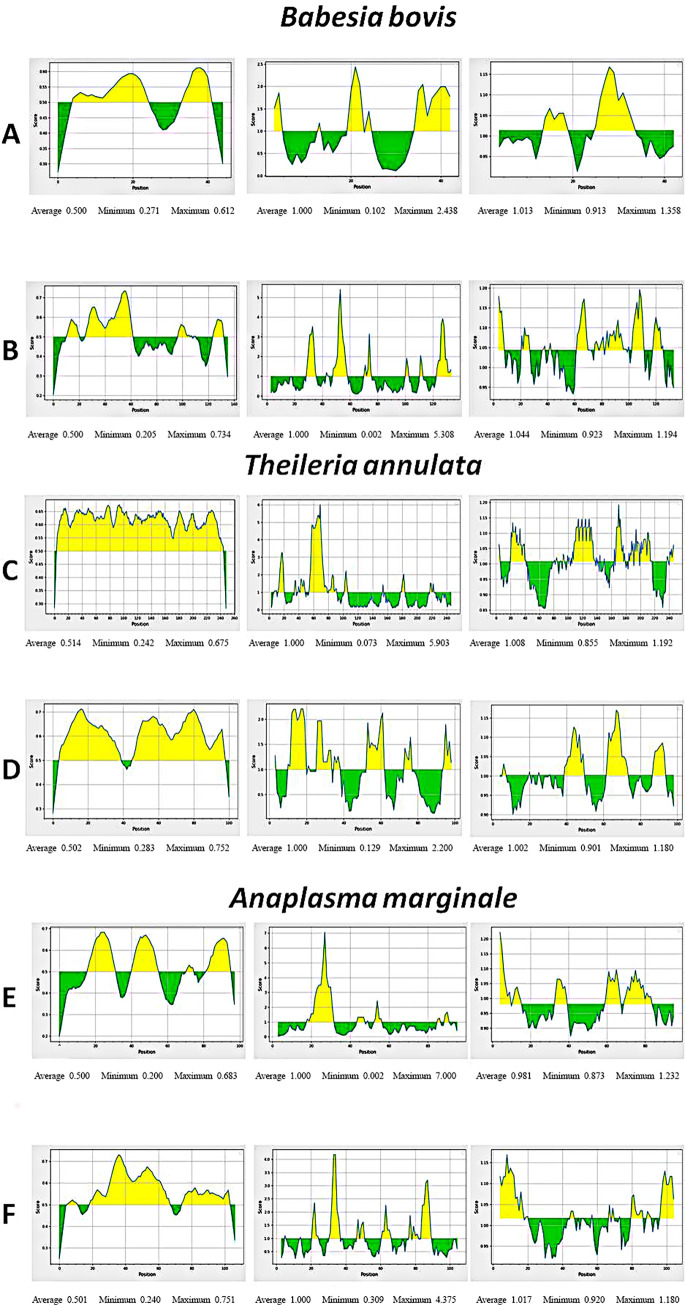
Three algorithm methods for b-cell epitope prediction for the two outer membrane proteins of *Babesia bovis*- MSA-2c(A), AMA-1(B). The X-axis of each graph displays position while the y-axis shows the score. Epitopes lying above the threshold value are situated in yellow color. Most favored position is represented by the longest peak found in yellow color. Bepipred linear Epitope Prediction Method (Left column), Emini Surface Accessibility Prediction Method (Centre), and Kolaskar and Tongaonkar Prediction Method (Right column).

**Fig 3:**
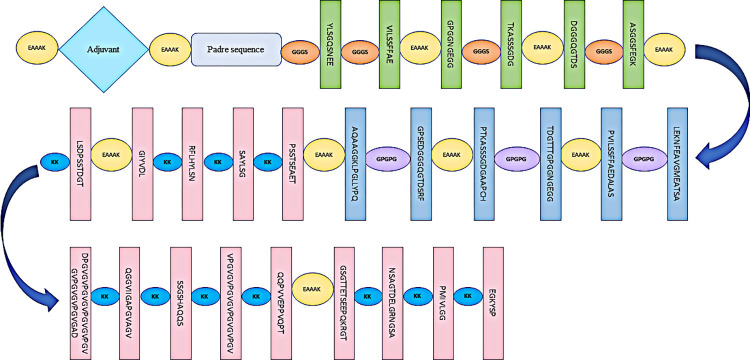
Three algorithm methods for b-cell epitope prediction for the two outer membrane proteins of *Theileria annulata*- SPAG-1(C), TASP(D). The X-axis of each graph displays position while the y-axis shows the score. Epitopes lying above the threshold value are situated in yellow color. Most favored position is represented by the longest peak found in yellow color. Bepipred linear Epitope Prediction Method (Left column), Emini Surface Accessibility Prediction Method (Centre), and Kolaskar and Tongaonkar Prediction Method (Right column).

**Fig 4:**
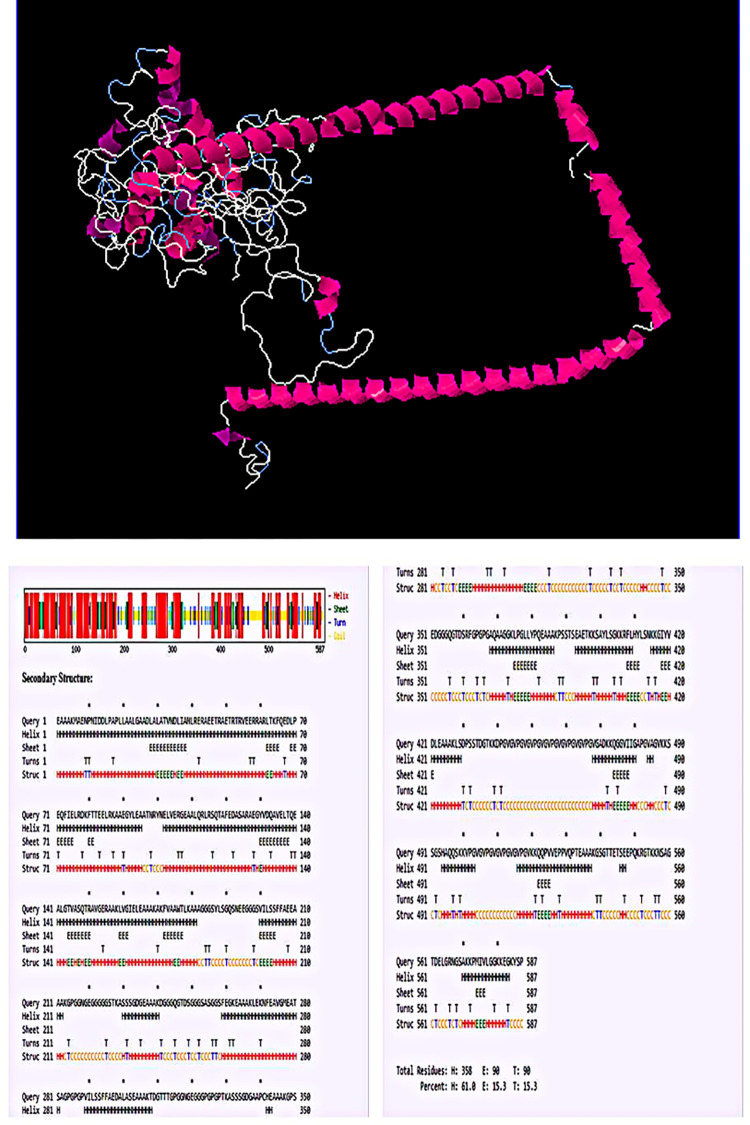
Three algorithm methods for b-cell epitope prediction for the two outer membrane proteins of *Anaplasma marginale*- Vir-B10(E) and OMP1(F). The X-axis of each graph displays position while the y-axis shows the score. Epitopes lying above the threshold value are situated in yellow color. Most favored position is represented by the longest peak found in yellow color. Bepipred linear Epitope Prediction Method (Left column), Emini Surface Accessibility Prediction Method (Centre), and Kolaskar and Tongaonkar Prediction Method (Right column).

B-cell epitopes were predicted for each of the six outer membrane proteins belonging to *B*. *bovis*, *T*. *annulata* and *A*. *marginale*. Three algorithm methods from IEDB were executed to perform B-cell epitope prediction. The epitopes which are predicted by Bepipred linear 2.0 prediction method and possessed residue scores above 0.5 were selected. The epitopes which are predicted by Kolaskar and Tongaonkar antigenicity method and had residue scores above 1.00 were selected. The epitopes which were predicted by Emini surface accessibility prediction algorithm method and have acquired residue scores above 1.00 were chosen. The predicted b-cell epitopes with the scores of their residues can be viewed in S28 File. For MSA-2c (*B*. *bovis*), Bepipred linear 2.0 prediction method showed the peptide sequence 34–42 to be antigenic, located outside the cell and is non-allergenic. Kolaskar and Tongaonkar antigenicity results predicted the peptide sequence 25–33 to be antigenic, located outside the cell and non-allergenic. The result of this method provides approval that these epitopes have the capacity to work as strong b-cell inducers. Emini surface accessibility prediction algorithm method does not show any peptide sequences for MSA-2c (S29 File). For AMA-1 (*B*. *bovis*), Bepipred linear 2.0 prediction method displayed the peptide sequences 12–21 to be non-antigenic; peptide sequences from 98–108, 125–133 and 48–58 were predicted to be antigenic but were located outside the cell. Hence, no probable peptide sequences were predicted for AMA-1 by the algorithm method. Kolaskar and Tongaonkar antigenicity results showed that the peptide sequence from 118–125 to be antigenic, located outside the cell and is non-allergenic. Other epitopes with very high antigenic scores were observed to be found inside the cell. The Emini surface accessibility prediction algorithm displayed the peptide sequence from 48–58 to be highly antigenic however, it is located inside the cell (S30 File). For SPAG-1 (*T*. *annulata*), Bepipred linear 2.0 prediction method did not display any probable peptide sequences that could efficiently work as b-cell epitopes. Kolaskar and Tongaonkar antigenicity results confirmed the peptide sequences from 200–213, 267–275, and 290–308 to be extremely antigenic, located outside the cell and non-allergenic. Emini surface accessibility prediction algorithm method predicted the peptide sequence from 415–424 to be accessible, antigenic, located outside the cell and is non-allergenic (S31 File). For TASP (*T*. *annulata*), Bepipred linear 2.0 prediction method did not predict any peptide sequences capable to work as efficient b-cell epitopes. Kolaskar and Tongaonkar antigenicity results displayed a peptide sequence from 62–73 to be highly antigenic, located outside the cell and is non allergenic. Emini Surface accessibility prediction result confirmed the peptide sequence 53–61 to be antigenic and present outside the cell but was later found to be allergenic (S32 File). For Vir-B10 (*A*. *marginale*), Bepipred linear epitope prediction method 2.0 predicted two peptide sequences from 17–32 and 83–96 to be capable of working as efficient b-cell epitopes. They have the highest antigenicity score, located outside the cell and are non-allergenic. Kolaskar and Tongaonkar antigenicity result predicted the peptide sequence 33–39 to be highly antigenic, located outside the cell and is non-allergenic. Emini surface accessibility prediction method displayed antigenic epitopes that are accessible but are located inside the cell (S33 File). For OMP1 (*A*. *marginale*), Bepipred linear 2.0 prediction method and Kolaskar and Tongaonkar antigenicity results did not predict any peptide sequences that were capable of inducing an immune response. Emini surface accessibility prediction confirmed that the peptide sequence from 61–66 to be more accessible and highly antigenic (S34 File). The best b-cell epitopes with their antigenic scores, transmembrane topology and allergenicity assessment results are shown in Table 9. All the figures of the Bepipred linear 2.0 prediction method, Kolaskar and Tongaonkar antigenicity results and Emini surface accessibility prediction for the six proteins- *Babesia bovis* MSA-2c (A), AMA-1 (B), *Theileria annulata* SPAG-1 (C), TASP (D) and *Anaplasma marginale* Vir-B10 (E), OMP1 (F) can be observed in Figs 2–4.

**Table 9:** Antigenicity prediction, screening of transmembrane topology, allergenicity, conservancy along with toxicity assessment of the best b-cell epitopes generated from each of the six proteins AMA-1, MSA-2c (*Babesia bovis*) TASP, SPAG-1 (*Theileria annulata*) and Vir-B10, OMP-1 (*Anaplasma marginale*).

Name of microorganism	Protein	Start	End	Length	Peptides	Antigenicity score	TMHMM	Allergenicity
*Babesia bovis*	**MSA-2c**	34	42	9	PSSTSEAET	0.8456	Outside	Probable non-allergen
		14	19	6	SAYLSG	2.8306	Outside	Probable non-allergen
	**AMA-1**	118	125	8	RFLHYLSN	0.4342	Outside	Probable non-allergen
		21	26	6	GIYVDL	2.6873	Outside	Probable non-allergen
*Theileria annulata*	**SPAG-1**	415	424	10	LSDPSSTDGT	0.4221	Outside	Probable non-allergen
		106	137	32	DPGVGVPGVGVPGVGVPGVGVPGVGVPGVGAD	1.1916	Outside	Probable non-allergen
		200	213	14	QGGVIIGAPGVAGV	0.7409	Outside	Probable non-allergen
		267	275	9	SSGSHAQQS	1.5257	Outside	Probable non-allergen
		290	308	19	VPGVGVPGVGVPGVGVPGV	1.0606	Outside	Probable non-allergen
	**TASP**	62	73	12	QQPVVEPPVQPT	0.7532	Outside	Probable non-allergen
*Anaplasma marginale*	**Vir-B10**	17	32	16	GSGTTETSEEPQKRGT	1.2386	Outside	Probable non-allergen
		83	96	14	NSAGTDELGRNGSA	0.6728	Outside	Probable non-allergen
		33	39	7	PMIVLGG	0.5947	Outside	Probable non-allergen
	**OMP1**	61	66	6	EGKYSP	0.6216	Outside	Probable non-allergen

### 3.8 Three chimeric vaccine sequences were constructed

**Fig 5:**
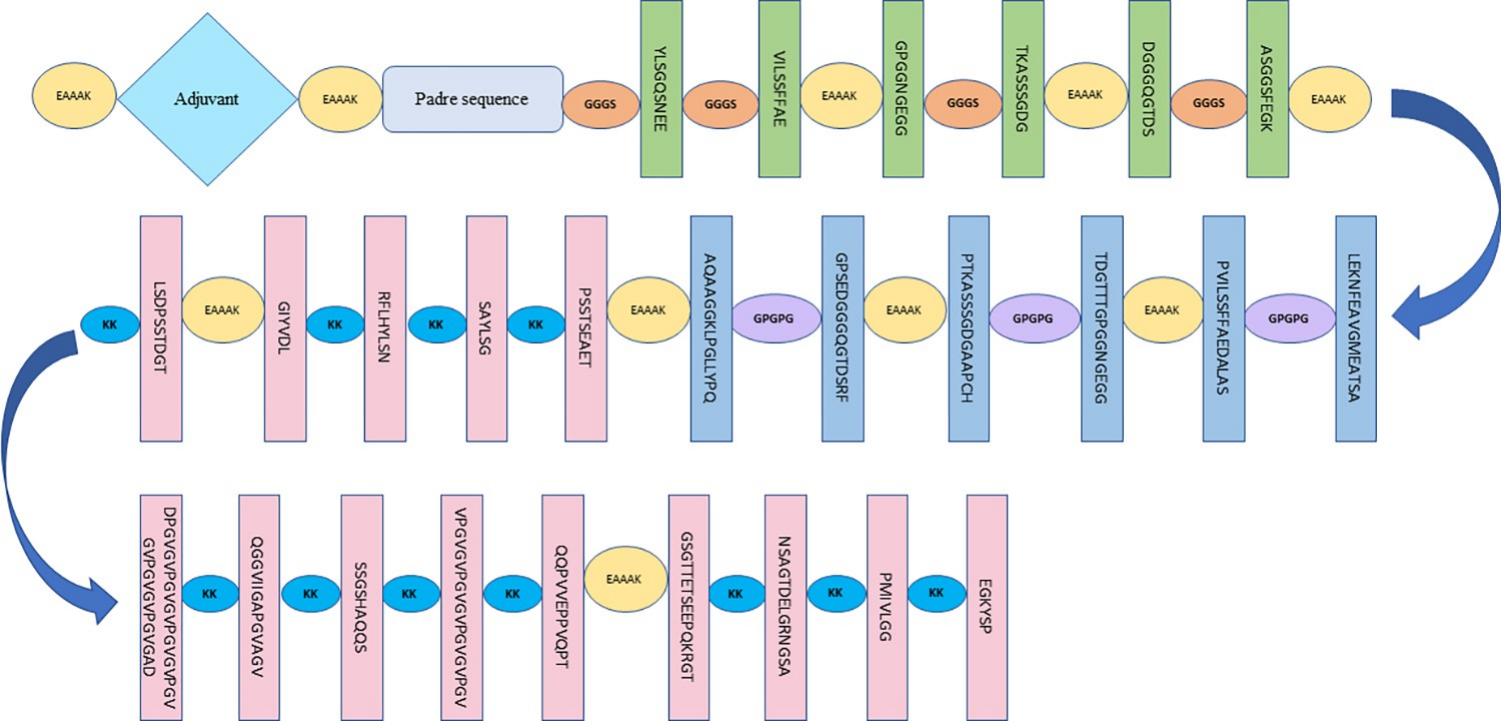
Graphical illustration of the newly designed chimeric vaccine construct.

Three vaccine sequences V1, V2 and V3 were modeled in this study possessing a different adjuvant for each chimeric vaccine. These adjuvants included bovine beta-defensin, L7/L12 ribosomal protein and HBHA protein. The bovine beta-defensin was incorporated as an adjuvant in V1 because it is known to activate dendritic cells and produce cytokines such as IL-12, IFN-, and IL-6, which in turn stimulate the Th1 response in cattle [89]. In the vaccine construct V2, L7/L12 ribosomal protein was incorporated as an adjuvant since it stimulates the immune response. This includes activating monocytes in animals and accelerating IFN-y expression. L7/L12 ribosomal protein-based vaccines are also unaffected by infections. Therefore, it has been used as adjuvants in lots of vaccines [90]. HBHA protein is used as an adjuvant in the vaccine construct, V3 to increase Th1 and Th17 immune responses [91]. The presence of adjuvants in vaccines accelerates the immunogenicity of vaccines, making them more efficient. The adjuvants were separated from the PADRE sequence by the linker EAAAK. The PADRE sequence is incorporated to enhance the efficiency and effectiveness of vaccine proteins. The addition of PADRE sequences also accelerates the activation of an increased number of cytotoxic T-cells than those vaccines without the PADRE sequence [92]. We added PADRE sequences after adjuvants to avoid the problems arising from polymorphic BOLA alleles [93]. The vaccine constructs also contained T-cell epitopes and B-cell epitopes connected by different linkers. Each chimeric vaccine construct consisted of six MHC I epitopes and six MHC II epitopes belonging to the six outer membrane proteins (two from *Babesia bovis*, two from *Theileria annulata* and two from *Anaplasma marginale*). Total of 19 B-cell epitopes from the three parasites were also added. Each chimeric vaccine construct consisted of 31 epitopes. Table 10 shows adjuvants, epitopes and linkers used in vaccine construction. All CTL epitopes, HTL epitopes, and BCL epitopes were joined together by GGGS, GPGPG, and KK linkers, respectively. The aim of the addition of linkers to the chimeric vaccines was to have sufficient separation between the epitopes in vivo. In addition, it was to establish an effective immune response [94]. The linker, EAAAK, separated epitopes from different parasites. The linker, EAAAK, was also used to separate T-cell epitopes and B-cell epitopes of one parasite from other parasites. The designed chimeric vaccine constructs V1, V2 and V3 were 492, 558 and 587 amino acids long, respectively. The graphical illustration of the designed chimeric vaccine construct is shown in (Fig 5).

**Table 10:** The best epitopes used for the vaccine construction along with adjuvants and linkers.

Name of Microorganism	MHC I epitopes	MHC II epitopes	B-cell epitopes	Adjuvants	PADRE sequence	Linkers
***Babesia bovis***	YLSGQSNEE	LEKNFEAVGMEATSA	PSSTSEAET	MRLHHLLLVLLFLVLSAGSGFTQVVRNPQSCRWNMGVCIPISCPGNMRQIGTCFGPRVPCCRRW	AKFVAAWTLKAAA	GGGS
	VILSSFFAE	PVILSSFFAEDALAS	SAYLSG	MAKLSTDELLDAFKEMTLLELSDFVKKFEETFEVTAAAPVAVAAAGAAPAGAAVEAAEEQSEFDVILEAAGDKKIGVIKVVREIVSGLGLKEAKDLVDGAPKPLLEKVAKEAADEAKAKLEAAGATVTVK		GPGPG
			RFLHYLSN			
			GIYVDL	MAENPNIDDLPAPLLAALGAADLALATVNDLIANLRERAEETRAETRTRVEERRARLTKFQEDLPEQFIELRDKFTTEELRKAAEGYLEAATNRYNELVERGEAALQRLRSQTAFEDASARAEGYVDQAVELTQEALGTVASQTRAVGERAAKLVGIEL		EAAAK
***Theileria annulata***	GPGGNGEGG	TDGTTTGPGGNGEGG	LSDPSSTDGT			
	TKASSSGDG	PTKASSSGDGAAPCH	DPGVGVPGVGVPGVGVPGVGVPGVGVPGVGAD			KK
			QGGVIIGAPGVAGV			
			SSGSHAQQS			
			VPGVGVPGVGVPGVGVPGV			
			QQPVVEPPVQPT			
***Anaplasma marginale***	DGGGQGTDS	GPSEDGGGQGTDSRF	GSGTTETSEEPQKRGT			
	ASGGSFEGK	AQAAGGKLPGLLYPQ	NSAGTDELGRNGSA			
			PMIVLGG			
			EGKYSP			

### 3.9 Assessment of allergenicity, antigenicity and solubility of individual chimeric vaccine constructs

The server, AlgPred v.2.0 is used to test for allergenicity of all chimeric vaccines. It was observed that the chimeric vaccines V1 and V3 were non-allergenic with values of -0.4557 and -0.5019, respectively. The chimeric vaccine V2 was found to be allergenic with an allergenicity score of 0.0778. The adjuvant, L7/L12 ribosomal protein was therefore not suitable for the chimeric vaccine. However, the chimeric vaccines containing bovine beta-defensin in V1 and HBHA protein in V3 as adjuvants were suitable. VaxiJen v2.0 server was utilized for the measuring of the antigenicity of the three chimeric vaccines. The antigenicity score of the chimeric vaccines V1, V2 and V3 was 1.0310, 0.9124 and 0.9978 respectively. This assures that all the chimeric vaccines are highly antigenic and V1 containing bovine beta-defensin as the adjuvant possessed the highest antigenic score. The solubility score was determined by the Protein Sol-software which proved that all the chimeric vaccines are soluble, having the scores above the threshold value of 0.45. The solubility scores of the chimeric vaccines V1 were 0.592, V2 was 0.595 while V3 possessed the highest solubility score of 0.646. The protein sol-software confirms that all chimeric vaccines are soluble upon overexpression in E. coli. The list includes all the scores of antigenicity, allergenicity and solubility related to the chimeric vaccines in Table 11.

**Table 11:** Allergenicity assessment, antigenicity prediction and solubility analysis of the three constructed vaccines.

Vaccine Constructs	Composition	Complete sequence of Vaccine constructs	Allergenicity (AlgPred) Threshold -0.4	Vaxijen score (Threshold 0.4)	Solubility Threshold (0.45)
**V1**	Predicted CTL, HTL & BCL epitopes of three microorganisms-*B*. *bovis*, *T*. *annulata*, and *A*. *marginale* with Bos Taurus β defensin adjuvant & PADRE sequence	**EAAAK**MRLHHLLLVLLFLVLSAGSGFTQVVRNPQSCRWNMGVCIPISCPGNMRQIGTCFGPRVPCCRRW**EAAAK**AKFVAAWTLKAAA**GGGS**YLSGQSNEE**GGGS**VILSSFFAE**EAAAK**GPGGNGEGG**GGGS**TKASSSGDG**EAAAK**DGGGQGTDS**GGGS**ASGGSFEGK**EAAAK**LEKNFEAVGMEATSA**GPGPG**PVILSSFFAEDALAS**EAAAK**TDGTTTGPGGNGEGG**GPGPG**PTKASSSGDGAAPCH**EAAAK**GPSEDGGGQGTDSRF**GPGPG**AQAAGGKLPGLLYPQ**EAAAK**PSSTSEAET**KK**SAYLSG**KK**RFLHYLSN**KK**GIYVDL**EAAAK**LSDPSSTDGT**KK**DPGVGVPGVGVPGVGVPGVGVPGVGVPGVGAD**KK**QGGVIIGAPGVAGV**KK**SSGSHAQQS**KK**VPGVGVPGVGVPGVGVPGV**KK**QQPVVEPPVQPT**EAAAK**GSGTTETSEEPQKRGT**KK**NSAGTDELGRNGSA**KK**PMIVLGG**KK**EGKYSP	**-0.4557** **(Non-allergen)**	** 1.0310 **	**0.592**
**V2**	Predicted CTL, HTL & BCL epitopes of three microorganisms-*B*. *bovis*, *T*. *annulata*, and *A*. *marginale* with L7/L12 ribosomal protein adjuvant & PADRE sequence	**EAAAK**MAKLSTDELLDAFKEMTLLELSDFVKKFEETFEVTAAAPVAVAAAGAAPAGAAVEAAEEQSEFDVILEAAGDKKIGVIKVVREIVSGLGLKEAKDLVDGAPKPLLEKVAKEAADEAKAKLEAAGATVTVK**EAAAK**AKFVAAWTLKAAA**GGGS**YLSGQSNEE**GGGS**VILSSFFAE**EAAAK**GPGGNGEGG**GGGS**TKASSSGDG**EAAAK**DGGGQGTDS**GGGS**ASGGSFEGK**EAAAK**LEKNFEAVGMEATSA**GPGPG**PVILSSFFAEDALAS**EAAAK**TDGTTTGPGGNGEGG**GPGPG**PTKASSSGDGAAPCH**EAAAK**GPSEDGGGQGTDSRF**GPGPG**AQAAGGKLPGLLYPQ**EAAAK**PSSTSEAET**KK**SAYLSG**KK**RFLHYLSN**KK**GIYVDL**EAAAK**LSDPSSTDGT**KK**DPGVGVPGVGVPGVGVPGVGVPGVGVPGVGAD**KK**QGGVIIGAPGVAGV**KK**SSGSHAQQS**KK**VPGVGVPGVGVPGVGVPGV**KK**QQPVVEPPVQPT**EAAAK**GSGTTETSEEPQKRGT**KK**NSAGTDELGRNGSA**KK**PMIVLGG**KK**EGKYSP	**0.0778** **(Allergen)**	** 0.9124 **	**0.595**
**V3**	Predicted CTL, HTL & BCL epitopes of three microorganisms-*B*. *bovis*, *T*. *annulata*, and *A*. *marginale* with HBHA protein adjuvant & PADRE sequence	**EAAAK**MAENPNIDDLPAPLLAALGAADLALATVNDLIANLRERAEETRAETRTRVEERRARLTKFQEDLPEQFIELRDKFTTEELRKAAEGYLEAATNRYNELVERGEAALQRLRSQTAFEDASARAEGYVDQAVELTQEALGTVASQTRAVGERAAKLVGIEL**EAAAK**AKFVAAWTLKAAA**GGGS**YLSGQSNEE**GGGS**VILSSFFAE**EAAAK**GPGGNGEGG**GGGS**TKASSSGDG**EAAAK**DGGGQGTDS**GGGS**ASGGSFEGK**EAAAK**LEKNFEAVGMEATSA**GPGPG**PVILSSFFAEDALAS**EAAAK**TDGTTTGPGGNGEGG**GPGPG**PTKASSSGDGAAPCH**EAAAK**GPSEDGGGQGTDSRF**GPGPG**AQAAGGKLPGLLYPQ**EAAAK**PSSTSEAET**KK**SAYLSG**KK**RFLHYLSN**KK**GIYVDL**EAAAK**LSDPSSTDGT**KK**DPGVGVPGVGVPGVGVPGVGVPGVGVPGVGAD**KK**QGGVIIGAPGVAGV**KK**SSGSHAQQS**KK**VPGVGVPGVGVPGVGVPGV**KK**QQPVVEPPVQPT**EAAAK**GSGTTETSEEPQKRGT**KK**NSAGTDELGRNGSA**KK**PMIVLGG**KK**EGKYSP	**-0.5019** **(Non-allergen)**	** 0.9978 **	**0.646**

### 3.10 Determination of the physicochemical properties and the prediction of the secondary structure of the chimeric vaccine constructs

In determining the stability of chimeric vaccines, ExPasy’s Protparam tool is observed to be the most valuable. This server was actually used to predict all the physicochemical properties of vaccines. The molecular weight of the chimeric vaccine constructs, V1 was confirmed to be 48KDa with the instability index computed to be above 40 i.e., 41.18. Thus, confirming that the protein will be unstable. The chimeric vaccine V1 has the highest antigenic score but could not be used as an effective vaccine. This is because it will degrade in the long run and will lose its antigenicity with the approaching time. The chimeric vaccine constructs V2 and V3 possess a molecular weight of 54KDa and 58KDa with instability indexes below 40 (V2- 34.18 and V3- 39.93). This proves that both V2 and V3 vaccines are stable over time. However, the chimeric vaccine construct, V2 was confirmed to be allergenic in the past. Compared to other chimeric vaccines, V3 with excellent antigenicity, non-allergenicity, solubility, and stability scores proved to be the most effective. Subsequently, after the prediction of physicochemical characteristics of the chimeric vaccine, it was revealed that the chimeric vaccine V3 consisting of the adjuvant HBHA protein is expected to be stable. Hence, the chimeric vaccine V3 was confirmed to be the highest-quality vaccine. The theoretical pI of the vaccine construct V3 was calculated to be 5.44 which assures that V3 will acquire a net negative charge above this pI and vice versa. The extinction coefficient of V3 was 18910 assuming all cysteine residues form cystines. The estimated half-life of the chimeric vaccine V3 was predicted to be 1 hour within mammals’ reticulocytes in vitro and more than 10 hours in *E*. *coli* in vivo. The aliphatic index of the chimeric vaccine was found to be 64.65 which suggests that the vaccine V3 is thermally stable. The negative value of the grand average of hydropathicity (GRAVY) of V3 is -0.467, showing that the vaccine is hydrophilic. All the physical properties of the chimeric vaccines are presented in Table 12. The secondary structure of the chimeric vaccine V3 was predicted by CFSSP. In its structure, there are 61.0% alpha helixes, 15.3% beta sheets, and 15.3% turns. The secondary structure of V3 predicted by PSIPRED v4.0 server is shown in S1 Fig.

**Table 12:** Physicochemical characteristics of the three chimeric vaccine constructs.

Vaccine Constructs	Isoelectric Point	Extinction Co-efficient	Instability Index	Aliphatic Index	Hydropathicity (GRAVY)	No. of Amino acids
**V1**	9.02	25815	41.18	60.16	-0.348	492
**V2**	5.48	14440	34.18	66.24	-0.308	558
**V3**	5.44	18910	39.93	64.65	-0.467	587

### 3.11 Three-dimensional structure prediction, refinement and disulfide engineering of the chimeric vaccine construct

**Fig 6:**
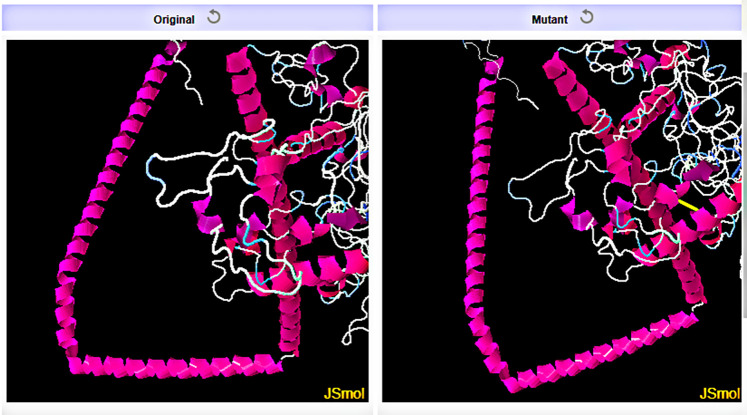
Structure prediction of the chimeric vaccine, V3. (A) Three-dimensional structure of the chimeric vaccine construct, V3 (ribbon model) predicted by the RaptorX server. This shows that the construct consists of three domains. The three types are: alpha-helix, beta sheet, and coils. (B) Secondary structure of the chimeric vaccine construct, V3 predicted by CFSSP server. This shows that the construct V3 consists of 61.0% of Helix, 15.3% of Beta-pleated sheet and 15.3% of Turns in their structure. There are a total of 358 helix residues, 90 Beta-sheet residues and 90 Turn residues. The letter ‘H’ is denoted for helix. The letter ‘E’ is denoted for Beta-sheet and the letter ‘T’ is denoted for turns.

**Fig 7:**
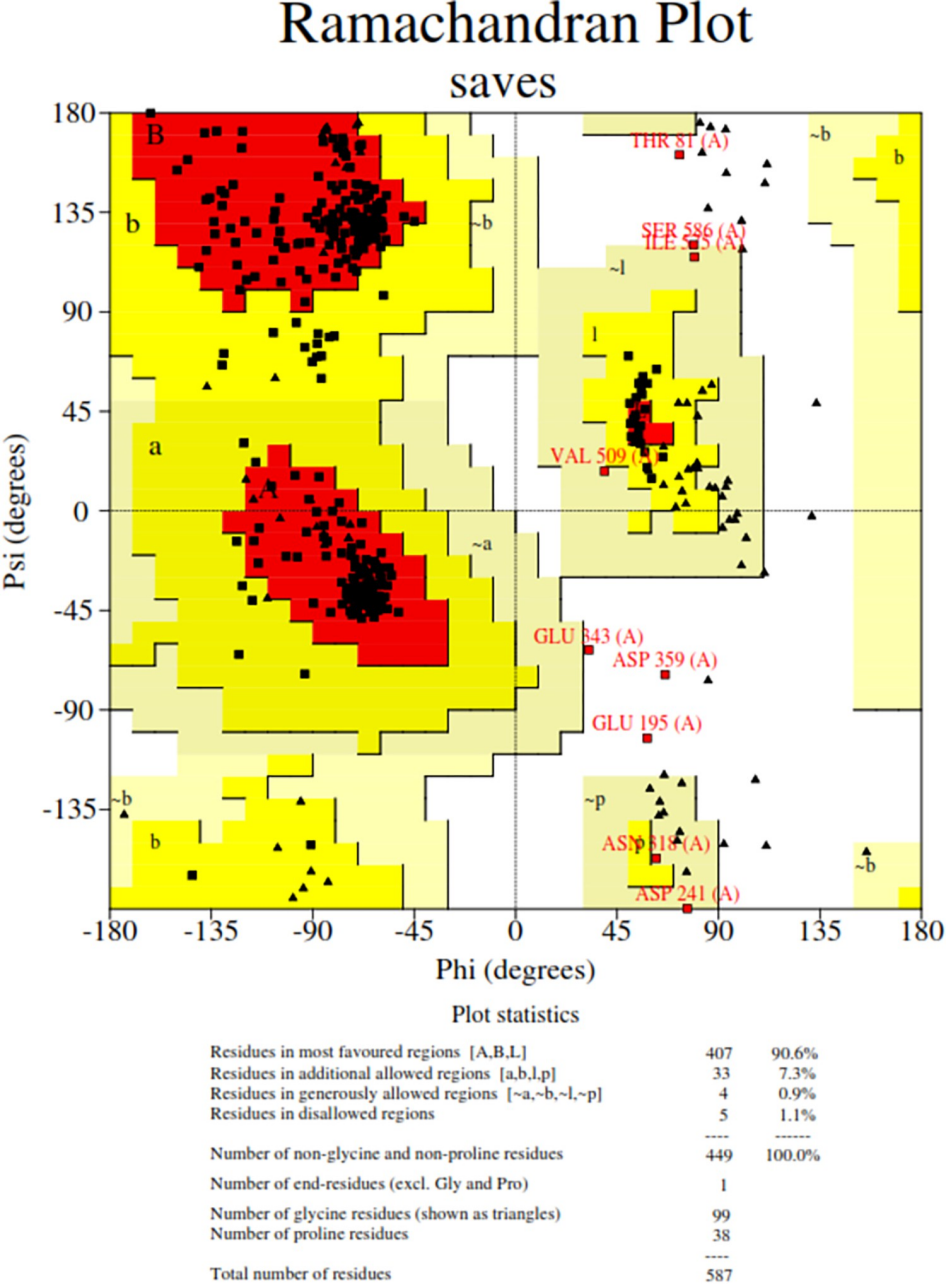
Ramachandran plot analysis of the chimeric vaccine construct, V3 (after refinement) predicted by the PROCHECK program of the SAVE v6.1 server.

**Fig 8:**
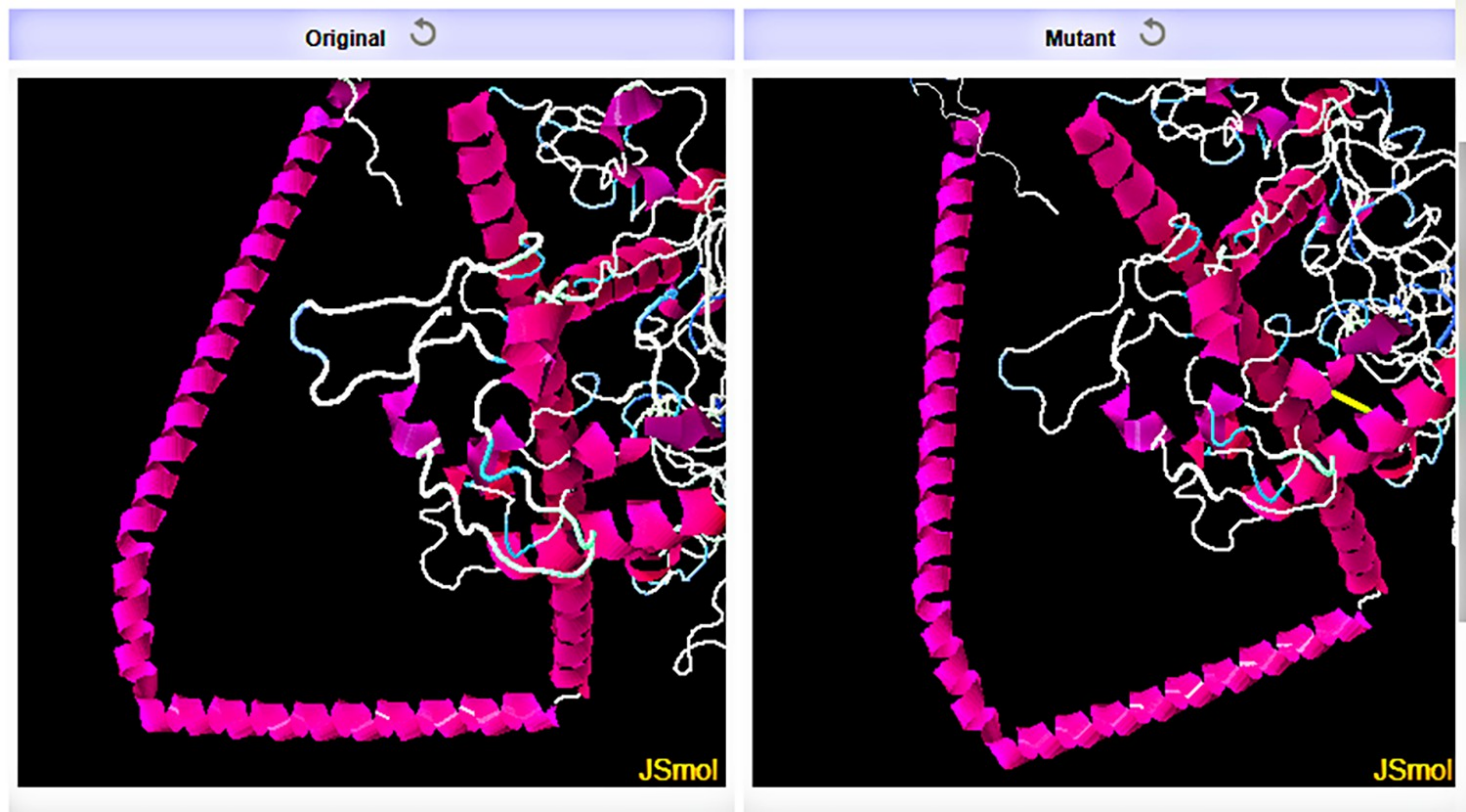
Disulphide engineering of the chimeric vaccine construct, V3 performed by the DbD2 v2.13 server. The right picture shows the original structure of the chimeric vaccine construct while the left picture shows the mutant chimeric vaccine construct with one disulphide bond (Yellow cylinders) created between single pair of amino acids.

The tertiary structure of the chimeric vaccine, V3 was produced by RaptorX and consists of three domains- alpha-helix, beta sheet, and coil. The three-dimensional structure of the chimeric vaccine, pictured in (Fig 6A). The secondary structure of the chimeric vaccine V3 modeled by CFSSP can be viewed in (Fig 6B). The server, GALAXYWEB, was used to operate the GALAXYrefine tool for the refinement of the vaccine construct, V3. This was done to increase the accuracy of 3D structures. The refining was done three times to achieve a high Ramachandran plot value. The PROCHECK program of the SAVES server was used to perform Ramachandran plot analysis. Before refinement, it was observed that the favored regions consisted of 84.0% of amino acid residues, the additional allowed regions consisted of 12.9% of amino acid residues, the generously permitted regions made up of 2.0% of amino acids and lastly, the disallowed regions consisted of 1.1% amino acids. Ramachandran plot analysis before refinement is illustrated by S2 Fig. However, after three refinements, the percentage of amino acids in favored regions increased to 90.6%, and the percentage of amino acids in additional allowed regions, generously allowed regions, and disallowed regions declined to 7.3%, 0.9% and 1.1%, respectively. The Ramachandran plot analysis after refinement is shown in (Fig 7). The ERRAT program of the SAVES v6.1 server predicted V3’s quality factor to be 85.230 in S3 Fig. After refinement, the quality factor of the chimeric vaccine construct, V3 remained the same in S4 Fig. These values confirm that the three-dimensional structure of the chimeric vaccine construct is accurate and is in excellent shape to perform the molecular docking process for further analysis. Moreover, the server DbD2 v2.13 was also utilized to predict disulfide engineering for the vaccine construct, V3. The server, DbD2 v2.13 displayed a list of 62 pairs of amino acids capable of producing disulfide bonds for the chimeric vaccine, V3. However, only the chosen amino acids were required to form disulphide bonds. These were the amino acids which had chi3 value between -87 to +97, B-factor to be 0.0 and energy value lower than 2.2. It was found that only one pair of amino acids matched the criteria. This pair of amino acids (LYS 405-ALA 425) was mutated to cysteine. The three-dimensional structure of chimeric vaccine prior to and after disulphide engineering can be viewed in (Fig 8).

### 3.12 Conformational B-cell epitopes were predicted by using IEDB

**Fig 9:**
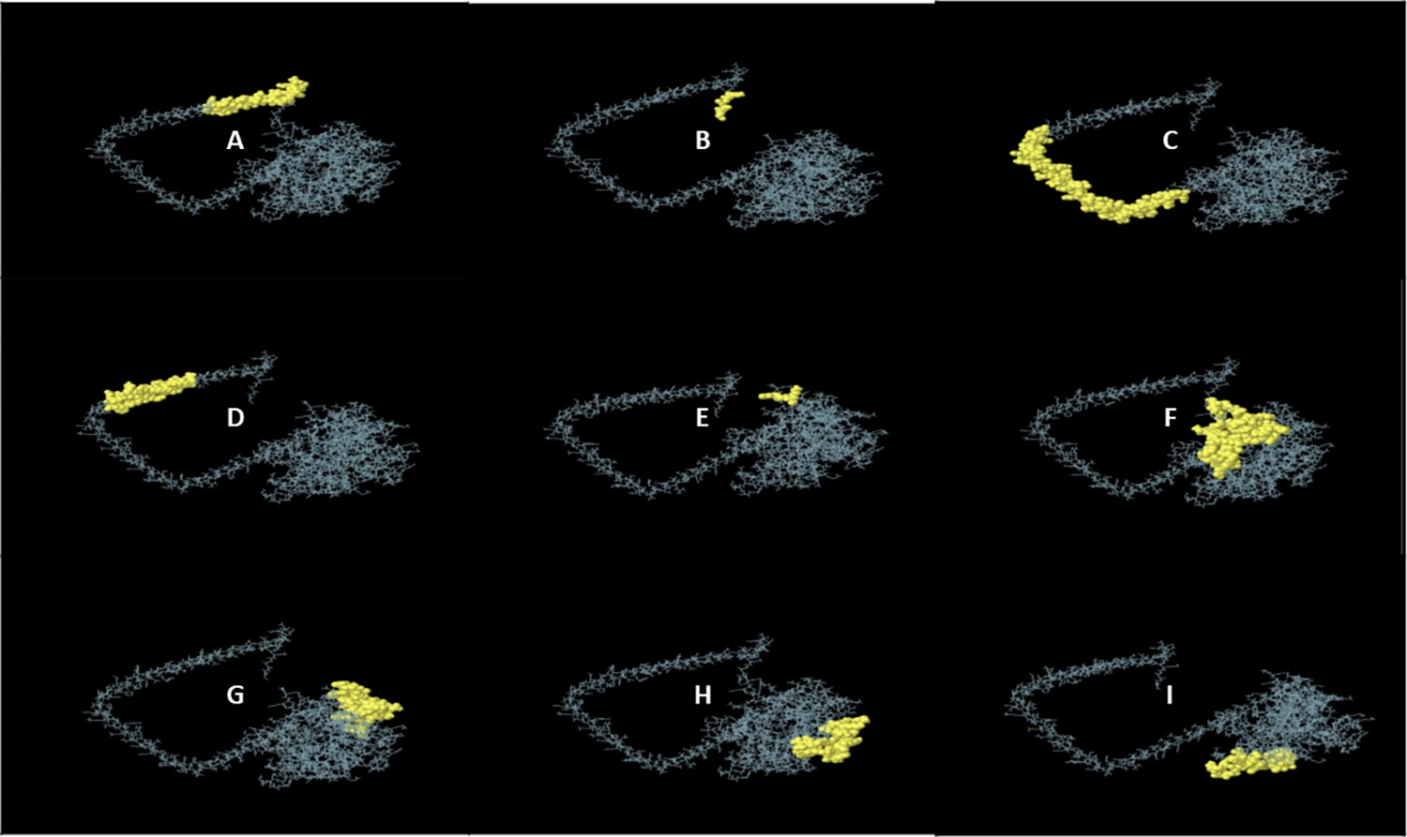
The discontinuous b-cell epitopes in the three-dimensional structure of the chimeric vaccine, V3 model. The gray stick models represent the 3D models of the chimeric vaccine, and the yellow spheres represent the discontinuous b-cell epitopes **(A)** 38 residues with a score of 0.948; **(B)** 5 residues with a score of 0.874; **(C)** 66 residues with a score of 0.838; **(D)** 30 residues with a score of 0.837; **(E)** 3 residues with a score of 0.78; **(F)** 55 residues with a score of 0.674; **(G)** 53 residues with a score of 0.552; **(H)** 35 residues with a score of 0.542; **(I)** 35 residues with a score of 0.53.

A total of nine conformational b-cell epitopes were predicted in the vaccine construct which possess higher scores than 0.5. The conformational b-cell epitopes with their scores can be observed in Table 13. All the locations of conformational b-cell epitopes in the three-dimensional structure of the chimeric vaccine can be viewed in (Fig 9).

**Table 13:** List of discontinuous epitopes and their predicted scores.

No.	Residues	No. of residues	Score
1	A:M6, A:A7, A:E8, A:N9, A:P10, A:N11, A:I12, A:D13, A:D14, A:L15, A:P16, A:A17, A:P18, A:L19, A:L20, A:A21, A:A22, A:L23, A:G24, A:A25, A:A26, A:D27, A:L28, A:A29, A:L30, A:A31, A:T32, A:V33, A:N34, A:D35, A:L36, A:I37, A:A38, A:N39, A:L40, A:R41, A:E42, A:R43	38	0.948
2	A:E1, A:A2, A:A3, A:A4, A:K5	5	0.874
3	A:I74, A:E75, A:L76, A:R77, A:D78, A:K79, A:F80, A:T81, A:T82, A:E83, A:E84, A:L85, A:R86, A:K87, A:A88, A:A89, A:E90, A:G91, A:Y92, A:L93, A:E94, A:A95, A:A96, A:T97, A:N98, A:R99, A:Y100, A:N101, A:E102, A:L103, A:V104, A:E105, A:R106, A:G107, A:E108, A:A109, A:A110, A:L111, A:Q112, A:R113, A:L114, A:R115, A:S116, A:Q117, A:T118, A:A119, A:F120, A:E121, A:D122, A:A123, A:S124, A:A125, A:R126, A:A127, A:E128, A:G129, A:Y130, A:V131, A:D132, A:Q133, A:A134, A:V135, A:E136, A:L137, A:Q139, A:E140	66	0.838
4	A:A44, A:E45, A:E46, A:T47, A:R48, A:A49, A:E50, A:T51, A:R52, A:T53, A:R54, A:V55, A:E56, A:E57, A:R58, A:R59, A:A60, A:R61, A:L62, A:T63, A:K64, A:F65, A:Q66, A:E67, A:D68, A:L69, A:P70, A:E71, A:Q72, A:F73	30	0.837
5	A:E582, A:G583, A:K584	3	0.78
6	A:P529, A:V530, A:Q531, A:P532, A:T533, A:E534, A:A535, A:A536, A:A537, A:K538, A:G539, A:S540, A:G541, A:T542, A:T543, A:E544, A:T545, A:S546, A:E547, A:E548, A:P549, A:Q550, A:K551, A:R552, A:G553, A:T554, A:K555, A:N557, A:S558, A:A559, A:G560, A:T561, A:D562, A:E563, A:L564, A:G565, A:R566, A:N567, A:G568, A:S569, A:A570, A:K571, A:K572, A:P573, A:M574, A:I575, A:V576, A:L577, A:G578, A:G579, A:K580, A:K581, A:Y585, A:S586, A:P587	55	0.674
7	A:A180, A:A181, A:A182, A:G183, A:G184, A:G185, A:S186, A:Y187, A:L188, A:S189, A:G190, A:Q191, A:S192, A:N193, A:E194, A:E195, A:G196, A:G197, A:G198, A:S199, A:V200, A:L202, A:S203, A:S204, A:F205, A:F206, A:A207, A:K213, A:G214, A:P215, A:G216, A:G217, A:N218, A:G219, A:E220, A:G221, A:G222, A:G223, A:G224, A:G225, A:S226, A:T227, A:K228, A:A229, A:S230, A:S231, A:S232, A:G233, A:D234, A:S253, A:A254, A:S255, A:G256	53	0.552
8	A:G469, A:A470, A:D471, A:K472, A:K473, A:Q474, A:G475, A:G476, A:V477, A:I478, A:G486, A:V487, A:K488, A:K489, A:S490, A:S491, A:G492, A:S493, A:H494, A:A495, A:Q496, A:Q497, A:S498, A:K499, A:K500, A:V501, A:G505, A:V506, A:V514, A:G515, A:V516, A:P517, A:G518, A:V519, A:K520	35	0.542
9	A:E303, A:A305, A:A306, A:G314, A:P315, A:G327, A:P328, A:T329, A:K330, A:A331, A:S332, A:S333, A:S334, A:G335, A:D336, A:G337, A:A338, A:A339, A:P340, A:C341, A:H342, A:E343, A:A344, A:A345, A:A346, A:K347, A:G348, A:P349, A:S350, A:E351, A:D352, A:G353, A:G354, A:T358, A:D359	35	0.53

### 3.13 Molecular docking of the chimeric vaccine construct with toll-like receptors

**Fig 10:**
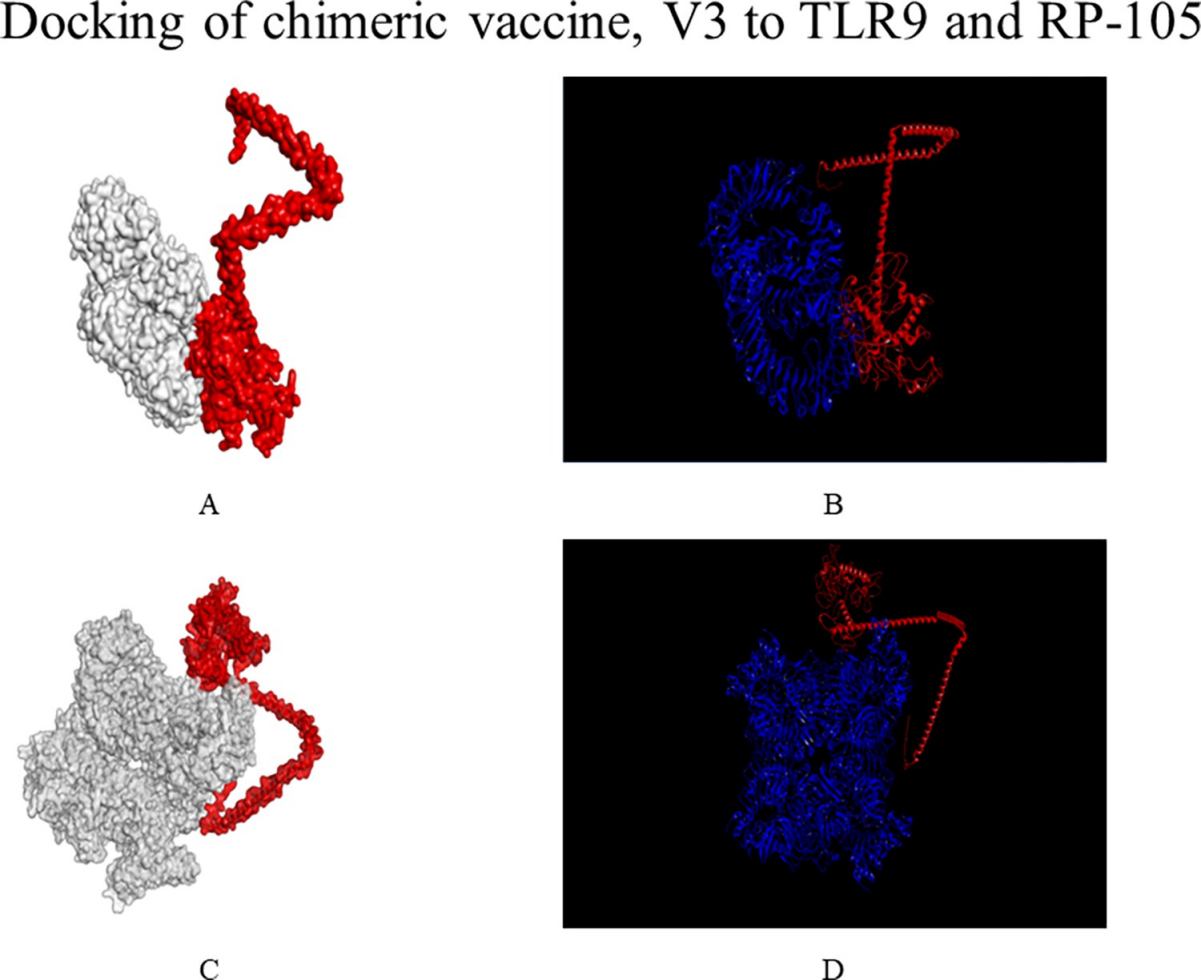
Molecular docking of the chimeric vaccine construct, V3 with Toll-like receptor TLR9 and with RP-105 (30% resemblance in sequence to TLR4) predicted by HDOC server. (A) The red colored model represents the chimeric vaccine construct, V3 while the white colored model represents the Toll-like receptor 9 (sphere model). (B) The red colored model represents the vaccine construct, V3 while the blue colored model represents Toll-like receptor 9 (ribbon model). (C) The red colored model represents the vaccine construct, V3 while the white colored model represents RP-105 (sphere model). (D) The red colored model represents the vaccine construct, V3 while the blue colored model represents RP-105 (ribbon model).

**Fig 11:**
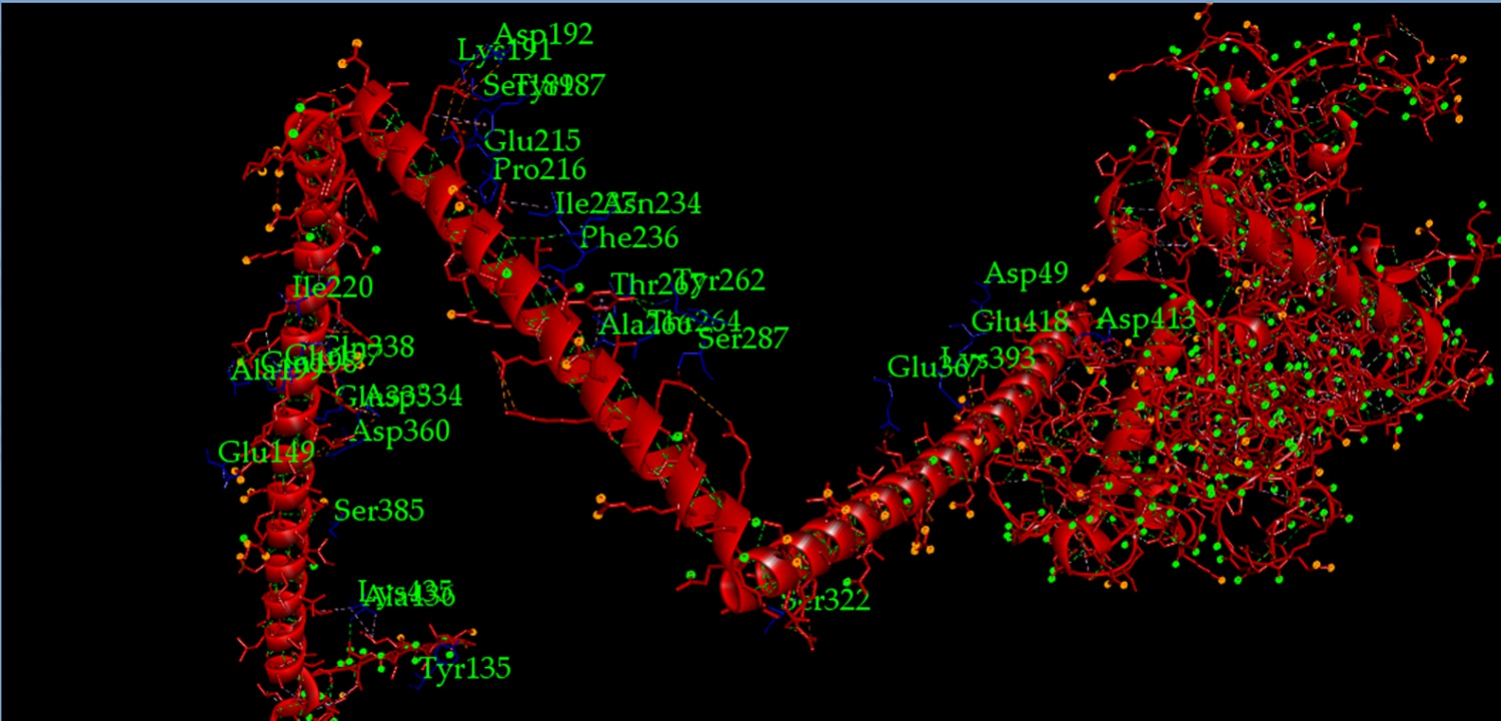
Amino acid residues of RP-105 involved in docking with the chimeric vaccine construct, V3. The red colored ribbon model represents the chimeric vaccine, V3 and the blue colored stick model represents the RP-105 receptor. The amino acids Ser322, Glu149, Glu197, Gln198, Ala199, Ile220, Tyr135, Asp49, Asp334, Gln335, Gln338, Asp360, Ser385, Lys435, Ala436, Tyr187, Ser189, Lys191, Asp192, Glu215, Pro216, Asn234, Phe236, Ile237, Tyr262, Thr264, Ala266, Thr267, Ser287, Glu367, Lys393, Asp413, and Glu418 of RP-105 have docked with chimeric vaccine, V3.

**Fig 12:**
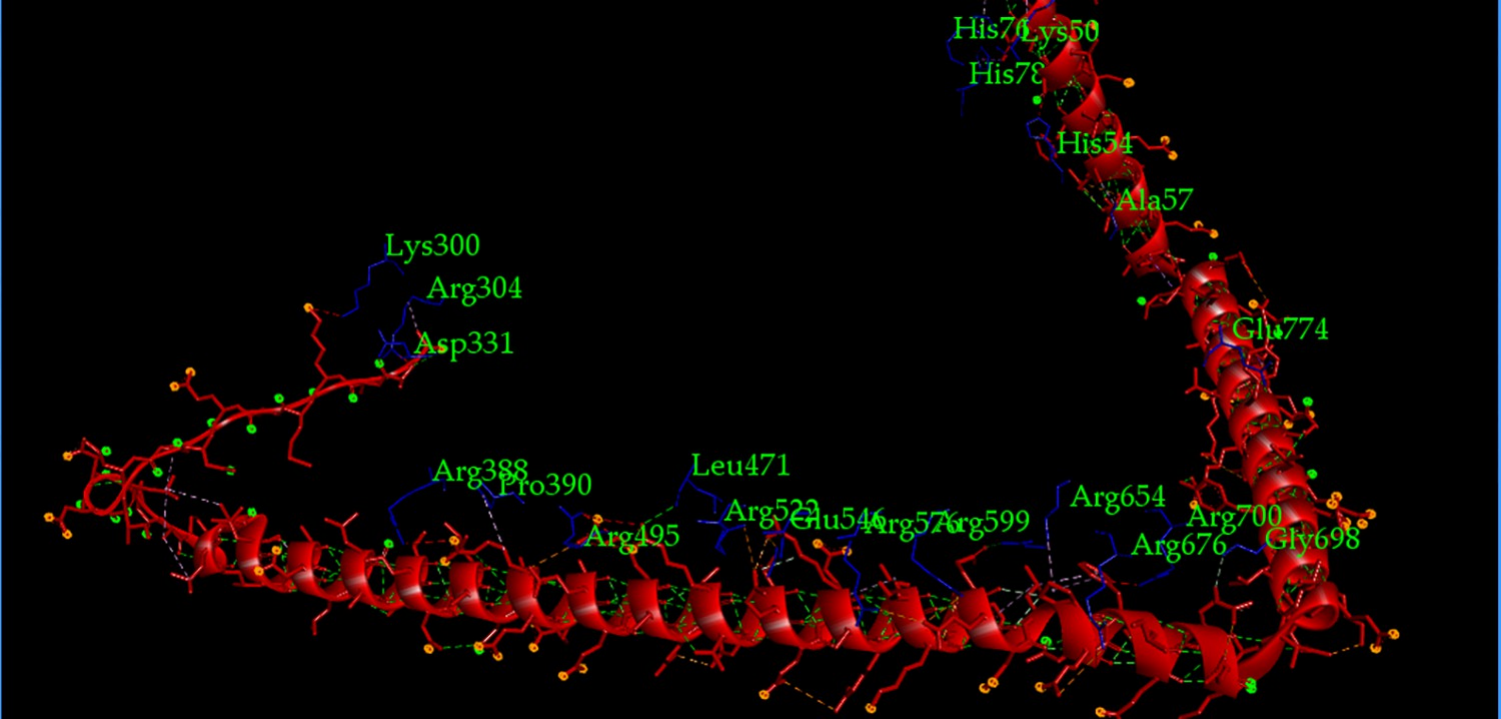
Amino acid residues of TLR9 involved in docking with the chimeric vaccine construct, V3. The red colored ribbon model represents the chimeric vaccine, V3 and the blue colored stick model represents the TLR-9 receptor. The amino acids Lys50, His54, Ala57, His76, His78, Lys300, Arg304, Asp331, Arg388, Pro390, Arg495, Leu471, Arg522, Glu546, Arg576, Arg599, Arg654, Arg676, Gly698, Arg700, and Glu774 of TLR-9 have docked with chimeric vaccine, V3.

PatchDock v1.3, HDOCK and ClusPro v2.0 servers were used to dock between the chimeric vaccine construct, V3 and toll-like receptors, i.e., bovine TLR-9 and RP-105 that resemble TLR4. All the results derived from PatchDock v1.3 server undergo refinement by using FireDock server to arrange the docking complex models according to their decreasing global binding energy. It was revealed that the chimeric vaccine construct, V3 docked with TLR-9 with a global binding energy of -10.79. It docked with RP-105 with a global binding energy of -3.39. The results prove that the chimeric vaccine, V3 docked much stronger with TLR-9 than V3 when docked with RP105 in Table 14. The docking of chimeric vaccine, V3 to TLR-9 and RP-105 can be seen in (Fig 10), respectively. The results derived from HDOCK server revealed that the global binding energy of the docking between chimeric vaccine, V3 and TLR-9 was -325.84 while the docking of chimeric vaccine, V3 and RP-105 (resembling TLR4) was -380.14. To identify the amino acids belonging to the Toll-like receptors which were involved in docking with the chimeric vaccine, V3, the software Discovery Studio Visualizer was used. **Thirty-three amino acids** of the RP-105 receptor had docked with the chimeric vaccine, V3. These involved Ser322, Glu149, Glu197, Gln198, Ala199, Ile220, Tyr135, Asp49, Asp334, Gln335, Gln338, Asp360, Ser385, Lys435, Ala436, Tyr187, Ser189, Lys191, Asp192, Glu215, Pro216, Asn234, Phe236, Ile237, Tyr262, Thr264, Ala266, Thr267, Ser287, Glu367, Lys393, Asp413, and Glu418. The interaction of amino acids between the chimeric vaccine, V3 and Rp-105 can be viewed in (Fig 11). **Twenty-one amino acids** belonging to Toll-like receptor 9 had docked with the chimeric vaccine, V3. These included Lys50, His54, Ala57, His76, His78, Lys300, Arg304, Asp331, Arg388, Pro390, Arg495, Leu471, Arg522, Glu546, Arg576, Arg599, Arg654, Arg676, Gly698, Arg700, and Glu774. The interaction of amino acids between chimeric vaccine, V3 and TLR9 can be observed in (Fig 12). Hence, the results from the HDOCK server show that the chimeric vaccine, V3 binds higher affinity to RP-105 than to TLR-9.

**Table 14:** Global binding Energy, Hydrogen Bond Energy, Atomic contact energy (ACE) and docking scores of the docked complexes V3-TLR9 and V3-TLR4.

Vaccine Construct	Toll-like receptor PDB ID’s	Global Energy	Docked Complex	Hydrogen Bond Energy	ACE	Score	Area
**V3**	5Y3M	-10.79	V3-TLR9	-3.48	8.99	12450	1780.10
	3RG1	-3.39	V3-TLR4	0.00	-0.24	15598	2101.40

### 3.14 Peptide docking with toll-like receptors

**Fig 13:**
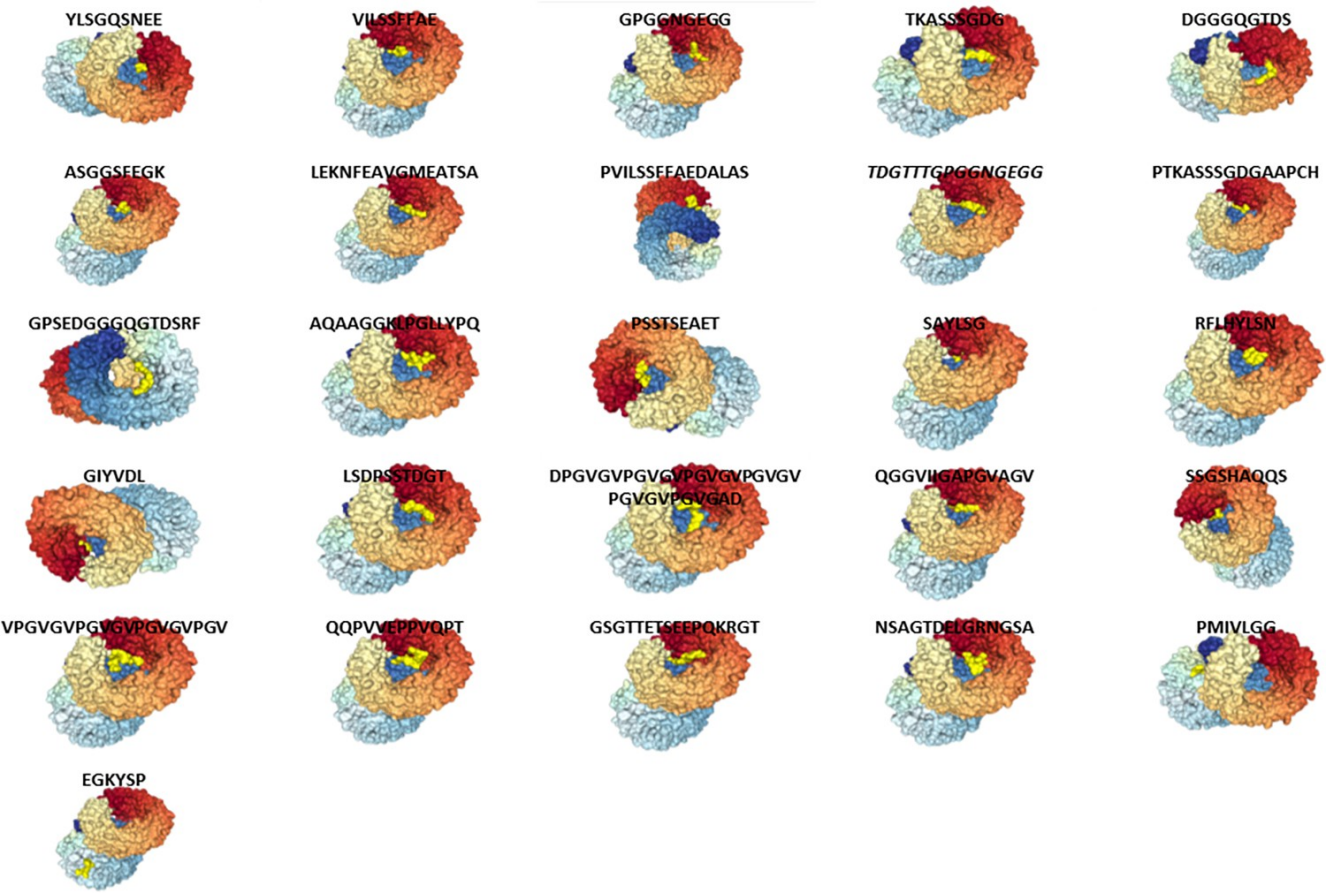
The molecular docking of the peptides with the toll-like receptor TLR-9. The peptides are represented in yellow colored surface model which is bonded to the rainbow-colored 3D surface models of TLR9.

Each of the peptides that were used to construct the chimeric vaccine was used to conduct molecular docking interactions with both the toll-like receptors, TLR9 and RP-105 by using the server, HPEPDOCK. We observe from the docking results that all the peptides have bonded with the toll-like receptors with high binding affinities. The docking energy scores of all the interactions of the peptides with TLR9 and Rp-105 can be viewed in Table 15. We can observe from the results that the b-cell epitopes of all the three rickettsial parasites have docked with both of the toll-like receptors with higher binding affinities compared to their counterparts MHC I and MHC II epitopes. We have also analyzed that the b-cell epitopes have strongly bonded to Rp-105 than to the receptor TLR9. Furthermore, the MHC I epitopes have bonded strongly with high binding affinities to Rp-105 whereas the MHC II epitopes have docked with high binding affinities to TLR9. This proves that all our MHC I, MHC II and b-cell epitopes present in the chimeric vaccine construct will bind efficiently to the bovine t-cell receptors and will efficiently stimulate both innate and adaptive immune responses. The docking of all the peptides with the three-dimensional model of both the receptors- TLR9 and Rp-105 can be observed in (Fig 13) and (S5 Fig).

**Table 15:** Assessment of the docking energy scores obtained from the molecular docking of MHC I, MHC II and b-cell epitopes with TLR9 and RP-105.

Type of epitopes	Position of epitopes	Peptides	Docking score with TLR9	Docking score with Rp-105 (resemblance to TLR4)
MHC I epitopes	**MSA-2c** 1–9	YLSGQSNEE	-194.956	-206.684
	**AMA-1** 3–11	VILSSFFAE	-219.925	-255.737
	**SPAG-1** 7–15	GPGGNGEGG	-147.623	-152.421
	**TASP** 2–10	TKASSSGDG	-164.748	-151.921
	**Vir-B10** 5–13	DGGGQGTDS	-162.661	-127.169
	**OMP-1** 12–20	ASGGSFEGK	-173.992	-218.568
MHC II epitopes	**MSA-2c** 1–15	LEKNFEAVGMEATSA	-195.623	-185.266
	**AMA-1** 2–16	PVILSSFFAEDALAS	-251.075	-301.903
	**SPAG-1** 1–15	TDGTTTGPGGNGEGG	-210.807	-167.410
	**TASP** 1–15	PTKASSSGDGAAPCH	-218.008	-236.129
	**Vir B-10** 1–15	GPSEDGGGQGTDSRF	-211.382	-185.108
	**OMP-1** 1–15	AQAAGGKLPGLLYPQ	-222.075	-227.901
B-cell epitopes	**MSA-2c** 34–42	PSSTSEAET	-166.620	-195.260
	**MSA-2c** 14–19	SAYLSG	-157.176	-203.865
	**AMA-1** 118–125	RFLHYLSN	-241.249	-255.155
	**AMA-1** 21–26	GIYVDL	-168.729	-204.633
	**SPAG-1** 415–424	LSDPSSTDGT	-164.233	-188.043
	**SPAG-1** 106–137	DPGVGVPGVGVPGVGVPGVGVPGVGVPGVGAD	-254.001	-220.964
	**SPAG-1** 200–213	QGGVIIGAPGVAGV	-209.151	-219.398
	**SPAG-1** 267–275	SSGSHAQQS	-190.122	-203.846
	**SPAG-1** 290–308	VPGVGVPGVGVPGVGVPGV	-234.885	-260.896
	**TASP** 62–73	QQPVVEPPVQPT	-210.180	-200.175
	**Vir B-10** 17–32	GSGTTETSEEPQKRGT	-192.834	-189.220
	**Vir B-10** 83–96	NSAGTDELGRNGSA	-185.400	-167.471
	**Vir B-10** 33–39	PMIVLGG	-179.337	-253.005
	**OMP-1** 61–66	EGKYSP	-152.629	-183.092

### 3.15 Molecular dynamic simulation of the vaccine-receptor complex

**Fig 14:**
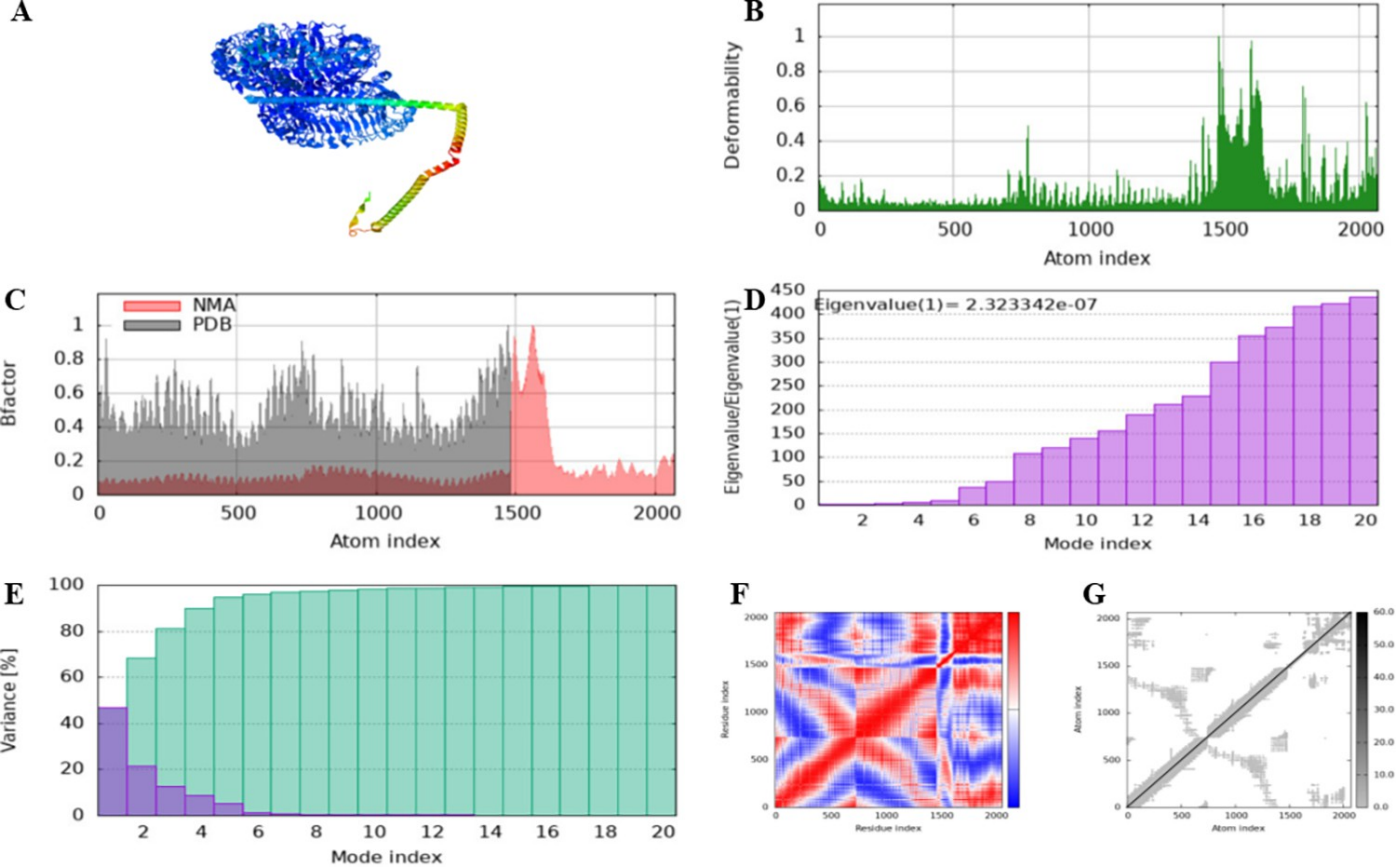
Molecular dynamic simulations of the chimeric vaccine construct, V3 with TLR-9 by the iMODS server. (A) The docking of chimeric V3 to TLR9 (B) Main chain deformability (C) B-factor values (D) The eigenvalue (E) Variance (F) Co-variance (G) Elastic network of the model.

According to the iMODS server a normal mode analysis of the vaccine-receptor complex, V3-TLR9 demonstrated large scale of mobility as well as protein stability. High deformability regions are observed as hinges between 500–1000 and 1500–2000 in the main chain. This means that the amino acid residues of the vaccine and TLR9 receptor in these areas were highly interacting with each other and flexible. The reduced B-factor value after normal mode analysis compared to the PDB B-factor value reduced the deformability. The B-factor values predicted from normal mode analysis were observed to be analogous to RMS from atom 1500 to 2000. Higher eigenvalues determine the protein-receptor complex stability. The eigenvalue was deduced to be 2.323342e-05 which explains that the V3-TLR9 complex motion is not stiff and the vaccine-receptor complex is stable. The variance was inversely proportional to the eigenvalue. The covariance matrix displayed highly correlated areas (red) in heat maps which demonstrated increased interaction between each residue. The elastic network revealed pairs of atoms connected through light gray dots which represent springs, demonstrating that they are not stiff. In (Fig 14) we illustrate the main-chain deformability of the chimeric vaccine V3-TLR9 complex.

### 3.16 Immunological analysis of the vaccine construct through immune simulation

**Fig 15:**
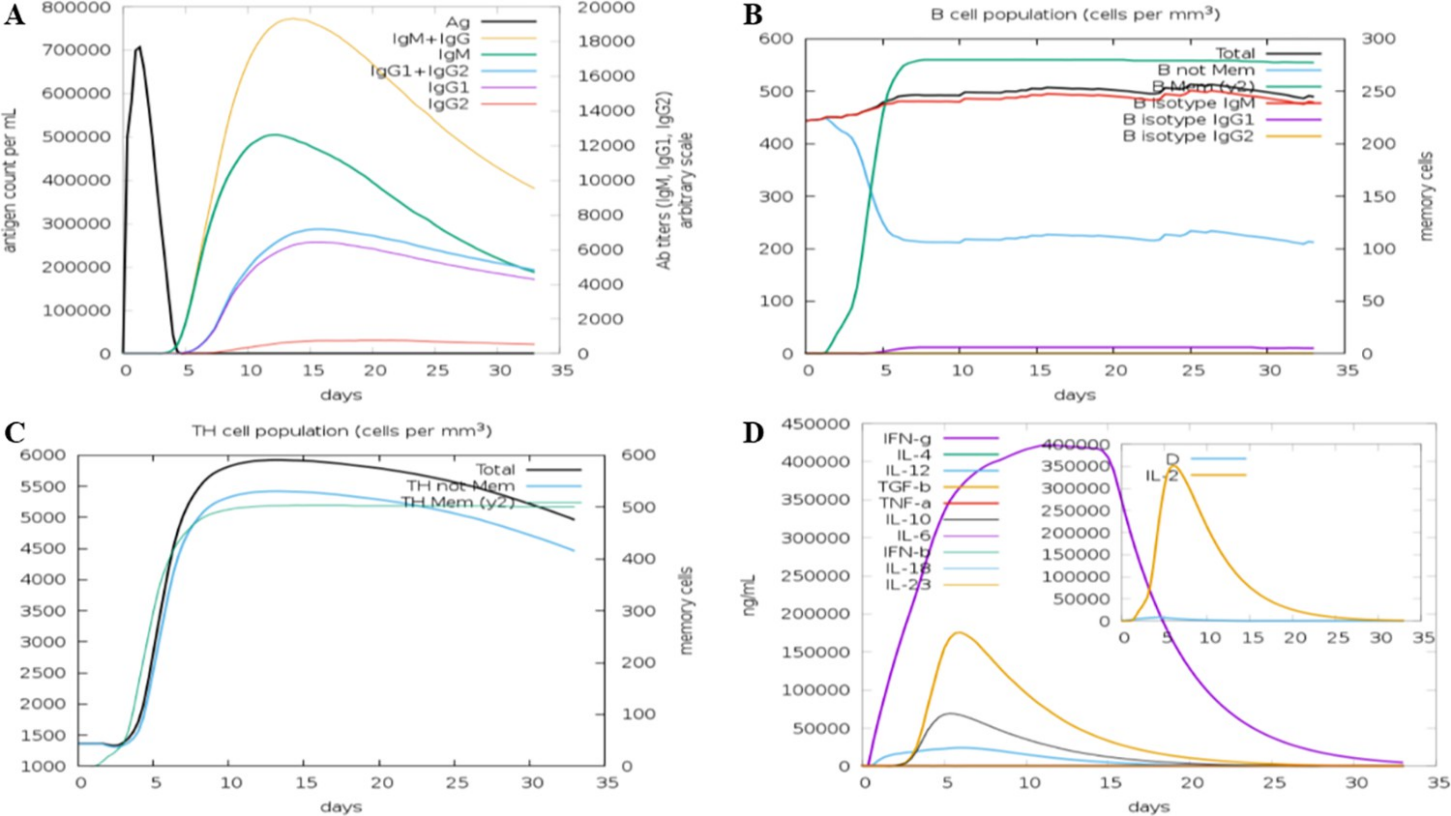
The immune simulation of chimeric vaccine, V3 by C-ImmSimm server. (A) Production of immunoglobulins in response to the chimeric vaccine, V3 (B) Population of B-cells in response to V3, γ2 represents the scale of memory B-cells (C) Population of Helper T-cells in response to V3, γ2 represents the scale of memory T-cells (D) Concentration of cytokines and interleukins produced.

The results of the immune simulation derived from the C-ImmSimm server [95] demonstrated that the chimeric vaccine construct, V3, had produced an effective immunological response. We even administered the vaccine three times to initiate a protective response. The results of the immune simulation provided by the C-ImmSimm server are illustrated in (Fig 15). It was observed that after 5 days of introducing the vaccine into the body’s circulation, the quantity of the antigen in the immune system decreased drastically. However, at the same time the production of both immunoglobulins IgM+IgG has increased greatly than the rest of the immunoglobulins. A significant rise in IgM, IgG1+IgG2, and IgG1 production is also observed. There is also a slight expression of immunoglobulin IgG2 in the immune system. Additionally, both cytotoxic and helper t-lymphocytes demonstrated an upregulated expression in the body’s immune system, confirming an effective and adequate response. The increased production of cytotoxic T-lymphocyte memory cells from the first day remained constant for many days. The helper T-lymphocyte memory cells largely increased from the 5th day and remained constant for many days. This offers assurance that the chimeric vaccine has the ability to create immunological memories. The presence of B-lymphocyte isotypes and their isotype switching has also been observed for an extended period of time along with an increase in B-lymphocyte memory cells. Insignificant increases were observed in dendritic cells that present antigens on both MHC I and MHC II molecules. Macrophage activity has increased greatly. Cytokines such as IFN-G display a greater increase in production than other cytokines such as TGF-b, IL-10 and IL-12.

### 3.17 Codon adaptations, in silico cloning and similarity analysis of the chimeric vaccine construct with cattle proteins

**Fig 16:**
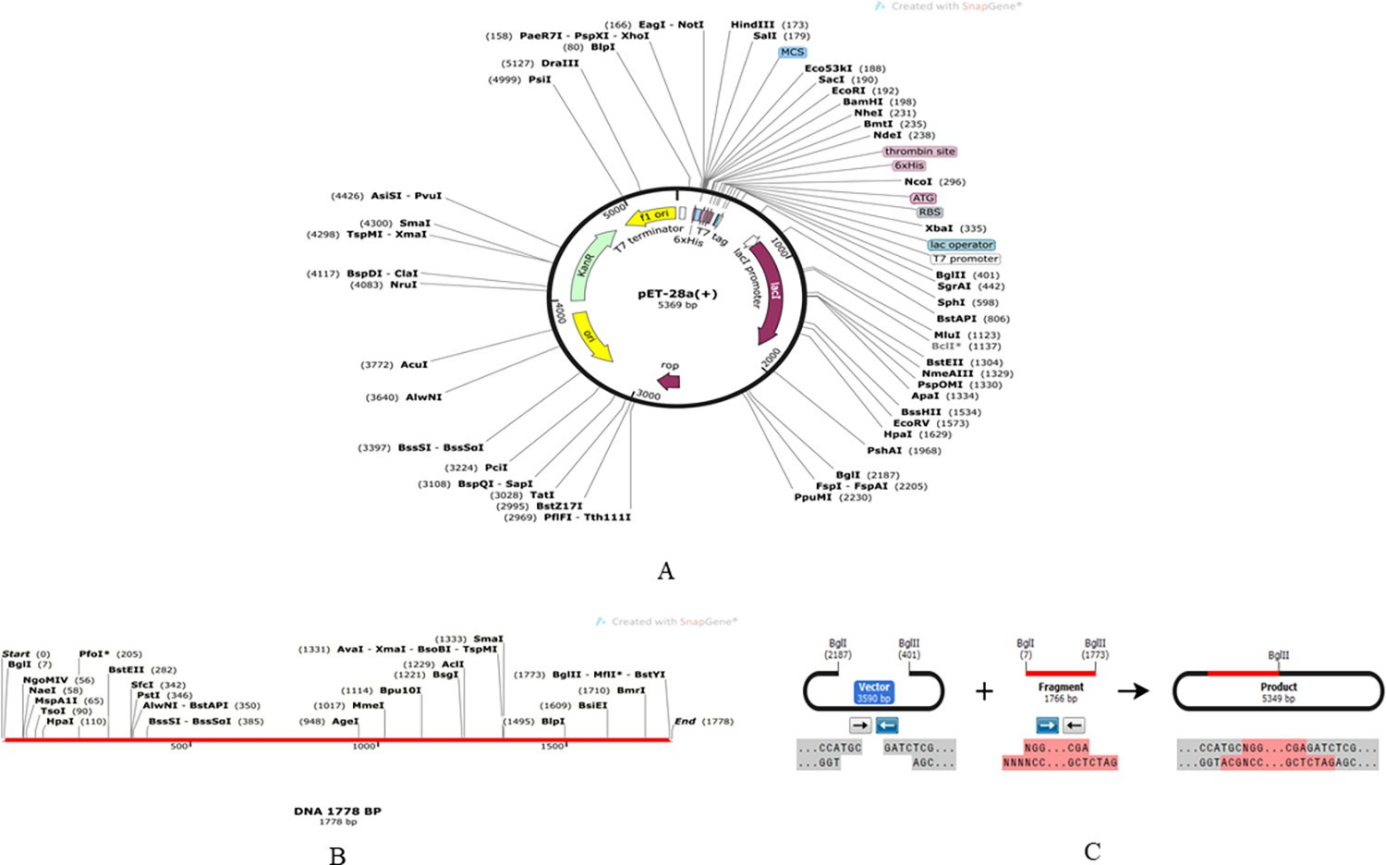
The insertion of the DNA sequence of the chimeric vaccine, V3 in to the plasmid vector, pET-28 (+). (A) The map of plasmid pET-28 (+). We can see that the vector, pET-28(+) represented here is intact and was 5369 bp before insertion of the chimeric vaccine, V3. (B) The adapted DNA sequence of the chimeric vaccine V3, to which the restriction enzymes BglI and BglII are added at each end. The adapted DNA sequence is 1778 bp long. We can observe all kinds of restriction sites present in the adapted DNA sequence. (C) Insertion of chimeric vaccine V3 DNA into the vector, pET-28 (+). The vector, pET-28(+) is 3590 bp in length and the cloned product (DNA) is 5349 bp in length.

Codon adaptation is usually performed to convert the amino acid sequence of the chimeric vaccine into DNA sequence. This is so that the DNA sequence could easily enter the host and become adapted to the environment from which the vector plasmid is extracted. This codon-adapted DNA sequence will be inserted into the plasmid for cloning and expression. The illustration of the vector plasmid, pET28a (+) is shown (Fig 16A). Reverse transcription is performed on the chimeric vaccine, V3, and it is converted to DNA sequences and adapted to E. coli using the codon adaptation tool, JCAT. The Codon adaptation index (CAI) value represents the number of adapted codons that was shifted to a maximum value of 1. The improved adapted DNA sequence was investigated to have a CAI value of 0.99 and the GC content to be 53.37. The chimeric vaccine did not consist of some restriction sites for BglI and BglII. Therefore, restriction sites were added to both ends of the chimeric vaccine V3 adapted sequence (Fig 16B). Insertion of the adapted chimeric vaccine DNA sequence into the pET-28a (+) could be seen in (Fig 16C). This insertion of the vaccine into the plasmid was carried out through SnapGene v6.2.0. As a result, a clone was produced consisting of 5356 bp, of which 1778 bp belonged to the adapted chimeric vaccine, V3. Cloned DNA is observed in S6 Fig. BLASTp was again run to check for similarity between the chimeric vaccine V3 and cattle proteins. The results revealed no similarities between them. It was found out that our chimeric vaccine, V3, was not similar to cattle proteins and hence, cattle will never develop immune tolerance towards the vaccine.

## 4. Discussion

Babesiosis, Theileriosis and bovine anaplasmosis are the three infectious diseases caused by *Babesia bovis*, *Theileria annulata* and *Anaplasma marginale* respectively. Many simultaneous multiple infections caused by all these three rickettsial parasites have been reported in the past as well as in the present. It is highly necessary to create vaccines that would fight against multiple tick-borne infections and assist the cattle to produce an immune response against all the parasites. Currently, *Babesia bovis* infections are controlled through the timely vaccinations of cattle and consumption of babesicidal drugs. Many countries such as Brazil, South Africa, Argentina, Australia and Uruguay are using live attenuated vaccinations to tackle Babesiosis, and Anaplasmosis [96]. However, these vaccines have many drawbacks as the attenuated vaccine can revert back to its virulent form, and have a shelf life of only four days when stored between 2–8°C [97,98]. Babesiosis is also controlled by using the drug, Imidocarb (3mg/kg), Tetracycline and, diminazene aceturate which remains in the edible tissues of cattle after long periods of administration [99]. The treatment of cattle from infectious diseases with antibiotics have led to concerns on the release of antibiotic drugs into animal products such as milk and meat which has become threatening for human consumption. This also has led to the emergence of antibiotic resistant pathogens [100].

Bovine Anaplasmosis treatment involves administration of drugs such as tetracycline compounds (5/10 mg; two times a day) during the early symptoms of the disease before parasitemia is elevated. In addition, imidocarb is also used to eliminate tick infestations [101]. Other than these treatments, bovine anaplasmosis is also treated by providing two types of vaccines: live and killed vaccines. These vaccines are synthesized by extracting *Anaplasma marginale* from bovine erythrocytes. These vaccines can reduce clinical illnesses, but do not prevent persistent infections caused by *A*. *marginale* [102]. However, these vaccines have high production costs, low immunogenic responses and require multiple booster doses. In the USA, expensive chemotherapy is provided to cattle to get rid of infected erythrocytes [99]. Theileriosis is treated by intramuscular drugs buparvaquone, parvaquone and oral drugs coccidiostat and halofuginone. However, these drugs never eradicate *T*. *annulata* infections and make cattle carriers. The disease is prevented by four measures: chemotherapy, cattle movement control, immunization and tick control. The immunization therapy for theileriosis includes two categories of vaccines: sporozoite vaccines and schizont vaccines which are derived from ticks and are not very effective [99]. These vaccines also possess many disadvantages which include a long period of time in their development, high genetic variability, evolution of dangerous microbes, inefficiency, and safety concerns [103].

Nowadays, strains of different microorganisms are evolving at a rapid rate. Hence, conventional making of strain-specific vaccines is inefficient to cope with the evolving strains. Similarly, it is becoming tougher to defeat rickettsial parasites. It has become a global problem with the aim of continuous resolvement for disease eradication followed by a recurrence of the same disease-causing microbes. The increasing resistance of antibiotics and drugs within hosts along with increasing mutations of infectious agents has led researchers to develop chimeric vaccines. These vaccines are easier to construct if we have the whole genome sequence of the disease-causing pathogen. Chimeric vaccines possess numerous advantages over conventional vaccines. This includes stronger immune response against multiple infectious microbes such as guaranteed humoral response produced by b-cells and cytotoxic immunity generated by t-cells, also adjuvants enhance their immune response, highly effective against microbes which have become highly resistant to multiple drugs, these vaccines are more stable, they have no risk of being infected by infectious microbes, their easy storage, transportation and rapid large-scale production. Lastly, the chimeric vaccine provides cross-neutralization in a single formulation [101]. With the aim to resolve all the concerns arising from conventional vaccines, we have constructed an effective and efficient in silico chimeric vaccine against the three parasites- *Babesia bovis*, *Theileria annulata*, and *Anaplasma marginale*. This research is completely original and has never been conducted before.

In this research, all the outer membrane proteins sequences belonging to the two apicomplexa- *Babesia bovis* and *Theileria annulata* along with rickettsia *Anaplasma marginale* were extracted from the site, UniProtKB (S35 File). The MHC I and MHC II epitopes predicted by the IEDB act as antigens that stimulate an immune response after activating both B-cells and T-cells. B cells are activated after the stimulation of helper T-cells that are produced in response to MHC II epitopes [104,105]. When the b-cells become activated, they produce specific antibodies against the antigens which mediate the effector functions after binding to the epitope or pathogen or toxin [106,107]. This is the principle which the vaccine mimics. Therefore, we constructed the chimeric vaccine with many antigenic epitopes. These epitopes could bind with high affinity with numerous BoLA MHC I and MHC II alleles [108]. Additionally, previous research involved the designing of separate in-silico vaccines against *Babesia bovis* and *Theileria annulata* [104]. However, none of the researchers have added MHC II epitopes (HTL epitopes) to their designed in silico vaccine due to the lack of bovine MHC II alleles in MHC prediction servers. Although, MHC II (HTL) epitopes are highly required in bovine vaccine designs as it not only activates the b-cells to secrete antibodies but also assists in the activation of macrophages and cytotoxic t-cells to kill infected cells [66]. In this research, we have studied about HLA alleles which possessed pseudo-sequence similarity to the BoLA alleles and then utilized those HLA alleles for the prediction of MHC II (HTL) epitopes. Earlier, we have designed a separate in silico multi-epitope vaccine against *A*. *marginale* consisting of all the MHC I, MHC II and b-cell epitopes using the same concept [109].

Later, we docked four newly modelled bovine MHC II alleles with the four epitopes TDGTTTGPGGNGEGG, PTKASSSGDGAAPCH, GPSEDGGGQGTDSRF, AQAAGGKLPGLLYPQ from SPAG-1 (*T*. *annulata*), TASP (*T*. *annulata*), Vir-B10 (*A*. *marginale*) and OMP-1 (*A*. *marginale*) respectively because our analysis showed that the lowest percentile values of the four predicted MHC II epitopes were very high above the threshold value, after the IEDB server predicted them. The reason for these results claiming a very high percentile value might be due to the predicted MHC II epitopes showing their binding affinity against human HLA MHC II alleles and not bovine MHC II alleles. The high affinity docking of the four bovine MHC II alleles with the four MHC II epitopes prove that the epitopes can easily bind with bovine MHC II alleles and initiate HTL response.

Furthermore, the chimeric vaccine V3 was allowed to dock with toll-like receptor 9 and RP-105 to predict the binding efficacy of the vaccine construct. Molecular docking is crucial to predicting whether our vaccine will adhere to receptors. We have used the bovine TLR-9 receptor for docking due to its high presence on bovine dendritic cells and macrophages. When the antigen binds to a bovine TLR9 receptor on dendritic cells, it stimulates IFN- α and IL-12. The IFN- α in turn stimulates the production of natural killer cells which kill cells infected with parasites and secrete IFN-γ. On the other hand, the TLR-9 receptors on the macrophages when stimulated initiate the production of pro-inflammatory cytokines, IL-6 and IL12 [110]. Bovine RP-105 activates B-cells. It also forms complexes with the LPS receptor TLR4/MD-2 complex and initiates LPS (from microbes) responses by B-cells [111]. It is also expressed on dendritic cells to negatively regulate TLR4-mediated LPS responses [61]. The docking results revealed that the chimeric vaccine V3 has made strong connections to both immune receptors with high binding affinities. Molecular dynamic simulations are also necessary to predict the stability of vaccine-receptor complexes as these complexes need to remain stable during their period in the bloodstream. The docked V3-TLR9 complex was then used to conduct molecular dynamic simulations. Since the toll-like receptor RP-105 was a very large receptor, we did not receive any results regarding molecular dynamic simulations for the docked V3-RP105 complex from the server. Results from the molecular dynamic simulations of V3-TLR9 complex revealed that very low energy is required for the docked complex to remain stable and execute powerful molecular interactions. The results of both molecular docking and dynamic simulations confirm that our newly designed chimeric vaccine, V3 can successfully bind to bovine immune receptors.

Moreover, our chimeric vaccine, V3 is effective in initiating an immune response which is proved by the immune simulation study. The major requirement of an effective vaccine is the production of both memory B-cells, T-cells and cytokines. After administering a vaccine with no lipopolysaccharides containing 1000 antigens, both memory b-cells and t-cells were generated. The immune simulation study also predicts the production of IFN-G which promotes both innate and adaptive immunity. IFN-G works as a macrophage-activating factor (MAF) that enables macrophages to synthesize proinflammatory cytokines, chemotaxis towards pathogens, and enhances their antigen presentation and phagocytosis. IFN-G influences the generation of antigenic peptides for antigen presentation and activation of the adaptive immune response. It likewise promotes the expression of both MHC class I and MHC class II molecules. IFN-G regulates class switching of antibodies and b-cell proliferation. IFN-G mediates IL-2 upregulation that generates cytotoxic T-cells and its cytolytic actions. [112]. Lastly, the chimeric vaccine, V3, was reverse transcribed to the DNA sequence adapted to the host, *E*. *coli* strain k12 so that it is easily inserted into the plasmid pET28a (+) and accepted by the host, *E*. *coli* for cloning and expression. This vector plasmid pET28a (+) was used because it contains a T7 promoter and a lac operator sequence to inhibit uninduced expression. The initiation of translation of proteins is controlled by the Shine-Dalgarno sequence it possesses. The coding sequence of the chimeric vaccine can be expressed downstream or in frame with the coding sequence for a poly-histidine purification tag (His6) and a thrombin protease recognition site (TPS) so that the recombinant protein produced can be easily purified using standardized protocols [113].

In this study, we have involved the usage of many online servers to identify potential vaccine candidate proteins that were nominated to be highly conserved among the majority of the strains of the related pathogen. Conservancy in proteins was maintained among *Babesia bovis*, *Theileria annulata* and *Anaplasma marginale*. All MHC I, MHC II and B-cell epitopes were predicted using the conserved regions of the proteins. Following the prediction of epitopes, the conservancy of all the epitopes was again rechecked using the conservancy tool of IEDB which proved to be 100%. In addition, all the epitopes had a high antigenic score, were non-allergenic, non-toxic and the vaccine was non-homologous to bovine proteins. This explains that our chimeric vaccine construct, V3 is highly cross reactive. It can induce immune responses against many strains of *Babesia bovis*, *Theileria annulata* and *Anaplasma marginale*. It is really remarkable that reverse vaccinology makes it possible to design chimeric vaccines without cultivating parasites. Through these innovative technologies we could create a better future without the need to create traditional, conventional vaccines which are very expensive and time-consuming.

## 5. Conclusion

In this research, we have brought an innovative idea of designing an essential, efficient and effective chimeric vaccine via in silico multi-epitope vaccine design that individually would have the potential to induce an immune response simultaneously against three bovine protozoan parasites; *Babesia bovis*, *Theileria annulata* and *Anaplasma marginale* as it contains highly immunogenic epitopes. The one and only limitation of this study is the lack of crystal structure of bovine TLR4 in the protein data bank. As the receptor is absent, we had to perform molecular docking and molecular dynamic simulation on Rp-105 (resembling TLR4) and the server, iMODS, did not provide any simulation results for the large structure size of Rp-105. It is our first attempt to develop a chimeric vaccine against three bovine protozoan parasites. No studies have yet been conducted on developing chimeric vaccine against the three bovine parasites. All the MHC I epitopes, MHC II epitopes and B-cell epitopes used in this study are highly immunogenic and antigenic. Linkers and adjuvants along with padre sequences were added to the chimeric vaccine design to increase its efficiency. It is necessary to conduct tests both in vitro and in vivo. This is to determine the capability of this chimeric vaccine to elicit an effective immune response against the three protozoan parasites. This attempt will help us save our cattle from infectious diseases and elevate the economy of many countries through increased meat and milk production.

## Supporting information

S1 Fig Secondary structures of the chimeric vaccine construct, V3 in cartoon model predicted by PSIPRED v4.0 server. This depicts navy-blue bars representing confidence of the prediction for different domains in the structure. Pink bars represent Helixes; Yellow bars represent beta-strands and Line represents coils in the structure.
(TIF)

S2 Fig Ramachandran plot analysis of the chimeric vaccine construct, V3 (before refinement) predicted by the PROCHECK program on the SAVE v6.1 server. Before refinement, the amino acid residues in the most favored regions are found to be 84.0%.
(TIF)

S3 Fig ERRAT results displaying the Quality factor value of the chimeric vaccine construct, V3 before refinement. The Quality factor value of the refined vaccine is 85.230.
(TIF)

S4 Fig ERRAT result displaying the Quality factor value of the chimeric vaccine construct, V3 after refinement. The Quality factor value of the refined vaccine remains 85.230.
(TIF)

S5 Fig The molecular docking of the peptides with the Rp-105 (resemblance to TLR4). The peptides are represented in yellow colored surface model which are bonded to the rainbow-colored 3D surface models of Rp-105.
(TIF)

S6 Fig Cloned DNA contains DNA of chimeric vaccine V3 fused to plasmid pET-28(+). We can see here that the final cloned DNA is 5356 bp in length. It consists of all genes and restriction sites in a common plasmid. The DNA sequence of the chimeric vaccine fused to pET-28(+) is shown red.
(TIF)

S1 File The table represents the BoLA class I supertypes and their related BoLA allele members which were selected for MHC I epitope prediction in IEDB server. (DOCX)

S2 File The tables of all the ten MHC I epitopes of AMA-1 with their scores and percentile rank representing their affinities for different BOLA alleles. (DOCX)

S3 File The tables of all the ten MHC I epitopes of MSA-2c with their scores and percentile rank representing their affinities for different BOLA alleles. (DOCX)

S4 File The tables of all the ten MHC I epitopes of SPAG-1 with their scores and percentile rank representing their affinities for different BOLA alleles. (DOCX)

S5 File The tables of all the ten MHC I epitopes of TASP with their scores and percentile rank representing their affinities for different BOLA alleles. (DOCX)

S6 File The tables of all the ten MHC I epitopes of Vir-B10 with their scores and percentile rank representing their affinities for different BOLA alleles. (DOCX)

S7 File The tables of all the ten MHC I epitopes of OMP-1 with their scores and percentile rank representing their affinities for different BOLA alleles. (DOCX)

S8 File The tables of all the ten MHC II epitopes of AMA-1 with their scores and percentile rank representing their affinities for different BOLA alleles. (DOCX)

S9 File The tables of all the ten MHC II epitopes of MSA-2c with their scores and percentile rank representing their affinities for different BOLA alleles. (DOCX)

S10 File The tables of all the ten MHC II epitopes of SPAG-1 with their scores and percentile rank representing their affinities for different BOLA alleles. (DOCX)

S11 File The tables of all the ten MHC II epitopes of TASP with their scores and percentile rank representing their affinities for different BOLA alleles. (DOCX)

S12 File The tables of all the ten MHC II epitopes of Vir B-10 with their scores and percentile rank representing their affinities for different BOLA alleles. (DOCX)

S13 File The tables of all the ten MHC II epitopes of OMP-1 with their scores and percentile rank representing their affinities for different BOLA alleles. (DOCX)

S14 File The table representing the BoLA alleles with their amino acid sequence, their length and the HLA-allele to which they are pseudo-sequence similar. (DOCX)

S15 File Conserved regions of each protein retrieved from Clustal Omega with their antigenic scores and transmembrane topology screening. (DOCX)

S16 File Antigenicity prediction, screening of transmembrane topology, allergenicity, conservancy along with toxicity assessment of the 10 best major histocompatibility complex class I epitopes of AMA-1. (DOCX)

S17 File Antigenicity prediction, screening of transmembrane topology, allergenicity, conservancy along with toxicity assessment of the 10 best major histocompatibility complex class I epitopes of MSA-2c. (DOCX)

S18 File Antigenicity prediction, screening of transmembrane topology, allergenicity, conservancy along with toxicity assessment of the 10 best major histocompatibility complex class I epitopes of SPAG-1. (DOCX)

S19 File Antigenicity prediction, screening of transmembrane topology, allergenicity, conservancy along with toxicity assessment of the 10 best major histocompatibility complex class I epitopes of TASP. (DOCX)

S20 File Antigenicity prediction, screening of transmembrane topology, allergenicity, conservancy along with toxicity assessment of the 10 best Major Histocompatibility complex class I epitopes of Vir-B10. (DOCX)

S21 File Antigenicity prediction, screening of transmembrane topology, allergenicity, conservancy along with toxicity assessment of the 10 best major histocompatibility complex class 1 epitopes of OMP-1. (DOCX)

S22 File Antigenicity prediction, screening of transmembrane topology, allergenicity, conservancy along with toxicity assessment of the 10 best major histocompatibility complex class II epitopes of AMA-1. (DOCX)

S23 File Antigenicity prediction, screening of transmembrane topology, allergenicity, conservancy along with toxicity assessment of the best major histocompatibility complex class IIepitope of MSA-2c. (Only one MHC II epitope was found for MSA-2c).
(DOCX)

S24 File Antigenicity prediction, screening of transmembrane topology, allergenicity, conservancy along with toxicity assessment of the 10 best major histocompatibility complex class II epitope of SPAG-1. (DOCX)

S25 File Antigenicity prediction, screening of transmembrane topology, allergenicity, conservancy along with toxicity assessment of the 10 best major histocompatibility complex class II epitope of TASP. (DOCX)

S26 File Antigenicity prediction, screening of transmembrane topology, allergenicity, conservancy along with toxicity assessment of the 10 best major histocompatibility complex class II epitope of OMP-1. (DOCX)

S27 File Antigenicity prediction, screening of transmembrane topology, allergenicity, conservancy along with toxicity assessment of the 10 best major histocompatibility complex class II epitope of Vir-B10. (DOCX)

S28 File The table representing all the predicted B-cell epitopes using three algorithm methods selected for chimeric vaccine construction with their position and residual scores. (DOCX)

S29 File B-cell epitope prediction of MSA-2c. (DOCX)

S30 File B-cell epitope prediction of AMA-1. (DOCX)

S31 File B-cell epitope prediction of SPAG-1. (DOCX)

S32 File B-cell epitope prediction of TASP. (DOCX)

S33 File B-cell epitope prediction of Vir-B10. (DOCX)

S34 File B-cell epitope prediction of OMP-1. (DOCX)

S35 File FASTA protein sequences of all the six proteins- MSA-2c AMA-1, SPAG-1, TASP, Vir B10 and OMP1 selected for chimeric vaccine construction. (DOCX)

## Abbreviations

MSA-2c: Merozoite surface antigen 2c
AMA-1: Apical membrane antigen
TASP: Theileria annulata surface protein
SPAG-1: Sporozoite antigen
OMP-1: Outer membrane protein-1
MHC I: Major Histocompatibility complex I
MHC II: Major Histocompatibility complex II
