# Supplementary figures and images for: Development of a multi-epitope chimeric vaccine in silico against Babesia bovis, Theileria annulata, and Anaplasma marginale using computational biology tools and reverse vaccinology approach
Source: PLoS One. 2025 Jan 24;20(1):e0312262. doi: 10.1371/journal.pone.0312262 (PMC11759392; doi:10.1371/journal.pone.0312262)

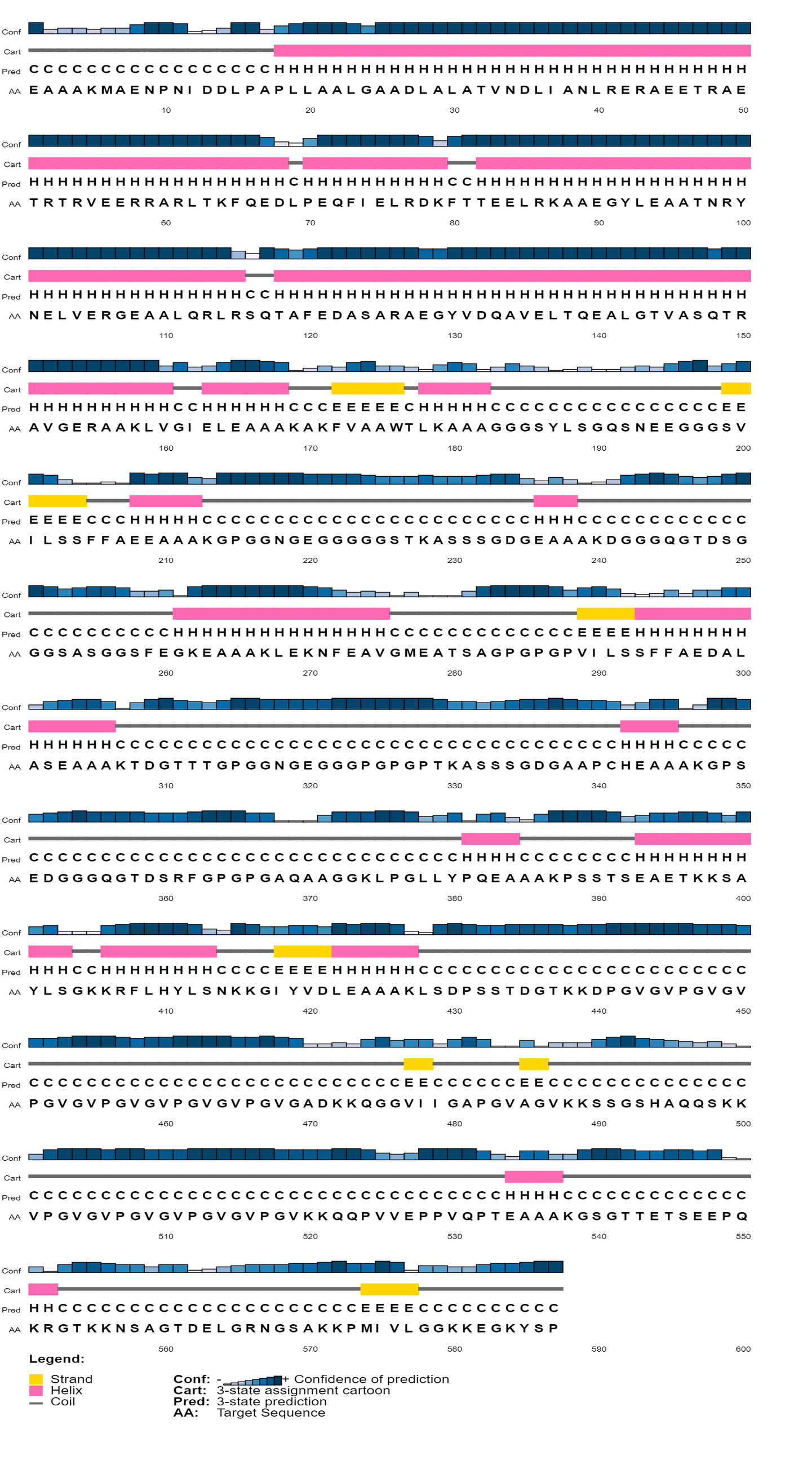

Supplement: S1 Fig — This depicts navy-blue bars representing confidence of the prediction for different domains in the structure. Pink bars represent Helixes; Yellow bars represent beta-strands and Line represents coils in the structure. (TIF) [file pone.0312262.s001.tif]

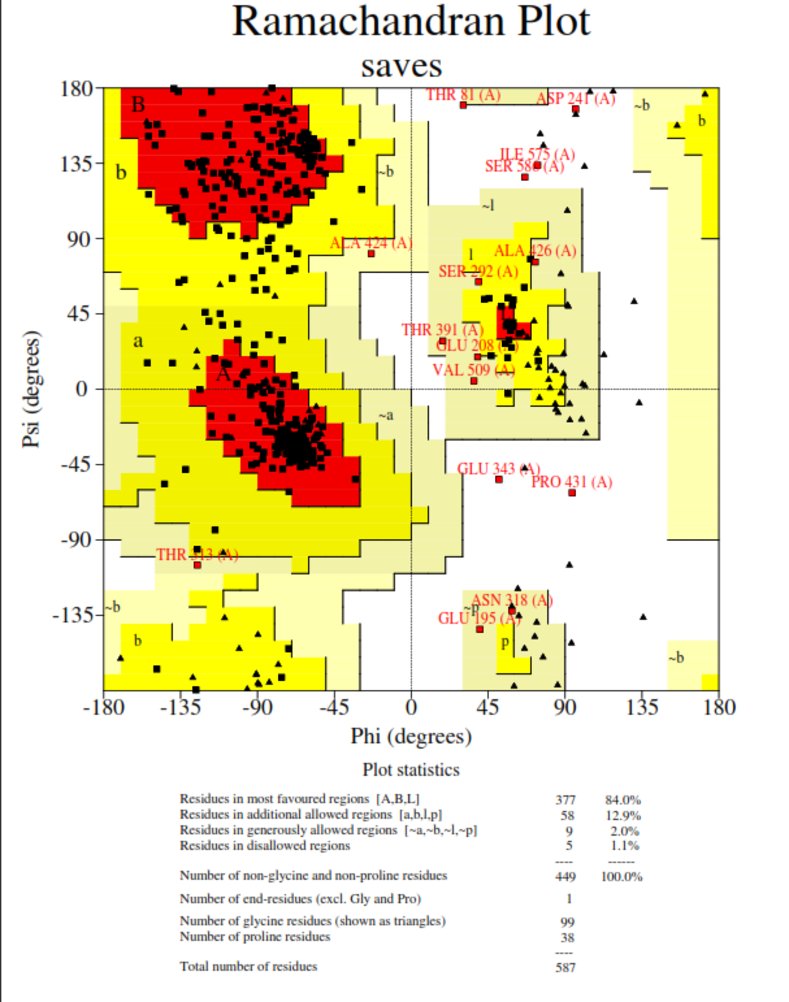

Supplement: S2 Fig — Before refinement, the amino acid residues in the most favored regions are found to be 84.0%. (TIF) [file pone.0312262.s002.tif]

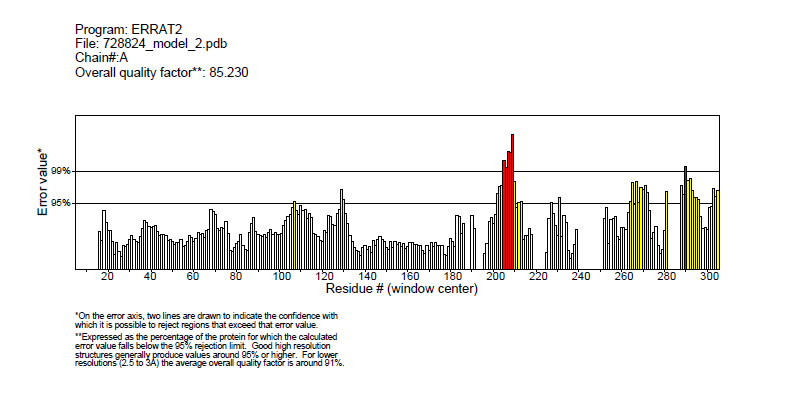

Supplement: S3 Fig — The Quality factor value of the refined vaccine is 85.230. (TIF) [file pone.0312262.s003.tif]

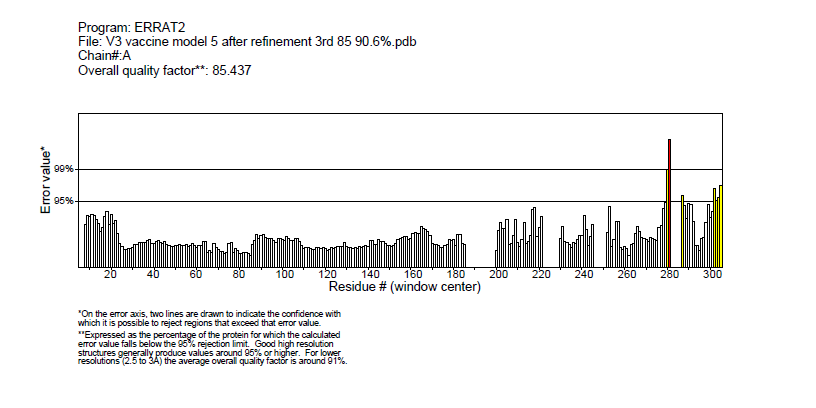

Supplement: S4 Fig — The Quality factor value of the refined vaccine remains 85.230. (TIF) [file pone.0312262.s004.tif]

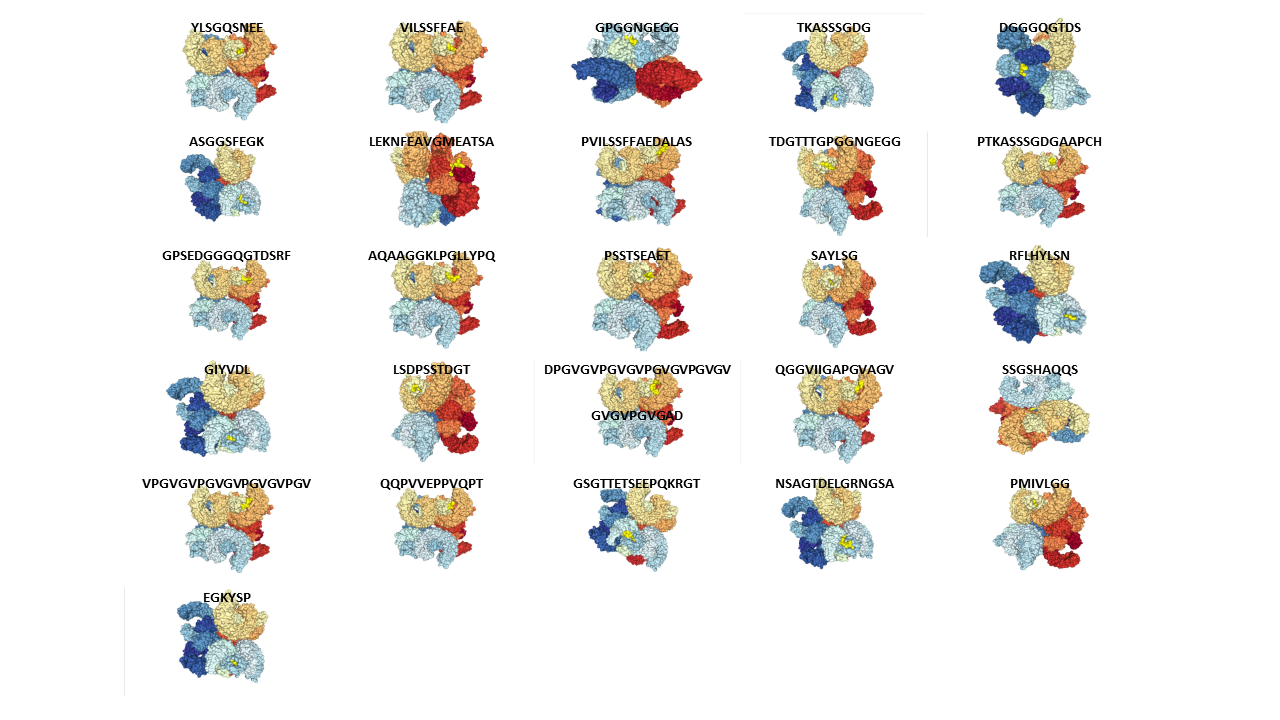

Supplement: S5 Fig — The peptides are represented in yellow colored surface model which are bonded to the rainbow-colored 3D surface models of Rp-105. (TIF) [file pone.0312262.s005.tif]

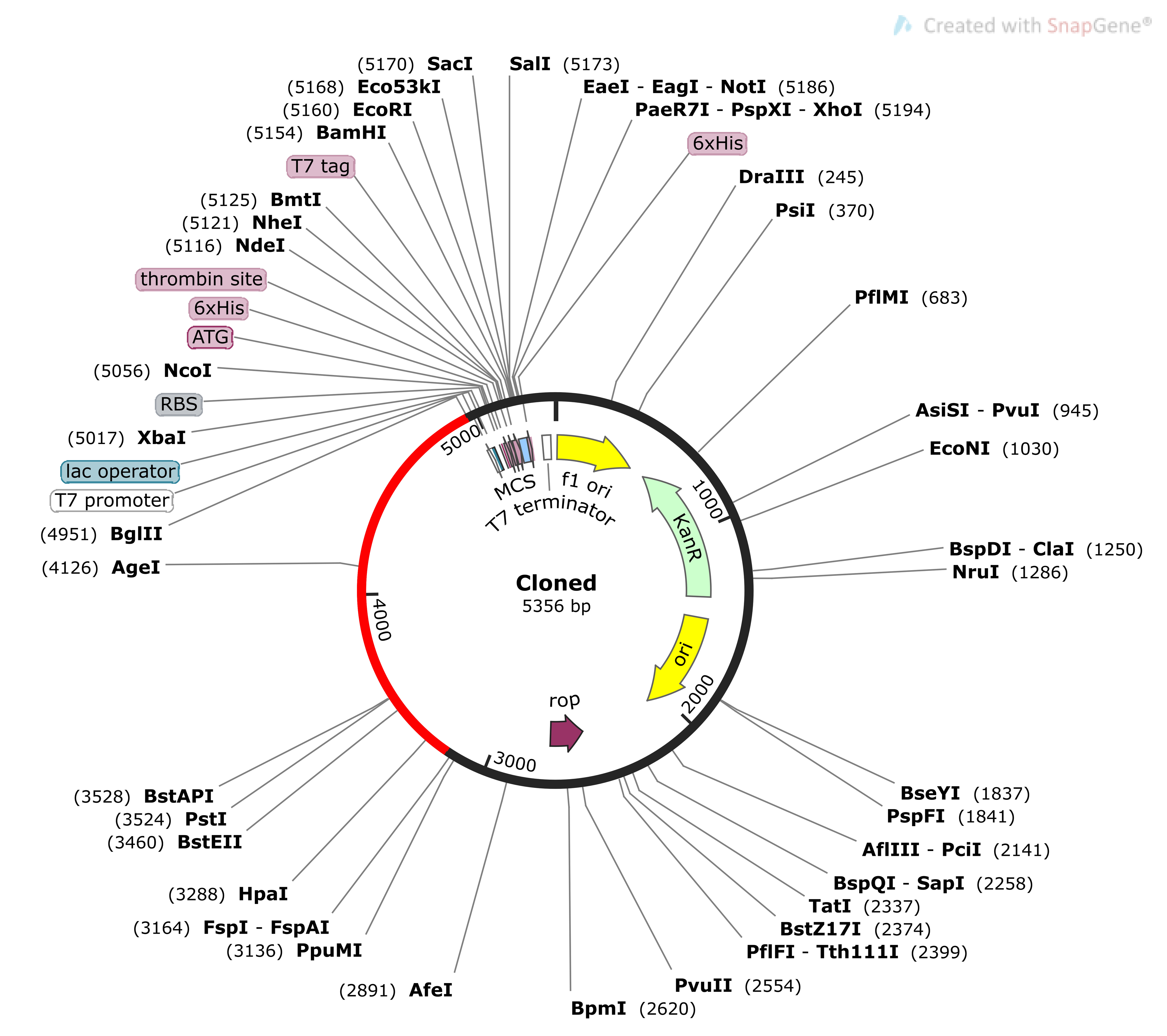

Supplement: S6 Fig — We can see here that the final cloned DNA is 5356 bp in length. It consists of all genes and restriction sites in a common plasmid. The DNA sequence of the chimeric vaccine fused to pET-28(+) is shown red. (TIF) [file pone.0312262.s006.tif]
