# Supplementary material for: Development of a multi-epitope chimeric vaccine in silico against Babesia bovis, Theileria annulata, and Anaplasma marginale using computational biology tools and reverse vaccinology approach
Source: PLoS One. 2025 Jan 24;20(1):e0312262. doi: 10.1371/journal.pone.0312262 (PMC11759392; doi:10.1371/journal.pone.0312262)
Supplement: S1 File — (DOCX) [file pone.0312262.s007.docx]

| **BoLA class I supertypes** | **BoLA allele members** |
| --- | --- |
| **BoLA-1** | BoLA-1:00901, BoLA-1:00902, BoLA-1:01901, BoLA-1:02001, BoLA-1:02101, BoLA-1:02301, BoLA-1:02801, BoLA-1:02901, BoLA-1:03101, BoLA-1:03102, BoLA-1:04201, BoLA-1:04901, BoLA-1:06101, BoLA-1:06701, BoLA-1:07401 |
| **BoLA-2** | BoLA-2:00501, BoLA-2:00601, BoLA-2:00602, BoLA-2:01801, BoLA-2:01802, BoLA-2:01601, BoLA-2:01201, BoLA-2:01602, BoLA-2:02201, BoLA-2:02601, BoLA-2:02602, BoLA-2:02603, BoLA-2:03001, BoLA-2:03202, BoLA-2:04301, BoLA-2:04401, BoLA-2:04402, BoLA-2:04501, BoLA-2:04601, BoLA-2:04701, BoLA-2:04801, BoLA-2:05401, BoLA-2:05501, BoLA-2:05601, BoLA-2:05701, BoLA-2:06001, BoLA-2:06201, BoLA-2:06901, BoLA-2:07001, BoLA-2:07101, BoLA-2:02501, BoLA-2:00801, BoLA-2:00802 |
| **BoLA-3** | BoLA-3:00101, BoLA-3:00102, BoLA-3:00103, BoLA-3:00201, BoLA-3:00401, BoLA-3:00402, BoLA-3:00403, BoLA-3:01001, BoLA-3:01101, BoLA-3:01701, BoLA-3:01702, BoLA-3:01703, BoLA-3:02701, BoLA-3:02702, BoLA-3:03501, BoLA-3:03601, BoLA-3:03701, BoLA-3:03801, BoLA-3:05001, BoLA-3:05101, BoLA-3:05201, BoLA-3:05801, BoLA-3:05901, BoLA-3:06501, BoLA-3:06601, BoLA-3:06602, BoLA-3:06801, BoLA-3:07301, BoLA-3:05301, BoLA-3:05002 |
| **BoLA-4** | BoLA-4:02401, BoLA-4:02402, BoLA-4:06301 |
| **BoLA-5** | BoLA-5:00301, BoLA-5:03901, BoLA-5:06401, BoLA-5:07201 |
| **BoLA-6** | BoLA-6:01301, BoLA-6:01302, BoLA-6:01401, BoLA-6:01402, BoLA-6:01501, BoLA-6:01502, BoLA-6:03401, BoLA-6:04001, BoLA-6:04101 |
| **Other alleles** | BoLA-amani.1, BoLA-AW10, BoLA-D18.4, BoLA-gb1.7, BoLA-HD6, BoLA-JSP.1, BoLA-T2a, BoLA-T2b, BoLA-T2c, BoLA-T5, and BoLA-T7 |

The table represents the BoLA class I supertypes and their related BoLA allele members which were selected for MHC I epitope prediction in IEDB server. Approximately 105 MHC I BoLA alleles were selected to predict promiscuous MHC I epitopes.
