# Supplementary material for: Development of a multi-epitope chimeric vaccine in silico against Babesia bovis, Theileria annulata, and Anaplasma marginale using computational biology tools and reverse vaccinology approach
Source: PLoS One. 2025 Jan 24;20(1):e0312262. doi: 10.1371/journal.pone.0312262 (PMC11759392; doi:10.1371/journal.pone.0312262)
Supplement: S2 File — (DOCX) [file pone.0312262.s008.docx]

The tables of all the ten MHC I epitopes of AMA-1 with their scores and percentile rank representing their affinities for different BOLA alleles. The peptide which has been selected for chimeric vaccine construction has been represented in bold letters. The BoLA alleles binding with the selected peptide possessing a percentile value >50 is highlighted as yellow. The BoLA allele that has bonded with the selected peptide with the lowest percentile rank is highlighted as green.

| Alleles | Peptides | Scores | Percentile rank |
| --- | --- | --- | --- |
| BoLA-2:01201  BoLA-T2a  BoLA-2:04401  BoLA-2:00501  BoLA-2:04501  BoLA-2:03202  BoLA-2:07101  BoLA-T2c  BoLA-3:03701  BoLA-5:00301  BoLA-1:00901  BoLA-2:04601  BoLA-1:06701  BoLA-2:04402  BoLA-5:03901  BoLA-3:06602  BoLA-3:03601  BoLA-3:05201  BoLA-2:05701  BoLA-2:05501  BoLA-3:00201  BoLA-JSP.1  BoLA-3:06601  BoLA-1:03102  BoLA-3:06501  BoLA-3:05801  BoLA-amani.1  BoLA-3:06801  BoLA-2:04701  BoLA-3:05101  BoLA-2:00602  BoLA-2:04301  BoLA-2:02501  BoLA-2:07001  BoLA-2:06201  BoLA-3:05001  BoLA-2:06001  BoLA-3:01001  BoLA-5:07201  BoLA-1:00902  BoLA-T5  BoLA-3:01702  BoLA-2:05601  BoLA-6:04001  BoLA-1:03101  BoLA-4:02402  BoLA-3:01703  BoLA-2:06901  BoLA-1:06101  BoLA-6:01402  BoLA-1:02301  BoLA-D18.4  BoLA-3:00103  BoLA-3:01701  BoLA-3:07301  BoLA-2:00601  BoLA-2:01602  BoLA-2:01601  BoLA-3:02701  BoLA-3:02702  BoLA-3:05901  BoLA-1:02801  BoLA-3:03801  BoLA-1:02001  BoLA-4:02401  BoLA-1:04901  BoLA-3:00401  BoLA-3:00402  BoLA-3:00403  BoLA-3:05301  BoLA-gb1.7  BoLA-2:02601  BoLA-2:02602  BoLA-2:02603  BoLA-1:04201  BoLA-3:05002  BoLA-2:01801  BoLA-2:01802  BoLA-6:01401  BoLA-2:00801  BoLA-2:03001  BoLA-3:00102  BoLA-T7  BoLA-5:06401  BoLA-3:00101  BoLA-AW10  BoLA-4:06301  BoLA-2:00802  BoLA-2:04801  BoLA-6:01302  BoLA-HD6  BoLA-6:01301  BoLA-3:03501  BoLA-3:01101  BoLA-1:01901  BoLA-6:01501  BoLA-2:02201  BoLA-6:04101  BoLA-T2b  BoLA-1:02101  BoLA-1:02901  BoLA-1:07401  BoLA-2:05401  BoLA-6:01502  BoLA-6:03401 | **VILSSFFAE** | 0.033706  0.033706  0.011193  0.005109  0.003835  0.003168  0.003143  0.002748  0.002282  0.001924  0.001309  0.001274  0.00124  0.001123  0.001049  0.000969  0.000953  0.000786  0.000739  0.000726  0.000702  0.000702  0.000527  0.000493  0.000484  0.000477  0.000466  0.000453  0.00045  0.000437  0.000385  0.000346  0.000324  0.000342  0.000304  0.000282  0.000274  0.000264  0.000261  0.000245  0.000245  0.000237  0.000207  0.000199  0.000193  0.000181  0.000171  0.00016  0.000137  0.000133  0.000131  0.000131  0.000127  0.000124  0.000104  8.3e-05  8.3e-05  7.3e-05  6.3e-05  6.3e-05  6.3e-05  5.6e-05  5.5e-05  5.4e-05  5.4e-05  5.1e-05  5e-05  5e-05  5e-05  5e-05  5e-05  4.9e-05  4.9e-05  4.9e-05  4.8e-05  4.7e-05  4.5e-05  4.5e-05  4.3e-05  4e-05  3.7e-05  3.7e-05  3.6e-05  2.9e-05  2.8e-05  2.8e-05  2.6e-05  2.4e-05  2.4e-05  2.3e-05  2.2e-05  1.4e-05  1.3e-05  1.2e-05  1e-05  9e-06  5e-06  5e-06  4e-06  4e-06  4e-06  4e-06  3e-06  3e-06  2e-06 | 5.7  5.7  22  24  20  17  19  29  47  45  21  32  39  27  24  38  35  27  40  34  39  39  42  43  56  54  28  45  48  36  45  42  32  31  53  59  39  37  38  36  36  50  39  53  52  44  64  35  55  53  50  50  37  55  36  63  63  56  63  63  68  42  72  58  41  60  66  66  66  66  66  63  63  63  65  74  59  59  65  43  52  53  65  52  63  63  48  51  66  40  52  47  52  74  77  70  67  70  74  74  62  84  67  75  84 |

| Alleles | Peptides | Scores | Percentile rank |
| --- | --- | --- | --- |
| BoLA-T2c  BoLA-2:01801  BoLA-2:01802  BoLA-2:00501  BoLA-3:03701  BoLA-3:01001  BoLA-2:03001  BoLA-3:05801  BoLA-2:04401  BoLA-2:00602  BoLA-2:06201  BoLA-2:04301  BoLA-2:00601  BoLA-2:01602  BoLA-1:06701  BoLA-4:02402  BoLA-2:06901  BoLA-3:03601  BoLA-3:06501  BoLA-3:03801  BoLA-3:06801  BoLA-2:04601  BoLA-2:06001  BoLA-3:01701  BoLA-5:00301  BoLA-2:05701  BoLA-3:00201  BoLA-JSP.1  BoLA-3:05001  BoLA-3:05901  BoLA-2:07001  BoLA-3:01703  BoLA-2:02501  BoLA-4:02401  BoLA-3:01702  BoLA-2:04801  BoLA-3:05101  BoLA-6:04001  BoLA-2:04501  BoLA-2:07101  BoLA-2:03202  BoLA-1:04901  BoLA-3:05002  BoLA-2:04402  BoLA-5:03901  BoLA-1:03101  BoLA-3:06601  BoLA-3:02701  BoLA-3:02702  BoLA-2:05601  BoLA-2:01601  BoLA-3:00103  BoLA-3:06602  BoLA-5:07201  BoLA-4:06301  BoLA-3:00101  BoLA-AW10  BoLA-2:04701  BoLA-1:02901  BoLA-T7  BoLA-3:00401  BoLA-3:00402  BoLA-3:00403  BoLA-3:05301  BoLA-gb1.7  BoLA-amani.1  BoLA-1:07401  BoLA-3:01101  BoLA-2:01201  BoLA-T2a  BoLA-3:05201  BoLA-2:02601  BoLA-2:02602  BoLA-2:02603  BoLA-2:02201  BoLA-3:00102  BoLA-1:02001  BoLA-2:05501  BoLA-1:06101  BoLA-1:02301  BoLA-D18.4  BoLA-2:00802  BoLA-1:03102  BoLA-6:04101  BoLA-T2b  BoLA-6:03401  BoLA-2:00801  BoLA-1:00902  BoLA-T5  BoLA-1:00901  BoLA-3:07301  BoLA-6:01402  BoLA-1:04201  BoLA-6:01401  BoLA-6:01501  BoLA-1:02801  BoLA-5:06401  BoLA-3:03501  BoLA-6:01301  BoLA-HD6  BoLA-6:01302  BoLA-6:01502  BoLA-1:01901  BoLA-2:05401  BoLA-1:02101 | VPVILSSFF | 0.190695  0.160236  0.160236  0.140923  0.135482  0.094206  0.072386  0.065873  0.063163  0.059101  0.051091  0.048813  0.048513  0.048513  0.046888  0.042651  0.037643  0.037457  0.036234  0.035141  0.032932  0.029117  0.023587  0.023198  0.023157  0.022205  0.020508  0.020508  0.020429  0.019364  0.019361  0.018862  0.018355  0.018112  0.017648  0.017407  0.016536  0.015738  0.014679  0.01447  0.014113  0.013718  0.013688  0.013263  0.01302  0.01289  0.012602  0.012582  0.012582  0.01218  0.011978  0.011181  0.010695  0.010377  0.009988  0.009576  0.009576  0.009159  0.007906  0.007677  0.007193  0.007193  0.007193  0.007193  0.007193  0.006493  0.005769  0.005762  0.005524  0.005524  0.005503  0.004977  0.004977  0.004977  0.004704  0.004375  0.003649  0.00332  0.003063  0.002993  0.002993  0.002919  0.002864  0.002775  0.002775  0.002101  0.001902  0.001712  0.001712  0.001642  0.001496  0.001403  0.001283  0.001253  0.001152  0.000796  0.000733  0.000578  0.000518  0.000518  0.000492  0.000413  0.000359  0.000267  6.3e-05 | 3.3  1.1  1.1  0.89  2.1  0.59  0.47  3.5  6.2  1.8  3.8  2.0  1.8  1.8  6.3  3.6  1.6  5.1  12  3.7  7.1  5.0  2.8  4.4  15  8.7  7.9  7.9  8.6  5.0  3.6  11  3.5  2.6  7.8  4.9  5.3  8.2  9.5  7.5  6.8  8.3  11  7.2  6.2  10  12  6.6  6.6  5.2  4.5  3.3  15  8.5  5.0  3.7  3.7  14  8.0  13  9.8  9.8  9.8  9.8  9.8  8.2  5.8  13  14  14  13  13  13  13  6.3  5.7  13  18  22  18  18  7.1  22  8.1  8.1  9.4  8.5  18  18  19  16  21  24  22  14  17  16  11  22  22  14  17  23  15  24 |

| Alleles | Peptide | Score | Percentile rank |
| --- | --- | --- | --- |
| BoLA-3:03701  BoLA-T2c  BoLA-2:04401  BoLA-3:05001  BoLA-5:00301  BoLA-3:03601  BoLA-1:06701  BoLA-1:06101  BoLA-2:00501  BoLA-3:06801  BoLA-2:05701  BoLA-3:05801  BoLA-3:01703  BoLA-3:01701  BoLA-2:05601  BoLA-3:01702  BoLA-2:04501  BoLA-2:04401  BoLA-3:05001  BoLA-5:00301  BoLA-3:03601  BoLA-1:06701  BoLA-1:06101  BoLA-3:06801  BoLA-2:00501  BoLA-2:05701  BoLA-3:05801  BoLA-3:01703  BoLA-3:01701  BoLA-2:05601  BoLA-3:01702  BoLA-2:04501  BoLA-3:06602  BoLA-3:05002  BoLA-3:06501  BoLA-2:07001  BoLA-2:03202  BoLA-2:04402  BoLA-2:01201  BoLA-T2a  BoLA-2:07101  BoLA-T7  BoLA-3:06601  BoLA-3:05201  BoLA-3:00201  BoLA-JSP.1  BoLA-3:00401  BoLA-3:00402  BoLA-3:00403  BoLA-3:05301  BoLA-gb1.7  BoLA-2:00602  BoLA-2:04301  BoLA-6:01401  BoLA-2:04601  BoLA-5:07201  BoLA-2:00601  BoLA-2:01602  BoLA-2:06201  BoLA-2:01801  BoLA-2:01802  BoLA-3:03801  BoLA-2:04701  BoLA-2:02501  BoLA-4:06301  BoLA-3:01101  BoLA-5:03901  BoLA-amani.1  BoLA-3:01001  BoLA-6:04001  BoLA-6:01402  BoLA-2:05501  BoLA-1:00901  BoLA-3:07301  BoLA-3:05101  BoLA-2:02601  BoLA-2:02602  BoLA-2:02603  BoLA-3:00103  BoLA-2:01601  BoLA-1:03101  BoLA-4:02402  BoLA-1:03102  BoLA-2:06001  BoLA-3:00101  BoLA-AW10  BoLA-1:01901  BoLA-1:04901  BoLA-3:02701  BoLA-3:02702  BoLA-5:06401  BoLA-1:02801  BoLA-2:00802  BoLA-1:00902  BoLA-T5  BoLA-1:02001  BoLA-1:04201  BoLA-3:00102  BoLA-1:02301  BoLA-D18.4  BoLA-2:00801  BoLA-2:04801  BoLA-4:02401  BoLA-6:03401  BoLA-2:06901  BoLA-2:05401  BoLA-6:01501  BoLA-1:07401  BoLA-1:02101  BoLA-1:02901  BoLA-2:03001  BoLA-3:03501  BoLA-2:02201  BoLA-6:01502  BoLA-6:01301  BoLA-6:04101  BoLA-HD6  BoLA-T2b  BoLA-6:01302 | PVILSSFFA | 0.010297  0.007375  0.004544  0.00368  0.003646  0.003612  0.002076  0.001501  0.001478  0.001451  0.001129  0.001045  0.000943  0.000873  0.000832  0.000761  0.000681  0.004544  0.00368  0.003646  0.003612  0.002076  0.001501  0.001478  0.001451  0.001129  0.001045  0.000943  0.000873  0.000832  0.000761  0.000681  0.000659  0.000569  0.000567  0.000566  0.00054  0.00043  0.000413  0.000413  0.000387  0.000385  0.000363  0.000315  0.000285  0.000285  0.000267  0.000267  0.000267  0.000267  0.000267  0.000265  0.000251  0.000223  0.000199  0.000195  0.000194  0.000194  0.000179  0.00017  0.00017  0.000164  0.000161  0.000158  0.000155  0.000151  0.000139  0.000129  0.000125  0.000122  0.000116  0.000114  0.000104  9.9e-05  9.6e-05  8.2e-05  8.2e-05  8.2e-05  7.7e-05  7.1e-05  6.5e-05  6.9e-05  6.5e-05  6.5e-05  5.8e-05  5.8e-05  5.6e-05  5.5e-05  5.1e-05  5.1e-05  5.1e-05  4.5e-05  4.3e-05  3.9e-05  3.9e-05  3.3e-05  3.2e-05  3.2e-05  2.7e-05  2.7e-05  2.6e-05  2.6e-05  2.4e-05  2.4e-05  2.1e-05  1.9e-05  1.9e-05  1.6e-05  1.3e-05  1.3e-05  1.2e-05  1.2e-05  1e-05  9e-06  5e-06  5e-06  5e-06  5e-06  2e-06 | 26  21  32  24  37  22  33  28  32  35  43  41  28  24  35  39  32  40  24  37  22  33  28  32  40  35  43  41  28  24  35  39  42  54  26  35  37  39  39  41  37  46  36  72  53  53  42  42  42  42  42  50  46  42  55  41  51  51  60  40  40  57  62  41  30  46  46  43  46  61  55  58  51  36  56  55  55  55  43  56  71  57  72  60  52  52  46  59  66  66  38  44  42  59  59  66  71  55  70  70  49  64  52  51  61  41  58  56  44  70  50  67  69  60  71  74  71  74  74 |

| Alleles | Peptide | Scores | Percentile rank |
| --- | --- | --- | --- |
| BoLA-5:00301  BoLA-2:04401  BoLA-T2c  BoLA-2:06201  BoLA-3:03601  BoLA-2:00501  BoLA-1:06701  BoLA-3:05201  BoLA-3:00201  BoLA-JSP.1  BoLA-3:06602  BoLA-3:05801  BoLA-2:00601  BoLA-2:01602  BoLA-2:04701  BoLA-2:04402  BoLA-3:01702  BoLA-2:05701  BoLA-3:03701  BoLA-3:06501  BoLA-3:06601  BoLA-1:03102  BoLA-2:04601  BoLA-3:01703  BoLA-2:04301  BoLA-1:03101  BoLA-3:01701  BoLA-1:06101  BoLA-2:06001  BoLA-2:01601  BoLA-6:04001  BoLA-2:02501  BoLA-4:02402  BoLA-T7  BoLA-3:06801  BoLA-2:03202  BoLA-2:04501  BoLA-3:05001  BoLA-3:05101  BoLA-3:05901  BoLA-2:00602  BoLA-5:07201  BoLA-2:01201  BoLA-T2a  BoLA-1:00901  BoLA-5:03901  BoLA-3:07301  BoLA-2:00801  BoLA-3:05002  BoLA-3:00103  BoLA-1:02301  BoLA-D18.4  BoLA-1:04901  BoLA-2:05501  BoLA-2:05601  BoLA-2:06901  BoLA-3:02701  BoLA-3:02702  BoLA-1:00902  BoLA-T5  BoLA-3:00401  BoLA-3:00402  BoLA-3:00403  BoLA-3:05301  BoLA-gb1.7  BoLA-amani.1  BoLA-2:07101  BoLA-4:02402  BoLA-T7  BoLA-3:06801  BoLA-2:03202  BoLA-2:04501  BoLA-3:05001  BoLA-3:05101  BoLA-3:05901  BoLA-2:00602  BoLA-5:07201  BoLA-2:01201  BoLA-T2a  BoLA-1:00901  BoLA-5:03901  BoLA-3:07301  BoLA-2:00801  BoLA-3:05002  BoLA-3:00103  BoLA-1:02301  BoLA-D18.4  BoLA-1:04901  BoLA-2:05501  BoLA-2:05601  BoLA-2:06901  BoLA-3:02701  BoLA-3:02702  BoLA-1:00902  BoLA-T5  BoLA-3:00401  BoLA-3:00402  BoLA-3:00403  BoLA-3:05301  BoLA-gb1.7  BoLA-amani.1  BoLA-2:07101 | ILSSFFAED | 0.007331  0.002401  0.001625  0.001471  0.001248  0.001154  0.000739  0.000552  0.000515  0.000515  0.000431  0.000393  0.000368  0.000368  0.000336  0.000309  0.0003  0.000267  0.000233  0.000224  0.000212  0.000207  0.00018  0.00018  0.000174  0.000173  0.000171  0.000156  0.000133  0.000123  0.00012  0.000114  0.000112  0.000111  0.000106  0.000105  9.8e-05  9.8e-05  9.2e-05  8.7e-05  8.6e-05  8e-05  7.6e-05  7.6e-05  7.2e-05  6.7e-05  6.3e-05  5.7e-05  5.3e-05  5.1e-05  4.9e-05  4.9e-05  4.3e-05  4e-05  3.7e-05  3.6e-05  3.4e-05  3.4e-05  3.2e-05  3.2e-05  3.1e-05  3.1e-05  3.1e-05  3.1e-05  3.1e-05  3e-05  2.6e-05  0.000112  0.000111  0.000106  0.000105  9.8e-05  9.8e-05  9.2e-05  8.7e-05  8.6e-05  8e-05  7.6e-05  7.6e-05  7.2e-05  6.7e-05  6.3e-05  5.7e-05  5.3e-05  5.1e-05  4.9e-05  4.9e-05  4.3e-05  4e-05  3.7e-05  3.6e-05  3.4e-05  3.4e-05  3.2e-05  3.2e-05  3.1e-05  3.1e-05  3.1e-05  3.1e-05  3.1e-05  3e-05  2.6e-05 | 28  40  33  32  32  43  46  30  44  44  49  57  42  42  52  41  47  53  77  66  53  55  57  63  52  58  50  54  49  48  61  46  50  51  62  56  64  73  56  63  66  52  63  63  56  56  41  38  72  49  63  63  62  71  60  54  72  72  62  62  72  72  72  72  72  63  76  50  51  62  56  64  73  56  63  66  52  63  63  56  56  41  38  72  49  63  63  62  71  60  54  72  72  62  62  72  72  72  72  72  63  76 |

| Alleles | Peptide | Score | Percentile rank |
| --- | --- | --- | --- |
| BoLA-3:03701  BoLA-2:04401  BoLA-2:04301  BoLA-1:04201  BoLA-2:05501  BoLA-1:00901  BoLA-2:06201  BoLA-1:03102  BoLA-2:04601  BoLA-3:05101  BoLA-1:03101  BoLA-2:04402  BoLA-1:00902  BoLA-T5  BoLA-2:04701  BoLA-3:05801  BoLA-2:04501  BoLA-2:03202  BoLA-3:05001  BoLA-3:03801  BoLA-3:06501  BoLA-2:02201  BoLA-2:07101  BoLA-3:05002  BoLA-3:06801  BoLA-1:02301  BoLA-D18.4  BoLA-2:01201  BoLA-T2a  BoLA-1:06701  BoLA-3:05901  BoLA-1:02001  BoLA-3:06602  BoLA-2:00602  BoLA-4:02402  BoLA-3:03601  BoLA-T2c  BoLA-2:06901  BoLA-2:04801  BoLA-3:00401  BoLA-3:00402  BoLA-3:00403  BoLA-3:05301  BoLA-gb1.7  BoLA-3:02701  BoLA-3:02702  BoLA-2:07001  BoLA-3:00201  BoLA-JSP.1  BoLA-6:01402  BoLA-2:06001  BoLA-amani.1  BoLA-6:04001  BoLA-3:01101  BoLA-3:06601  BoLA-3:01703  BoLA-4:02401  BoLA-5:00301  BoLA-2:02501  BoLA-2:00501  BoLA-2:05601  BoLA-3:01701  BoLA-2:02601  BoLA-2:02602  BoLA-2:02603  BoLA-3:01702  BoLA-2:00601  BoLA-2:01602  BoLA-2:05701  BoLA-3:01001  BoLA-3:05201  BoLA-1:04901  BoLA-3:00101  BoLA-AW10  BoLA-2:01601  BoLA-4:06301  BoLA-2:03001  BoLA-2:00801  BoLA-2:00802  BoLA-T7  BoLA-2:05401  BoLA-3:03501  BoLA-6:01302  BoLA-3:00102  BoLA-3:00103  BoLA-2:01801  BoLA-2:01802  BoLA-1:02901  BoLA-1:02101  BoLA-1:06101  BoLA-5:03901  BoLA-1:01901  BoLA-5:07201  BoLA-6:01501  BoLA-6:04101  BoLA-T2b  BoLA-6:01301  BoLA-HD6  BoLA-1:02801  BoLA-5:06401  BoLA-6:01502  BoLA-6:03401  BoLA-1:07401  BoLA-3:07301  BoLA-6:01401 | PNWFIRFLH | 0.043276  0.028707  0.014818  0.010465  0.009575  0.007667  0.006987  0.006949  0.006902  0.006357  0.005076  0.004871  0.004747  0.004747  0.004064  0.004056  0.003886  0.003818  0.003761  0.003346  0.003343  0.003181  0.002137  0.001885  0.001545  0.00141  0.00141  0.001304  0.001304  0.000938  0.000864  0.000856  0.000809  0.0006  0.000575  0.000533  0.000481  0.000437  0.000435  0.000404  0.000404  0.000404  0.000404  0.000404  0.000361  0.000361  0.000345  0.000319  0.000319  0.000268  0.000262  0.000252  0.000235  0.000228  0.000227  0.000223  0.000213  0.000208  0.0002  0.000179  0.000166  0.000102  0.000101  0.000101  0.000101  9.6e-05  9.4e-05  9.4e-05  9e-05  8.1e-05  6.2e-05  4.9e-05  4.7e-05  4.7e-05  4.4e-05  3.5e-05  3.4e-05  3.2e-05  2.9e-05  2.7e-05  2.4e-05  2.1e-05  2.1e-05  1.9e-05  1.6e-05  1.5e-05  1.5e-05  1.4e-05  1.2e-05  1.2e-05  1.1e-05  1e-05  1e-05  1e-05  1e-05  1e-05  8e-06  8e-06  5e-06  5e-06  5e-06  5e-06  4e-06  4e-06  2.6e-05 | 8.8  12  6.2  8.2  9.4  8.6  16  14  15  11  17  14  11  11  22  26  20  16  24  22  34  7.9  22  27  31  24  24  26  26  43  31  24  40  39  30  42  44  25  28  37  37  37  37  37  37  37  31  51  51  42  40  35  51  41  53  60  25  74  38  69  41  58  52  52  52  62  61  61  67  52  55  61  55  55  63  45  53  46  48  69  38  42  41  64  66  75  75  69  45  82  80  72  78  67  64  64  65  65  72  68  68  72  74  74  71 |

| Alleles | Peptide | Score | Percentile rank |
| --- | --- | --- | --- |
| BoLA-6:01402  BoLA-1:02901  BoLA-6:01401  BoLA-1:01901  BoLA-1:07401  BoLA-2:01601  BoLA-1:02001  BoLA-6:03401  BoLA-2:00602  BoLA-5:00301  BoLA-1:04201  BoLA-2:00601  BoLA-2:01602  BoLA-3:05001  BoLA-3:05002  BoLA-1:06701  BoLA-1:04901  BoLA-3:03801  BoLA-6:04101  BoLA-T2b  BoLA-3:03701  BoLA-2:00501  BoLA-1:06101  BoLA-3:01101  BoLA-2:01801  BoLA-2:01802  BoLA-1:02801  BoLA-3:06501  BoLA-3:05801  BoLA-6:01501  BoLA-3:05901  BoLA-3:01001  BoLA-2:06001  BoLA-2:05601  BoLA-1:03101  BoLA-3:02701  BoLA-3:02702  BoLA-2:04301  BoLA-2:06201  BoLA-2:02601  BoLA-2:02602  BoLA-2:02603  BoLA-3:00401  BoLA-3:00402  BoLA-3:00403  BoLA-3:05301  BoLA-gb1.7  BoLA-2:05701  BoLA-2:04801  BoLA-T2c  BoLA-2:04401  BoLA-3:06801  BoLA-3:01703  BoLA-3:03601  BoLA-1:02101  BoLA-3:01702  BoLA-1:03102  BoLA-5:07201  BoLA-3:07301  BoLA-3:06602  BoLA-2:04402  BoLA-2:02501  BoLA-6:01502  BoLA-2:04701  BoLA-3:00201  BoLA-JSP.1  BoLA-3:05201  BoLA-2:03001  BoLA-1:02301  BoLA-D18.4  BoLA-3:01701  BoLA-6:04001  BoLA-2:04601  BoLA-1:00901  BoLA-2:06901  BoLA-3:06601  BoLA-amani.1  BoLA-4:02402  BoLA-6:01301  BoLA-HD6  BoLA-5:06401  BoLA-1:00902  BoLA-T5  BoLA-3:05101  BoLA-2:05401  BoLA-3:00101  BoLA-4:06301  BoLA-AW10  BoLA-2:05501  BoLA-2:02201  BoLA-T7  BoLA-2:00802  BoLA-2:02601  BoLA-2:02602  BoLA-2:02603  BoLA-3:00401  BoLA-3:00402  BoLA-3:00403  BoLA-3:05301  BoLA-gb1.7  BoLA-2:05701  BoLA-2:04801  BoLA-T2c  BoLA-2:04401  BoLA-3:06801  BoLA-3:01703  BoLA-3:03601  BoLA-1:02101  BoLA-3:01702  BoLA-1:03102  BoLA-5:07201  BoLA-3:07301  BoLA-3:06602  BoLA-2:04402  BoLA-2:02501  BoLA-6:01502  BoLA-2:04701  BoLA-3:00201  BoLA-JSP.1  BoLA-3:05201  BoLA-2:03001  BoLA-1:02301  BoLA-D18.4  BoLA-3:01701  BoLA-6:04001  BoLA-2:04601  BoLA-1:00901  BoLA-2:06901  BoLA-3:06601  BoLA-amani.1  BoLA-4:02402  BoLA-6:01301  BoLA-HD6  BoLA-5:06401  BoLA-1:00902  BoLA-T5  BoLA-3:05101  BoLA-2:05401  BoLA-3:00101  BoLA-4:06301  BoLA-AW10  BoLA-2:05501  BoLA-2:02201  BoLA-T7  BoLA-2:00802  BoLA-2:03202  BoLA-2:00801  BoLA-6:01302  BoLA-5:03901  BoLA-2:07101  BoLA-3:00102  BoLA-3:03501  BoLA-2:04501  BoLA-3:00103  BoLA-4:02401  BoLA-2:01201  BoLA-T2a  BoLA-2:07001 | PETAVDSNI | 0.031766  0.015254  0.01139  0.011343  0.006911  0.006469  0.006328  0.00609  0.005425  0.003771  0.00352  0.003056  0.003056  0.002092  0.001984  0.001929  0.00171  0.001708  0.001663  0.001663  0.001508  0.001454  0.001322  0.001291  0.001232  0.001232  0.001108  0.000874  0.000832  0.000814  0.00075  0.000662  0.000654  0.000627  0.000622  0.000613  0.000613  0.000596  0.000592  0.000576  0.000576  0.000576  0.000562  0.000562  0.000562  0.000562  0.000562  0.000539  0.000457  0.000428  0.00042  0.000412  0.0004  0.000387  0.000339  0.000323  0.000245  0.00023  0.000222  0.000212  0.000208  0.000207  0.000206  0.000193  0.000167  0.000167  0.00016  0.000144  0.000143  0.000143  0.000134  0.000134  0.000125  0.000123  9.9e-05 41  9.8e-05 64  9.6e-05 47  9.4e-05 53  9.2e-05 37  9.2e-05 37  8.1e-05 33  7.5e-05 51  7.5e-05 51  6.4e-05 61  5.7e-05 28  5.5e-05 52  5.5e-05 40  5.5e-05 52  5.2e-05 68  5e-05 45  4.8e-05 61  4.6e-05 41  0.000576  0.000576  0.000576  0.000562  0.000562  0.000562  0.000562  0.000562  0.000539  0.000457  0.000428  0.00042  0.000412  0.0004  0.000387  0.000339  0.000323  0.000245  0.00023  0.000222  0.000212  0.000208  0.000207  0.000206  0.000193  0.000167  0.000167  0.00016  0.000144  0.000143  0.000143  0.000134  0.000134  0.000125  0.000123  9.9e-05  9.8e-05  9.6e-05  9.4e-05  9.2e-05  9.2e-05  8.1e-05  7.5e-05  7.5e-05  6.4e-05  5.7e-05  5.5e-05  5.5e-05  5.5e-05  5.2e-05  5e-05  4.8e-05  4.6e-05  4.4e-05  3.9e-05  3.4e-05  3.2e-05  3e-05  2.9e-05  2.6e-05  2.2e-05  2e-05  1.7e-05  1.6e-05  1.6e-05  8e-06 | 4.4  5.3  6.6  4.5  5.2  7.1  9.1  5.3  15  36  16  18  18  30  27  34  22  29  11  11  53  40  30  23  19  19  15  49  46  17  33  26  28  27  40  30  30  35  44  31  31  31  33  33  33  33  33  44  28  45  63  46  52  46  11  46  53  39  29  58  46  37  23  60  61  61  44  34  49  49  54  60  62  49  41  64  47  53  37  37  33  51  51  61  28  52  40  52  68  45  61  41  31  31  31  33  33  33  33  33  44  28  45  63  46  52  46  11  46  53  39  29  58  46  37  23  60  61  61  44  34  49  49  54  60  62  49  41  64  47  53  37  37  33  51  51  61  28  52  40  52  68  45  61  41  67  43  36  66  74  57  39  82  63  57  83  83  76 |

| Alleles | Peptide | Score | Percentile rank |
| --- | --- | --- | --- |
| BoLA-3:05001 | ETAVDSNIP | 0.008806 | 15 |
| BoLA-3:03601 |  | 0.008195 | 15 |
| BoLA-3:01703 |  | 0.005061 | 22 |
| BoLA-3:05002 |  | 0.005061 | 18 |
| BoLA-3:03701 |  | 0.004657 | 37 |
| BoLA-1:06701 |  | 0.004093 | 26 |
| BoLA-2:00501 |  | 0.003667 | 28 |
| BoLA-T2c |  | 0.003624 | 26 |
| BoLA-3:01702 |  | 0.003468 | 20 |
| BoLA-6:01402 |  | 0.002119 | 18 |
| BoLA-6:01401 |  | 0.001608 | 20 |
| BoLA-2:01601 |  | 0.001261 | 19 |
| BoLA-1:06101 |  | 0.001217 | 31 |
| BoLA-3:00401 |  | 0.001213 | 24 |
| BoLA-3:00402 |  | 0.001213 | 24 |
| BoLA-3:00403 |  | 0.001213 | 24 |
| BoLA-3:05301 |  | 0.001213 | 24 |
| BoLA-gb1.7 |  | 0.001213 | 24 |
| BoLA-2:01801 |  | 0.000876 | 22 |
| BoLA-2:01802 |  | 0.000876 | 22 |
| BoLA-3:06801 |  | 0.000823 | 38 |
| BoLA-3:01701 |  | 0.000822 | 29 |
| BoLA-2:00601 |  | 0.000767 | 33 |
| BoLA-2:01602 |  | 0.000767 | 33 |
| BoLA-2:04401 |  | 0.000572 | 59 |
| BoLA-2:04301 |  | 0.000546 | 36 |
| BoLA-2:05601 |  | 0.00052 | 29 |
| BoLA-2:04402 |  | 0.000515 | 35 |
| BoLA-2:04501 |  | 0.000502 | 43 |
| BoLA-1:01901 |  | 0.000435 | 21 |
| BoLA-3:05901 |  | 0.00043 | 40 |
| BoLA-2:06201 |  | 0.000385 | 49 |
| BoLA-3:01101 |  | 0.000365 | 36 |
| BoLA-3:05801 |  | 0.000361 | 58 |
| BoLA-3:02701 |  | 0.000321 | 39 |
| BoLA-3:02702 |  | 0.000321 | 39 |
| BoLA-T7 |  | 0.000316 | 39 |
| BoLA-3:03801 |  | 0.000312 | 49 |
| BoLA-4:06301 |  | 0.000301 | 24 |
| BoLA-5:07201 |  | 0.000296 | 36 |
| BoLA-3:06501 |  | 0.000279 | 63 |
| BoLA-2:00602 |  | 0.000269 | 50 |
| BoLA-3:01001 |  | 0.000265 | 36 |
| BoLA-2:07001 |  | 0.000238 | 35 |
| BoLA-2:05701 |  | 0.000237 | 55 |
| BoLA-2:07101 |  | 0.000233 | 48 |
| BoLA-5:00301 |  | 0.00023 | 73 |
| BoLA-1:02001 |  | 0.000227 | 38 |
| BoLA-2:05401 |  | 0.000223 | 16 |
| BoLA-1:02801 |  | 0.000209 | 28 |
| BoLA-2:04701 |  | 0.000186 | 60 |
| BoLA-2:04801 |  | 0.000138 | 42 |
| BoLA-1:02901 |  | 0.000135 | 40 |
| BoLA-2:02601 |  | 0.000133 | 48 |
| BoLA-2:02602 |  | 0.000133 | 48 |
| BoLA-2:02603 |  | 0.000133 | 48 |
| BoLA-2:06001 |  | 0.000131 | 50 |
| BoLA-2:03202 |  | 0.00013 | 53 |
| BoLA-amani.1 |  | 0.000119 | 44 |
| BoLA-3:06602 |  | 9.5e-05 | 69 |
| BoLA-2:01201 |  | 9.3e-05 | 60 |
| BoLA-T2a |  | 9.3e-05 | 60 |
| BoLA-2:02501 |  | 8.9e-05 | 49 |
| BoLA-2:00802 |  | 8.8e-05 | 33 |
| BoLA-3:00201 |  | 8.1e-05 | 73 |
| BoLA-JSP.1 |  | 8.1e-05 | 73 |
| BoLA-1:04201 |  | 7.9e-05 | 59 |
| BoLA-6:03401 |  | 7.8e-05 | 36 |
| BoLA-1:00901 |  | 6.9e-05 | 57 |
| BoLA-2:04601 |  | 6.8e-05 | 69 |
| BoLA-2:00801 |  | 6.1e-05 | 37 |
| BoLA-3:06601 |  | 6.1e-05 | 70 |
| BoLA-3:05201 |  | 5.9e-05 | 56 |
| BoLA-6:04001 |  | 5.7e-05 | 73 |
| BoLA-2:05501 |  | 5.2e-05 | 68 |
| BoLA-1:07401 |  | 5e-05 | 41 |
| BoLA-2:03001 |  | 5e-05 | 48 |
| BoLA-3:05101 |  | 4.3e-05 | 66 |
| BoLA-1:03101 |  | 4.2e-05 | 77 |
| BoLA-5:03901 |  | 3.8e-05 | 64 |
| BoLA-1:04901 |  | 3.7e-05 | 64 |
| BoLA-3:07301 |  | 3.5e-05 | 47 |
| BoLA-1:03102 |  | 3.2e-05 | 81 |
| BoLA-3:00101 |  | 2.8e-05 | 63 |
| BoLA-AW10 |  | 2.8e-05 | 63 |
| BoLA-4:02402 |  | 2.7e-05 | 69 |
| BoLA-6:01501 |  | 2.6e-05 | 54 |
| BoLA-1:02101 |  | 2.5e-05 | 35 |
| BoLA-3:00102 |  | 2.4e-05 | 60 |
| BoLA-3:00103 |  | 2.2e-05 | 61 |
| BoLA-6:04101 |  | 2.2e-05 | 52 |
| BoLA-T2b |  | 2.2e-05 | 52 |
| BoLA-1:00902 |  | 2.1e-05 | 67 |
| BoLA-T5 |  | 2.1e-05 | 67 |
| BoLA-6:01502 |  | 2e-05 | 49 |
| BoLA-1:02301 |  | 1.9e-05 | 75 |
| BoLA-D18.4 |  | 1.9e-05 | 75 |
| BoLA-2:06901 |  | 1.6e-05 | 64 |
| BoLA-5:06401 |  | 1.5e-05 | 54 |
| BoLA-4:02401 |  | 9e-06 | 66 |
| BoLA-3:03501 |  | 4e-06 | 65 |
| BoLA-2:02201 |  | 3e-06 | 86 |
| BoLA-6:01301 |  | 2e-06 | 83 |
| BoLA-6:01302 |  | 2e-06 | 74 |
| BoLA-HD6 |  | 2e-06 | 83 |

| Alleles | Peptide | Score | Percentile rank |
| --- | --- | --- | --- |
| BoLA-3:00201 | HGSGIYVDL | 0.37677 | 0.26 |
| BoLA-JSP.1 |  | 0.37677 | 0.26 |
| BoLA-3:06501 |  | 0.370093 | 0.43 |
| BoLA-6:04001 |  | 0.360216 | 0.17 |
| BoLA-T2c |  | 0.2604 | 2.4 |
| BoLA-3:01703 |  | 0.240689 | 0.62 |
| BoLA-3:01702 |  | 0.23258 | 0.4 |
| BoLA-3:01701 |  | 0.212595 | 0.44 |
| BoLA-1:06701 |  | 0.201464 | 1.1 |
| BoLA-3:06602 |  | 0.19742 | 0.85 |
| BoLA-3:06801 |  | 0.158385 | 1.1 |
| BoLA-3:05801 |  | 0.157341 | 0.85 |
| BoLA-3:06601 |  | 0.156598 | 1.1 |
| BoLA-3:03701 |  | 0.143642 | 1.9 |
| BoLA-3:03801 |  | 0.118882 | 0.5 |
| BoLA-3:01101 |  | 0.105758 | 1.8 |
| BoLA-3:05201 |  | 0.101359 | 1.1 |
| BoLA-3:03601 |  | 0.096303 | 1.8 |
| BoLA-3:00101 |  | 0.092907 | 0.18 |
| BoLA-AW10 |  | 0.092907 | 0.18 |
| BoLA-5:00301 |  | 0.090746 | 4.2 |
| BoLA-2:00501 |  | 0.090279 | 1.9 |
| BoLA-2:04401 |  | 0.081454 | 4.7 |
| BoLA-3:05002 |  | 0.071168 | 2.4 |
| BoLA-T7 |  | 0.069837 | 2.2 |
| BoLA-2:04601 |  | 0.068342 | 1.9 |
| BoLA-3:05101 |  | 0.066052 | 1.3 |
| BoLA-5:07201 |  | 0.058795 | 1.7 |
| BoLA-3:00401 |  | 0.058588 | 1.5 |
| BoLA-3:00402 |  | 0.058588 | 1.5 |
| BoLA-3:00403 |  | 0.058588 | 1.5 |
| BoLA-3:05301 |  | 0.058588 | 1.5 |
| BoLA-gb1.7 |  | 0.058588 | 1.5 |
| BoLA-2:04701 |  | 0.056875 | 3.0 |
| BoLA-2:04301 |  | 0.05554 | 1.7 |
| BoLA-3:00103 |  | 0.05404 | 0.54 |
| BoLA-3:05001 |  | 0.053662 | 3.8 |
| BoLA-3:00102 |  | 0.051955 | 0.35 |
| BoLA-4:06301 |  | 0.041806 | 1.5 |
| BoLA-2:05701 |  | 0.04178 | 5.3 |
| BoLA-5:03901 |  | 0.038374 | 2.2 |
| BoLA-3:07301 |  | 0.035456 | 2.5 |
| BoLA-2:04402 |  | 0.030217 | 3.7 |
| BoLA-2:05601 |  | 0.028368 | 2.2 |
| BoLA-3:05901 |  | 0.022538 | 4.4 |
| BoLA-2:03202 |  | 0.021081 | 5.1 |
| BoLA-3:02701 |  | 0.019191 | 5.0 |
| BoLA-3:02702 |  | 0.019191 | 5.0 |
| BoLA-2:02601 |  | 0.017681 | 6.1 |
| BoLA-2:02602 |  | 0.017681 | 6.1 |
| BoLA-2:02603 |  | 0.017681 | 6.1 |
| BoLA-1:04901 |  | 0.017621 | 7.1 |
| BoLA-2:07001 |  | 0.017493 | 3.9 |
| BoLA-2:03001 |  | 0.01626 | 2.9 |
| BoLA-2:06901 |  | 0.015379 | 3.9 |
| BoLA-2:06201 |  | 0.015256 | 9.4 |
| BoLA-3:01001 |  | 0.015243 | 4.7 |
| BoLA-1:06101 |  | 0.014394 | 9.4 |
| BoLA-2:04501 |  | 0.01399 | 9.8 |
| BoLA-3:03501 |  | 0.01347 | 0.92 |
| BoLA-2:07101 |  | 0.011823 | 8.7 |
| BoLA-2:04801 |  | 0.010019 | 6.9 |
| BoLA-2:01801 |  | 0.008705 | 7.5 |
| BoLA-2:01802 |  | 0.008705 | 7.5 |
| BoLA-2:05501 |  | 0.008651 | 11 |
| BoLA-2:00602 |  | 0.006746 | 13 |
| BoLA-2:01201 |  | 0.006237 | 14 |
| BoLA-T2a |  | 0.006237 | 14 |
| BoLA-2:00601 |  | 0.005237 | 14 |
| BoLA-2:01602 |  | 0.005237 | 14 |
| BoLA-2:02501 |  | 0.005181 | 8.6 |
| BoLA-1:02901 |  | 0.00517 | 11 |
| BoLA-5:06401 |  | 0.004682 | 5.9 |
| BoLA-1:02301 |  | 0.004536 | 15 |
| BoLA-D18.4 |  | 0.004536 | 15 |
| BoLA-2:06001 |  | 0.003952 | 12 |
| BoLA-amani.1 |  | 0.003695 | 11 |
| BoLA-1:03101 |  | 0.00345 | 21 |
| BoLA-6:01501 |  | 0.003361 | 7.9 |
| BoLA-1:03102 |  | 0.003302 | 20 |
| BoLA-1:02801 |  | 0.00303 | 9.0 |
| BoLA-4:02402 |  | 0.002652 | 17 |
| BoLA-6:01301 |  | 0.002519 | 13 |
| BoLA-HD6 |  | 0.002519 | 13 |
| BoLA-1:07401 |  | 0.002444 | 9.4 |
| BoLA-2:05401 |  | 0.002128 | 4.5 |
| BoLA-1:00902 |  | 0.001563 | 18 |
| BoLA-T5 |  | 0.001563 | 18 |
| BoLA-6:01302 |  | 0.001435 | 8.5 |
| BoLA-1:00901 |  | 0.001377 | 21 |
| BoLA-2:02201 |  | 0.001213 | 14 |
| BoLA-6:01502 |  | 0.001081 | 11 |
| BoLA-2:01601 |  | 0.000778 | 24 |
| BoLA-2:00802 |  | 0.000742 | 14 |
| BoLA-6:04101 |  | 0.000676 | 16 |
| BoLA-T2b |  | 0.000676 | 16 |
| BoLA-1:02001 |  | 0.000607 | 27 |
| BoLA-4:02401 |  | 0.00057 | 16 |
| BoLA-6:01402 |  | 0.000499 | 34 |
| BoLA-6:01401 |  | 0.000461 | 33 |
| BoLA-6:03401 |  | 0.000457 | 19 |
| BoLA-1:04201 |  | 0.000449 | 36 |
| BoLA-2:00801 |  | 0.000349 | 19 |
| BoLA-1:01901 |  | 0.000307 | 24 |
| BoLA-1:02101 |  | 7.4e-05 | 23 |

| Alleles | Peptide | Score | Percentile rank |
| --- | --- | --- | --- |
| BoLA-2:04501 | LTAIGSPLE | 0.010101 | 12 |
| BoLA-2:04401 |  | 0.008869 | 24 |
| BoLA-2:01201 |  | 0.008463 | 12 |
| BoLA-T2a |  | 0.008463 | 12 |
| BoLA-2:07001 |  | 0.006652 | 8.1 |
| BoLA-2:03202 |  | 0.006058 | 12 |
| BoLA-3:03701 |  | 0.005225 | 36 |
| BoLA-2:07101 |  | 0.004066 | 17 |
| BoLA-1:06701 |  | 0.003453 | 27 |
| BoLA-2:04402 |  | 0.003078 | 17 |
| BoLA-2:00501 |  | 0.002945 | 30 |
| BoLA-amani.1 |  | 0.002719 | 13 |
| BoLA-T2c |  | 0.002254 | 30 |
| BoLA-2:04501 |  | 0.010101 | 12 |
| BoLA-2:04401 |  | 0.008869 | 24 |
| BoLA-2:01201 |  | 0.008463 | 12 |
| BoLA-T2a |  | 0.008463 | 12 |
| BoLA-2:07001 |  | 0.006652 | 8.1 |
| BoLA-2:03202 |  | 0.006058 | 12 |
| BoLA-3:03701 |  | 0.005225 | 36 |
| BoLA-2:07101 |  | 0.004066 | 17 |
| BoLA-1:06701 |  | 0.003453 | 27 |
| BoLA-2:04402 |  | 0.003078 | 17 |
| BoLA-2:00501 |  | 0.002945 | 30 |
| BoLA-amani.1 |  | 0.002719 | 13 |
| BoLA-T2c |  | 0.002254 | 30 |
| BoLA-2:04701 |  | 0.001247 | 35 |
| BoLA-2:04601 |  | 0.000975 | 35 |
| BoLA-3:01703 |  | 0.000862 | 42 |
| BoLA-3:06801 |  | 0.000801 | 38 |
| BoLA-3:06602 |  | 0.000791 | 41 |
| BoLA-3:06601 |  | 0.000698 | 38 |
| BoLA-3:05001 |  | 0.00069 | 45 |
| BoLA-3:05201 |  | 0.000616 | 29 |
| BoLA-5:00301 |  | 0.000614 | 61 |
| BoLA-3:05801 |  | 0.000607 | 51 |
| BoLA-2:04301 |  | 0.000417 | 39 |
| BoLA-4:02402 |  | 0.000377 | 35 |
| BoLA-T7 |  | 0.00036 | 38 |
| BoLA-3:05101 |  | 0.000354 | 39 |
| BoLA-1:03102 |  | 0.000346 | 48 |
| BoLA-3:00401 |  | 0.000338 | 39 |
| BoLA-3:00402 |  | 0.000338 | 39 |
| BoLA-3:00403 |  | 0.000338 | 39 |
| BoLA-3:05301 |  | 0.000338 | 39 |
| BoLA-gb1.7 |  | 0.000338 | 39 |
| BoLA-2:05501 |  | 0.000334 | 43 |
| BoLA-3:01001 |  | 0.000329 | 34 |
| BoLA-3:01101 |  | 0.000324 | 37 |
| BoLA-3:01702 |  | 0.000321 | 46 |
| BoLA-3:03801 |  | 0.000309 | 49 |
| BoLA-3:00201 |  | 0.000308 | 51 |
| BoLA-JSP.1 |  | 0.000308 | 51 |
| BoLA-3:06501 |  | 0.000299 | 62 |
| BoLA-1:06101 |  | 0.000266 | 47 |
| BoLA-6:01402 |  | 0.000263 | 43 |
| BoLA-3:05002 |  | 0.000253 | 52 |
| BoLA-5:03901 |  | 0.000245 | 39 |
| BoLA-3:01701 |  | 0.000244 | 45 |
| BoLA-1:03101 |  | 0.000226 | 54 |
| BoLA-1:00901 |  | 0.000225 | 41 |
| BoLA-3:07301 |  | 0.000191 | 30 |
| BoLA-2:00601 |  | 0.000187 | 51 |
| BoLA-2:01602 |  | 0.000187 | 51 |
| BoLA-2:00602 |  | 0.000159 | 57 |
| BoLA-5:07201 |  | 0.000159 | 43 |
| BoLA-4:06301 |  | 0.000151 | 30 |
| BoLA-1:00902 |  | 0.000145 | 43 |
| BoLA-T5 |  | 0.000145 | 43 |
| BoLA-2:06901 |  | 0.00014 | 37 |
| BoLA-3:05901 |  | 0.000115 | 59 |
| BoLA-6:04001 |  | 0.000111 | 63 |
| BoLA-2:03001 |  | 0.000108 | 38 |
| BoLA-2:01801 |  | 0.000103 | 47 |
| BoLA-2:01802 |  | 0.000103 | 47 |
| BoLA-3:02701 |  | 9.2e-05 | 57 |
| BoLA-3:02702 |  | 9.2e-05 | 57 |
| BoLA-5:06401 |  | 8.3e-05 | 33 |
| BoLA-1:02001 |  | 8.1e-05 | 52 |
| BoLA-2:00801 |  | 8e-05 | 34 |
| BoLA-1:02301 |  | 6.5e-05 | 59 |
| BoLA-D18.4 |  | 6.5e-05 | 59 |
| BoLA-2:06001 |  | 6.4e-05 | 61 |
| BoLA-2:01601 |  | 6.2e-05 | 58 |
| BoLA-2:00802 |  | 5.8e-05 | 38 |
| BoLA-3:00102 |  | 5.6e-05 | 47 |
| BoLA-3:00103 |  | 5.5e-05 | 48 |
| BoLA-2:02501 |  | 5.2e-05 | 58 |
| BoLA-2:02601 |  | 5.2e-05 | 62 |
| BoLA-2:02602 |  | 5.2e-05 | 62 |
| BoLA-2:02603 |  | 5.2e-05 | 62 |
| BoLA-2:05401 |  | 4.5e-05 | 31 |
| BoLA-4:02401 |  | 4.3e-05 | 44 |
| BoLA-1:04201 |  | 4e-05 | 68 |
| BoLA-2:04801 |  | 3.8e-05 | 59 |
| BoLA-1:04901 |  | 3.7e-05 | 64 |
| BoLA-6:01401 |  | 3.7e-05 | 67 |
| BoLA-3:00101 |  | 3.4e-05 | 60 |
| BoLA-AW10 |  | 3.4e-05 | 60 |
| BoLA-1:07401 |  | 1.7e-05 | 55 |
| BoLA-3:07301 |  | 0.000191 | 30 |
| BoLA-2:00601 |  | 0.000187 | 51 |
| BoLA-2:01602 |  | 0.000187 | 51 |
| BoLA-2:00602 |  | 0.000159 | 57 |
| BoLA-5:07201 |  | 0.000159 | 43 |
| BoLA-4:06301 |  | 0.000151 | 30 |
| BoLA-1:00902 |  | 0.000145 | 43 |
| BoLA-T5 |  | 0.000145 | 43 |
| BoLA-2:06901 |  | 0.00014 | 37 |
| BoLA-3:05901 |  | 0.000115 | 59 |
| BoLA-6:04001 |  | 0.000111 | 63 |
| BoLA-2:03001 |  | 0.000108 | 38 |
| BoLA-2:01801 |  | 0.000103 | 47 |
| BoLA-2:01802 |  | 0.000103 | 47 |
| BoLA-3:02701 |  | 9.2e-05 | 57 |
| BoLA-3:02702 |  | 9.2e-05 | 57 |
| BoLA-5:06401 |  | 8.3e-05 | 33 |
| BoLA-1:02001 |  | 8.1e-05 | 52 |
| BoLA-2:00801 |  | 8e-05 | 34 |
| BoLA-1:02301 |  | 6.5e-05 | 59 |
| BoLA-D18.4 |  | 6.5e-05 | 59 |
| BoLA-2:06001 |  | 6.4e-05 | 61 |
| BoLA-2:01601 |  | 6.2e-05 | 58 |
| BoLA-2:00802 |  | 5.8e-05 | 38 |
| BoLA-3:00102 |  | 5.6e-05 | 47 |
| BoLA-3:00103 |  | 5.5e-05 | 48 |
| BoLA-2:02501 |  | 5.2e-05 | 58 |
| BoLA-2:02601 |  | 5.2e-05 | 62 |
| BoLA-2:02602 |  | 5.2e-05 | 62 |
| BoLA-2:02603 |  | 5.2e-05 | 62 |
| BoLA-2:05401 |  | 4.5e-05 | 31 |
| BoLA-4:02401 |  | 4.3e-05 | 44 |
| BoLA-1:04201 |  | 4e-05 | 68 |
| BoLA-2:04801 |  | 3.8e-05 | 59 |
| BoLA-1:04901 |  | 3.7e-05 | 64 |
| BoLA-6:01401 |  | 3.7e-05 | 67 |
| BoLA-3:00101 |  | 3.4e-05 | 60 |
| BoLA-AW10 |  | 3.4e-05 | 60 |
| BoLA-1:07401 |  | 1.7e-05 | 55 |
| BoLA-1:07401 |  | 1.7e-05 | 55 |
| BoLA-3:03501 |  | 1.5e-05 | 46 |
| BoLA-1:02801 |  | 1.4e-05 | 59 |
| BoLA-1:02901 |  | 1.3e-05 | 70 |
| BoLA-6:01501 |  | 1.3e-05 | 63 |
| BoLA-6:01502 |  | 1e-05 | 58 |
| BoLA-6:03401 |  | 9e-06 | 64 |
| BoLA-1:01901 |  | 8e-06 | 76 |
| BoLA-2:02201 |  | 6e-06 | 76 |
| BoLA-1:02101 |  | 4e-06 | 62 |
| BoLA-6:01301 |  | 4e-06 | 74 |
| BoLA-HD6 |  | 4e-06 | 74 |
| BoLA-6:04101 |  | 3e-06 | 82 |
| BoLA-T2b |  | 3e-06 | 82 |
| BoLA-6:01302 |  | 2e-06 | 74 |

| Alleles | Peptide | Score | Percentile rank |
| --- | --- | --- | --- |
| BoLA-2:06201 | TAIGSPLEY | 0.761719 | 0.01 |
| BoLA-1:00901 |  | 0.713974 | 0.11 |
| BoLA-2:04501 |  | 0.670997 | 0.01 |
| BoLA-3:03701 |  | 0.634888 | 0.01 |
| BoLA-2:04401 |  | 0.587345 | 0.03 |
| BoLA-2:07001 |  | 0.554396 | 0.01 |
| BoLA-amani.1 |  | 0.5529 | 0.17 |
| BoLA-1:00902 |  | 0.519999 | 0.07 |
| BoLA-T5 |  | 0.519999 | 0.07 |
| BoLA-2:04601 |  | 0.482249 | 0.01 |
| BoLA-4:02402 |  | 0.480304 | 0.23 |
| BoLA-2:01201 |  | 0.472351 | 0.52 |
| BoLA-T2a |  | 0.472351 | 0.52 |
| BoLA-2:04402 |  | 0.471028 | 0.01 |
| BoLA-2:07101 |  | 0.459025 | 0.02 |
| BoLA-1:02301 |  | 0.450359 | 0.4 |
| BoLA-D18.4 |  | 0.450359 | 0.4 |
| BoLA-2:06901 |  | 0.447092 | 0.01 |
| BoLA-3:05101 |  | 0.42943 | 0.01 |
| BoLA-2:04301 |  | 0.410228 | 0.01 |
| BoLA-3:05801 |  | 0.409984 | 0.02 |
| BoLA-2:03202 |  | 0.36103 | 0.06 |
| BoLA-2:00801 |  | 0.358307 | 0.23 |
| BoLA-2:00802 |  | 0.337676 | 0.11 |
| BoLA-2:04801 |  | 0.332171 | 0.29 |
| BoLA-3:06801 |  | 0.329017 | 0.18 |
| BoLA-2:04701 |  | 0.315392 | 0.06 |
| BoLA-T2c |  | 0.30097 | 2.0 |
| BoLA-2:05501 |  | 0.294836 | 0.07 |
| BoLA-1:03101 |  | 0.281587 | 0.26 |
| BoLA-4:02401 |  | 0.276173 | 0.3 |
| BoLA-2:05701 |  | 0.247583 | 0.42 |
| BoLA-3:03601 |  | 0.24497 | 0.28 |
| BoLA-2:05601 |  | 0.189625 | 0.07 |
| BoLA-1:06701 |  | 0.1869 | 1.3 |
| BoLA-3:05002 |  | 0.167863 | 0.48 |
| BoLA-4:06301 |  | 0.165507 | 0.18 |
| BoLA-3:01703 |  | 0.164145 | 1.3 |
| BoLA-1:03102 |  | 0.160372 | 0.41 |
| BoLA-2:00602 |  | 0.155035 | 0.3 |
| BoLA-3:05001 |  | 0.152334 | 1.1 |
| BoLA-2:00601 |  | 0.146493 | 0.21 |
| BoLA-2:01602 |  | 0.146493 | 0.21 |
| BoLA-1:04201 |  | 0.144013 | 0.59 |
| BoLA-2:02501 |  | 0.114207 | 0.34 |
| BoLA-3:01702 |  | 0.109257 | 1.4 |
| BoLA-1:06101 |  | 0.108167 | 1.4 |
| BoLA-3:01101 |  | 0.103302 | 1.8 |
| BoLA-2:02201 |  | 0.095497 | 0.36 |
| BoLA-2:06001 |  | 0.093258 | 0.38 |
| BoLA-2:03001 |  | 0.092687 | 0.27 |
| BoLA-2:00501 |  | 0.090834 | 1.9 |
| BoLA-3:02701 |  | 0.090035 | 1.2 |
| BoLA-3:02702 |  | 0.090035 | 1.2 |
| BoLA-3:06601 |  | 0.089884 | 2.4 |
| BoLA-2:01601 |  | 0.087713 | 0.53 |
| BoLA-3:06602 |  | 0.086213 | 3.0 |
| BoLA-3:00401 |  | 0.083495 | 0.89 |
| BoLA-3:00402 |  | 0.083495 | 0.89 |
| BoLA-3:00403 |  | 0.083495 | 0.89 |
| BoLA-3:05301 |  | 0.083495 | 0.89 |
| BoLA-gb1.7 |  | 0.083495 | 0.89 |
| BoLA-1:04901 |  | 0.076971 | 2.4 |
| BoLA-1:02001 |  | 0.076438 | 1.6 |
| BoLA-3:05901 |  | 0.075289 | 1.1 |
| BoLA-3:06501 |  | 0.073736 | 6.6 |
| BoLA-5:06401 |  | 0.07274 | 0.38 |
| BoLA-T7 |  | 0.06933 | 2.2 |
| BoLA-3:01001 |  | 0.066655 | 0.97 |
| BoLA-3:05201 |  | 0.063606 | 2.0 |
| BoLA-3:03801 |  | 0.056775 | 1.9 |
| BoLA-2:01801 |  | 0.052919 | 2.4 |
| BoLA-2:01802 |  | 0.052919 | 2.4 |
| BoLA-5:00301 |  | 0.050999 | 7.9 |
| BoLA-3:01701 |  | 0.048625 | 2.3 |
| BoLA-5:07201 |  | 0.043329 | 2.4 |
| BoLA-5:03901 |  | 0.0382 | 2.2 |
| BoLA-6:01402 |  | 0.037358 | 4.0 |
| BoLA-2:05401 |  | 0.035859 | 0.21 |
| BoLA-3:07301 |  | 0.03505 | 2.5 |
| BoLA-2:02601 |  | 0.031421 | 4.2 |
| BoLA-2:02602 |  | 0.031421 | 4.2 |
| BoLA-2:02603 |  | 0.031421 | 4.2 |
| BoLA-1:02801 |  | 0.020799 | 2.5 |
| BoLA-1:02901 |  | 0.019193 | 4.6 |
| BoLA-3:00103 |  | 0.016977 | 2.2 |
| BoLA-3:00201 |  | 0.013816 | 11 |
| BoLA-JSP.1 |  | 0.013816 | 11 |
| BoLA-6:04001 |  | 0.0119 | 9.7 |
| BoLA-6:01401 |  | 0.010958 | 6.8 |
| BoLA-6:03401 |  | 0.009568 | 4.0 |
| BoLA-3:03501 |  | 0.008616 | 1.6 |
| BoLA-3:00101 |  | 0.007749 | 4.4 |
| BoLA-AW10 |  | 0.007749 | 4.4 |
| BoLA-1:01901 |  | 0.006399 | 6.0 |
| BoLA-1:02101 |  | 0.004945 | 1.8 |
| BoLA-6:01501 |  | 0.004807 | 6.3 |
| BoLA-3:00102 |  | 0.004719 | 5.4 |
| BoLA-1:07401 |  | 0.004653 | 6.6 |
| BoLA-6:01502 |  | 0.001096 | 11 |
| BoLA-6:04101 |  | 0.001012 | 14 |
| BoLA-T2b |  | 0.001012 | 14 |
| BoLA-6:01301 |  | 0.00094 | 19 |
| BoLA-HD6 |  | 0.00094 | 19 |
| BoLA-6:01302 |  | 0.000701 | 12 |
