# Supplementary material for: Development of a multi-epitope chimeric vaccine in silico against Babesia bovis, Theileria annulata, and Anaplasma marginale using computational biology tools and reverse vaccinology approach
Source: PLoS One. 2025 Jan 24;20(1):e0312262. doi: 10.1371/journal.pone.0312262 (PMC11759392; doi:10.1371/journal.pone.0312262)
Supplement: S3 File — (DOCX) [file pone.0312262.s009.docx]

The tables of all the ten MHC I epitopes of MSA-2c with their scores and percentile rank representing their affinities for different BOLA alleles. The peptide which has been selected for chimeric vaccine construction has been represented in bold letters. The BoLA alleles binding with the selected peptide possessing a percentile value >50 is highlighted as yellow. The BoLA allele that has bonded with the selected peptide with the lowest percentile rank is highlighted as green.

| Alleles | Peptide | Score | Percentile rank |
| --- | --- | --- | --- |
| BoLA-2:00501 | **YLSGQSNEE** | 0.012596 | 14 |
| BoLA-2:01602 |  | 0.007787 | 11 |
| BoLA-2:00601 |  | 0.007787 | 11 |
| BoLA-1:00901 |  | 0.005796 | 10 |
| BoLA-5:00301 |  | 0.005716 | 31 |
| BoLA-1:06701 |  | 0.005533 | 23 |
| BoLA-1:06101 |  | 0.003476 | 21 |
| BoLA-2:00602 |  | 0.003137 | 20 |
| BoLA-2:06201 |  | 0.00301 | 24 |
| BoLA-2:05701 |  | 0.002435 | 27 |
| BoLA-T5 |  | 0.002046 | 16 |
| BoLA-1:00902 |  | 0.002046 | 16 |
| BoLA-D18.4 |  | 0.001783 | 22 |
| BoLA-1:02301 |  | 0.001783 | 22 |
| BoLA-2:06001 |  | 0.001481 | 20 |
| BoLA-5:07201 |  | 0.001433 | 22 |
| BoLA-2:04601 |  | 0.001431 | 31 |
| BoLA-2:02501 |  | 0.001268 | 18 |
| BoLA-3:03701 |  | 0.001062 | 58 |
| BoLA-2:04401 |  | 0.00104 | 51 |
| BoLA-6:03401 |  | 0.000999 | 14 |
| BoLA-1:03101 |  | 0.00084 | 36 |
| BoLA-2:04501 |  | 0.000819 | 37 |
| BoLA-1:02001 |  | 0.000665 | 26 |
| BoLA-2:04701 |  | 0.000654 | 43 |
| BoLA-3:07301 |  | 0.000612 | 21 |
| BoLA-3:06602 |  | 0.000606 | 44 |
| BoLA-2:07001 |  | 0.000592 | 26 |
| BoLA-3:01703 |  | 0.00057 | 48 |
| BoLA-3:01702 |  | 0.000565 | 39 |
| BoLA-T7 |  | 0.000556 | 33 |
| BoLA-gb1.7 |  | 0.000552 | 33 |
| BoLA-3:05301 |  | 0.000552 | 33 |
| BoLA-3:00403 |  | 0.000552 | 33 |
| BoLA-3:00402 |  | 0.000552 | 33 |
| BoLA-3:00401 |  | 0.000552 | 33 |
| BoLA-1:03102 |  | 0.000548 | 41 |
| BoLA-2:01601 |  | 0.000546 | 28 |
| BoLA-JSP.1 |  | 0.000544 | 43 |
| BoLA-3:00201 |  | 0.000544 | 43 |
| BoLA-2:03001 |  | 0.00052 | 22 |
| BoLA-3:06601 |  | 0.000508 | 42 |
| BoLA-6:01402 |  | 0.000506 | 33 |
| BoLA-3:03601 |  | 0.000503 | 43 |
| BoLA-2:00802 |  | 0.00044 | 18 |
| BoLA-2:06901 |  | 0.000398 | 26 |
| BoLA-3:05801 |  | 0.000396 | 57 |
| BoLA-4:02402 |  | 0.000394 | 34 |
| BoLA-2:04301 |  | 0.00039 | 40 |
| BoLA-2:03202 |  | 0.000367 | 40 |
| BoLA-2:01802 |  | 0.000357 | 31 |
| BoLA-2:01801 |  | 0.000357 | 31 |
| BoLA-2:04402 |  | 0.000356 | 39 |
| BoLA-2:05501 |  | 0.00034 | 43 |
| BoLA-3:05001 |  | 0.000337 | 56 |
| BoLA-3:01701 |  | 0.000321 | 41 |
| BoLA-5:03901 |  | 0.000301 | 37 |
| BoLA-T2a |  | 0.000286 | 44 |
| BoLA-2:01201 |  | 0.000286 | 44 |
| BoLA-3:06501 |  | 0.00027 | 64 |
| BoLA-3:05101 |  | 0.000242 | 44 |
| BoLA-1:01901 |  | 0.000237 | 27 |
| BoLA-3:01001 |  | 0.000223 | 39 |
| BoLA-3:05901 |  | 0.000181 | 52 |
| BoLA-3:05201 |  | 0.000168 | 43 |
| BoLA-2:07101 |  | 0.000157 | 53 |
| BoLA-2:00801 |  | 0.000155 | 26 |
| BoLA-3:05002 |  | 0.000152 | 59 |
| BoLA-1:02801 |  | 0.00015 | 31 |
| BoLA-6:01401 |  | 0.000135 | 49 |
| BoLA-5:06401 |  | 0.000132 | 29 |
| BoLA-3:00103 |  | 0.000128 | 36 |
| BoLA-2:04801 |  | 0.000127 | 43 |
| BoLA-4:02401 |  | 0.000126 | 30 |
| BoLA-amani.1 |  | 0.000125 | 44 |
| BoLA-3:06801 |  | 0.00012 | 60 |
| BoLA-1:04201 |  | 0.000111 | 54 |
| BoLA-6:04001 |  | 0.000102 | 64 |
| BoLA-3:02702 |  | 0.000102 | 55 |
| BoLA-3:02701 |  | 0.000102 | 55 |
| BoLA-2:05601 |  | 9.60E-05 | 48 |
| BoLA-1:04901 |  | 8.60E-05 | 53 |
| BoLA-3:03801 |  | 8.40E-05 | 66 |
| BoLA-1:07401 |  | 8.30E-05 | 36 |
| BoLA-1:02101 |  | 6.00E-05 | 25 |
| BoLA-2:02603 |  | 4.90E-05 | 63 |
| BoLA-2:02602 |  | 4.90E-05 | 63 |
| BoLA-2:02601 |  | 4.90E-05 | 63 |
| BoLA-HD6 |  | 4.70E-05 | 44 |
| BoLA-6:01301 |  | 4.70E-05 | 44 |
| BoLA-AW10 |  | 4.40E-05 | 56 |
| BoLA-6:01502 |  | 4.40E-05 | 39 |
| BoLA-3:00102 |  | 4.40E-05 | 50 |
| BoLA-3:00101 |  | 4.40E-05 | 56 |
| BoLA-1:02901 |  | 4.30E-05 | 54 |
| BoLA-6:01501 |  | 4.10E-05 | 48 |
| BoLA-T2b |  | 3.70E-05 | 45 |
| BoLA-6:04101 |  | 3.70E-05 | 45 |
| BoLA-2:02201 |  | 2.70E-05 | 54 |
| BoLA-3:01101 |  | 2.50E-05 | 69 |
| BoLA-4:06301 |  | 2.40E-05 | 49 |
| BoLA-2:05401 |  | 1.80E-05 | 42 |
| BoLA-6:01302 |  | 1.40E-05 | 46 |
| BoLA-3:03501 |  | 8.00E-06 | 55 |

| Alleles | Peptide | Score | Percentile rank |
| --- | --- | --- | --- |
| BoLA-1:06701 | LSGQSNEEL | 0.223419 | 0.88 |
| BoLA-3:06501 |  | 0.154689 | 2.9 |
| BoLA-T2c |  | 0.145061 | 4.4 |
| BoLA-3:01101 |  | 0.130022 | 1.4 |
| BoLA-JSP.1 |  | 0.098461 | 2.2 |
| BoLA-3:00201 |  | 0.098461 | 2.2 |
| BoLA-5:00301 |  | 0.091712 | 4.2 |
| BoLA-3:06602 |  | 0.088449 | 2.9 |
| BoLA-3:06801 |  | 0.087713 | 2.6 |
| BoLA-5:07201 |  | 0.087498 | 0.92 |
| BoLA-3:01702 |  | 0.079793 | 2.1 |
| BoLA-3:03601 |  | 0.079341 | 2.3 |
| BoLA-3:05201 |  | 0.07671 | 1.6 |
| BoLA-3:05801 |  | 0.074633 | 3.1 |
| BoLA-3:01703 |  | 0.073672 | 3.5 |
| BoLA-5:03901 |  | 0.070977 | 0.93 |
| BoLA-3:06601 |  | 0.065323 | 3.3 |
| BoLA-3:07301 |  | 0.063112 | 1.5 |
| BoLA-2:05601 |  | 0.061747 | 0.77 |
| BoLA-2:04401 |  | 0.057214 | 6.8 |
| BoLA-4:06301 |  | 0.054015 | 1 |
| BoLA-3:03701 |  | 0.048712 | 7.8 |
| BoLA-3:03801 |  | 0.045539 | 2.7 |
| BoLA-6:04001 |  | 0.042195 | 3.8 |
| BoLA-1:06101 |  | 0.041644 | 4.1 |
| BoLA-2:00501 |  | 0.040997 | 4.8 |
| BoLA-T7 |  | 0.034474 | 4.6 |
| BoLA-2:07001 |  | 0.032927 | 2.2 |
| BoLA-2:04402 |  | 0.031225 | 3.6 |
| BoLA-2:05701 |  | 0.029825 | 7 |
| BoLA-gb1.7 |  | 0.026228 | 3.6 |
| BoLA-3:05301 |  | 0.026228 | 3.6 |
| BoLA-3:00403 |  | 0.026228 | 3.6 |
| BoLA-3:00402 |  | 0.026228 | 3.6 |
| BoLA-3:00401 |  | 0.026228 | 3.6 |
| BoLA-2:04501 |  | 0.020308 | 7.6 |
| BoLA-3:05002 |  | 0.020042 | 7.9 |
| BoLA-2:06201 |  | 0.01996 | 7.8 |
| BoLA-5:06401 |  | 0.019336 | 1.9 |
| BoLA-amani.1 |  | 0.018791 | 4.4 |
| BoLA-AW10 |  | 0.01536 | 2.3 |
| BoLA-3:00101 |  | 0.01536 | 2.3 |
| BoLA-2:04601 |  | 0.013554 | 9.4 |
| BoLA-3:05101 |  | 0.012896 | 6.4 |
| BoLA-3:00103 |  | 0.012531 | 3 |
| BoLA-2:04301 |  | 0.010923 | 7.8 |
| BoLA-3:01701 |  | 0.010821 | 7.8 |
| BoLA-3:05901 |  | 0.010435 | 8.1 |
| BoLA-2:06901 |  | 0.010335 | 5.2 |
| BoLA-2:03001 |  | 0.010248 | 4.2 |
| BoLA-2:04701 |  | 0.009173 | 14 |
| BoLA-3:00102 |  | 0.009002 | 3.1 |
| BoLA-3:02702 |  | 0.008019 | 8.7 |
| BoLA-3:02701 |  | 0.008019 | 8.7 |
| BoLA-2:04801 |  | 0.007932 | 7.9 |
| BoLA-2:05401 |  | 0.007762 | 1.6 |
| BoLA-2:01802 |  | 0.007344 | 8.2 |
| BoLA-2:01801 |  | 0.007344 | 8.2 |
| BoLA-2:07101 |  | 0.007199 | 12 |
| BoLA-6:01501 |  | 0.006948 | 4.9 |
| BoLA-1:02901 |  | 0.006361 | 9.1 |
| BoLA-1:07401 |  | 0.005845 | 5.7 |
| BoLA-1:04901 |  | 0.005359 | 14 |
| BoLA-1:02801 |  | 0.005357 | 6.5 |
| BoLA-HD6 |  | 0.005274 | 9.5 |
| BoLA-6:01301 |  | 0.005274 | 9.5 |
| BoLA-2:01602 |  | 0.005065 | 14 |
| BoLA-2:00601 |  | 0.005065 | 14 |
| BoLA-2:03202 |  | 0.00499 | 14 |
| BoLA-3:03501 |  | 0.004765 | 2.7 |
| BoLA-2:05501 |  | 0.004602 | 15 |
| BoLA-4:02402 |  | 0.004583 | 13 |
| BoLA-3:05001 |  | 0.004467 | 22 |
| BoLA-6:01502 |  | 0.003655 | 5.2 |
| BoLA-2:00602 |  | 0.003639 | 18 |
| BoLA-2:02603 |  | 0.003592 | 15 |
| BoLA-2:02602 |  | 0.003592 | 15 |
| BoLA-2:02601 |  | 0.003592 | 15 |
| BoLA-D18.4 |  | 0.003234 | 18 |
| BoLA-1:02301 |  | 0.003234 | 18 |
| BoLA-3:01001 |  | 0.002845 | 14 |
| BoLA-1:03101 |  | 0.002431 | 24 |
| BoLA-T2a |  | 0.002268 | 21 |
| BoLA-2:01201 |  | 0.002268 | 21 |
| BoLA-2:06001 |  | 0.002178 | 16 |
| BoLA-4:02401 |  | 0.002064 | 8.7 |
| BoLA-2:02501 |  | 0.002022 | 15 |
| BoLA-2:00802 |  | 0.001762 | 9 |
| BoLA-6:01402 |  | 0.001703 | 20 |
| BoLA-T5 |  | 0.001533 | 18 |
| BoLA-1:00902 |  | 0.001533 | 18 |
| BoLA-6:01401 |  | 0.001501 | 21 |
| BoLA-1:03102 |  | 0.001404 | 29 |
| BoLA-1:00901 |  | 0.00116 | 22 |
| BoLA-1:04201 |  | 0.001125 | 26 |
| BoLA-2:00801 |  | 0.001061 | 12 |
| BoLA-T2b |  | 0.000737 | 16 |
| BoLA-6:04101 |  | 0.000737 | 16 |
| BoLA-2:01601 |  | 0.000678 | 25 |
| BoLA-2:02201 |  | 0.000625 | 18 |
| BoLA-6:01302 |  | 0.000561 | 13 |
| BoLA-6:03401 |  | 0.000548 | 18 |
| BoLA-1:02001 |  | 0.000342 | 34 |
| BoLA-1:01901 |  | 0.000295 | 25 |
| BoLA-1:02101 |  | 0.000109 | 19 |

| Alleles | Peptide | Score | Percentile rank |
| --- | --- | --- | --- |
| BoLA-3:06501 | SGQSNEELL | 0.424075 | 0.25 |
| BoLA-3:01702 |  | 0.397857 | 0.1 |
| BoLA-6:04001 |  | 0.395831 | 0.13 |
| BoLA-3:01703 |  | 0.342042 | 0.27 |
| BoLA-JSP.1 |  | 0.311077 | 0.41 |
| BoLA-3:00201 |  | 0.311077 | 0.41 |
| BoLA-3:01701 |  | 0.220598 | 0.41 |
| BoLA-1:06701 |  | 0.216889 | 0.93 |
| BoLA-3:05801 |  | 0.196337 | 0.51 |
| BoLA-3:06801 |  | 0.176706 | 0.82 |
| BoLA-3:01101 |  | 0.17308 | 0.98 |
| BoLA-5:00301 |  | 0.166292 | 1.7 |
| BoLA-5:07201 |  | 0.134682 | 0.43 |
| BoLA-AW10 |  | 0.114542 | 0.13 |
| BoLA-3:00101 |  | 0.114542 | 0.13 |
| BoLA-3:06602 |  | 0.100947 | 2.4 |
| BoLA-3:03801 |  | 0.097567 | 0.75 |
| BoLA-3:05201 |  | 0.096813 | 1.2 |
| BoLA-3:03701 |  | 0.094536 | 3.6 |
| BoLA-3:00102 |  | 0.092398 | 0.12 |
| BoLA-T2c |  | 0.091964 | 6.1 |
| BoLA-5:03901 |  | 0.091726 | 0.57 |
| BoLA-3:06601 |  | 0.082698 | 2.6 |
| BoLA-2:00501 |  | 0.082525 | 2.1 |
| BoLA-3:00103 |  | 0.07306 | 0.3 |
| BoLA-3:05101 |  | 0.068812 | 1.2 |
| BoLA-3:03601 |  | 0.06857 | 2.7 |
| BoLA-4:06301 |  | 0.065945 | 0.78 |
| BoLA-gb1.7 |  | 0.063713 | 1.4 |
| BoLA-3:05301 |  | 0.063713 | 1.4 |
| BoLA-3:00403 |  | 0.063713 | 1.4 |
| BoLA-3:00402 |  | 0.063713 | 1.4 |
| BoLA-3:00401 |  | 0.063713 | 1.4 |
| BoLA-2:04401 |  | 0.063015 | 6.2 |
| BoLA-2:05601 |  | 0.058464 | 0.83 |
| BoLA-T7 |  | 0.057793 | 2.8 |
| BoLA-2:04701 |  | 0.054074 | 3.2 |
| BoLA-3:07301 |  | 0.051846 | 1.8 |
| BoLA-3:05901 |  | 0.051624 | 1.9 |
| BoLA-3:05002 |  | 0.047764 | 3.7 |
| BoLA-2:04601 |  | 0.045666 | 3.2 |
| BoLA-1:06101 |  | 0.034547 | 4.8 |
| BoLA-3:02702 |  | 0.033619 | 3.2 |
| BoLA-3:02701 |  | 0.033619 | 3.2 |
| BoLA-2:04301 |  | 0.032192 | 3.2 |
| BoLA-6:01501 |  | 0.030306 | 1.3 |
| BoLA-2:04402 |  | 0.028893 | 3.8 |
| BoLA-5:06401 |  | 0.028209 | 1.3 |
| BoLA-2:01602 |  | 0.024452 | 4 |
| BoLA-2:00601 |  | 0.024452 | 4 |
| BoLA-2:00602 |  | 0.02143 | 5.5 |
| BoLA-3:03501 |  | 0.019407 | 0.58 |
| BoLA-2:03001 |  | 0.018898 | 2.5 |
| BoLA-1:02801 |  | 0.018535 | 2.7 |
| BoLA-2:05401 |  | 0.01808 | 0.58 |
| BoLA-2:04801 |  | 0.016658 | 5 |
| BoLA-2:06901 |  | 0.01589 | 3.8 |
| BoLA-HD6 |  | 0.015861 | 5.8 |
| BoLA-6:01301 |  | 0.015861 | 5.8 |
| BoLA-6:01502 |  | 0.015447 | 1.7 |
| BoLA-2:05701 |  | 0.015331 | 11 |
| BoLA-2:07101 |  | 0.014934 | 7.4 |
| BoLA-2:02501 |  | 0.013368 | 4.4 |
| BoLA-1:02901 |  | 0.012864 | 6 |
| BoLA-1:07401 |  | 0.012737 | 3.4 |
| BoLA-2:07001 |  | 0.011375 | 5.5 |
| BoLA-2:06201 |  | 0.011076 | 12 |
| BoLA-1:04901 |  | 0.010957 | 9.4 |
| BoLA-2:06001 |  | 0.010659 | 5.8 |
| BoLA-2:02603 |  | 0.010386 | 8.2 |
| BoLA-2:02602 |  | 0.010386 | 8.2 |
| BoLA-2:02601 |  | 0.010386 | 8.2 |
| BoLA-3:01001 |  | 0.010362 | 6.5 |
| BoLA-3:05001 |  | 0.010226 | 14 |
| BoLA-2:04501 |  | 0.009786 | 13 |
| BoLA-2:01802 |  | 0.008258 | 7.7 |
| BoLA-2:01801 |  | 0.008258 | 7.7 |
| BoLA-D18.4 |  | 0.008156 | 12 |
| BoLA-1:02301 |  | 0.008156 | 12 |
| BoLA-6:01401 |  | 0.006526 | 9.4 |
| BoLA-6:01402 |  | 0.005809 | 11 |
| BoLA-6:03401 |  | 0.005434 | 5.7 |
| BoLA-2:01601 |  | 0.00538 | 8 |
| BoLA-T2a |  | 0.005096 | 15 |
| BoLA-2:01201 |  | 0.005096 | 15 |
| BoLA-1:03101 |  | 0.004768 | 18 |
| BoLA-T2b |  | 0.004703 | 6 |
| BoLA-6:04101 |  | 0.004703 | 6 |
| BoLA-amani.1 |  | 0.004621 | 9.8 |
| BoLA-2:03202 |  | 0.004529 | 14 |
| BoLA-6:01302 |  | 0.003718 | 5.2 |
| BoLA-2:05501 |  | 0.003489 | 18 |
| BoLA-4:02402 |  | 0.003317 | 15 |
| BoLA-1:03102 |  | 0.003313 | 20 |
| BoLA-T5 |  | 0.002196 | 16 |
| BoLA-1:00902 |  | 0.002196 | 16 |
| BoLA-1:04201 |  | 0.001675 | 22 |
| BoLA-2:00802 |  | 0.0014 | 11 |
| BoLA-1:00901 |  | 0.00132 | 21 |
| BoLA-1:02001 |  | 0.001111 | 21 |
| BoLA-1:01901 |  | 0.001042 | 14 |
| BoLA-2:02201 |  | 0.00085 | 16 |
| BoLA-1:02101 |  | 0.000395 | 9.5 |

| Alleles | Peptide | Scores | Percentile rank |
| --- | --- | --- | --- |
| BoLA-1:01901 | LEKNFEAVG | 0.046046 | 2.1 |
| BoLA-1:02001 |  | 0.026117 | 3.9 |
| BoLA-6:01402 |  | 0.017192 | 6.1 |
| BoLA-6:01401 |  | 0.016573 | 5.1 |
| BoLA-1:04201 |  | 0.003984 | 15 |
| BoLA-2:01602 |  | 0.003939 | 16 |
| BoLA-2:00601 |  | 0.003939 | 16 |
| BoLA-3:05001 |  | 0.003 | 26 |
| BoLA-3:03701 |  | 0.002903 | 44 |
| BoLA-2:00602 |  | 0.002623 | 21 |
| BoLA-1:03101 |  | 0.002248 | 25 |
| BoLA-3:05002 |  | 0.002202 | 26 |
| BoLA-3:05901 |  | 0.002165 | 20 |
| BoLA-3:02702 |  | 0.001884 | 19 |
| BoLA-3:02701 |  | 0.001884 | 19 |
| BoLA-2:02603 |  | 0.001784 | 20 |
| BoLA-2:02602 |  | 0.001784 | 20 |
| BoLA-2:02601 |  | 0.001784 | 20 |
| BoLA-2:01601 |  | 0.001243 | 19 |
| BoLA-6:03401 |  | 0.001214 | 13 |
| BoLA-1:04901 |  | 0.001163 | 25 |
| BoLA-D18.4 |  | 0.001149 | 26 |
| BoLA-1:02301 |  | 0.001149 | 26 |
| BoLA-1:03102 |  | 0.001051 | 33 |
| BoLA-2:06201 |  | 0.000791 | 40 |
| BoLA-2:04301 |  | 0.000711 | 33 |
| BoLA-2:05501 |  | 0.000676 | 35 |
| BoLA-2:06001 |  | 0.000605 | 29 |
| BoLA-2:00501 |  | 0.00059 | 53 |
| BoLA-gb1.7 |  | 0.000567 | 33 |
| BoLA-3:05301 |  | 0.000567 | 33 |
| BoLA-3:00403 |  | 0.000567 | 33 |
| BoLA-3:00402 |  | 0.000567 | 33 |
| BoLA-3:00401 |  | 0.000567 | 33 |
| BoLA-6:01501 |  | 0.000457 | 22 |
| BoLA-1:06101 |  | 0.000446 | 41 |
| BoLA-T5 |  | 0.000429 | 30 |
| BoLA-1:00902 |  | 0.000429 | 30 |
| BoLA-3:05801 |  | 0.000398 | 57 |
| BoLA-5:00301 |  | 0.000358 | 68 |
| BoLA-3:01703 |  | 0.000337 | 55 |
| BoLA-1:02901 |  | 0.0003 | 32 |
| BoLA-3:06501 |  | 0.000287 | 63 |
| BoLA-T2b |  | 0.000268 | 23 |
| BoLA-6:04101 |  | 0.000268 | 23 |
| BoLA-2:04701 |  | 0.000219 | 58 |
| BoLA-2:04401 |  | 0.000211 | 71 |
| BoLA-1:00901 |  | 0.00018 | 44 |
| BoLA-1:06701 |  | 0.000174 | 64 |
| BoLA-JSP.1 |  | 0.00017 | 61 |
| BoLA-3:00201 |  | 0.00017 | 61 |
| BoLA-2:05701 |  | 0.000163 | 60 |
| BoLA-3:03801 |  | 0.000153 | 58 |
| BoLA-2:01802 |  | 0.000143 | 42 |
| BoLA-2:01801 |  | 0.000143 | 42 |
| BoLA-1:02101 |  | 0.000141 | 17 |
| BoLA-2:02501 |  | 0.000121 | 45 |
| BoLA-6:01502 |  | 0.000113 | 28 |
| BoLA-3:06602 |  | 0.000108 | 67 |
| BoLA-5:07201 |  | 0.000107 | 48 |
| BoLA-2:04402 |  | 0.000106 | 55 |
| BoLA-3:01001 |  | 0.0001 | 49 |
| BoLA-1:02801 |  | 8.60E-05 | 37 |
| BoLA-3:01702 |  | 8.40E-05 | 64 |
| BoLA-1:07401 |  | 7.90E-05 | 36 |
| BoLA-3:06801 |  | 7.70E-05 | 65 |
| BoLA-2:04601 |  | 7.60E-05 | 68 |
| BoLA-3:06601 |  | 7.20E-05 | 68 |
| BoLA-3:01701 |  | 7.10E-05 | 64 |
| BoLA-2:02201 |  | 6.70E-05 | 41 |
| BoLA-2:00802 |  | 6.70E-05 | 37 |
| BoLA-HD6 |  | 5.40E-05 | 42 |
| BoLA-6:01301 |  | 5.40E-05 | 42 |
| BoLA-2:04501 |  | 5.40E-05 | 72 |
| BoLA-4:02402 |  | 4.70E-05 | 62 |
| BoLA-3:00102 |  | 4.70E-05 | 49 |
| BoLA-6:04001 |  | 4.60E-05 | 77 |
| BoLA-3:01101 |  | 4.50E-05 | 62 |
| BoLA-2:04801 |  | 4.50E-05 | 57 |
| BoLA-3:05101 |  | 4.20E-05 | 67 |
| BoLA-T2c |  | 3.60E-05 | 71 |
| BoLA-2:07101 |  | 3.60E-05 | 72 |
| BoLA-3:03501 |  | 3.50E-05 | 36 |
| BoLA-AW10 |  | 3.10E-05 | 61 |
| BoLA-3:00101 |  | 3.10E-05 | 61 |
| BoLA-2:05601 |  | 2.90E-05 | 63 |
| BoLA-3:05201 |  | 2.60E-05 | 66 |
| BoLA-T2a |  | 2.50E-05 | 78 |
| BoLA-2:01201 |  | 2.50E-05 | 78 |
| BoLA-3:03601 |  | 2.40E-05 | 79 |
| BoLA-2:03202 |  | 2.40E-05 | 75 |
| BoLA-2:03001 |  | 2.40E-05 | 58 |
| BoLA-5:03901 |  | 2.30E-05 | 71 |
| BoLA-2:05401 |  | 2.10E-05 | 40 |
| BoLA-T7 |  | 1.60E-05 | 75 |
| BoLA-4:02401 |  | 1.40E-05 | 60 |
| BoLA-3:00103 |  | 1.40E-05 | 68 |
| BoLA-2:06901 |  | 1.30E-05 | 67 |
| BoLA-2:00801 |  | 1.30E-05 | 59 |
| BoLA-6:01302 |  | 1.00E-05 | 50 |
| BoLA-amani.1 |  | 9.00E-06 | 79 |
| BoLA-3:07301 |  | 9.00E-06 | 64 |
| BoLA-5:06401 |  | 8.00E-06 | 62 |
| BoLA-2:07001 |  | 6.00E-06 | 80 |
| BoLA-4:06301 |  | 2.00E-06 | 80 |

| Alleles | Peptide | Score | Percentile rank |
| --- | --- | --- | --- |
| BoLA-6:01402 | NEELLKLLI | 0.623755 | 0.24 |
| BoLA-1:01901 |  | 0.444186 | 0.29 |
| BoLA-6:01401 |  | 0.344564 | 0.13 |
| BoLA-1:02901 |  | 0.30492 | 0.12 |
| BoLA-1:07401 |  | 0.262913 | 0.07 |
| BoLA-6:03401 |  | 0.262886 | 0.11 |
| BoLA-2:01601 |  | 0.171719 | 0.14 |
| BoLA-1:02001 |  | 0.169425 | 0.58 |
| BoLA-2:00602 |  | 0.139293 | 0.39 |
| BoLA-T2b |  | 0.122354 | 0.49 |
| BoLA-6:04101 |  | 0.122354 | 0.49 |
| BoLA-2:01602 |  | 0.097774 | 0.52 |
| BoLA-2:00601 |  | 0.097774 | 0.52 |
| BoLA-3:05002 |  | 0.067137 | 2.5 |
| BoLA-3:06501 |  | 0.065782 | 7.3 |
| BoLA-1:04201 |  | 0.062433 | 1.8 |
| BoLA-1:06701 |  | 0.061965 | 4.9 |
| BoLA-3:05001 |  | 0.057163 | 3.5 |
| BoLA-1:04901 |  | 0.054847 | 3.3 |
| BoLA-6:01501 |  | 0.053088 | 0.63 |
| BoLA-2:06001 |  | 0.041959 | 1.4 |
| BoLA-3:03701 |  | 0.041379 | 9.2 |
| BoLA-3:03801 |  | 0.037396 | 3.4 |
| BoLA-T2c |  | 0.035402 | 11 |
| BoLA-6:01502 |  | 0.034995 | 0.61 |
| BoLA-3:06801 |  | 0.033432 | 7.1 |
| BoLA-2:01802 |  | 0.031349 | 3.4 |
| BoLA-2:01801 |  | 0.031349 | 3.4 |
| BoLA-3:05801 |  | 0.031043 | 7.5 |
| BoLA-2:06201 |  | 0.029129 | 6 |
| BoLA-1:06101 |  | 0.026089 | 6.1 |
| BoLA-3:01703 |  | 0.025339 | 8.7 |
| BoLA-1:03101 |  | 0.025057 | 6.2 |
| BoLA-1:02101 |  | 0.024267 | 0.4 |
| BoLA-3:01702 |  | 0.022749 | 6.6 |
| BoLA-2:04301 |  | 0.021279 | 4.7 |
| BoLA-3:05901 |  | 0.020509 | 4.8 |
| BoLA-2:02603 |  | 0.018102 | 6 |
| BoLA-2:02602 |  | 0.018102 | 6 |
| BoLA-2:02601 |  | 0.018102 | 6 |
| BoLA-1:02801 |  | 0.017343 | 2.9 |
| BoLA-3:03601 |  | 0.015733 | 9.9 |
| BoLA-3:01101 |  | 0.015582 | 7.4 |
| BoLA-5:00301 |  | 0.015115 | 20 |
| BoLA-3:06602 |  | 0.014007 | 13 |
| BoLA-2:00501 |  | 0.013593 | 13 |
| BoLA-2:04801 |  | 0.01201 | 6.2 |
| BoLA-3:02702 |  | 0.01198 | 6.8 |
| BoLA-3:02701 |  | 0.01198 | 6.8 |
| BoLA-3:01001 |  | 0.011799 | 5.9 |
| BoLA-2:05601 |  | 0.009729 | 6.2 |
| BoLA-2:05401 |  | 0.008877 | 1.4 |
| BoLA-3:01701 |  | 0.008659 | 9.1 |
| BoLA-1:03102 |  | 0.008506 | 12 |
| BoLA-3:05201 |  | 0.008334 | 11 |
| BoLA-2:04401 |  | 0.00812 | 25 |
| BoLA-3:06601 |  | 0.008102 | 15 |
| BoLA-2:05501 |  | 0.00799 | 11 |
| BoLA-D18.4 |  | 0.007887 | 12 |
| BoLA-1:02301 |  | 0.007887 | 12 |
| BoLA-gb1.7 |  | 0.007541 | 9.5 |
| BoLA-3:05301 |  | 0.007541 | 9.5 |
| BoLA-3:00403 |  | 0.007541 | 9.5 |
| BoLA-3:00402 |  | 0.007541 | 9.5 |
| BoLA-3:00401 |  | 0.007541 | 9.5 |
| BoLA-2:02501 |  | 0.007518 | 6.8 |
| BoLA-2:05701 |  | 0.006225 | 18 |
| BoLA-2:02201 |  | 0.005648 | 5.6 |
| BoLA-4:06301 |  | 0.004972 | 7.7 |
| BoLA-3:05101 |  | 0.003521 | 15 |
| BoLA-2:04601 |  | 0.003463 | 21 |
| BoLA-5:07201 |  | 0.003348 | 16 |
| BoLA-2:04701 |  | 0.003214 | 24 |
| BoLA-2:03001 |  | 0.003146 | 9.3 |
| BoLA-2:04402 |  | 0.003049 | 17 |
| BoLA-JSP.1 |  | 0.002629 | 24 |
| BoLA-3:00201 |  | 0.002629 | 24 |
| BoLA-3:07301 |  | 0.002271 | 13 |
| BoLA-4:02402 |  | 0.002157 | 18 |
| BoLA-T5 |  | 0.002126 | 16 |
| BoLA-1:00902 |  | 0.002126 | 16 |
| BoLA-2:06901 |  | 0.002107 | 13 |
| BoLA-5:06401 |  | 0.001966 | 9.6 |
| BoLA-6:04001 |  | 0.001709 | 25 |
| BoLA-HD6 |  | 0.001502 | 16 |
| BoLA-6:01301 |  | 0.001502 | 16 |
| BoLA-6:01302 |  | 0.001392 | 8.6 |
| BoLA-6:01402 |  | 0.623755 | 0.24 |
| BoLA-1:01901 |  | 0.444186 | 0.29 |
| BoLA-6:01401 |  | 0.344564 | 0.13 |
| BoLA-1:02901 |  | 0.30492 | 0.12 |
| BoLA-1:07401 |  | 0.262913 | 0.07 |
| BoLA-6:03401 |  | 0.262886 | 0.11 |
| BoLA-2:01601 |  | 0.171719 | 0.14 |
| BoLA-1:02001 |  | 0.169425 | 0.58 |
| BoLA-2:00602 |  | 0.139293 | 0.39 |
| BoLA-T2b |  | 0.122354 | 0.49 |
| BoLA-6:04101 |  | 0.122354 | 0.49 |
| BoLA-2:01602 |  | 0.097774 | 0.52 |
| BoLA-2:00601 |  | 0.097774 | 0.52 |
| BoLA-3:05002 |  | 0.067137 | 2.5 |
| BoLA-3:06501 |  | 0.065782 | 7.3 |
| BoLA-1:04201 |  | 0.062433 | 1.8 |
| BoLA-1:06701 |  | 0.061965 | 4.9 |
| BoLA-3:05001 |  | 0.057163 | 3.5 |
| BoLA-1:04901 |  | 0.054847 | 3.3 |
| BoLA-6:01501 |  | 0.053088 | 0.63 |
| BoLA-2:06001 |  | 0.041959 | 1.4 |
| BoLA-3:03701 |  | 0.041379 | 9.2 |
| BoLA-3:03801 |  | 0.037396 | 3.4 |
| BoLA-T2c |  | 0.035402 | 11 |
| BoLA-6:01502 |  | 0.034995 | 0.61 |
| BoLA-3:06801 |  | 0.033432 | 7.1 |
| BoLA-2:01802 |  | 0.031349 | 3.4 |
| BoLA-2:01801 |  | 0.031349 | 3.4 |
| BoLA-3:05801 |  | 0.031043 | 7.5 |
| BoLA-2:06201 |  | 0.029129 | 6 |
| BoLA-1:06101 |  | 0.026089 | 6.1 |
| BoLA-3:01703 |  | 0.025339 | 8.7 |
| BoLA-1:03101 |  | 0.025057 | 6.2 |
| BoLA-1:02101 |  | 0.024267 | 0.4 |
| BoLA-3:01702 |  | 0.022749 | 6.6 |
| BoLA-2:04301 |  | 0.021279 | 4.7 |
| BoLA-3:05901 |  | 0.020509 | 4.8 |
| BoLA-2:02603 |  | 0.018102 | 6 |
| BoLA-2:02602 |  | 0.018102 | 6 |
| BoLA-2:02601 |  | 0.018102 | 6 |
| BoLA-1:02801 |  | 0.017343 | 2.9 |
| BoLA-3:03601 |  | 0.015733 | 9.9 |
| BoLA-3:01101 |  | 0.015582 | 7.4 |
| BoLA-5:00301 |  | 0.015115 | 20 |
| BoLA-3:06602 |  | 0.014007 | 13 |
| BoLA-2:00501 |  | 0.013593 | 13 |
| BoLA-2:04801 |  | 0.01201 | 6.2 |
| BoLA-3:02702 |  | 0.01198 | 6.8 |
| BoLA-3:02701 |  | 0.01198 | 6.8 |
| BoLA-3:01001 |  | 0.011799 | 5.9 |
| BoLA-2:05601 |  | 0.009729 | 6.2 |
| BoLA-2:05401 |  | 0.008877 | 1.4 |
| BoLA-3:01701 |  | 0.008659 | 9.1 |
| BoLA-1:03102 |  | 0.008506 | 12 |
| BoLA-3:05201 |  | 0.008334 | 11 |
| BoLA-2:04401 |  | 0.00812 | 25 |
| BoLA-3:06601 |  | 0.008102 | 15 |
| BoLA-2:05501 |  | 0.00799 | 11 |
| BoLA-D18.4 |  | 0.007887 | 12 |
| BoLA-1:02301 |  | 0.007887 | 12 |
| BoLA-gb1.7 |  | 0.007541 | 9.5 |
| BoLA-3:05301 |  | 0.007541 | 9.5 |
| BoLA-3:00403 |  | 0.007541 | 9.5 |
| BoLA-3:00402 |  | 0.007541 | 9.5 |
| BoLA-3:00401 |  | 0.007541 | 9.5 |
| BoLA-2:02501 |  | 0.007518 | 6.8 |
| BoLA-2:05701 |  | 0.006225 | 18 |
| BoLA-2:02201 |  | 0.005648 | 5.6 |
| BoLA-4:06301 |  | 0.004972 | 7.7 |
| BoLA-3:05101 |  | 0.003521 | 15 |
| BoLA-2:04601 |  | 0.003463 | 21 |
| BoLA-5:07201 |  | 0.003348 | 16 |
| BoLA-2:04701 |  | 0.003214 | 24 |
| BoLA-2:03001 |  | 0.003146 | 9.3 |
| BoLA-2:04402 |  | 0.003049 | 17 |
| BoLA-JSP.1 |  | 0.002629 | 24 |
| BoLA-3:00201 |  | 0.002629 | 24 |
| BoLA-3:07301 |  | 0.002271 | 13 |
| BoLA-4:02402 |  | 0.002157 | 18 |
| BoLA-T5 |  | 0.002126 | 16 |
| BoLA-1:00902 |  | 0.002126 | 16 |
| BoLA-2:06901 |  | 0.002107 | 13 |
| BoLA-5:06401 |  | 0.001966 | 9.6 |
| BoLA-6:04001 |  | 0.001709 | 25 |
| BoLA-HD6 |  | 0.001502 | 16 |
| BoLA-6:01301 |  | 0.001502 | 16 |
| BoLA-6:01302 |  | 0.001392 | 8.6 |
| BoLA-6:01402 |  | 0.623755 | 0.24 |
| BoLA-1:01901 |  | 0.444186 | 0.29 |
| BoLA-6:01401 |  | 0.344564 | 0.13 |
| BoLA-1:02901 |  | 0.30492 | 0.12 |
| BoLA-1:07401 |  | 0.262913 | 0.07 |
| BoLA-6:03401 |  | 0.262886 | 0.11 |
| BoLA-2:01601 |  | 0.171719 | 0.14 |
| BoLA-1:02001 |  | 0.169425 | 0.58 |
| BoLA-2:00602 |  | 0.139293 | 0.39 |
| BoLA-T2b |  | 0.122354 | 0.49 |
| BoLA-6:04101 |  | 0.122354 | 0.49 |
| BoLA-2:01602 |  | 0.097774 | 0.52 |
| BoLA-2:00601 |  | 0.097774 | 0.52 |
| BoLA-3:05002 |  | 0.067137 | 2.5 |
| BoLA-3:06501 |  | 0.065782 | 7.3 |
| BoLA-1:04201 |  | 0.062433 | 1.8 |
| BoLA-1:06701 |  | 0.061965 | 4.9 |
| BoLA-3:05001 |  | 0.057163 | 3.5 |
| BoLA-1:04901 |  | 0.054847 | 3.3 |
| BoLA-6:01501 |  | 0.053088 | 0.63 |
| BoLA-2:06001 |  | 0.041959 | 1.4 |
| BoLA-3:03701 |  | 0.041379 | 9.2 |
| BoLA-3:03801 |  | 0.037396 | 3.4 |
| BoLA-T2c |  | 0.035402 | 11 |
| BoLA-6:01502 |  | 0.034995 | 0.61 |
| BoLA-3:06801 |  | 0.033432 | 7.1 |
| BoLA-2:01802 |  | 0.031349 | 3.4 |
| BoLA-2:01801 |  | 0.031349 | 3.4 |
| BoLA-3:05801 |  | 0.031043 | 7.5 |
| BoLA-2:06201 |  | 0.029129 | 6 |
| BoLA-1:06101 |  | 0.026089 | 6.1 |
| BoLA-3:01703 |  | 0.025339 | 8.7 |
| BoLA-1:03101 |  | 0.025057 | 6.2 |
| BoLA-1:02101 |  | 0.024267 | 0.4 |
| BoLA-3:01702 |  | 0.022749 | 6.6 |
| BoLA-2:04301 |  | 0.021279 | 4.7 |
| BoLA-3:05901 |  | 0.020509 | 4.8 |
| BoLA-2:02603 |  | 0.018102 | 6 |
| BoLA-2:02602 |  | 0.018102 | 6 |
| BoLA-2:02601 |  | 0.018102 | 6 |
| BoLA-1:02801 |  | 0.017343 | 2.9 |
| BoLA-3:03601 |  | 0.015733 | 9.9 |
| BoLA-3:01101 |  | 0.015582 | 7.4 |
| BoLA-5:00301 |  | 0.015115 | 20 |
| BoLA-3:06602 |  | 0.014007 | 13 |
| BoLA-2:00501 |  | 0.013593 | 13 |
| BoLA-2:04801 |  | 0.01201 | 6.2 |
| BoLA-3:02702 |  | 0.01198 | 6.8 |
| BoLA-3:02701 |  | 0.01198 | 6.8 |
| BoLA-3:01001 |  | 0.011799 | 5.9 |
| BoLA-2:05601 |  | 0.009729 | 6.2 |
| BoLA-2:05401 |  | 0.008877 | 1.4 |
| BoLA-3:01701 |  | 0.008659 | 9.1 |
| BoLA-1:03102 |  | 0.008506 | 12 |
| BoLA-3:05201 |  | 0.008334 | 11 |
| BoLA-2:04401 |  | 0.00812 | 25 |
| BoLA-3:06601 |  | 0.008102 | 15 |
| BoLA-2:05501 |  | 0.00799 | 11 |
| BoLA-D18.4 |  | 0.007887 | 12 |
| BoLA-1:02301 |  | 0.007887 | 12 |
| BoLA-gb1.7 |  | 0.007541 | 9.5 |
| BoLA-3:05301 |  | 0.007541 | 9.5 |
| BoLA-3:00403 |  | 0.007541 | 9.5 |
| BoLA-3:00402 |  | 0.007541 | 9.5 |
| BoLA-3:00401 |  | 0.007541 | 9.5 |
| BoLA-2:02501 |  | 0.007518 | 6.8 |
| BoLA-2:05701 |  | 0.006225 | 18 |
| BoLA-2:02201 |  | 0.005648 | 5.6 |
| BoLA-4:06301 |  | 0.004972 | 7.7 |
| BoLA-3:05101 |  | 0.003521 | 15 |
| BoLA-2:04601 |  | 0.003463 | 21 |
| BoLA-5:07201 |  | 0.003348 | 16 |
| BoLA-2:04701 |  | 0.003214 | 24 |
| BoLA-2:03001 |  | 0.003146 | 9.3 |
| BoLA-2:04402 |  | 0.003049 | 17 |
| BoLA-JSP.1 |  | 0.002629 | 24 |
| BoLA-3:00201 |  | 0.002629 | 24 |
| BoLA-3:07301 |  | 0.002271 | 13 |
| BoLA-4:02402 |  | 0.002157 | 18 |
| BoLA-T5 |  | 0.002126 | 16 |
| BoLA-1:00902 |  | 0.002126 | 16 |
| BoLA-2:06901 |  | 0.002107 | 13 |
| BoLA-5:06401 |  | 0.001966 | 9.6 |
| BoLA-6:04001 |  | 0.001709 | 25 |
| BoLA-HD6 |  | 0.001502 | 16 |
| BoLA-6:01301 |  | 0.001502 | 16 |
| BoLA-6:01302 |  | 0.001392 | 8.6 |

| Alleles | Peptide | Score | Percentile rank |
| --- | --- | --- | --- |
| BoLA-3:01703 | TSEAETPSP | 0.048262 | 5.2 |
| BoLA-1:06701 |  | 0.033848 | 8.2 |
| BoLA-3:01702 |  | 0.032583 | 4.9 |
| BoLA-1:06101 |  | 0.01596 | 8.8 |
| BoLA-6:01402 |  | 0.013093 | 7.1 |
| BoLA-3:01701 |  | 0.011216 | 7.7 |
| BoLA-3:05002 |  | 0.010911 | 12 |
| BoLA-3:03701 |  | 0.010389 | 26 |
| BoLA-2:01602 |  | 0.01027 | 8.6 |
| BoLA-2:00601 |  | 0.01027 | 8.6 |
| BoLA-2:07001 |  | 0.009972 | 6.1 |
| BoLA-2:05601 |  | 0.009545 | 6.3 |
| BoLA-3:06801 |  | 0.007739 | 17 |
| BoLA-2:00501 |  | 0.006374 | 21 |
| BoLA-3:01101 |  | 0.005391 | 13 |
| BoLA-3:05001 |  | 0.004703 | 21 |
| BoLA-2:01601 |  | 0.004269 | 9.3 |
| BoLA-5:00301 |  | 0.004188 | 35 |
| BoLA-2:00602 |  | 0.003676 | 18 |
| BoLA-T2c |  | 0.003524 | 27 |
| BoLA-2:04401 |  | 0.00314 | 36 |
| BoLA-3:05801 |  | 0.002754 | 31 |
| BoLA-gb1.7 |  | 0.002602 | 17 |
| BoLA-3:05301 |  | 0.002602 | 17 |
| BoLA-3:00403 |  | 0.002602 | 17 |
| BoLA-3:00402 |  | 0.002602 | 17 |
| BoLA-3:00401 |  | 0.002602 | 17 |
| BoLA-3:05901 |  | 0.0025 | 19 |
| BoLA-6:01401 |  | 0.002206 | 17 |
| BoLA-3:06501 |  | 0.002031 | 39 |
| BoLA-2:05401 |  | 0.001763 | 5.1 |
| BoLA-5:07201 |  | 0.001507 | 21 |
| BoLA-2:04402 |  | 0.001348 | 25 |
| BoLA-2:04501 |  | 0.001235 | 32 |
| BoLA-2:06201 |  | 0.001193 | 34 |
| BoLA-3:06602 |  | 0.00107 | 37 |
| BoLA-2:06001 |  | 0.000935 | 24 |
| BoLA-2:02501 |  | 0.000917 | 21 |
| BoLA-2:01802 |  | 0.000877 | 22 |
| BoLA-2:01801 |  | 0.000877 | 22 |
| BoLA-3:03801 |  | 0.000844 | 37 |
| BoLA-3:02702 |  | 0.00081 | 27 |
| BoLA-3:02701 |  | 0.00081 | 27 |
| BoLA-1:01901 |  | 0.000766 | 16 |
| BoLA-2:02603 |  | 0.000741 | 28 |
| BoLA-2:02602 |  | 0.000741 | 28 |
| BoLA-2:02601 |  | 0.000741 | 28 |
| BoLA-2:05701 |  | 0.000726 | 40 |
| BoLA-4:06301 |  | 0.000723 | 18 |
| BoLA-3:05201 |  | 0.00072 | 28 |
| BoLA-5:03901 |  | 0.000624 | 29 |
| BoLA-2:04301 |  | 0.000615 | 35 |
| BoLA-6:03401 |  | 0.000592 | 17 |
| BoLA-T2a |  | 0.000581 | 35 |
| BoLA-2:01201 |  | 0.000581 | 35 |
| BoLA-2:03202 |  | 0.000577 | 34 |
| BoLA-3:06601 |  | 0.000546 | 41 |
| BoLA-2:07101 |  | 0.000484 | 38 |
| BoLA-JSP.1 |  | 0.00045 | 46 |
| BoLA-3:00201 |  | 0.00045 | 46 |
| BoLA-3:03501 |  | 0.000449 | 13 |
| BoLA-3:07301 |  | 0.000424 | 24 |
| BoLA-2:04701 |  | 0.000422 | 49 |
| BoLA-5:06401 |  | 0.000401 | 20 |
| BoLA-amani.1 |  | 0.000398 | 30 |
| BoLA-2:03001 |  | 0.000398 | 24 |
| BoLA-2:04801 |  | 0.000394 | 29 |
| BoLA-3:05101 |  | 0.000392 | 37 |
| BoLA-1:02901 |  | 0.000386 | 29 |
| BoLA-3:00103 |  | 0.000351 | 25 |
| BoLA-AW10 |  | 0.000341 | 28 |
| BoLA-3:00101 |  | 0.000341 | 28 |
| BoLA-1:04201 |  | 0.000338 | 39 |
| BoLA-1:00901 |  | 0.000331 | 36 |
| BoLA-2:04601 |  | 0.000314 | 49 |
| BoLA-3:00102 |  | 0.000262 | 27 |
| BoLA-1:07401 |  | 0.000255 | 25 |
| BoLA-2:06901 |  | 0.000233 | 31 |
| BoLA-T7 |  | 0.000217 | 43 |
| BoLA-1:02001 |  | 0.000206 | 40 |
| BoLA-T5 |  | 0.000191 | 39 |
| BoLA-1:00902 |  | 0.000191 | 39 |
| BoLA-2:05501 |  | 0.00019 | 51 |
| BoLA-D18.4 |  | 0.000187 | 46 |
| BoLA-1:02301 |  | 0.000187 | 46 |
| BoLA-3:01001 |  | 0.000167 | 42 |
| BoLA-6:04001 |  | 0.000166 | 56 |
| BoLA-1:04901 |  | 0.000146 | 47 |
| BoLA-1:03101 |  | 0.000128 | 62 |
| BoLA-4:02402 |  | 0.000126 | 49 |
| BoLA-1:03102 |  | 0.000125 | 62 |
| BoLA-1:02101 |  | 0.000119 | 18 |
| BoLA-2:00802 |  | 8.70E-05 | 33 |
| BoLA-2:00801 |  | 8.40E-05 | 33 |
| BoLA-6:01501 |  | 7.10E-05 | 41 |
| BoLA-6:01502 |  | 5.70E-05 | 36 |
| BoLA-1:02801 |  | 5.70E-05 | 41 |
| BoLA-4:02401 |  | 3.50E-05 | 46 |
| BoLA-T2b |  | 2.90E-05 | 48 |
| BoLA-6:04101 |  | 2.90E-05 | 48 |
| BoLA-HD6 |  | 1.30E-05 | 59 |
| BoLA-6:01301 |  | 1.30E-05 | 59 |
| BoLA-2:02201 |  | 8.00E-06 | 72 |
| BoLA-6:01302 |  | 7.00E-06 | 55 |

| Alleles | Peptide | Score | Percentile rank |
| --- | --- | --- | --- |
| BoLA-3:03701 | STSEAETPS | 0.015359 | 21 |
| BoLA-1:06701 |  | 0.012661 | 16 |
| BoLA-2:04401 |  | 0.01256 | 20 |
| BoLA-3:01703 |  | 0.012114 | 14 |
| BoLA-5:00301 |  | 0.012095 | 22 |
| BoLA-3:06801 |  | 0.011656 | 14 |
| BoLA-3:03601 |  | 0.011579 | 12 |
| BoLA-2:04501 |  | 0.007072 | 15 |
| BoLA-2:07001 |  | 0.006537 | 8.1 |
| BoLA-T2c |  | 0.005347 | 24 |
| BoLA-2:00501 |  | 0.004648 | 25 |
| BoLA-3:06602 |  | 0.004234 | 23 |
| BoLA-T2a |  | 0.004177 | 16 |
| BoLA-2:01201 |  | 0.004177 | 16 |
| BoLA-3:01702 |  | 0.004107 | 18 |
| BoLA-2:03202 |  | 0.003898 | 15 |
| BoLA-1:06101 |  | 0.003651 | 20 |
| BoLA-3:05001 |  | 0.003079 | 26 |
| BoLA-3:05002 |  | 0.002846 | 23 |
| BoLA-5:07201 |  | 0.002823 | 17 |
| BoLA-3:06501 |  | 0.00249 | 37 |
| BoLA-3:06601 |  | 0.002426 | 25 |
| BoLA-3:01701 |  | 0.002288 | 19 |
| BoLA-2:06201 |  | 0.001887 | 29 |
| BoLA-3:05801 |  | 0.001786 | 36 |
| BoLA-2:04701 |  | 0.001658 | 32 |
| BoLA-2:05601 |  | 0.001588 | 18 |
| BoLA-3:05201 |  | 0.00148 | 22 |
| BoLA-2:07101 |  | 0.001249 | 28 |
| BoLA-amani.1 |  | 0.001196 | 19 |
| BoLA-2:05701 |  | 0.001131 | 35 |
| BoLA-2:04402 |  | 0.001101 | 27 |
| BoLA-T7 |  | 0.001064 | 27 |
| BoLA-5:03901 |  | 0.001028 | 24 |
| BoLA-2:04601 |  | 0.001022 | 35 |
| BoLA-gb1.7 |  | 0.001005 | 26 |
| BoLA-3:05301 |  | 0.001005 | 26 |
| BoLA-3:00403 |  | 0.001005 | 26 |
| BoLA-3:00402 |  | 0.001005 | 26 |
| BoLA-3:00401 |  | 0.001005 | 26 |
| BoLA-2:01602 |  | 0.000977 | 30 |
| BoLA-2:00601 |  | 0.000977 | 30 |
| BoLA-3:01101 |  | 0.000835 | 27 |
| BoLA-1:00901 |  | 0.000747 | 27 |
| BoLA-JSP.1 |  | 0.000707 | 39 |
| BoLA-3:00201 |  | 0.000707 | 39 |
| BoLA-6:01401 |  | 0.000663 | 29 |
| BoLA-6:01402 |  | 0.000626 | 31 |
| BoLA-2:00602 |  | 0.000618 | 38 |
| BoLA-2:02501 |  | 0.000596 | 25 |
| BoLA-2:06001 |  | 0.000504 | 31 |
| BoLA-2:06901 |  | 0.000471 | 24 |
| BoLA-2:03001 |  | 0.000414 | 23 |
| BoLA-2:05501 |  | 0.000393 | 41 |
| BoLA-T5 |  | 0.000376 | 32 |
| BoLA-1:00902 |  | 0.000376 | 32 |
| BoLA-1:03101 |  | 0.000366 | 47 |
| BoLA-1:03102 |  | 0.000359 | 47 |
| BoLA-3:03801 |  | 0.000349 | 47 |
| BoLA-3:05901 |  | 0.00033 | 43 |
| BoLA-3:01001 |  | 0.000329 | 34 |
| BoLA-D18.4 |  | 0.000312 | 40 |
| BoLA-1:02301 |  | 0.000312 | 40 |
| BoLA-4:06301 |  | 0.000304 | 24 |
| BoLA-3:07301 |  | 0.000287 | 27 |
| BoLA-3:05101 |  | 0.00027 | 42 |
| BoLA-1:04201 |  | 0.00023 | 44 |
| BoLA-3:02702 |  | 0.000228 | 43 |
| BoLA-3:02701 |  | 0.000228 | 43 |
| BoLA-2:04301 |  | 0.000224 | 48 |
| BoLA-AW10 |  | 0.000216 | 33 |
| BoLA-3:00101 |  | 0.000216 | 33 |
| BoLA-2:01601 |  | 0.000202 | 40 |
| BoLA-2:01802 |  | 0.000196 | 38 |
| BoLA-2:01801 |  | 0.000196 | 38 |
| BoLA-2:02603 |  | 0.000193 | 44 |
| BoLA-2:02602 |  | 0.000193 | 44 |
| BoLA-2:02601 |  | 0.000193 | 44 |
| BoLA-3:00103 |  | 0.000176 | 33 |
| BoLA-2:00801 |  | 0.000172 | 25 |
| BoLA-1:01901 |  | 0.000165 | 31 |
| BoLA-1:02001 |  | 0.000158 | 43 |
| BoLA-3:00102 |  | 0.000125 | 36 |
| BoLA-1:02101 |  | 0.000105 | 19 |
| BoLA-6:04001 |  | 0.000103 | 64 |
| BoLA-5:06401 |  | 9.70E-05 | 32 |
| BoLA-1:02901 |  | 9.60E-05 | 44 |
| BoLA-1:04901 |  | 8.90E-05 | 53 |
| BoLA-2:05401 |  | 8.60E-05 | 24 |
| BoLA-1:02801 |  | 8.10E-05 | 37 |
| BoLA-2:00802 |  | 7.60E-05 | 35 |
| BoLA-4:02402 |  | 6.70E-05 | 57 |
| BoLA-6:03401 |  | 6.40E-05 | 38 |
| BoLA-6:01501 |  | 5.90E-05 | 43 |
| BoLA-2:04801 |  | 4.40E-05 | 57 |
| BoLA-6:01502 |  | 3.70E-05 | 41 |
| BoLA-3:03501 |  | 3.60E-05 | 35 |
| BoLA-T2b |  | 3.10E-05 | 47 |
| BoLA-6:04101 |  | 3.10E-05 | 47 |
| BoLA-HD6 |  | 2.70E-05 | 50 |
| BoLA-6:01301 |  | 2.70E-05 | 50 |
| BoLA-1:07401 |  | 2.50E-05 | 50 |
| BoLA-4:02401 |  | 1.80E-05 | 56 |
| BoLA-6:01302 |  | 1.10E-05 | 49 |
| BoLA-2:02201 |  | 6.00E-06 | 76 |

| Alleles | Peptide | Score | Percentile rank |
| --- | --- | --- | --- |
| BoLA-2:01802 | NPSSTSEAE | 0.011812 | 6.3 |
| BoLA-2:01801 |  | 0.011812 | 6.3 |
| BoLA-2:00501 |  | 0.004889 | 24 |
| BoLA-2:01602 |  | 0.003818 | 16 |
| BoLA-2:00601 |  | 0.003818 | 16 |
| BoLA-3:03701 |  | 0.003669 | 41 |
| BoLA-2:00602 |  | 0.00304 | 20 |
| BoLA-2:03001 |  | 0.002593 | 11 |
| BoLA-3:01001 |  | 0.001297 | 20 |
| BoLA-3:05001 |  | 0.001267 | 37 |
| BoLA-T2c |  | 0.001222 | 35 |
| BoLA-1:06701 |  | 0.001203 | 40 |
| BoLA-3:05002 |  | 0.001018 | 34 |
| BoLA-1:02001 |  | 0.001005 | 22 |
| BoLA-2:01601 |  | 0.001001 | 21 |
| BoLA-3:01703 |  | 0.00099 | 40 |
| BoLA-6:01402 |  | 0.000785 | 28 |
| BoLA-3:01702 |  | 0.000671 | 36 |
| BoLA-3:01701 |  | 0.000611 | 33 |
| BoLA-3:03801 |  | 0.000606 | 41 |
| BoLA-2:02501 |  | 0.000588 | 25 |
| BoLA-2:06001 |  | 0.000522 | 31 |
| BoLA-3:05901 |  | 0.000422 | 40 |
| BoLA-5:00301 |  | 0.000352 | 68 |
| BoLA-2:04801 |  | 0.000346 | 31 |
| BoLA-1:03102 |  | 0.000343 | 48 |
| BoLA-1:01901 |  | 0.000323 | 24 |
| BoLA-1:06101 |  | 0.000313 | 45 |
| BoLA-gb1.7 |  | 0.00031 | 40 |
| BoLA-3:05301 |  | 0.00031 | 40 |
| BoLA-3:00403 |  | 0.00031 | 40 |
| BoLA-3:00402 |  | 0.00031 | 40 |
| BoLA-3:00401 |  | 0.00031 | 40 |
| BoLA-2:06201 |  | 0.000308 | 52 |
| BoLA-2:04701 |  | 0.000293 | 54 |
| BoLA-6:01401 |  | 0.000259 | 40 |
| BoLA-1:00901 |  | 0.000226 | 41 |
| BoLA-2:07101 |  | 0.000215 | 49 |
| BoLA-3:05801 |  | 0.000209 | 66 |
| BoLA-T2a |  | 0.000193 | 50 |
| BoLA-2:01201 |  | 0.000193 | 50 |
| BoLA-2:04301 |  | 0.000186 | 51 |
| BoLA-3:07301 |  | 0.000172 | 31 |
| BoLA-1:03101 |  | 0.000165 | 59 |
| BoLA-2:04401 |  | 0.000162 | 74 |
| BoLA-2:05701 |  | 0.000158 | 60 |
| BoLA-D18.4 |  | 0.000157 | 48 |
| BoLA-1:02301 |  | 0.000157 | 48 |
| BoLA-5:07201 |  | 0.000155 | 43 |
| BoLA-2:05601 |  | 0.000151 | 43 |
| BoLA-3:02702 |  | 0.000143 | 50 |
| BoLA-3:02701 |  | 0.000143 | 50 |
| BoLA-6:03401 |  | 0.000135 | 30 |
| BoLA-2:04601 |  | 0.000125 | 62 |
| BoLA-2:04501 |  | 0.000114 | 62 |
| BoLA-T2b |  | 0.000106 | 32 |
| BoLA-6:04101 |  | 0.000106 | 32 |
| BoLA-2:05501 |  | 9.50E-05 | 60 |
| BoLA-3:06801 |  | 9.00E-05 | 64 |
| BoLA-3:03601 |  | 8.80E-05 | 65 |
| BoLA-3:06501 |  | 8.70E-05 | 77 |
| BoLA-2:06901 |  | 8.70E-05 | 43 |
| BoLA-T5 |  | 8.60E-05 | 49 |
| BoLA-JSP.1 |  | 8.60E-05 | 72 |
| BoLA-3:00201 |  | 8.60E-05 | 72 |
| BoLA-1:00902 |  | 8.60E-05 | 49 |
| BoLA-3:05101 |  | 8.30E-05 | 58 |
| BoLA-6:01501 |  | 8.10E-05 | 39 |
| BoLA-1:07401 |  | 7.90E-05 | 36 |
| BoLA-2:03202 |  | 7.10E-05 | 61 |
| BoLA-1:02901 |  | 7.00E-05 | 48 |
| BoLA-4:02402 |  | 6.70E-05 | 57 |
| BoLA-1:04201 |  | 6.30E-05 | 62 |
| BoLA-6:04001 |  | 6.00E-05 | 73 |
| BoLA-1:02801 |  | 6.00E-05 | 41 |
| BoLA-3:00103 |  | 5.90E-05 | 47 |
| BoLA-1:04901 |  | 5.60E-05 | 59 |
| BoLA-2:02201 |  | 5.40E-05 | 44 |
| BoLA-2:07001 |  | 5.30E-05 | 53 |
| BoLA-2:04402 |  | 5.30E-05 | 64 |
| BoLA-3:06602 |  | 4.70E-05 | 77 |
| BoLA-2:02603 |  | 4.00E-05 | 66 |
| BoLA-2:02602 |  | 4.00E-05 | 66 |
| BoLA-2:02601 |  | 4.00E-05 | 66 |
| BoLA-3:01101 |  | 3.80E-05 | 64 |
| BoLA-AW10 |  | 3.50E-05 | 59 |
| BoLA-3:00101 |  | 3.50E-05 | 59 |
| BoLA-4:06301 |  | 3.00E-05 | 47 |
| BoLA-T7 |  | 2.90E-05 | 68 |
| BoLA-4:02401 |  | 2.50E-05 | 51 |
| BoLA-5:03901 |  | 2.40E-05 | 70 |
| BoLA-3:06601 |  | 2.20E-05 | 81 |
| BoLA-amani.1 |  | 2.10E-05 | 68 |
| BoLA-3:00102 |  | 1.80E-05 | 64 |
| BoLA-1:02101 |  | 1.50E-05 | 42 |
| BoLA-5:06401 |  | 1.40E-05 | 54 |
| BoLA-3:05201 |  | 1.20E-05 | 75 |
| BoLA-6:01502 |  | 1.10E-05 | 57 |
| BoLA-2:00802 |  | 1.10E-05 | 63 |
| BoLA-6:01302 |  | 7.00E-06 | 55 |
| BoLA-3:03501 |  | 7.00E-06 | 57 |
| BoLA-HD6 |  | 4.00E-06 | 74 |
| BoLA-6:01301 |  | 4.00E-06 | 74 |
| BoLA-2:00801 |  | 4.00E-06 | 76 |
| BoLA-2:05401 |  | 1.00E-06 | 85 |

| Alleles | Peptide | Score | Percentile rank |
| --- | --- | --- | --- |
| BoLA-3:01703 | PSSTSEAET | 0.00432 | 23 |
| BoLA-3:01702 |  | 0.002771 | 21 |
| BoLA-1:06701 |  | 0.002065 | 33 |
| BoLA-3:03701 |  | 0.001804 | 51 |
| BoLA-3:01701 |  | 0.001271 | 24 |
| BoLA-3:05002 |  | 0.000759 | 38 |
| BoLA-2:04401 |  | 0.000595 | 58 |
| BoLA-2:00501 |  | 0.000585 | 53 |
| BoLA-3:05001 |  | 0.000489 | 50 |
| BoLA-5:00301 |  | 0.00046 | 64 |
| BoLA-3:01101 |  | 0.000449 | 33 |
| BoLA-3:03601 |  | 0.000417 | 45 |
| BoLA-gb1.7 |  | 0.000399 | 37 |
| BoLA-3:05301 |  | 0.000399 | 37 |
| BoLA-3:00403 |  | 0.000399 | 37 |
| BoLA-3:00402 |  | 0.000399 | 37 |
| BoLA-3:00401 |  | 0.000399 | 37 |
| BoLA-2:05601 |  | 0.000295 | 35 |
| BoLA-2:04501 |  | 0.00027 | 51 |
| BoLA-3:06801 |  | 0.000238 | 52 |
| BoLA-3:05801 |  | 0.00023 | 64 |
| BoLA-2:00602 |  | 0.00023 | 52 |
| BoLA-3:06602 |  | 0.000189 | 60 |
| BoLA-2:04402 |  | 0.000184 | 48 |
| BoLA-1:06101 |  | 0.000154 | 54 |
| BoLA-2:04701 |  | 0.000147 | 63 |
| BoLA-3:06501 |  | 0.000145 | 71 |
| BoLA-JSP.1 |  | 0.000141 | 64 |
| BoLA-3:00201 |  | 0.000141 | 64 |
| BoLA-3:05901 |  | 0.000134 | 57 |
| BoLA-2:07001 |  | 0.000121 | 43 |
| BoLA-3:06601 |  | 0.000111 | 62 |
| BoLA-2:06201 |  | 0.000109 | 67 |
| BoLA-2:01602 |  | 0.000103 | 60 |
| BoLA-2:00601 |  | 0.000103 | 60 |
| BoLA-3:02702 |  | 0.0001 | 56 |
| BoLA-3:02701 |  | 0.0001 | 56 |
| BoLA-T7 |  | 9.70E-05 | 52 |
| BoLA-3:05201 |  | 9.20E-05 | 51 |
| BoLA-2:03202 |  | 9.20E-05 | 58 |
| BoLA-2:04301 |  | 8.20E-05 | 62 |
| BoLA-6:01402 |  | 7.30E-05 | 62 |
| BoLA-T2a |  | 7.00E-05 | 65 |
| BoLA-2:01201 |  | 7.00E-05 | 65 |
| BoLA-amani.1 |  | 6.90E-05 | 52 |
| BoLA-2:07101 |  | 6.40E-05 | 65 |
| BoLA-2:04601 |  | 5.80E-05 | 71 |
| BoLA-3:03801 |  | 5.70E-05 | 71 |
| BoLA-6:04001 |  | 5.20E-05 | 75 |
| BoLA-3:05101 |  | 4.60E-05 | 66 |
| BoLA-2:05701 |  | 4.50E-05 | 75 |
| BoLA-2:03001 |  | 3.70E-05 | 52 |
| BoLA-4:06301 |  | 3.60E-05 | 45 |
| BoLA-6:01401 |  | 3.50E-05 | 67 |
| BoLA-5:07201 |  | 3.20E-05 | 64 |
| BoLA-2:02501 |  | 3.20E-05 | 65 |
| BoLA-3:00103 |  | 3.10E-05 | 56 |
| BoLA-AW10 |  | 2.90E-05 | 62 |
| BoLA-3:00101 |  | 2.90E-05 | 62 |
| BoLA-2:01601 |  | 2.90E-05 | 69 |
| BoLA-2:01802 |  | 2.80E-05 | 66 |
| BoLA-2:01801 |  | 2.80E-05 | 66 |
| BoLA-2:02603 |  | 2.70E-05 | 71 |
| BoLA-2:02602 |  | 2.70E-05 | 71 |
| BoLA-2:02601 |  | 2.70E-05 | 71 |
| BoLA-5:03901 |  | 2.60E-05 | 69 |
| BoLA-2:06001 |  | 2.30E-05 | 76 |
| BoLA-1:04901 |  | 2.20E-05 | 71 |
| BoLA-1:03102 |  | 1.80E-05 | 87 |
| BoLA-3:00102 |  | 1.70E-05 | 65 |
| BoLA-1:07401 |  | 1.60E-05 | 56 |
| BoLA-1:04201 |  | 1.60E-05 | 79 |
| BoLA-2:04801 |  | 1.50E-05 | 72 |
| BoLA-1:03101 |  | 1.50E-05 | 87 |
| BoLA-5:06401 |  | 1.40E-05 | 54 |
| BoLA-T2c |  | 1.30E-05 | 81 |
| BoLA-2:06901 |  | 1.30E-05 | 67 |
| BoLA-2:05501 |  | 1.30E-05 | 84 |
| BoLA-2:05401 |  | 1.20E-05 | 47 |
| BoLA-1:01901 |  | 1.20E-05 | 70 |
| BoLA-3:01001 |  | 1.10E-05 | 79 |
| BoLA-1:02901 |  | 1.10E-05 | 72 |
| BoLA-D18.4 |  | 1.00E-05 | 82 |
| BoLA-4:02402 |  | 1.00E-05 | 82 |
| BoLA-1:02301 |  | 1.00E-05 | 82 |
| BoLA-1:00901 |  | 1.00E-05 | 81 |
| BoLA-3:03501 |  | 9.00E-06 | 53 |
| BoLA-1:02001 |  | 7.00E-06 | 86 |
| BoLA-6:03401 |  | 6.00E-06 | 70 |
| BoLA-1:02101 |  | 4.00E-06 | 62 |
| BoLA-6:01501 |  | 3.00E-06 | 82 |
| BoLA-4:02401 |  | 3.00E-06 | 82 |
| BoLA-3:07301 |  | 3.00E-06 | 77 |
| BoLA-2:00801 |  | 3.00E-06 | 80 |
| BoLA-1:02801 |  | 3.00E-06 | 79 |
| BoLA-T5 |  | 2.00E-06 | 92 |
| BoLA-T2b |  | 2.00E-06 | 87 |
| BoLA-HD6 |  | 2.00E-06 | 83 |
| BoLA-6:04101 |  | 2.00E-06 | 87 |
| BoLA-6:01502 |  | 2.00E-06 | 81 |
| BoLA-6:01301 |  | 2.00E-06 | 83 |
| BoLA-2:02201 |  | 2.00E-06 | 90 |
| BoLA-2:00802 |  | 2.00E-06 | 87 |
| BoLA-1:00902 |  | 2.00E-06 | 92 |
| BoLA-6:01302 |  | 1.00E-06 | 86 |

| Alleles | Peptide | Score | Percentile rank |
| --- | --- | --- | --- |
| BoLA-T2a | GQSNEELLK | 0.12982 | 2.4 |
| BoLA-2:01201 |  | 0.12982 | 2.4 |
| BoLA-2:04401 |  | 0.09716 | 3.8 |
| BoLA-1:00901 |  | 0.075997 | 2 |
| BoLA-1:03102 |  | 0.073966 | 1.6 |
| BoLA-T5 |  | 0.059842 | 1.9 |
| BoLA-1:00902 |  | 0.059842 | 1.9 |
| BoLA-2:05501 |  | 0.058732 | 1.6 |
| BoLA-2:06201 |  | 0.055759 | 3.5 |
| BoLA-1:04201 |  | 0.050005 | 2.3 |
| BoLA-1:03101 |  | 0.04841 | 3.5 |
| BoLA-2:03202 |  | 0.042882 | 2.7 |
| BoLA-2:04701 |  | 0.038628 | 4.7 |
| BoLA-D18.4 |  | 0.029149 | 5.7 |
| BoLA-1:02301 |  | 0.029149 | 5.7 |
| BoLA-2:04501 |  | 0.028506 | 5.8 |
| BoLA-1:02001 |  | 0.022806 | 4.2 |
| BoLA-2:04402 |  | 0.019487 | 5.3 |
| BoLA-3:03701 |  | 0.018747 | 18 |
| BoLA-5:00301 |  | 0.015032 | 20 |
| BoLA-3:05801 |  | 0.014282 | 14 |
| BoLA-3:06501 |  | 0.013042 | 20 |
| BoLA-2:04601 |  | 0.012263 | 11 |
| BoLA-6:01402 |  | 0.012062 | 7.4 |
| BoLA-3:06602 |  | 0.01152 | 15 |
| BoLA-5:06401 |  | 0.008296 | 3.9 |
| BoLA-2:07101 |  | 0.007977 | 12 |
| BoLA-1:06101 |  | 0.007444 | 14 |
| BoLA-3:05101 |  | 0.007073 | 9.7 |
| BoLA-2:02201 |  | 0.00671 | 4.9 |
| BoLA-2:04801 |  | 0.006615 | 8.7 |
| BoLA-4:02402 |  | 0.006123 | 12 |
| BoLA-2:05701 |  | 0.005937 | 19 |
| BoLA-amani.1 |  | 0.005703 | 8.8 |
| BoLA-1:06701 |  | 0.005627 | 23 |
| BoLA-3:06601 |  | 0.005454 | 18 |
| BoLA-2:06901 |  | 0.00541 | 8 |
| BoLA-3:06801 |  | 0.005339 | 20 |
| BoLA-2:04301 |  | 0.005244 | 13 |
| BoLA-2:07001 |  | 0.004713 | 9.9 |
| BoLA-2:00602 |  | 0.004417 | 17 |
| BoLA-3:05201 |  | 0.004064 | 15 |
| BoLA-5:07201 |  | 0.003842 | 15 |
| BoLA-2:01602 |  | 0.00358 | 17 |
| BoLA-2:00601 |  | 0.00358 | 17 |
| BoLA-6:01401 |  | 0.002514 | 16 |
| BoLA-3:01101 |  | 0.002391 | 18 |
| BoLA-1:02801 |  | 0.002279 | 11 |
| BoLA-2:00501 |  | 0.002191 | 34 |
| BoLA-2:06001 |  | 0.002047 | 17 |
| BoLA-3:03601 |  | 0.002024 | 27 |
| BoLA-2:02501 |  | 0.001741 | 16 |
| BoLA-6:01501 |  | 0.001502 | 13 |
| BoLA-1:04901 |  | 0.001487 | 23 |
| BoLA-1:02901 |  | 0.001291 | 19 |
| BoLA-2:01601 |  | 0.001263 | 19 |
| BoLA-1:02101 |  | 0.001222 | 4.9 |
| BoLA-4:02401 |  | 0.001022 | 12 |
| BoLA-4:06301 |  | 0.001002 | 16 |
| BoLA-1:01901 |  | 0.000962 | 15 |
| BoLA-2:00801 |  | 0.000961 | 12 |
| BoLA-3:01703 |  | 0.000886 | 42 |
| BoLA-2:00802 |  | 0.000852 | 13 |
| BoLA-2:02603 |  | 0.000818 | 27 |
| BoLA-2:02602 |  | 0.000818 | 27 |
| BoLA-2:02601 |  | 0.000818 | 27 |
| BoLA-3:05001 |  | 0.000716 | 45 |
| BoLA-3:01702 |  | 0.000686 | 36 |
| BoLA-gb1.7 |  | 0.000625 | 32 |
| BoLA-3:05301 |  | 0.000625 | 32 |
| BoLA-3:00403 |  | 0.000625 | 32 |
| BoLA-3:00402 |  | 0.000625 | 32 |
| BoLA-3:00401 |  | 0.000625 | 32 |
| BoLA-6:01502 |  | 0.000619 | 14 |
| BoLA-6:03401 |  | 0.000608 | 17 |
| BoLA-3:03801 |  | 0.000597 | 41 |
| BoLA-3:01001 |  | 0.000537 | 29 |
| BoLA-2:05601 |  | 0.000498 | 29 |
| BoLA-6:04001 |  | 0.000495 | 40 |
| BoLA-HD6 |  | 0.000474 | 23 |
| BoLA-6:01301 |  | 0.000474 | 23 |
| BoLA-3:07301 |  | 0.000402 | 24 |
| BoLA-T2b |  | 0.000342 | 21 |
| BoLA-6:04101 |  | 0.000342 | 21 |
| BoLA-5:03901 |  | 0.000341 | 35 |
| BoLA-T2c |  | 0.000288 | 49 |
| BoLA-3:01701 |  | 0.000268 | 44 |
| BoLA-1:07401 |  | 0.000255 | 25 |
| BoLA-2:05401 |  | 0.000238 | 16 |
| BoLA-2:03001 |  | 0.000233 | 29 |
| BoLA-3:05002 |  | 0.000227 | 54 |
| BoLA-3:05901 |  | 0.000221 | 49 |
| BoLA-3:02702 |  | 0.000184 | 46 |
| BoLA-3:02701 |  | 0.000184 | 46 |
| BoLA-6:01302 |  | 0.000139 | 23 |
| BoLA-JSP.1 |  | 0.000127 | 66 |
| BoLA-3:00201 |  | 0.000127 | 66 |
| BoLA-2:01802 |  | 0.000112 | 46 |
| BoLA-2:01801 |  | 0.000112 | 46 |
| BoLA-T7 |  | 0.00011 | 51 |
| BoLA-3:00103 |  | 5.00E-05 | 49 |
| BoLA-3:00102 |  | 3.70E-05 | 53 |
| BoLA-AW10 |  | 2.60E-05 | 64 |
| BoLA-3:00101 |  | 2.60E-05 | 64 |
| BoLA-3:03501 |  | 1.80E-05 | 44 |
