# Supplementary material for: Development of a multi-epitope chimeric vaccine in silico against Babesia bovis, Theileria annulata, and Anaplasma marginale using computational biology tools and reverse vaccinology approach
Source: PLoS One. 2025 Jan 24;20(1):e0312262. doi: 10.1371/journal.pone.0312262 (PMC11759392; doi:10.1371/journal.pone.0312262)
Supplement: S4 File — (DOCX) [file pone.0312262.s010.docx]

The tables of all the ten MHC I epitopes of SPAG-1 with their scores and percentile rank representing their affinities for different BOLA alleles. The peptide which has been selected for chimeric vaccine construction has been represented in bold letters. The BoLA alleles binding with the selected peptide possessing a percentile value >50 is highlighted as yellow. The BoLA allele that has bonded with the selected peptide with the lowest percentile rank is highlighted as green.

| Alleles | Peptide | Score | Percentile rank |
| --- | --- | --- | --- |
| BoLA-2:01801 | **GPGGNGEGG** | 0.00151 | 18 |
| BoLA-2:01802 |  | 0.00151 | 18 |
| BoLA-3:01702 |  | 0.000673 | 36 |
| BoLA-2:00501 |  | 0.00064 | 52 |
| BoLA-3:01703 |  | 0.000514 | 49 |
| BoLA-6:01402 |  | 0.000383 | 37 |
| BoLA-2:01601 |  | 0.00034 | 33 |
| BoLA-2:00602 |  | 0.000344 | 46 |
| BoLA-2:01601 |  | 0.00034 | 33 |
| BoLA-2:00601 |  | 0.000312 | 44 |
| BoLA-2:01602 |  | 0.000312 | 44 |
| BoLA-3:01701 |  | 0.000297 | 42 |
| BoLA-1:06101 |  | 0.000239 | 48 |
| BoLA-3:03701 |  | 0.000174 | 80 |
| BoLA-3:01001 |  | 0.000171 | 42 |
| BoLA-6:01401 |  | 0.000144 | 48 |
| BoLA-5:00301 |  | 0.000132 | 80 |
| BoLA-2:06201 |  | 0.000128 | 65 |
| BoLA-3:03801 |  | 0.000127 | 61 |
| BoLA-2:03001 |  | 0.000104 | 38 |
| BoLA-1:06701 |  | 9.6e-05 | 71 |
| BoLA-1:01901 |  | 9e-05 | 39 |
| BoLA-2:06001 |  | 8e-05 | 57 |
| BoLA-3:05002 |  | 7.2e-05 | 68 |
| BoLA-2:05701 |  | 6.1e-05 | 72 |
| BoLA-2:02501 |  | 5.2e-05 | 58 |
| BoLA-3:02701 |  | 5.2e-05 | 65 |
| BoLA-3:02702 |  | 5.2e-05 | 65 |
| BoLA-3:05001 |  | 4.4e-05 | 82 |
| BoLA-1:02001 |  | 4.1e-05 | 62 |
| BoLA-2:04401 |  | 4e-05 | 88 |
| BoLA-3:05801 |  | 4e-05 | 85 |
| BoLA-3:05901 |  | 3.4e-05 | 77 |
| BoLA-1:00901 |  | 3.1e-05 | 67 |
| BoLA-2:02201 |  | 3.1e-05 | 52 |
| BoLA-2:04301 |  | 3.1e-05 | 75 |
| BoLA-2:04801 |  | 3.1e-05 | 62 |
| BoLA-3:00401 |  | 3.1e-05 | 72 |
| BoLA-3:00402 |  | 3.1e-05 | 72 |
| BoLA-3:00403 |  | 3.1e-05 | 72 |
| BoLA-3:05301 |  | 3.1e-05 | 72 |
| BoLA-gb1.7 |  | 3.1e-05 | 72 |
| BoLA-1:03102 |  | 2.8e-05 | 82 |
| BoLA-2:04701 |  | 2.5e-05 | 84 |
| BoLA-3:06501 |  | 2.5e-05 | 89 |
| BoLA-3:06801 |  | 2.5e-05 | 77 |
| BoLA-3:00101 |  | 2.2e-05 | 67 |
| BoLA-3:00102 |  | 2.2e-05 | 61 |
| BoLA-AW10 |  | 2.2e-05 | 67 |
| BoLA-2:01201 |  | 2.1e-05 | 80 |
| BoLA-T2a |  | 2.1e-05 | 80 |
| BoLA-1:03101 |  | 1.7e-05 | 86 |
| BoLA-3:00103 |  | 1.7e-05 | 65 |
| BoLA-4:02402 |  | 1.7e-05 | 75 |
| BoLA-1:02101 |  | 1.6e-05 | 41 |
| BoLA-1:02901 |  | 1.6e-05 | 67 |
| BoLA-3:01101 |  | 1.6e-05 | 75 |
| BoLA-5:07201 |  | 1.6e-05 | 73 |
| BoLA-1:02301 |  | 1.5e-05 | 77 |
| BoLA-2:05601 |  | 1.5e-05 | 71 |
| BoLA-2:07001 |  | 1.5e-05 | 69 |
| BoLA-6:04001 |  | 1.5e-05 | 90 |
| BoLA-D18.4 |  | 1.5e-05 | 77 |
| BoLA-T2c |  | 1.4e-05 | 80 |
| BoLA-1:04201 |  | 1.3e-05 | 81 |
| BoLA-3:03601 |  | 1.3e-05 | 85 |
| BoLA-6:01501 |  | 1.3e-05 | 63 |
| BoLA-2:02601 |  | 1.2e-05 | 81 |
| BoLA-2:02602 |  | 1.2e-05 | 81 |
| BoLA-2:02603 |  | 1.2e-05 | 81 |
| BoLA-3:00201 |  | 1.2e-05 | 94 |
| BoLA-amani.1 |  | 1.2e-05 | 75 |
| BoLA-JSP.1 |  | 1.2e-05 | 94 |
| BoLA-2:04501 |  | 1.1e-05 | 88 |
| BoLA-6:03401 |  | 1.1e-05 | 61 |
| BoLA-1:07401 |  | 1e-05 | 62 |
| BoLA-2:03202 |  | 1e-05 | 84 |
| BoLA-2:05501 |  | 1e-05 | 87 |
| BoLA-4:02401 |  | 1e-05 | 65 |
| BoLA-2:04601 |  | 9e-06 | 89 |
| BoLA-1:00902 |  | 8e-06 | 79 |
| BoLA-5:03901 |  | 8e-06 | 84 |
| BoLA-T5 |  | 8e-06 | 79 |
| BoLA-2:04402 |  | 7e-06 | 86 |
| BoLA-3:05101 |  | 7e-06 | 86 |
| BoLA-3:07301 |  | 7e-06 | 67 |
| BoLA-6:04101 |  | 7e-06 | 69 |
| BoLA-T2b |  | 7e-06 | 69 |
| BoLA-2:06901 |  | 6e-06 | 76 |
| BoLA-3:06601 |  | 6e-06 | 92 |
| BoLA-3:06602 |  | 5e-06 | 95 |
| BoLA-1:02801 |  | 4e-06 | 75 |
| BoLA-1:04901 |  | 4e-06 | 90 |
| BoLA-2:07101 |  | 4e-06 | 93 |
| BoLA-6:01502 |  | 3e-06 | 75 |
| BoLA-T7 |  | 3e-06 | 92 |
| BoLA-2:00801 |  | 2e-06 | 86 |
| BoLA-3:05201 |  | 2e-06 | 92 |
| BoLA-4:06301 |  | 2e-06 | 80 |
| BoLA-2:00802 |  | 1e-06 | 95 |
| BoLA-3:03501 |  | 1e-06 | 86 |
| BoLA-5:06401 |  | 1e-06 | 90 |
| BoLA-6:01301 |  | 1e-06 | 92 |
| BoLA-HD6 |  | 1e-06 | 92 |
| BoLA-2:05401 |  | 0.0 | 100 |
| BoLA-6:01302 |  | 0.0 | 100 |

| Alleles | Peptide | Score | Percentile rank |
| --- | --- | --- | --- |
| BoLA-3:01703 | TGPGGNGEG | 0.015284 | 12 |
| BoLA-3:01703 |  | 0.015284 | 12 |
| BoLA-3:01702 |  | 0.01252 | 9.8 |
| BoLA-3:01701 |  | 0.004866 | 13 |
| BoLA-3:03701 |  | 0.004464 | 38 |
| BoLA-3:00201 |  | 0.00254 | 24 |
| BoLA-JSP.1 |  | 0.00254 | 24 |
| BoLA-3:00401 |  | 0.001599 | 22 |
| BoLA-3:00402 |  | 0.001599 | 22 |
| BoLA-3:00403 |  | 0.001599 | 22 |
| BoLA-3:05301 |  | 0.001599 | 22 |
| BoLA-gb1.7 |  | 0.001599 | 22 |
| BoLA-1:06701 |  | 0.001492 | 37 |
| BoLA-5:03901 |  | 0.00143 | 21 |
| BoLA-3:05801 |  | 0.001369 | 40 |
| BoLA-2:00601 |  | 0.001268 | 27 |
| BoLA-2:01602 |  | 0.001268 | 27 |
| BoLA-3:05002 |  | 0.001089 | 34 |
| BoLA-3:05901 |  | 0.001085 | 28 |
| BoLA-3:00101 |  | 0.001006 | 18 |
| BoLA-AW10 |  | 0.001006 | 18 |
| BoLA-3:02701 |  | 0.000996 | 25 |
| BoLA-3:02702 |  | 0.000996 | 25 |
| BoLA-6:04001 |  | 0.000965 | 31 |
| BoLA-3:00103 |  | 0.000724 | 19 |
| BoLA-3:00102 |  | 0.000615 | 19 |
| BoLA-3:03801 |  | 0.000598 | 41 |
| BoLA-3:05001 |  | 0.000595 | 48 |
| BoLA-3:06501 |  | 0.000429 | 58 |
| BoLA-2:07001 |  | 0.000424 | 29 |
| BoLA-3:06801 |  | 0.00038 | 47 |
| BoLA-3:01101 |  | 0.00035 | 36 |
| BoLA-2:00602 |  | 0.000322 | 47 |
| BoLA-2:05701 |  | 0.000309 | 51 |
| BoLA-3:03601 |  | 0.000292 | 50 |
| BoLA-1:06101 |  | 0.000277 | 47 |
| BoLA-2:04401 |  | 0.000276 | 68 |
| BoLA-5:07201 |  | 0.000264 | 37 |
| BoLA-2:04402 |  | 0.000252 | 44 |
| BoLA-3:06602 |  | 0.00025 | 56 |
| BoLA-2:04701 |  | 0.000234 | 57 |
| BoLA-2:00501 |  | 0.000228 | 66 |
| BoLA-2:04601 |  | 0.000206 | 55 |
| BoLA-3:06601 |  | 0.000184 | 55 |
| BoLA-2:04301 |  | 0.000179 | 51 |
| BoLA-5:00301 |  | 0.000147 | 78 |
| BoLA-3:05101 |  | 0.000116 | 53 |
| BoLA-2:03001 |  | 0.000112 | 37 |
| BoLA-3:05201 |  | 7.3e-05 | 53 |
| BoLA-2:01601 |  | 7.1e-05 | 56 |
| BoLA-2:02501 |  | 7.1e-05 | 53 |
| BoLA-T7 |  | 6.5e-05 | 57 |
| BoLA-3:01001 |  | 6.2e-05 | 56 |
| BoLA-2:05601 |  | 5.6e-05 | 55 |
| BoLA-2:06001 |  | 5.3e-05 | 63 |
| BoLA-amani.1 |  | 5e-05 | 56 |
| BoLA-3:03501 |  | 4.7e-05 | 32 |
| BoLA-2:02601 |  | 4.4e-05 | 64 |
| BoLA-2:02602 |  | 4.4e-05 | 64 |
| BoLA-2:02603 |  | 4.4e-05 | 64 |
| BoLA-6:01402 |  | 4.1e-05 | 70 |
| BoLA-2:04501 |  | 3.6e-05 | 76 |
| BoLA-T2c |  | 3.3e-05 | 72 |
| BoLA-2:01801 |  | 3.1e-05 | 65 |
| BoLA-2:01802 |  | 3.1e-05 | 65 |
| BoLA-6:01401 |  | 3.1e-05 | 69 |
| BoLA-2:01201 |  | 3e-05 | 76 |
| BoLA-T2a |  | 3e-05 | 76 |
| BoLA-2:07101 |  | 2.8e-05 | 75 |
| BoLA-1:02301 |  | 2.4e-05 | 72 |
| BoLA-D18.4 |  | 2.4e-05 | 72 |
| BoLA-4:06301 |  | 2.3e-05 | 50 |
| BoLA-1:01901 |  | 2.2e-05 | 60 |
| BoLA-1:03101 |  | 2.2e-05 | 84 |
| BoLA-2:06201 |  | 2.2e-05 | 85 |
| BoLA-1:02901 |  | 1.8e-05 | 66 |
| BoLA-2:03202 |  | 1.7e-05 | 79 |
| BoLA-2:04801 |  | 1.7e-05 | 70 |
| BoLA-1:00901 |  | 1.6e-05 | 76 |
| BoLA-1:04901 |  | 1.5e-05 | 76 |
| BoLA-5:06401 |  | 1.4e-05 | 54 |
| BoLA-3:07301 |  | 1.3e-05 | 59 |
| BoLA-1:02101 |  | 1.1e-05 | 46 |
| BoLA-2:06901 |  | 1e-05 | 70 |
| BoLA-6:01501 |  | 1e-05 | 67 |
| BoLA-1:00902 |  | 9e-06 | 77 |
| BoLA-T5 |  | 9e-06 | 77 |
| BoLA-1:03102 |  | 8e-06 | 93 |
| BoLA-4:02402 |  | 8e-06 | 84 |
| BoLA-1:02001 |  | 7e-06 | 86 |
| BoLA-1:02801 |  | 6e-06 | 70 |
| BoLA-1:04201 |  | 6e-06 | 89 |
| BoLA-6:04101 |  | 6e-06 | 71 |
| BoLA-T2b |  | 6e-06 | 71 |
| BoLA-1:07401 |  | 5e-06 | 71 |
| BoLA-2:00801 |  | 4e-06 | 76 |
| BoLA-2:00802 |  | 4e-06 | 78 |
| BoLA-2:05501 |  | 4e-06 | 94 |
| BoLA-6:03401 |  | 4e-06 | 75 |
| BoLA-2:05401 |  | 3e-06 | 67 |
| BoLA-6:01502 |  | 3e-06 | 75 |
| BoLA-2:02201 |  | 2e-06 | 90 |
| BoLA-4:02401 |  | 2e-06 | 87 |
| BoLA-6:01301 |  | 1e-06 | 92 |
| BoLA-6:01302 |  | 1e-06 | 86 |
| BoLA-HD6 |  | 1e-06 | 92 |

| Alleles | Peptide | Score | Percentile rank |
| --- | --- | --- | --- |
| BoLA-2:04401 | TTTGPGGNG | 0.012515 | 20 |
| BoLA-3:03601 |  | 0.00772 | 15 |
| BoLA-2:07001 |  | 0.00426 | 11 |
| BoLA-2:04402 |  | 0.00393 | 15 |
| BoLA-2:04501 |  | 0.003863 | 20 |
| BoLA-1:06701 |  | 0.003604 | 27 |
| BoLA-2:06201 |  | 0.003451 | 22 |
| BoLA-3:03701 |  | 0.003375 | 42 |
| BoLA-3:05001 |  | 0.002133 | 30 |
| BoLA-3:01703 |  | 0.001865 | 33 |
| BoLA-T7 |  | 0.001752 | 23 |
| BoLA-2:07101 |  | 0.00127 | 28 |
| BoLA-3:06801 |  | 0.001166 | 34 |
| BoLA-3:06601 |  | 0.001053 | 33 |
| BoLA-3:01101 |  | 0.000941 | 26 |
| BoLA-amani.1 |  | 0.00089 | 22 |
| BoLA-3:00401 |  | 0.000873 | 28 |
| BoLA-3:00402 |  | 0.000873 | 28 |
| BoLA-3:00403 |  | 0.000873 | 28 |
| BoLA-3:05301 |  | 0.000873 | 28 |
| BoLA-gb1.7 |  | 0.000873 | 28 |
| BoLA-3:05801 |  | 0.000872 | 46 |
| BoLA-2:03202 |  | 0.00083 | 30 |
| BoLA-2:05701 |  | 0.00081 | 39 |
| BoLA-2:04701 |  | 0.000739 | 41 |
| BoLA-3:03801 |  | 0.000705 | 39 |
| BoLA-2:05601 |  | 0.000702 | 26 |
| BoLA-3:01701 |  | 0.000687 | 31 |
| BoLA-2:04601 |  | 0.000664 | 40 |
| BoLA-3:06602 |  | 0.000628 | 44 |
| BoLA-3:01702 |  | 0.000607 | 38 |
| BoLA-1:06101 |  | 0.00056 | 39 |
| BoLA-2:00501 |  | 0.000542 | 54 |
| BoLA-3:05901 |  | 0.000533 | 37 |
| BoLA-2:00602 |  | 0.000469 | 42 |
| BoLA-3:05002 |  | 0.000451 | 45 |
| BoLA-5:00301 |  | 0.000439 | 65 |
| BoLA-2:01201 |  | 0.000421 | 39 |
| BoLA-T2a |  | 0.000421 | 39 |
| BoLA-5:03901 |  | 0.000299 | 37 |
| BoLA-3:05201 |  | 0.000291 | 37 |
| BoLA-2:01601 |  | 0.000252 | 37 |
| BoLA-2:01801 |  | 0.000245 | 35 |
| BoLA-2:01802 |  | 0.000245 | 35 |
| BoLA-3:00201 |  | 0.000241 | 55 |
| BoLA-JSP.1 |  | 0.000241 | 55 |
| BoLA-3:02701 |  | 0.000238 | 43 |
| BoLA-3:02702 |  | 0.000238 | 43 |
| BoLA-6:01402 |  | 0.000217 | 45 |
| BoLA-2:00801 |  | 0.00021 | 24 |
| BoLA-2:04301 |  | 0.000205 | 49 |
| BoLA-3:00101 |  | 0.000185 | 35 |
| BoLA-AW10 |  | 0.000185 | 35 |
| BoLA-3:05101 |  | 0.000183 | 47 |
| BoLA-2:00601 |  | 0.000159 | 54 |
| BoLA-2:01602 |  | 0.000159 | 54 |
| BoLA-2:06901 |  | 0.000155 | 36 |
| BoLA-2:05401 |  | 0.000151 | 19 |
| BoLA-6:01401 |  | 0.000144 | 48 |
| BoLA-3:06501 |  | 0.000125 | 73 |
| BoLA-2:05501 |  | 0.00012 | 57 |
| BoLA-T2c |  | 0.000119 | 58 |
| BoLA-1:00901 |  | 0.000111 | 50 |
| BoLA-2:00802 |  | 0.000106 | 31 |
| BoLA-2:02601 |  | 0.000106 | 52 |
| BoLA-2:02602 |  | 0.000106 | 52 |
| BoLA-2:02603 |  | 0.000106 | 52 |
| BoLA-1:03102 |  | 0.000104 | 65 |
| BoLA-1:03101 |  | 9.5e-05 | 66 |
| BoLA-3:01001 |  | 9.1e-05 | 50 |
| BoLA-5:06401 |  | 8.7e-05 | 33 |
| BoLA-4:06301 |  | 8.3e-05 | 36 |
| BoLA-3:00103 |  | 7.6e-05 | 43 |
| BoLA-1:04201 |  | 6.8e-05 | 61 |
| BoLA-4:02402 |  | 6.7e-05 | 57 |
| BoLA-6:04001 |  | 6.7e-05 | 71 |
| BoLA-2:06001 |  | 6.6e-05 | 60 |
| BoLA-1:02301 |  | 6.4e-05 | 59 |
| BoLA-D18.4 |  | 6.4e-05 | 59 |
| BoLA-3:00102 |  | 6e-05 | 46 |
| BoLA-5:07201 |  | 5.4e-05 | 57 |
| BoLA-2:02501 |  | 5.3e-05 | 57 |
| BoLA-1:02101 |  | 5.2e-05 | 26 |
| BoLA-2:03001 |  | 5.1e-05 | 48 |
| BoLA-2:04801 |  | 5e-05 | 55 |
| BoLA-1:00902 |  | 3.9e-05 | 59 |
| BoLA-T5 |  | 3.9e-05 | 59 |
| BoLA-1:04901 |  | 3.3e-05 | 66 |
| BoLA-1:02001 |  | 3.2e-05 | 66 |
| BoLA-4:02401 |  | 3.1e-05 | 48 |
| BoLA-1:02901 |  | 2.4e-05 | 62 |
| BoLA-6:04101 |  | 2.1e-05 | 53 |
| BoLA-T2b |  | 2.1e-05 | 53 |
| BoLA-6:03401 |  | 1.9e-05 | 54 |
| BoLA-1:01901 |  | 1.8e-05 | 63 |
| BoLA-1:02801 |  | 1.7e-05 | 57 |
| BoLA-3:03501 |  | 1.3e-05 | 48 |
| BoLA-3:07301 |  | 1.1e-05 | 61 |
| BoLA-6:01501 |  | 9e-06 | 68 |
| BoLA-1:07401 |  | 7e-06 | 67 |
| BoLA-6:01502 |  | 6e-06 | 65 |
| BoLA-6:01301 |  | 4e-06 | 74 |
| BoLA-HD6 |  | 4e-06 | 74 |
| BoLA-2:02201 |  | 3e-06 | 86 |
| BoLA-6:01302 |  | 2e-06 | 74 |

| Alleles | Peptide | Score | Percentile rank |
| --- | --- | --- | --- |
| BoLA-2:00501 | TTGPGGNGE | 0.004399 | 26 |
| BoLA-2:04402 |  | 0.004166 | 15 |
| BoLA-2:04501 |  | 0.004154 | 20 |
| BoLA-3:03701 |  | 0.003858 | 40 |
| BoLA-2:04701 |  | 0.002759 | 26 |
| BoLA-2:01201 |  | 0.002595 | 20 |
| BoLA-T2a |  | 0.002595 | 20 |
| BoLA-2:04401 |  | 0.002571 | 39 |
| BoLA-2:07001 |  | 0.002303 | 15 |
| BoLA-2:03202 |  | 0.002097 | 21 |
| BoLA-1:06701 |  | 0.001906 | 34 |
| BoLA-3:03601 |  | 0.001658 | 29 |
| BoLA-2:07101 |  | 0.001605 | 25 |
| BoLA-2:04601 |  | 0.001024 | 35 |
| BoLA-3:01703 |  | 0.000723 | 45 |
| BoLA-5:07201 |  | 0.000713 | 28 |
| BoLA-3:05001 |  | 0.000681 | 46 |
| BoLA-3:06601 |  | 0.000669 | 39 |
| BoLA-3:05201 |  | 0.000647 | 29 |
| BoLA-2:05601 |  | 0.000646 | 26 |
| BoLA-T2c |  | 0.000635 | 41 |
| BoLA-2:06201 |  | 0.000603 | 43 |
| BoLA-HD6 |  | 0.000594 | 21 |
| BoLA-HD6 |  | 0.000591 | 21 |
| BoLA-3:06602 |  | 0.000547 | 45 |
| BoLA-1:06101 |  | 0.00051 | 40 |
| BoLA-2:00601 |  | 0.000501 | 38 |
| BoLA-2:01602 |  | 0.000501 | 38 |
| BoLA-2:05701 |  | 0.000469 | 46 |
| BoLA-1:03102 |  | 0.000436 | 44 |
| BoLA-5:00301 |  | 0.000424 | 65 |
| BoLA-3:06801 |  | 0.000401 | 46 |
| BoLA-3:05101 |  | 0.000378 | 38 |
| BoLA-1:03101 |  | 0.000357 | 48 |
| BoLA-3:05002 |  | 0.000304 | 50 |
| BoLA-3:01702 |  | 0.00028 | 48 |
| BoLA-2:00602 |  | 0.000265 | 50 |
| BoLA-6:01402 |  | 0.00026 | 43 |
| BoLA-3:03801 |  | 0.000245 | 52 |
| BoLA-1:02901 |  | 0.000234 | 34 |
| BoLA-1:00901 |  | 0.000232 | 40 |
| BoLA-2:04301 |  | 0.00023 | 48 |
| BoLA-3:01701 |  | 0.00023 | 46 |
| BoLA-3:05801 |  | 0.000226 | 65 |
| BoLA-3:00401 |  | 0.000223 | 45 |
| BoLA-3:00402 |  | 0.000223 | 45 |
| BoLA-3:00403 |  | 0.000223 | 45 |
| BoLA-3:05301 |  | 0.000223 | 45 |
| BoLA-gb1.7 |  | 0.000223 | 45 |
| BoLA-amani.1 |  | 0.00022 | 37 |
| BoLA-2:02501 |  | 0.0002 | 38 |
| BoLA-3:06501 |  | 0.000194 | 68 |
| BoLA-2:01601 |  | 0.000174 | 43 |
| BoLA-T7 |  | 0.00017 | 46 |
| BoLA-3:00201 |  | 0.000163 | 62 |
| BoLA-JSP.1 |  | 0.000163 | 62 |
| BoLA-3:01001 |  | 0.000148 | 44 |
| BoLA-5:03901 |  | 0.000138 | 46 |
| BoLA-2:06001 |  | 0.000131 | 50 |
| BoLA-2:01801 |  | 0.000121 | 45 |
| BoLA-2:01802 |  | 0.000121 | 45 |
| BoLA-2:05501 |  | 0.000119 | 57 |
| BoLA-3:01101 |  | 0.000103 | 51 |
| BoLA-1:02001 |  | 0.000101 | 49 |
| BoLA-2:06901 |  | 9.6e-05 | 41 |
| BoLA-6:01401 |  | 8.9e-05 | 55 |
| BoLA-4:02402 |  | 8.2e-05 | 54 |
| BoLA-3:02701 |  | 6.6e-05 | 62 |
| BoLA-3:02702 |  | 6.6e-05 | 62 |
| BoLA-2:00801 |  | 6.3e-05 | 37 |
| BoLA-6:04001 |  | 6.3e-05 | 72 |
| BoLA-1:00902 |  | 6.2e-05 | 53 |
| BoLA-T5 |  | 6.2e-05 | 53 |
| BoLA-1:04901 |  | 6e-05 | 58 |
| BoLA-2:00802 |  | 5.7e-05 | 39 |
| BoLA-3:05901 |  | 5.7e-05 | 70 |
| BoLA-1:02301 |  | 5.1e-05 | 62 |
| BoLA-D18.4 |  | 5.1e-05 | 62 |
| BoLA-2:03001 |  | 5e-05 | 48 |
| BoLA-1:04201 |  | 4.9e-05 | 65 |
| BoLA-2:04801 |  | 4.9e-05 | 56 |
| BoLA-3:00101 |  | 4.1e-05 | 57 |
| BoLA-AW10 |  | 4.1e-05 | 57 |
| BoLA-2:02601 |  | 4e-05 | 66 |
| BoLA-2:02602 |  | 4e-05 | 66 |
| BoLA-2:02603 |  | 4e-05 | 66 |
| BoLA-4:06301 |  | 3.5e-05 | 45 |
| BoLA-1:01901 |  | 3.3e-05 | 54 |
| BoLA-1:07401 |  | 2.7e-05 | 49 |
| BoLA-2:05401 |  | 2.7e-05 | 37 |
| BoLA-3:07301 |  | 2.7e-05 | 50 |
| BoLA-6:01501 |  | 2.5e-05 | 54 |
| BoLA-1:02101 |  | 2.4e-05 | 35 |
| BoLA-3:00103 |  | 2.1e-05 | 62 |
| BoLA-5:06401 |  | 2.1e-05 | 49 |
| BoLA-6:03401 |  | 2e-05 | 53 |
| BoLA-3:00102 |  | 1.7e-05 | 65 |
| BoLA-4:02401 |  | 1.4e-05 | 60 |
| BoLA-3:03501 |  | 1.3e-05 | 48 |
| BoLA-6:01502 |  | 1.3e-05 | 54 |
| BoLA-1:02801 |  | 1.2e-05 | 61 |
| BoLA-2:02201 |  | 9e-06 | 70 |
| BoLA-6:04101 |  | 7e-06 | 69 |
| BoLA-T2b |  | 7e-06 | 69 |
| BoLA-6:01301 |  | 3e-06 | 78 |
| BoLA-HD6 |  | 3e-06 | 78 |
| BoLA-6:01302 |  | 1e-06 | 86 |

| Alleles | Peptide | Score | Percentile rank |
| --- | --- | --- | --- |
| BoLA-3:01703 | GSGSDEDED | 6.2e-05 | 76 |
| BoLA-2:00501 |  | 2.2e-05 | 91 |
| BoLA-3:03701 |  | 2.2e-05 | 95 |
| BoLA-6:01402 |  | 1.9e-05 | 80 |
| BoLA-2:03001 |  | 1.3e-05 | 66 |
| BoLA-3:01702 |  | 1.2e-05 | 86 |
| BoLA-3:05801 |  | 1.2e-05 | 94 |
| BoLA-1:06701 |  | 9e-06 | 92 |
| BoLA-2:06201 |  | 9e-06 | 93 |
| BoLA-3:06501 |  | 9e-06 | 95 |
| BoLA-2:04401 |  | 8e-06 | 97 |
| BoLA-2:07101 |  | 8e-06 | 88 |
| BoLA-3:00401 |  | 7e-06 | 88 |
| BoLA-3:00402 |  | 7e-06 | 88 |
| BoLA-3:00403 |  | 7e-06 | 88 |
| BoLA-3:05301 |  | 7e-06 | 88 |
| BoLA-gb1.7 |  | 7e-06 | 88 |
| BoLA-1:03101 |  | 6e-06 | 94 |
| BoLA-2:04501 |  | 6e-06 | 92 |
| BoLA-2:07001 |  | 6e-06 | 80 |
| BoLA-3:02701 |  | 6e-06 | 92 |
| BoLA-3:02702 |  | 6e-06 | 92 |
| BoLA-3:05901 |  | 6e-06 | 94 |
| BoLA-3:06602 |  | 6e-06 | 94 |
| BoLA-5:03901 |  | 6e-06 | 87 |
| BoLA-1:03102 |  | 5e-06 | 96 |
| BoLA-2:05601 |  | 5e-06 | 83 |
| BoLA-3:01101 |  | 5e-06 | 87 |
| BoLA-3:06801 |  | 5e-06 | 91 |
| BoLA-2:00602 |  | 4e-06 | 96 |
| BoLA-3:06601 |  | 4e-06 | 94 |
| BoLA-5:00301 |  | 4e-06 | 99 |
| BoLA-1:06101 |  | 3e-06 | 93 |
| BoLA-2:00601 |  | 3e-06 | 96 |
| BoLA-2:00801 |  | 3e-06 | 80 |
| BoLA-2:01201 |  | 3e-06 | 96 |
| BoLA-2:01602 |  | 3e-06 | 96 |
| BoLA-2:04402 |  | 3e-06 | 93 |
| BoLA-2:04701 |  | 3e-06 | 98 |
| BoLA-3:00201 |  | 3e-06 | 99 |
| BoLA-3:01701 |  | 3e-06 | 97 |
| BoLA-6:04001 |  | 3e-06 | 99 |
| BoLA-amani.1 |  | 3e-06 | 90 |
| BoLA-JSP.1 |  | 3e-06 | 99 |
| BoLA-T2a |  | 3e-06 | 96 |
| BoLA-1:02001 |  | 2e-06 | 96 |
| BoLA-1:02101 |  | 2e-06 | 73 |
| BoLA-1:02301 |  | 2e-06 | 95 |
| BoLA-2:02501 |  | 2e-06 | 97 |
| BoLA-2:03202 |  | 2e-06 | 96 |
| BoLA-2:04301 |  | 2e-06 | 98 |
| BoLA-2:04601 |  | 2e-06 | 98 |
| BoLA-2:06001 |  | 2e-06 | 98 |
| BoLA-3:00101 |  | 2e-06 | 95 |
| BoLA-3:00103 |  | 2e-06 | 92 |
| BoLA-3:05001 |  | 2e-06 | 99 |
| BoLA-3:05002 |  | 2e-06 | 98 |
| BoLA-5:07201 |  | 2e-06 | 94 |
| BoLA-AW10 |  | 2e-06 | 95 |
| BoLA-D18.4 |  | 2e-06 | 95 |
| BoLA-1:00901 |  | 1e-06 | 99 |
| BoLA-1:00902 |  | 1e-06 | 97 |
| BoLA-1:02901 |  | 1e-06 | 97 |
| BoLA-1:04201 |  | 1e-06 | 99 |
| BoLA-1:04901 |  | 1e-06 | 99 |
| BoLA-2:00802 |  | 1e-06 | 95 |
| BoLA-2:01601 |  | 1e-06 | 99 |
| BoLA-2:01801 |  | 1e-06 | 99 |
| BoLA-2:01802 |  | 1e-06 | 99 |
| BoLA-2:02601 |  | 1e-06 | 100 |
| BoLA-2:02602 |  | 1e-06 | 100 |
| BoLA-2:02603 |  | 1e-06 | 100 |
| BoLA-2:04801 |  | 1e-06 | 98 |
| BoLA-2:05501 |  | 1e-06 | 100 |
| BoLA-2:05701 |  | 1e-06 | 100 |
| BoLA-3:00102 |  | 1e-06 | 98 |
| BoLA-3:03501 |  | 1e-06 | 86 |
| BoLA-3:03601 |  | 1e-06 | 99 |
| BoLA-3:03801 |  | 1e-06 | 100 |
| BoLA-3:05101 |  | 1e-06 | 99 |
| BoLA-3:05201 |  | 1e-06 | 97 |
| BoLA-4:02402 |  | 1e-06 | 99 |
| BoLA-5:06401 |  | 1e-06 | 90 |
| BoLA-6:01401 |  | 1e-06 | 99 |
| BoLA-T5 |  | 1e-06 | 97 |
| BoLA-1:01901 |  | 0.0 | 100 |
| BoLA-1:02801 |  | 0.0 | 100 |
| BoLA-1:07401 |  | 0.0 | 100 |
| BoLA-2:02201 |  | 0.0 | 100 |
| BoLA-2:05401 |  | 0.0 | 100 |
| BoLA-2:06901 |  | 0.0 | 100 |
| BoLA-3:01001 |  | 0.0 | 100 |
| BoLA-3:07301 |  | 0.0 | 100 |
| BoLA-4:02401 |  | 0.0 | 100 |
| BoLA-4:06301 |  | 0.0 | 100 |
| BoLA-6:01301 |  | 0.0 | 100 |
| BoLA-6:01302 |  | 0.0 | 100 |
| BoLA-6:01501 |  | 0.0 | 100 |
| BoLA-6:01502 |  | 0.0 | 100 |
| BoLA-6:03401 |  | 0.0 | 100 |
| BoLA-6:04101 |  | 0.0 | 100 |
| BoLA-HD6 |  | 0.0 | 100 |
| BoLA-T2b |  | 0.0 | 100 |
| BoLA-T2c |  | 0.0 | 100 |
| BoLA-T7 |  | 0.0 | 100 |

| Allele | Peptide | Score | Percentile rank |
| --- | --- | --- | --- |
| BoLA-3:01701 | KGQGSGLQG | 0.051018 | 2.1 |
| BoLA-3:01703 |  | 0.036336 | 6.6 |
| BoLA-3:01702 |  | 0.033102 | 4.8 |
| BoLA-3:01101 |  | 0.019226 | 6.6 |
| BoLA-2:04701 |  | 0.014476 | 11 |
| BoLA-5:00301 |  | 0.010403 | 24 |
| BoLA-3:05801 |  | 0.007324 | 20 |
| BoLA-3:03701 |  | 0.006531 | 32 |
| BoLA-3:06801 |  | 0.006524 | 18 |
| BoLA-6:04001 |  | 0.006448 | 14 |
| BoLA-2:04401 |  | 0.005831 | 29 |
| BoLA-2:04601 |  | 0.00576 | 16 |
| BoLA-3:05101 |  | 0.004679 | 13 |
| BoLA-1:06101 |  | 0.004533 | 18 |
| BoLA-3:06501 |  | 0.004465 | 30 |
| BoLA-2:06201 |  | 0.0038 | 21 |
| BoLA-3:06602 |  | 0.003519 | 24 |
| BoLA-2:04301 |  | 0.00347 | 16 |
| BoLA-5:03901 |  | 0.002759 | 16 |
| BoLA-2:04402 |  | 0.002675 | 18 |
| BoLA-2:00501 |  | 0.002342 | 33 |
| BoLA-3:05002 |  | 0.00225 | 26 |
| BoLA-2:01201 |  | 0.002133 | 21 |
| BoLA-T2a |  | 0.002133 | 21 |
| BoLA-3:06601 |  | 0.001969 | 27 |
| BoLA-1:06701 |  | 0.001716 | 35 |
| BoLA-2:00601 |  | 0.001326 | 27 |
| BoLA-2:01602 |  | 0.001326 | 27 |
| BoLA-3:00401 |  | 0.001286 | 24 |
| BoLA-3:00402 |  | 0.001286 | 24 |
| BoLA-3:00403 |  | 0.001286 | 24 |
| BoLA-3:05301 |  | 0.001286 | 24 |
| BoLA-gb1.7 |  | 0.001286 | 24 |
| BoLA-3:00102 |  | 0.00125 | 13 |
| BoLA-2:03202 |  | 0.001242 | 26 |
| BoLA-2:04501 |  | 0.001164 | 33 |
| BoLA-3:03501 |  | 0.001064 | 7.7 |
| BoLA-amani.1 |  | 0.00102 | 21 |
| BoLA-3:00101 |  | 0.001006 | 18 |
| BoLA-AW10 |  | 0.001006 | 18 |
| BoLA-6:01401 |  | 0.000985 | 25 |
| BoLA-3:05001 |  | 0.000867 | 42 |
| BoLA-2:07001 |  | 0.000836 | 23 |
| BoLA-1:03101 |  | 0.000766 | 38 |
| BoLA-5:07201 |  | 0.000659 | 28 |
| BoLA-2:05601 |  | 0.000642 | 26 |
| BoLA-2:02601 |  | 0.000629 | 30 |
| BoLA-2:02602 |  | 0.000629 | 30 |
| BoLA-2:02603 |  | 0.000629 | 30 |
| BoLA-5:06401 |  | 0.00061 | 17 |
| BoLA-3:02701 |  | 0.000606 | 31 |
| BoLA-3:02702 |  | 0.000606 | 31 |
| BoLA-1:02301 |  | 0.000585 | 33 |
| BoLA-D18.4 |  | 0.000585 | 33 |
| BoLA-1:03102 |  | 0.000576 | 41 |
| BoLA-3:05901 |  | 0.000541 | 37 |
| BoLA-6:01402 |  | 0.000459 | 35 |
| BoLA-2:00602 |  | 0.000454 | 42 |
| BoLA-3:00201 |  | 0.00045 | 46 |
| BoLA-JSP.1 |  | 0.00045 | 46 |
| BoLA-3:00103 |  | 0.00043 | 23 |
| BoLA-1:00902 |  | 0.000417 | 31 |
| BoLA-T5 |  | 0.000417 | 31 |
| BoLA-2:04801 |  | 0.000394 | 29 |
| BoLA-2:05701 |  | 0.00039 | 48 |
| BoLA-3:07301 |  | 0.000377 | 25 |
| BoLA-1:00901 |  | 0.000373 | 34 |
| BoLA-4:02402 |  | 0.000367 | 35 |
| BoLA-2:07101 |  | 0.000326 | 43 |
| BoLA-3:05201 |  | 0.000313 | 36 |
| BoLA-2:05501 |  | 0.00031 | 44 |
| BoLA-3:03801 |  | 0.000308 | 49 |
| BoLA-3:03601 |  | 0.000306 | 49 |
| BoLA-2:06901 |  | 0.000233 | 31 |
| BoLA-1:04901 |  | 0.000207 | 43 |
| BoLA-2:02501 |  | 0.000204 | 38 |
| BoLA-2:01601 |  | 0.000195 | 41 |
| BoLA-2:06001 |  | 0.000159 | 47 |
| BoLA-2:03001 |  | 0.000149 | 34 |
| BoLA-1:02801 |  | 0.000108 | 34 |
| BoLA-2:01801 |  | 9.8e-05 | 48 |
| BoLA-2:01802 |  | 9.8e-05 | 48 |
| BoLA-2:05401 |  | 9.5e-05 | 23 |
| BoLA-1:04201 |  | 9.1e-05 | 57 |
| BoLA-T2c |  | 8.4e-05 | 62 |
| BoLA-1:02901 |  | 7.4e-05 | 47 |
| BoLA-6:01301 |  | 7e-05 | 39 |
| BoLA-HD6 |  | 7e-05 | 39 |
| BoLA-4:02401 |  | 6.3e-05 | 39 |
| BoLA-1:02101 |  | 6.1e-05 | 25 |
| BoLA-1:02001 |  | 4.9e-05 | 60 |
| BoLA-3:01001 |  | 4.1e-05 | 62 |
| BoLA-1:01901 |  | 3.5e-05 | 53 |
| BoLA-6:01501 |  | 3.4e-05 | 50 |
| BoLA-1:07401 |  | 3.2e-05 | 47 |
| BoLA-2:02201 |  | 3.2e-05 | 51 |
| BoLA-6:01302 |  | 3.2e-05 | 36 |
| BoLA-T7 |  | 2.5e-05 | 69 |
| BoLA-4:06301 |  | 2.2e-05 | 50 |
| BoLA-6:03401 |  | 2.2e-05 | 52 |
| BoLA-2:00801 |  | 1.7e-05 | 55 |
| BoLA-6:01502 |  | 1.6e-05 | 52 |
| BoLA-6:04101 |  | 1.3e-05 | 60 |
| BoLA-T2b |  | 1.3e-05 | 60 |
| BoLA-2:00802 |  | 1.1e-05 | 63 |

| Alleles | Peptide | Score | Percentile rank |
| --- | --- | --- | --- |
| BoLA-2:00501 | KPSGGGVPG | 0.089848 | 1.9 |
| BoLA-3:03701 |  | 0.045935 | 8.2 |
| BoLA-2:01801 |  | 0.040972 | 2.8 |
| BoLA-2:01802 |  | 0.040972 | 2.8 |
| BoLA-3:01701 |  | 0.027555 | 3.8 |
| BoLA-1:06701 |  | 0.02582 | 10 |
| BoLA-3:01703 |  | 0.024633 | 8.8 |
| BoLA-2:00601 |  | 0.021989 | 4.4 |
| BoLA-2:01602 |  | 0.021989 | 4.4 |
| BoLA-3:05801 |  | 0.020499 | 11 |
| BoLA-3:01702 |  | 0.018251 | 7.6 |
| BoLA-2:06201 |  | 0.016495 | 9.0 |
| BoLA-2:01201 |  | 0.013513 | 9.1 |
| BoLA-T2a |  | 0.013513 | 9.1 |
| BoLA-2:04401 |  | 0.013471 | 20 |
| BoLA-2:00602 |  | 0.013297 | 8.0 |
| BoLA-2:03001 |  | 0.012601 | 3.6 |
| BoLA-T2c |  | 0.0126 | 18 |
| BoLA-2:04701 |  | 0.012581 | 12 |
| BoLA-3:05002 |  | 0.012417 | 11 |
| BoLA-2:04301 |  | 0.01215 | 7.2 |
| BoLA-3:06501 |  | 0.012062 | 21 |
| BoLA-3:03801 |  | 0.011165 | 11 |
| BoLA-2:04601 |  | 0.01075 | 12 |
| BoLA-3:01001 |  | 0.010746 | 6.3 |
| BoLA-2:06901 |  | 0.010213 | 5.3 |
| BoLA-3:05001 |  | 0.010089 | 14 |
| BoLA-2:05601 |  | 0.009651 | 6.2 |
| BoLA-5:00301 |  | 0.009073 | 26 |
| BoLA-6:01402 |  | 0.007957 | 9.1 |
| BoLA-3:05101 |  | 0.007472 | 9.3 |
| BoLA-4:02402 |  | 0.006878 | 11 |
| BoLA-2:04501 |  | 0.006607 | 16 |
| BoLA-3:06602 |  | 0.006077 | 20 |
| BoLA-4:02401 |  | 0.00545 | 5.3 |
| BoLA-3:06801 |  | 0.005147 | 20 |
| BoLA-2:03202 |  | 0.004616 | 14 |
| BoLA-2:05501 |  | 0.004611 | 15 |
| BoLA-2:07101 |  | 0.004428 | 16 |
| BoLA-1:03102 |  | 0.004412 | 18 |
| BoLA-3:00401 |  | 0.003865 | 14 |
| BoLA-3:00402 |  | 0.003865 | 14 |
| BoLA-3:00403 |  | 0.003865 | 14 |
| BoLA-3:05301 |  | 0.003865 | 14 |
| BoLA-gb1.7 |  | 0.003865 | 14 |
| BoLA-2:05701 |  | 0.003726 | 23 |
| BoLA-1:03101 |  | 0.003662 | 20 |
| BoLA-2:02501 |  | 0.003501 | 11 |
| BoLA-1:06101 |  | 0.003341 | 21 |
| BoLA-1:02001 |  | 0.003281 | 13 |
| BoLA-3:05901 |  | 0.003158 | 17 |
| BoLA-3:03601 |  | 0.003031 | 23 |
| BoLA-2:01601 |  | 0.002899 | 12 |
| BoLA-2:04402 |  | 0.002854 | 18 |
| BoLA-2:06001 |  | 0.002762 | 14 |
| BoLA-3:06601 |  | 0.002575 | 24 |
| BoLA-2:07001 |  | 0.002517 | 14 |
| BoLA-6:01501 |  | 0.002485 | 9.5 |
| BoLA-2:04801 |  | 0.002396 | 15 |
| BoLA-3:00201 |  | 0.002384 | 25 |
| BoLA-JSP.1 |  | 0.002384 | 25 |
| BoLA-5:07201 |  | 0.002314 | 18 |
| BoLA-6:01401 |  | 0.002297 | 17 |
| BoLA-1:04901 |  | 0.002265 | 20 |
| BoLA-3:01101 |  | 0.002263 | 19 |
| BoLA-2:02601 |  | 0.002195 | 18 |
| BoLA-2:02602 |  | 0.002195 | 18 |
| BoLA-2:02603 |  | 0.002195 | 18 |
| BoLA-3:02701 |  | 0.002023 | 18 |
| BoLA-3:02702 |  | 0.002023 | 18 |
| BoLA-1:04201 |  | 0.0018 | 21 |
| BoLA-6:04001 |  | 0.001736 | 25 |
| BoLA-1:00902 |  | 0.001677 | 18 |
| BoLA-T5 |  | 0.001677 | 18 |
| BoLA-1:00901 |  | 0.00164 | 19 |
| BoLA-1:01901 |  | 0.001501 | 12 |
| BoLA-1:02301 |  | 0.001383 | 24 |
| BoLA-D18.4 |  | 0.001383 | 24 |
| BoLA-T7 |  | 0.001163 | 26 |
| BoLA-4:06301 |  | 0.001061 | 16 |
| BoLA-5:03901 |  | 0.000925 | 25 |
| BoLA-3:05201 |  | 0.000899 | 26 |
| BoLA-2:02201 |  | 0.000827 | 16 |
| BoLA-1:07401 |  | 0.000809 | 16 |
| BoLA-amani.1 |  | 0.000786 | 23 |
| BoLA-1:02901 |  | 0.00078 | 23 |
| BoLA-3:07301 |  | 0.000752 | 20 |
| BoLA-3:00103 |  | 0.00058 | 21 |
| BoLA-6:03401 |  | 0.000509 | 18 |
| BoLA-3:00101 |  | 0.000413 | 26 |
| BoLA-AW10 |  | 0.000413 | 26 |
| BoLA-2:00801 |  | 0.000395 | 18 |
| BoLA-3:00102 |  | 0.000356 | 24 |
| BoLA-5:06401 |  | 0.000336 | 21 |
| BoLA-6:04101 |  | 0.000318 | 22 |
| BoLA-T2b |  | 0.000318 | 22 |
| BoLA-3:03501 |  | 0.000257 | 17 |
| BoLA-1:02101 |  | 0.000204 | 14 |
| BoLA-1:02801 |  | 0.00018 | 29 |
| BoLA-2:05401 |  | 0.000115 | 22 |
| BoLA-6:01302 |  | 7.6e-05 | 28 |
| BoLA-6:01502 |  | 7e-05 | 33 |
| BoLA-6:01301 |  | 5.9e-05 | 41 |
| BoLA-HD6 |  | 5.9e-05 | 41 |
| BoLA-2:00802 |  | 3.7e-05 | 44 |

| Alleles | Peptide | Score | Percentile rank |
| --- | --- | --- | --- |
| BoLA-1:02001 | NESGSSSEG | 0.029492 | 3.5 |
| BoLA-6:01402 |  | 0.018286 | 5.9 |
| BoLA-1:01901 |  | 0.007882 | 5.4 |
| BoLA-6:01401 |  | 0.002961 | 15 |
| BoLA-2:00602 |  | 0.001454 | 28 |
| BoLA-1:04201 |  | 0.001045 | 26 |
| BoLA-6:03401 |  | 0.000948 | 14 |
| BoLA-2:01601 |  | 0.000877 | 23 |
| BoLA-2:00601 |  | 0.000849 | 32 |
| BoLA-2:01602 |  | 0.000849 | 32 |
| BoLA-3:03701 |  | 0.00081 | 62 |
| BoLA-2:06001 |  | 0.000766 | 26 |
| BoLA-6:04101 |  | 0.000577 | 17 |
| BoLA-T2b |  | 0.000577 | 17 |
| BoLA-3:05001 |  | 0.000514 | 50 |
| BoLA-1:03102 |  | 0.000491 | 43 |
| BoLA-2:02501 |  | 0.000341 | 31 |
| BoLA-1:06101 |  | 0.000292 | 46 |
| BoLA-1:02901 |  | 0.000274 | 32 |
| BoLA-2:01801 |  | 0.000211 | 37 |
| BoLA-2:01802 |  | 0.000211 | 37 |
| BoLA-1:07401 |  | 0.000198 | 27 |
| BoLA-1:00901 |  | 0.000184 | 43 |
| BoLA-3:05901 |  | 0.000179 | 53 |
| BoLA-1:03101 |  | 0.000173 | 58 |
| BoLA-3:05002 |  | 0.000173 | 57 |
| BoLA-1:02101 |  | 0.00016 | 16 |
| BoLA-2:05501 |  | 0.000143 | 55 |
| BoLA-2:04701 |  | 0.000142 | 64 |
| BoLA-1:00902 |  | 0.000133 | 44 |
| BoLA-T5 |  | 0.000133 | 44 |
| BoLA-1:02301 |  | 0.000127 | 51 |
| BoLA-D18.4 |  | 0.000127 | 51 |
| BoLA-1:06701 |  | 0.00012 | 68 |
| BoLA-2:04601 |  | 0.000113 | 63 |
| BoLA-2:06201 |  | 9.9e-05 | 68 |
| BoLA-3:03801 |  | 8e-05 | 67 |
| BoLA-3:02701 |  | 7.2e-05 | 61 |
| BoLA-3:02702 |  | 7.2e-05 | 61 |
| BoLA-2:04301 |  | 7.1e-05 | 64 |
| BoLA-2:00501 |  | 6.9e-05 | 81 |
| BoLA-3:05801 |  | 6.8e-05 | 79 |
| BoLA-3:01703 |  | 4.6e-05 | 80 |
| BoLA-3:01001 |  | 4.5e-05 | 60 |
| BoLA-3:03601 |  | 4.5e-05 | 73 |
| BoLA-1:04901 |  | 4e-05 | 63 |
| BoLA-6:01502 |  | 3.8e-05 | 40 |
| BoLA-2:04401 |  | 3.7e-05 | 88 |
| BoLA-3:01702 |  | 3.3e-05 | 75 |
| BoLA-3:06501 |  | 3.3e-05 | 87 |
| BoLA-6:01501 |  | 3.3e-05 | 51 |
| BoLA-3:00401 |  | 3e-05 | 72 |
| BoLA-3:00402 |  | 3e-05 | 72 |
| BoLA-3:00403 |  | 3e-05 | 72 |
| BoLA-3:05101 |  | 3e-05 | 71 |
| BoLA-3:05301 |  | 3e-05 | 72 |
| BoLA-5:00301 |  | 3e-05 | 92 |
| BoLA-gb1.7 |  | 3e-05 | 72 |
| BoLA-2:03001 |  | 2.9e-05 | 55 |
| BoLA-T2c |  | 2.9e-05 | 73 |
| BoLA-2:02201 |  | 2.8e-05 | 53 |
| BoLA-2:02601 |  | 2.8e-05 | 70 |
| BoLA-2:02602 |  | 2.8e-05 | 70 |
| BoLA-2:02603 |  | 2.8e-05 | 70 |
| BoLA-3:00201 |  | 2.8e-05 | 86 |
| BoLA-JSP.1 |  | 2.8e-05 | 86 |
| BoLA-2:04801 |  | 2.5e-05 | 65 |
| BoLA-3:01701 |  | 2.4e-05 | 78 |
| BoLA-2:04501 |  | 1.9e-05 | 83 |
| BoLA-5:07201 |  | 1.5e-05 | 73 |
| BoLA-2:01201 |  | 1.4e-05 | 85 |
| BoLA-2:05601 |  | 1.4e-05 | 72 |
| BoLA-T2a |  | 1.4e-05 | 85 |
| BoLA-1:02801 |  | 1.3e-05 | 60 |
| BoLA-2:03202 |  | 1.2e-05 | 82 |
| BoLA-3:07301 |  | 1.2e-05 | 60 |
| BoLA-2:05701 |  | 1.1e-05 | 89 |
| BoLA-2:07101 |  | 1.1e-05 | 85 |
| BoLA-2:04402 |  | 8e-06 | 85 |
| BoLA-3:06601 |  | 8e-06 | 90 |
| BoLA-3:06602 |  | 8e-06 | 92 |
| BoLA-3:05201 |  | 7e-06 | 81 |
| BoLA-3:00103 |  | 6e-06 | 80 |
| BoLA-3:06801 |  | 6e-06 | 90 |
| BoLA-5:03901 |  | 6e-06 | 87 |
| BoLA-6:04001 |  | 6e-06 | 96 |
| BoLA-2:06901 |  | 5e-06 | 78 |
| BoLA-2:07001 |  | 5e-06 | 82 |
| BoLA-3:00101 |  | 5e-06 | 87 |
| BoLA-3:00102 |  | 5e-06 | 83 |
| BoLA-4:02402 |  | 5e-06 | 89 |
| BoLA-AW10 |  | 5e-06 | 87 |
| BoLA-3:01101 |  | 4e-06 | 89 |
| BoLA-2:00802 |  | 3e-06 | 82 |
| BoLA-3:03501 |  | 3e-06 | 69 |
| BoLA-4:02401 |  | 3e-06 | 82 |
| BoLA-amani.1 |  | 3e-06 | 90 |
| BoLA-T7 |  | 3e-06 | 92 |
| BoLA-2:00801 |  | 2e-06 | 86 |
| BoLA-5:06401 |  | 2e-06 | 81 |
| BoLA-6:01301 |  | 2e-06 | 83 |
| BoLA-HD6 |  | 2e-06 | 83 |
| BoLA-2:05401 |  | 1e-06 | 85 |
| BoLA-4:06301 |  | 1e-06 | 89 |
| BoLA-6:01302 |  | 1e-06 | 86 |

| Alleles | Peptide | Score | Percentile rank |
| --- | --- | --- | --- |
| BoLA-3:01703 | SSGLPGSGG | 0.011576 | 15 |
| BoLA-3:06801 |  | 0.008829 | 16 |
| BoLA-2:00501 |  | 0.004284 | 26 |
| BoLA-3:03701 |  | 0.004012 | 39 |
| BoLA-3:01702 |  | 0.003499 | 19 |
| BoLA-3:01701 |  | 0.003461 | 15 |
| BoLA-3:03601 |  | 0.003343 | 22 |
| BoLA-1:06701 |  | 0.003141 | 29 |
| BoLA-3:01101 |  | 0.003037 | 17 |
| BoLA-3:05801 |  | 0.002883 | 30 |
| BoLA-3:06601 |  | 0.002801 | 24 |
| BoLA-2:04701 |  | 0.002744 | 26 |
| BoLA-3:05001 |  | 0.002516 | 28 |
| BoLA-2:04401 |  | 0.002337 | 40 |
| BoLA-3:06602 |  | 0.002172 | 29 |
| BoLA-2:04402 |  | 0.001998 | 21 |
| BoLA-2:04501 |  | 0.001903 | 27 |
| BoLA-2:04601 |  | 0.001413 | 31 |
| BoLA-2:05601 |  | 0.001325 | 20 |
| BoLA-2:07001 |  | 0.001263 | 19 |
| BoLA-3:00401 |  | 0.001169 | 25 |
| BoLA-3:00402 |  | 0.001169 | 25 |
| BoLA-3:00403 |  | 0.001169 | 25 |
| BoLA-3:05301 |  | 0.001169 | 25 |
| BoLA-gb1.7 |  | 0.001169 | 25 |
| BoLA-2:07101 |  | 0.001086 | 29 |
| BoLA-3:06501 |  | 0.001055 | 47 |
| BoLA-2:06201 |  | 0.000982 | 37 |
| BoLA-2:01201 |  | 0.000958 | 29 |
| BoLA-T2a |  | 0.000958 | 29 |
| BoLA-3:05002 |  | 0.000834 | 37 |
| BoLA-5:00301 |  | 0.000787 | 57 |
| BoLA-1:06101 |  | 0.000729 | 36 |
| BoLA-5:03901 |  | 0.000711 | 27 |
| BoLA-2:05701 |  | 0.000675 | 41 |
| BoLA-3:05201 |  | 0.000661 | 29 |
| BoLA-2:00602 |  | 0.000596 | 39 |
| BoLA-2:05401 |  | 0.000595 | 9.7 |
| BoLA-3:05101 |  | 0.000487 | 35 |
| BoLA-3:00102 |  | 0.000476 | 21 |
| BoLA-3:00201 |  | 0.000475 | 45 |
| BoLA-JSP.1 |  | 0.000475 | 45 |
| BoLA-2:03202 |  | 0.000447 | 37 |
| BoLA-2:00601 |  | 0.000438 | 40 |
| BoLA-2:01602 |  | 0.000438 | 40 |
| BoLA-amani.1 |  | 0.00037 | 31 |
| BoLA-3:00101 |  | 0.000343 | 28 |
| BoLA-AW10 |  | 0.000343 | 28 |
| BoLA-1:03102 |  | 0.000336 | 48 |
| BoLA-2:04301 |  | 0.000315 | 43 |
| BoLA-T7 |  | 0.000304 | 39 |
| BoLA-3:05901 |  | 0.000283 | 46 |
| BoLA-2:01601 |  | 0.000276 | 36 |
| BoLA-6:01401 |  | 0.000276 | 40 |
| BoLA-6:04001 |  | 0.000275 | 48 |
| BoLA-1:03101 |  | 0.000255 | 52 |
| BoLA-3:03801 |  | 0.00025 | 52 |
| BoLA-5:07201 |  | 0.000238 | 38 |
| BoLA-5:06401 |  | 0.000217 | 25 |
| BoLA-2:00801 |  | 0.000216 | 23 |
| BoLA-3:00103 |  | 0.000213 | 31 |
| BoLA-3:02701 |  | 0.000209 | 45 |
| BoLA-3:02702 |  | 0.000209 | 45 |
| BoLA-3:07301 |  | 0.0002 | 30 |
| BoLA-T2c |  | 0.000189 | 53 |
| BoLA-2:06901 |  | 0.000185 | 34 |
| BoLA-1:00901 |  | 0.000164 | 45 |
| BoLA-1:02301 |  | 0.000129 | 50 |
| BoLA-D18.4 |  | 0.000129 | 50 |
| BoLA-2:02601 |  | 0.000125 | 49 |
| BoLA-2:02602 |  | 0.000125 | 49 |
| BoLA-2:02603 |  | 0.000125 | 49 |
| BoLA-6:01402 |  | 0.000123 | 54 |
| BoLA-3:03501 |  | 0.00012 | 23 |
| BoLA-2:03001 |  | 0.000111 | 37 |
| BoLA-2:05501 |  | 9.3e-05 | 60 |
| BoLA-1:04201 |  | 9.1e-05 | 57 |
| BoLA-2:04801 |  | 8.6e-05 | 48 |
| BoLA-4:02402 |  | 8.1e-05 | 55 |
| BoLA-2:02501 |  | 7.9e-05 | 51 |
| BoLA-2:01801 |  | 7.6e-05 | 52 |
| BoLA-2:01802 |  | 7.6e-05 | 52 |
| BoLA-1:00902 |  | 6.7e-05 | 52 |
| BoLA-T5 |  | 6.7e-05 | 52 |
| BoLA-2:06001 |  | 6e-05 | 62 |
| BoLA-1:02901 |  | 5.5e-05 | 51 |
| BoLA-3:01001 |  | 5.2e-05 | 58 |
| BoLA-4:06301 |  | 5e-05 | 41 |
| BoLA-1:04901 |  | 4.2e-05 | 63 |
| BoLA-1:02801 |  | 3.4e-05 | 48 |
| BoLA-2:00802 |  | 3.3e-05 | 46 |
| BoLA-1:02001 |  | 3e-05 | 67 |
| BoLA-1:01901 |  | 2.1e-05 | 61 |
| BoLA-6:03401 |  | 1.7e-05 | 55 |
| BoLA-6:01501 |  | 1.6e-05 | 60 |
| BoLA-1:07401 |  | 1.5e-05 | 57 |
| BoLA-4:02401 |  | 1.4e-05 | 60 |
| BoLA-6:04101 |  | 1e-05 | 64 |
| BoLA-T2b |  | 1e-05 | 64 |
| BoLA-6:01502 |  | 9e-06 | 60 |
| BoLA-1:02101 |  | 7e-06 | 53 |
| BoLA-6:01301 |  | 6e-06 | 69 |
| BoLA-HD6 |  | 6e-06 | 69 |
| BoLA-6:01302 |  | 5e-06 | 60 |
| BoLA-2:02201 |  | 3e-06 | 86 |

| Alleles | Peptide | Score | Percentile rank |
| --- | --- | --- | --- |
| BoLA-3:05001 | DSSGLPGSG | 0.029802 | 6.4 |
| BoLA-3:03701 |  | 0.014955 | 21 |
| BoLA-3:05002 |  | 0.013423 | 11 |
| BoLA-3:00401 |  | 0.008139 | 9.0 |
| BoLA-3:00402 |  | 0.008139 | 9.0 |
| BoLA-3:00403 |  | 0.008139 | 9.0 |
| BoLA-3:05301 |  | 0.008139 | 9.0 |
| BoLA-gb1.7 |  | 0.008139 | 9.0 |
| BoLA-3:03601 |  | 0.007604 | 15 |
| BoLA-2:05701 |  | 0.007068 | 17 |
| BoLA-3:05901 |  | 0.003521 | 16 |
| BoLA-2:00501 |  | 0.002726 | 31 |
| BoLA-2:04402 |  | 0.002415 | 19 |
| BoLA-2:04401 |  | 0.00239 | 40 |
| BoLA-3:06801 |  | 0.002282 | 27 |
| BoLA-3:01703 |  | 0.002202 | 31 |
| BoLA-3:02701 |  | 0.00157 | 21 |
| BoLA-3:02702 |  | 0.00157 | 21 |
| BoLA-3:03801 |  | 0.001396 | 31 |
| BoLA-2:00602 |  | 0.001107 | 31 |
| BoLA-1:06701 |  | 0.001065 | 41 |
| BoLA-3:05801 |  | 0.001045 | 43 |
| BoLA-3:01702 |  | 0.000961 | 32 |
| BoLA-3:01701 |  | 0.000887 | 28 |
| BoLA-2:01801 |  | 0.000743 | 23 |
| BoLA-2:01802 |  | 0.000743 | 23 |
| BoLA-3:01101 |  | 0.00053 | 31 |
| BoLA-T7 |  | 0.000483 | 35 |
| BoLA-2:07101 |  | 0.000415 | 40 |
| BoLA-2:04301 |  | 0.000405 | 40 |
| BoLA-2:06201 |  | 0.000388 | 49 |
| BoLA-2:00601 |  | 0.000383 | 42 |
| BoLA-2:01602 |  | 0.000383 | 42 |
| BoLA-2:04501 |  | 0.000373 | 47 |
| BoLA-2:04601 |  | 0.000367 | 47 |
| BoLA-T2c |  | 0.000338 | 48 |
| BoLA-2:07001 |  | 0.00033 | 31 |
| BoLA-2:04701 |  | 0.000329 | 52 |
| BoLA-2:03202 |  | 0.000318 | 41 |
| BoLA-5:00301 |  | 0.000314 | 69 |
| BoLA-2:01601 |  | 0.000283 | 36 |
| BoLA-3:06501 |  | 0.000282 | 63 |
| BoLA-3:06601 |  | 0.00025 | 51 |
| BoLA-2:04801 |  | 0.000248 | 35 |
| BoLA-3:01001 |  | 0.000245 | 37 |
| BoLA-amani.1 |  | 0.000237 | 36 |
| BoLA-2:05601 |  | 0.000226 | 38 |
| BoLA-3:06602 |  | 0.000226 | 57 |
| BoLA-3:05201 |  | 0.00021 | 41 |
| BoLA-2:02601 |  | 0.000187 | 44 |
| BoLA-2:02602 |  | 0.000187 | 44 |
| BoLA-2:02603 |  | 0.000187 | 44 |
| BoLA-2:06001 |  | 0.000182 | 45 |
| BoLA-3:05101 |  | 0.000177 | 48 |
| BoLA-3:00201 |  | 0.000173 | 61 |
| BoLA-JSP.1 |  | 0.000173 | 61 |
| BoLA-1:06101 |  | 0.000165 | 53 |
| BoLA-2:06901 |  | 0.000142 | 37 |
| BoLA-6:01402 |  | 0.000139 | 52 |
| BoLA-2:02501 |  | 0.00013 | 44 |
| BoLA-2:01201 |  | 0.000128 | 56 |
| BoLA-T2a |  | 0.000128 | 56 |
| BoLA-4:02402 |  | 0.00011 | 50 |
| BoLA-2:00802 |  | 0.000107 | 31 |
| BoLA-4:06301 |  | 9.4e-05 | 34 |
| BoLA-3:00101 |  | 8.7e-05 | 46 |
| BoLA-AW10 |  | 8.7e-05 | 46 |
| BoLA-2:03001 |  | 8.5e-05 | 41 |
| BoLA-2:00801 |  | 7.9e-05 | 34 |
| BoLA-3:00103 |  | 7.1e-05 | 44 |
| BoLA-1:02001 |  | 6.9e-05 | 55 |
| BoLA-5:03901 |  | 6.6e-05 | 57 |
| BoLA-1:04201 |  | 6.5e-05 | 61 |
| BoLA-3:00102 |  | 6.5e-05 | 45 |
| BoLA-1:02301 |  | 6.4e-05 | 59 |
| BoLA-D18.4 |  | 6.4e-05 | 59 |
| BoLA-1:04901 |  | 5.8e-05 | 59 |
| BoLA-6:01401 |  | 5.6e-05 | 61 |
| BoLA-2:05501 |  | 5.5e-05 | 67 |
| BoLA-1:02901 |  | 5.4e-05 | 51 |
| BoLA-1:03101 |  | 5.4e-05 | 74 |
| BoLA-5:07201 |  | 4.6e-05 | 59 |
| BoLA-2:05401 |  | 3.8e-05 | 33 |
| BoLA-1:00902 |  | 3e-05 | 63 |
| BoLA-T5 |  | 3e-05 | 63 |
| BoLA-1:03102 |  | 2.9e-05 | 82 |
| BoLA-1:01901 |  | 2.6e-05 | 58 |
| BoLA-1:00901 |  | 2.5e-05 | 70 |
| BoLA-6:04001 |  | 2.5e-05 | 85 |
| BoLA-3:07301 |  | 2.3e-05 | 52 |
| BoLA-4:02401 |  | 2.1e-05 | 54 |
| BoLA-3:03501 |  | 1.8e-05 | 44 |
| BoLA-6:01501 |  | 1.2e-05 | 64 |
| BoLA-1:07401 |  | 1.1e-05 | 61 |
| BoLA-6:04101 |  | 1.1e-05 | 62 |
| BoLA-T2b |  | 1.1e-05 | 62 |
| BoLA-6:03401 |  | 1e-05 | 63 |
| BoLA-5:06401 |  | 7e-06 | 64 |
| BoLA-1:02801 |  | 6e-06 | 70 |
| BoLA-2:02201 |  | 5e-06 | 79 |
| BoLA-1:02101 |  | 4e-06 | 62 |
| BoLA-6:01301 |  | 4e-06 | 74 |
| BoLA-HD6 |  | 4e-06 | 74 |
| BoLA-6:01502 |  | 3e-06 | 75 |
| BoLA-6:01302 |  | 2e-06 | 74 |
