# Supplementary material for: Development of a multi-epitope chimeric vaccine in silico against Babesia bovis, Theileria annulata, and Anaplasma marginale using computational biology tools and reverse vaccinology approach
Source: PLoS One. 2025 Jan 24;20(1):e0312262. doi: 10.1371/journal.pone.0312262 (PMC11759392; doi:10.1371/journal.pone.0312262)
Supplement: S5 File — (DOCX) [file pone.0312262.s011.docx]

The tables of all the ten MHC I epitopes of TASP with their scores and percentile rank representing their affinities for different BOLA alleles. The peptide which has been selected for chimeric vaccine construction has been represented in bold letters. The BoLA alleles binding with the selected peptide possessing a percentile value >50 is highlighted as yellow. The BoLA allele that has bonded with the selected peptide with the lowest percentile rank is highlighted as green.

| Alleles | Peptide | Score | Percentile rank |
| --- | --- | --- | --- |
| BoLA-3:03701 | **TKASSSGDG** | 0.000782 | 62 |
| BoLA-3:05901 |  | 0.000255 | 47 |
| BoLA-2:02601 |  | 0.000237 | 41 |
| BoLA-2:02602 |  | 0.000237 | 41 |
| BoLA-2:02603 |  | 0.000237 | 41 |
| BoLA-3:01001 |  | 0.000219 | 39 |
| BoLA-1:02301 |  | 0.000217 | 44 |
| BoLA-D18.4 |  | 0.000217 | 44 |
| BoLA-3:02701 |  | 0.000208 | 45 |
| BoLA-3:02702 |  | 0.000208 | 45 |
| BoLA-3:01703 |  | 0.000192 | 62 |
| BoLA-3:05002 |  | 0.000183 | 57 |
| BoLA-3:05001 |  | 0.000156 | 67 |
| BoLA-2:06201 |  | 0.000138 | 64 |
| BoLA-1:04901 |  | 0.000132 | 48 |
| BoLA-3:00401 |  | 0.000127 | 53 |
| BoLA-3:00402 |  | 0.000127 | 53 |
| BoLA-3:00403 |  | 0.000127 | 53 |
| BoLA-3:05301 |  | 0.000127 | 53 |
| BoLA-gb1.7 |  | 0.000127 | 53 |
| BoLA-3:05801 |  | 0.00011 | 74 |
| BoLA-6:01402 |  | 8.5e-05 | 60 |
| BoLA-2:04301 |  | 8.4e-05 | 62 |
| BoLA-1:00902 |  | 8e-05 | 50 |
| BoLA-T5 |  | 8e-05 | 50 |
| BoLA-2:04801 |  | 7.9e-05 | 49 |
| BoLA-3:06501 |  | 7.3e-05 | 79 |
| BoLA-1:03102 |  | 6.7e-05 | 71 |
| BoLA-1:04201 |  | 6.2e-05 | 62 |
| BoLA-2:00601 |  | 5.3e-05 | 69 |
| BoLA-2:01602 |  | 5.3e-05 | 69 |
| BoLA-3:01702 |  | 5.1e-05 | 70 |
| BoLA-2:02501 |  | 4.9e-05 | 59 |
| BoLA-1:02001 |  | 4.8e-05 | 60 |
| BoLA-1:03101 |  | 4.8e-05 | 75 |
| BoLA-2:00602 |  | 4.4e-05 | 75 |
| BoLA-2:04701 |  | 4.4e-05 | 78 |
| BoLA-2:05501 |  | 4.3e-05 | 71 |
| BoLA-2:01601 |  | 4e-05 | 64 |
| BoLA-3:01701 |  | 3.6e-05 | 73 |
| BoLA-2:04401 |  | 3.5e-05 | 89 |
| BoLA-1:06101 |  | 3.1e-05 | 73 |
| BoLA-6:04001 |  | 2.9e-05 | 83 |
| BoLA-2:03202 |  | 2.7e-05 | 73 |
| BoLA-2:06001 |  | 2.7e-05 | 73 |
| BoLA-3:03801 |  | 2.7e-05 | 80 |
| BoLA-5:00301 |  | 2.5e-05 | 93 |
| BoLA-1:00901 |  | 2.4e-05 | 71 |
| BoLA-1:01901 |  | 2.4e-05 | 59 |
| BoLA-2:05701 |  | 2.2e-05 | 83 |
| BoLA-2:07101 |  | 2.2e-05 | 78 |
| BoLA-3:06601 |  | 2e-05 | 82 |
| BoLA-6:01401 |  | 2e-05 | 75 |
| BoLA-2:01801 |  | 1.9e-05 | 72 |
| BoLA-2:01802 |  | 1.9e-05 | 72 |
| BoLA-2:02201 |  | 1.9e-05 | 59 |
| BoLA-2:04601 |  | 1.8e-05 | 84 |
| BoLA-2:00501 |  | 1.6e-05 | 93 |
| BoLA-3:06602 |  | 1.6e-05 | 88 |
| BoLA-3:01101 |  | 1.5e-05 | 75 |
| BoLA-2:04501 |  | 1.4e-05 | 86 |
| BoLA-1:02901 |  | 1.3e-05 | 70 |
| BoLA-4:02402 |  | 1.1e-05 | 81 |
| BoLA-6:03401 |  | 1.1e-05 | 61 |
| BoLA-1:06701 |  | 9e-06 | 92 |
| BoLA-2:06901 |  | 9e-06 | 71 |
| BoLA-2:01201 |  | 8e-06 | 90 |
| BoLA-3:06801 |  | 8e-06 | 88 |
| BoLA-T2a |  | 8e-06 | 90 |
| BoLA-2:05601 |  | 7e-06 | 79 |
| BoLA-3:00201 |  | 7e-06 | 97 |
| BoLA-JSP.1 |  | 7e-06 | 97 |
| BoLA-3:05101 |  | 5e-06 | 89 |
| BoLA-1:02101 |  | 4e-06 | 62 |
| BoLA-3:00101 |  | 4e-06 | 89 |
| BoLA-3:00103 |  | 4e-06 | 85 |
| BoLA-3:03601 |  | 4e-06 | 94 |
| BoLA-4:02401 |  | 4e-06 | 78 |
| BoLA-6:01502 |  | 4e-06 | 71 |
| BoLA-AW10 |  | 4e-06 | 89 |
| BoLA-T2c |  | 4e-06 | 90 |
| BoLA-2:03001 |  | 3e-06 | 84 |
| BoLA-2:04402 |  | 3e-06 | 93 |
| BoLA-3:00102 |  | 3e-06 | 88 |
| BoLA-3:05201 |  | 3e-06 | 89 |
| BoLA-5:03901 |  | 3e-06 | 93 |
| BoLA-6:01501 |  | 3e-06 | 82 |
| BoLA-amani.1 |  | 3e-06 | 90 |
| BoLA-1:02801 |  | 2e-06 | 83 |
| BoLA-1:07401 |  | 2e-06 | 83 |
| BoLA-3:03501 |  | 2e-06 | 75 |
| BoLA-5:07201 |  | 2e-06 | 94 |
| BoLA-6:01301 |  | 2e-06 | 83 |
| BoLA-HD6 |  | 2e-06 | 83 |
| BoLA-2:00801 |  | 1e-06 | 94 |
| BoLA-2:00802 |  | 1e-06 | 95 |
| BoLA-2:05401 |  | 1e-06 | 85 |
| BoLA-2:07001 |  | 1e-06 | 97 |
| BoLA-3:07301 |  | 1e-06 | 90 |
| BoLA-6:01302 |  | 1e-06 | 86 |
| BoLA-6:04101 |  | 1e-06 | 95 |
| BoLA-T2b |  | 1e-06 | 95 |
| BoLA-T7 |  | 1e-06 | 98 |
| BoLA-4:06301 |  | 0.0 | 100 |
| BoLA-5:06401 |  | 0.0 | 100 |

| Alleles | Peptide | Score | Percentile rank |
| --- | --- | --- | --- |
| BoLA-3:01703 | ASSSGDGAA | 0.044067 | 5.6 |
| BoLA-3:01702 |  | 0.02577 | 6.0 |
| BoLA-1:06101 |  | 0.022939 | 6.8 |
| BoLA-1:06701 |  | 0.020675 | 12 |
| BoLA-3:03701 |  | 0.015257 | 21 |
| BoLA-5:00301 |  | 0.012108 | 22 |
| BoLA-3:01701 |  | 0.008985 | 8.9 |
| BoLA-2:07001 |  | 0.007211 | 7.6 |
| BoLA-3:05001 |  | 0.007139 | 17 |
| BoLA-2:04401 |  | 0.006513 | 28 |
| BoLA-3:06602 |  | 0.006089 | 20 |
| BoLA-3:06601 |  | 0.005936 | 17 |
| BoLA-2:05701 |  | 0.005338 | 19 |
| BoLA-3:03601 |  | 0.004517 | 20 |
| BoLA-3:01101 |  | 0.004282 | 15 |
| BoLA-3:06801 |  | 0.004121 | 22 |
| BoLA-3:05801 |  | 0.003941 | 27 |
| BoLA-3:05002 |  | 0.003807 | 21 |
| BoLA-2:00501 |  | 0.003654 | 28 |
| BoLA-3:06501 |  | 0.003594 | 33 |
| BoLA-6:01402 |  | 0.003566 | 14 |
| BoLA-6:01501 |  | 0.002825 | 8.8 |
| BoLA-5:07201 |  | 0.002788 | 17 |
| BoLA-6:01401 |  | 0.0027 | 16 |
| BoLA-2:05601 |  | 0.002329 | 15 |
| BoLA-T7 |  | 0.002258 | 21 |
| BoLA-6:01502 |  | 0.002254 | 7. |
| BoLA-2:04701 |  | 0.001886 | 30 |
| BoLA-3:05201 |  | 0.001824 | 20 |
| BoLA-2:04501 |  | 0.001614 | 29 |
| BoLA-2:06201 |  | 0.001552 | 31 |
| BoLA-3:00401 |  | 0.001453 | 23 |
| BoLA-3:00402 |  | 0.001453 | 23 |
| BoLA-3:00403 |  | 0.001453 | 23 |
| BoLA-3:05301 |  | 0.001453 | 23 |
| BoLA-gb1.7 |  | 0.001453 | 23 |
| BoLA-T2c |  | 0.001291 | 35 |
| BoLA-3:07301 |  | 0.001141 | 17 |
| BoLA-1:00902 |  | 0.001089 | 21 |
| BoLA-T5 |  | 0.001089 | 21 |
| BoLA-1:00901 |  | 0.001087 | 23 |
| BoLA-2:01201 |  | 0.000995 | 29 |
| BoLA-T2a |  | 0.000995 | 29 |
| BoLA-2:04402 |  | 0.000928 | 29 |
| BoLA-1:02301 |  | 0.000875 | 29 |
| BoLA-D18.4 |  | 0.000875 | 29 |
| BoLA-2:07101 |  | 0.000866 | 32 |
| BoLA-2:04601 |  | 0.000818 | 37 |
| BoLA-2:03202 |  | 0.000813 | 30 |
| BoLA-2:00601 |  | 0.00075 | 33 |
| BoLA-2:01602 |  | 0.00075 | 33 |
| BoLA-3:00201 |  | 0.000722 | 39 |
| BoLA-JSP.1 |  | 0.000722 | 39 |
| BoLA-amani.1 |  | 0.000684 | 25 |
| BoLA-2:06901 |  | 0.000648 | 21 |
| BoLA-1:01901 |  | 0.000643 | 18 |
| BoLA-5:03901 |  | 0.000604 | 29 |
| BoLA-1:02001 |  | 0.000587 | 27 |
| BoLA-3:00101 |  | 0.000574 | 23 |
| BoLA-AW10 |  | 0.000574 | 23 |
| BoLA-1:02901 |  | 0.000561 | 26 |
| BoLA-3:05901 |  | 0.000492 | 38 |
| BoLA-1:03101 |  | 0.00048 | 44 |
| BoLA-2:02601 |  | 0.000453 | 33 |
| BoLA-2:02602 |  | 0.000453 | 33 |
| BoLA-2:02603 |  | 0.000453 | 33 |
| BoLA-2:05501 |  | 0.000432 | 40 |
| BoLA-2:00602 |  | 0.000431 | 43 |
| BoLA-6:04001 |  | 0.000425 | 42 |
| BoLA-3:01001 |  | 0.0004 | 32 |
| BoLA-3:05101 |  | 0.000374 | 38 |
| BoLA-1:04201 |  | 0.000362 | 38 |
| BoLA-3:00103 |  | 0.000355 | 25 |
| BoLA-3:03801 |  | 0.000353 | 47 |
| BoLA-2:06001 |  | 0.000336 | 36 |
| BoLA-2:03001 |  | 0.000313 | 26 |
| BoLA-2:05401 |  | 0.000312 | 14 |
| BoLA-2:01801 |  | 0.0003 | 33 |
| BoLA-2:01802 |  | 0.0003 | 33 |
| BoLA-3:02701 |  | 0.000298 | 40 |
| BoLA-3:02702 |  | 0.000298 | 40 |
| BoLA-2:04801 |  | 0.000295 | 33 |
| BoLA-1:02801 |  | 0.000292 | 25 |
| BoLA-5:06401 |  | 0.000289 | 22 |
| BoLA-2:04301 |  | 0.000282 | 45 |
| BoLA-1:03102 |  | 0.00028 | 51 |
| BoLA-2:02501 |  | 0.000272 | 34 |
| BoLA-1:04901 |  | 0.000269 | 40 |
| BoLA-1:02101 |  | 0.000265 | 12 |
| BoLA-3:00102 |  | 0.000259 | 27 |
| BoLA-2:01601 |  | 0.000254 | 37 |
| BoLA-4:06301 |  | 0.000216 | 27 |
| BoLA-3:03501 |  | 0.000183 | 19 |
| BoLA-2:00801 |  | 0.000156 | 26 |
| BoLA-6:03401 |  | 0.000132 | 30 |
| BoLA-2:00802 |  | 0.000112 | 30 |
| BoLA-4:02402 |  | 0.000107 | 51 |
| BoLA-1:07401 |  | 9.4e-05 | 34 |
| BoLA-6:01301 |  | 6.8e-05 | 40 |
| BoLA-HD6 |  | 6.8e-05 | 40 |
| BoLA-6:04101 |  | 3e-05 | 48 |
| BoLA-T2b |  | 3e-05 | 48 |
| BoLA-2:02201 |  | 2.1e-05 | 57 |
| BoLA-4:02401 |  | 1.6e-05 | 58 |
| BoLA-6:01302 |  | 1.5e-05 | 45 |

| Alleles | Peptide | Score | Percentile rank |
| --- | --- | --- | --- |
| BoLA-1:00901 | SGDGAAPCH | 0.128672 | 1.3 |
| BoLA-3:03701 |  | 0.056765 | 6.6 |
| BoLA-2:04601 |  | 0.043178 | 3.4 |
| BoLA-3:05101 |  | 0.034257 | 2.7 |
| BoLA-2:04401 |  | 0.033476 | 11 |
| BoLA-1:00902 |  | 0.031817 | 3.3 |
| BoLA-T5 |  | 0.031817 | 3.3 |
| BoLA-3:05801 |  | 0.031786 | 7.4 |
| BoLA-2:04301 |  | 0.029842 | 3.4 |
| BoLA-2:06201 |  | 0.028287 | 6.1 |
| BoLA-3:01703 |  | 0.027532 | 8.1 |
| BoLA-2:07001 |  | 0.026287 | 2.7 |
| BoLA-2:04402 |  | 0.026118 | 4.2 |
| BoLA-2:04501 |  | 0.020302 | 7.6 |
| BoLA-3:01702 |  | 0.016363 | 8.3 |
| BoLA-3:01701 |  | 0.013084 | 6.9 |
| BoLA-2:07101 |  | 0.013083 | 8.1 |
| BoLA-2:02501 |  | 0.012818 | 4.6 |
| BoLA-1:06701 |  | 0.011832 | 16 |
| BoLA-3:06801 |  | 0.011301 | 14 |
| BoLA-2:00601 |  | 0.011259 | 8.0 |
| BoLA-2:01602 |  | 0.011259 | 8.0 |
| BoLA-2:04701 |  | 0.010924 | 13 |
| BoLA-2:01201 |  | 0.010257 | 11 |
| BoLA-T2a |  | 0.010257 | 11 |
| BoLA-3:03601 |  | 0.010243 | 13 |
| BoLA-3:05201 |  | 0.009292 | 9.5 |
| BoLA-2:05501 |  | 0.008568 | 11 |
| BoLA-1:02301 |  | 0.007662 | 12 |
| BoLA-D18.4 |  | 0.007662 | 12 |
| BoLA-6:01402 |  | 0.007333 | 9.4 |
| BoLA-1:03101 |  | 0.006932 | 15 |
| BoLA-2:03202 |  | 0.006825 | 11 |
| BoLA-2:06001 |  | 0.006799 | 8.0 |
| BoLA-2:00602 |  | 0.006753 | 13 |
| BoLA-1:02001 |  | 0.005852 | 9.5 |
| BoLA-2:05601 |  | 0.00552 | 9.2 |
| BoLA-2:03001 |  | 0.004663 | 7.4 |
| BoLA-1:06101 |  | 0.004637 | 18 |
| BoLA-1:03102 |  | 0.003874 | 19 |
| BoLA-2:06901 |  | 0.003746 | 9.6 |
| BoLA-2:01601 |  | 0.003369 | 11 |
| BoLA-3:06501 |  | 0.003273 | 34 |
| BoLA-2:00501 |  | 0.00271 | 32 |
| BoLA-2:04801 |  | 0.002669 | 14 |
| BoLA-1:04201 |  | 0.00258 | 18 |
| BoLA-3:03801 |  | 0.002332 | 25 |
| BoLA-5:07201 |  | 0.002195 | 19 |
| BoLA-T2c |  | 0.002132 | 31 |
| BoLA-3:05002 |  | 0.002011 | 27 |
| BoLA-3:06602 |  | 0.001922 | 30 |
| BoLA-3:00101 |  | 0.001784 | 13 |
| BoLA-AW10 |  | 0.001784 | 13 |
| BoLA-4:02402 |  | 0.001768 | 20 |
| BoLA-2:02201 |  | 0.001378 | 13 |
| BoLA-4:06301 |  | 0.001327 | 15 |
| BoLA-5:00301 |  | 0.001324 | 50 |
| BoLA-5:06401 |  | 0.001183 | 13 |
| BoLA-3:06601 |  | 0.001166 | 32 |
| BoLA-5:03901 |  | 0.001118 | 23 |
| BoLA-3:00401 |  | 0.001078 | 26 |
| BoLA-3:00402 |  | 0.001078 | 26 |
| BoLA-3:00403 |  | 0.001078 | 26 |
| BoLA-3:05301 |  | 0.001078 | 26 |
| BoLA-gb1.7 |  | 0.001078 | 26 |
| BoLA-6:04001 |  | 0.00103 | 31 |
| BoLA-3:01101 |  | 0.001002 | 25 |
| BoLA-6:03401 |  | 0.000999 | 14 |
| BoLA-3:02701 |  | 0.000881 | 26 |
| BoLA-3:02702 |  | 0.000881 | 26 |
| BoLA-2:05701 |  | 0.000735 | 40 |
| BoLA-3:05901 |  | 0.000734 | 33 |
| BoLA-2:00802 |  | 0.000707 | 14 |
| BoLA-3:05001 |  | 0.000671 | 46 |
| BoLA-1:07401 |  | 0.000646 | 17 |
| BoLA-3:00201 |  | 0.000641 | 41 |
| BoLA-JSP.1 |  | 0.000641 | 41 |
| BoLA-4:02401 |  | 0.000591 | 16 |
| BoLA-3:00103 |  | 0.00052 | 22 |
| BoLA-1:02901 |  | 0.000507 | 27 |
| BoLA-3:00102 |  | 0.00049 | 21 |
| BoLA-3:01001 |  | 0.000369 | 33 |
| BoLA-2:05401 |  | 0.000359 | 13 |
| BoLA-2:01801 |  | 0.000355 | 31 |
| BoLA-2:01802 |  | 0.000355 | 31 |
| BoLA-1:02801 |  | 0.000336 | 24 |
| BoLA-amani.1 |  | 0.000332 | 32 |
| BoLA-3:07301 |  | 0.000322 | 26 |
| BoLA-T7 |  | 0.000303 | 39 |
| BoLA-6:01401 |  | 0.000286 | 39 |
| BoLA-6:01501 |  | 0.000245 | 27 |
| BoLA-2:00801 |  | 0.000224 | 23 |
| BoLA-1:04901 |  | 0.000177 | 44 |
| BoLA-2:02601 |  | 0.000165 | 46 |
| BoLA-2:02602 |  | 0.000165 | 46 |
| BoLA-2:02603 |  | 0.000165 | 46 |
| BoLA-6:01502 |  | 0.000151 | 25 |
| BoLA-3:03501 |  | 0.000136 | 22 |
| BoLA-1:01901 |  | 6.8e-05 | 43 |
| BoLA-1:02101 |  | 4.1e-05 | 29 |
| BoLA-6:04101 |  | 2e-05 | 54 |
| BoLA-T2b |  | 2e-05 | 54 |
| BoLA-6:01301 |  | 1.2e-05 | 60 |
| BoLA-HD6 |  | 1.2e-05 | 60 |
| BoLA-6:01302 |  | 1.1e-05 | 49 |

| Alleles | Peptide | Score | Percentile rank |
| --- | --- | --- | --- |
| BoLA-2:04401 | PTKASSSGD | 0.000151 | 75 |
| BoLA-3:03701 |  | 9e-05 | 86 |
| BoLA-3:05001 |  | 6.1e-05 | 79 |
| BoLA-2:04501 |  | 4.2e-05 | 74 |
| BoLA-3:03601 |  | 4.1e-05 | 74 |
| BoLA-3:01703 |  | 3.2e-05 | 83 |
| BoLA-3:06602 |  | 2.1e-05 | 85 |
| BoLA-2:03202 |  | 1.9e-05 | 77 |
| BoLA-2:04701 |  | 1.8e-05 | 87 |
| BoLA-2:06201 |  | 1.7e-05 | 88 |
| BoLA-2:07101 |  | 1.7e-05 | 81 |
| BoLA-1:06701 |  | 1.6e-05 | 88 |
| BoLA-2:05701 |  | 1.6e-05 | 86 |
| BoLA-amani.1 |  | 1.5e-05 | 72 |
| BoLA-3:01701 |  | 1.4e-05 | 84 |
| BoLA-3:05002 |  | 1.4e-05 | 86 |
| BoLA-3:06601 |  | 1.4e-05 | 85 |
| BoLA-5:00301 |  | 1.3e-05 | 96 |
| BoLA-1:03102 |  | 1.2e-05 | 90 |
| BoLA-2:04402 |  | 1.2e-05 | 81 |
| BoLA-2:04601 |  | 1.2e-05 | 87 |
| BoLA-3:05901 |  | 1.2e-05 | 89 |
| BoLA-3:01702 |  | 1.1e-05 | 87 |
| BoLA-3:00401 |  | 1e-05 | 85 |
| BoLA-3:00402 |  | 1e-05 | 85 |
| BoLA-3:00403 |  | 1e-05 | 85 |
| BoLA-3:05301 |  | 1e-05 | 85 |
| BoLA-gb1.7 |  | 1e-05 | 85 |
| BoLA-1:03101 |  | 9e-06 | 91 |
| BoLA-2:00501 |  | 9e-06 | 95 |
| BoLA-T7 |  | 9e-06 | 81 |
| BoLA-3:06801 |  | 8e-06 | 88 |
| BoLA-2:01201 |  | 7e-06 | 91 |
| BoLA-3:05801 |  | 7e-06 | 96 |
| BoLA-6:04001 |  | 7e-06 | 96 |
| BoLA-T2a |  | 7e-06 | 91 |
| BoLA-2:00801 |  | 6e-06 | 70 |
| BoLA-2:02501 |  | 6e-06 | 87 |
| BoLA-3:02701 |  | 6e-06 | 92 |
| BoLA-3:02702 |  | 6e-06 | 92 |
| BoLA-1:06101 |  | 5e-06 | 90 |
| BoLA-2:04301 |  | 5e-06 | 93 |
| BoLA-2:05601 |  | 5e-06 | 83 |
| BoLA-3:00201 |  | 5e-06 | 98 |
| BoLA-3:01101 |  | 5e-06 | 87 |
| BoLA-3:06501 |  | 5e-06 | 98 |
| BoLA-4:02402 |  | 5e-06 | 89 |
| BoLA-JSP.1 |  | 5e-06 | 98 |
| BoLA-2:02601 |  | 4e-06 | 92 |
| BoLA-2:02602 |  | 4e-06 | 92 |
| BoLA-2:02603 |  | 4e-06 | 92 |
| BoLA-2:05501 |  | 4e-06 | 94 |
| BoLA-3:05101 |  | 4e-06 | 91 |
| BoLA-2:00802 |  | 3e-06 | 82 |
| BoLA-2:01601 |  | 3e-06 | 94 |
| BoLA-2:01801 |  | 3e-06 | 92 |
| BoLA-2:01802 |  | 3e-06 | 92 |
| BoLA-2:06001 |  | 3e-06 | 97 |
| BoLA-3:00101 |  | 3e-06 | 92 |
| BoLA-3:03801 |  | 3e-06 | 97 |
| BoLA-5:03901 |  | 3e-06 | 93 |
| BoLA-6:01402 |  | 3e-06 | 96 |
| BoLA-AW10 |  | 3e-06 | 92 |
| BoLA-1:00901 |  | 2e-06 | 95 |
| BoLA-1:02301 |  | 2e-06 | 95 |
| BoLA-2:03001 |  | 2e-06 | 89 |
| BoLA-2:07001 |  | 2e-06 | 91 |
| BoLA-3:01001 |  | 2e-06 | 95 |
| BoLA-3:05201 |  | 2e-06 | 92 |
| BoLA-4:02401 |  | 2e-06 | 87 |
| BoLA-6:01401 |  | 2e-06 | 96 |
| BoLA-D18.4 |  | 2e-06 | 95 |
| BoLA-T2c |  | 2e-06 | 95 |
| BoLA-1:00902 |  | 1e-06 | 97 |
| BoLA-1:02001 |  | 1e-06 | 99 |
| BoLA-1:02901 |  | 1e-06 | 97 |
| BoLA-1:04201 |  | 1e-06 | 99 |
| BoLA-1:04901 |  | 1e-06 | 99 |
| BoLA-2:00601 |  | 1e-06 | 100 |
| BoLA-2:00602 |  | 1e-06 | 100 |
| BoLA-2:01602 |  | 1e-06 | 100 |
| BoLA-2:05401 |  | 1e-06 | 85 |
| BoLA-3:00102 |  | 1e-06 | 98 |
| BoLA-3:00103 |  | 1e-06 | 97 |
| BoLA-5:07201 |  | 1e-06 | 98 |
| BoLA-6:04101 |  | 1e-06 | 95 |
| BoLA-T2b |  | 1e-06 | 95 |
| BoLA-T5 |  | 1e-06 | 97 |
| BoLA-1:01901 |  | 0.0 | 100 |
| BoLA-1:02101 |  | 0.0 | 100 |
| BoLA-1:02801 |  | 0.0 | 100 |
| BoLA-1:07401 |  | 0.0 | 100 |
| BoLA-2:02201 |  | 0.0 | 100 |
| BoLA-2:04801 |  | 0.0 | 100 |
| BoLA-2:06901 |  | 0.0 | 100 |
| BoLA-3:03501 |  | 0.0 | 100 |
| BoLA-3:07301 |  | 0.0 | 100 |
| BoLA-4:06301 |  | 0.0 | 100 |
| BoLA-5:06401 |  | 0.0 | 100 |
| BoLA-6:01301 |  | 0.0 | 100 |
| BoLA-6:01302 |  | 0.0 | 100 |
| BoLA-6:01501 |  | 0.0 | 100 |
| BoLA-6:01502 |  | 0.0 | 100 |
| BoLA-6:03401 |  | 0.0 | 100 |
| BoLA-HD6 |  | 0.0 | 100 |

| Alleles | Peptide | Score | Percentile rank |
| --- | --- | --- | --- |
| BoLA-3:01703 | SSSGDGAAP | 0.056295 | 4.5 |
| BoLA-3:01702 |  | 0.03755 | 4.4 |
| BoLA-1:06101 |  | 0.028994 | 5.6 |
| BoLA-1:06701 |  | 0.020221 | 12 |
| BoLA-3:01701 |  | 0.012261 | 7.2 |
| BoLA-6:01401 |  | 0.011885 | 6.4 |
| BoLA-6:01402 |  | 0.010781 | 7.8 |
| BoLA-2:04401 |  | 0.010764 | 22 |
| BoLA-2:00501 |  | 0.009532 | 17 |
| BoLA-3:03701 |  | 0.009354 | 27 |
| BoLA-3:01101 |  | 0.009219 | 9.8 |
| BoLA-3:03601 |  | 0.008764 | 14 |
| BoLA-3:05001 |  | 0.007564 | 17 |
| BoLA-3:05002 |  | 0.007075 | 16 |
| BoLA-5:00301 |  | 0.006941 | 29 |
| BoLA-2:04402 |  | 0.006793 | 11 |
| BoLA-2:07001 |  | 0.006443 | 8.2 |
| BoLA-3:06602 |  | 0.005922 | 20 |
| BoLA-3:06801 |  | 0.005495 | 20 |
| BoLA-5:07201 |  | 0.004596 | 14 |
| BoLA-2:04501 |  | 0.00447 | 19 |
| BoLA-3:06601 |  | 0.004403 | 20 |
| BoLA-2:06201 |  | 0.004369 | 20 |
| BoLA-T2c |  | 0.003922 | 26 |
| BoLA-3:05801 |  | 0.003462 | 28 |
| BoLA-2:04701 |  | 0.00313 | 24 |
| BoLA-2:05701 |  | 0.002729 | 26 |
| BoLA-2:05601 |  | 0.002394 | 15 |
| BoLA-3:05101 |  | 0.002327 | 18 |
| BoLA-3:05901 |  | 0.00212 | 21 |
| BoLA-2:04301 |  | 0.002115 | 21 |
| BoLA-2:03202 |  | 0.001944 | 21 |
| BoLA-2:00601 |  | 0.001915 | 23 |
| BoLA-2:01602 |  | 0.001915 | 23 |
| BoLA-3:06501 |  | 0.001852 | 40 |
| BoLA-2:01201 |  | 0.001757 | 23 |
| BoLA-T2a |  | 0.001757 | 23 |
| BoLA-3:00401 |  | 0.001681 | 21 |
| BoLA-3:00402 |  | 0.001681 | 21 |
| BoLA-3:00403 |  | 0.001681 | 21 |
| BoLA-3:05301 |  | 0.001681 | 21 |
| BoLA-gb1.7 |  | 0.001681 | 21 |
| BoLA-1:02901 |  | 0.00158 | 17 |
| BoLA-1:00901 |  | 0.00148 | 20 |
| BoLA-amani.1 |  | 0.001422 | 18 |
| BoLA-2:05401 |  | 0.001406 | 5.9 |
| BoLA-2:06901 |  | 0.00119 | 17 |
| BoLA-3:05201 |  | 0.001157 | 24 |
| BoLA-2:07101 |  | 0.001111 | 29 |
| BoLA-2:06001 |  | 0.000983 | 24 |
| BoLA-2:01601 |  | 0.000934 | 22 |
| BoLA-3:00101 |  | 0.000929 | 18 |
| BoLA-AW10 |  | 0.000929 | 18 |
| BoLA-1:01901 |  | 0.000871 | 16 |
| BoLA-3:02701 |  | 0.000818 | 27 |
| BoLA-3:02702 |  | 0.000818 | 27 |
| BoLA-3:03801 |  | 0.000811 | 37 |
| BoLA-2:01801 |  | 0.000804 | 23 |
| BoLA-2:01802 |  | 0.000804 | 23 |
| BoLA-1:02301 |  | 0.0008 | 29 |
| BoLA-D18.4 |  | 0.0008 | 29 |
| BoLA-2:04601 |  | 0.000786 | 38 |
| BoLA-2:00602 |  | 0.000778 | 35 |
| BoLA-2:04801 |  | 0.000749 | 23 |
| BoLA-3:07301 |  | 0.000643 | 21 |
| BoLA-1:02101 |  | 0.000589 | 7.6 |
| BoLA-3:00201 |  | 0.000588 | 42 |
| BoLA-JSP.1 |  | 0.000588 | 42 |
| BoLA-1:00902 |  | 0.000564 | 28 |
| BoLA-T5 |  | 0.000564 | 28 |
| BoLA-2:02601 |  | 0.000548 | 31 |
| BoLA-2:02602 |  | 0.000548 | 31 |
| BoLA-2:02603 |  | 0.000548 | 31 |
| BoLA-2:03001 |  | 0.000532 | 21 |
| BoLA-3:00103 |  | 0.000518 | 22 |
| BoLA-2:02501 |  | 0.000504 | 27 |
| BoLA-T7 |  | 0.0005 | 34 |
| BoLA-1:02001 |  | 0.000475 | 30 |
| BoLA-2:00802 |  | 0.000434 | 18 |
| BoLA-1:04201 |  | 0.000419 | 37 |
| BoLA-5:03901 |  | 0.000406 | 33 |
| BoLA-6:01502 |  | 0.000406 | 17 |
| BoLA-3:00102 |  | 0.000396 | 23 |
| BoLA-3:01001 |  | 0.000379 | 32 |
| BoLA-2:05501 |  | 0.000376 | 42 |
| BoLA-5:06401 |  | 0.000369 | 20 |
| BoLA-4:06301 |  | 0.000344 | 23 |
| BoLA-6:03401 |  | 0.000338 | 21 |
| BoLA-6:01501 |  | 0.000322 | 25 |
| BoLA-1:03102 |  | 0.000307 | 49 |
| BoLA-1:03101 |  | 0.000287 | 51 |
| BoLA-2:00801 |  | 0.00027 | 21 |
| BoLA-1:07401 |  | 0.000269 | 24 |
| BoLA-6:04001 |  | 0.000216 | 52 |
| BoLA-1:02801 |  | 0.000202 | 28 |
| BoLA-1:04901 |  | 0.000199 | 43 |
| BoLA-4:02402 |  | 0.000147 | 46 |
| BoLA-3:03501 |  | 0.000105 | 24 |
| BoLA-6:04101 |  | 6.2e-05 | 39 |
| BoLA-T2b |  | 6.2e-05 | 39 |
| BoLA-4:02401 |  | 3.8e-05 | 45 |
| BoLA-6:01301 |  | 1.9e-05 | 54 |
| BoLA-HD6 |  | 1.9e-05 | 54 |
| BoLA-2:02201 |  | 1.5e-05 | 63 |
| BoLA-6:01302 |  | 1e-05 | 50 |

| Alleles | Peptide | Score | Percentile rank |
| --- | --- | --- | --- |
| BoLA-3:01703 | HGKHHDDDS | 0.002 | 32 |
| BoLA-3:01702 |  | 0.000993 | 32 |
| BoLA-6:04001 |  | 0.00082 | 33 |
| BoLA-3:01701 |  | 0.000796 | 30 |
| BoLA-3:05001 |  | 0.000637 | 47 |
| BoLA-3:05002 |  | 0.000364 | 48 |
| BoLA-3:00101 |  | 0.000342 | 28 |
| BoLA-AW10 |  | 0.000342 | 28 |
| BoLA-2:04701 |  | 0.000306 | 53 |
| BoLA-1:06101 |  | 0.000285 | 46 |
| BoLA-3:03701 |  | 0.000257 | 76 |
| BoLA-3:05901 |  | 0.000223 | 49 |
| BoLA-5:00301 |  | 0.000214 | 74 |
| BoLA-2:04601 |  | 0.000192 | 56 |
| BoLA-3:05801 |  | 0.000162 | 69 |
| BoLA-3:02701 |  | 0.00013 | 52 |
| BoLA-3:02702 |  | 0.00013 | 52 |
| BoLA-3:00401 |  | 0.000108 | 55 |
| BoLA-3:00402 |  | 0.000108 | 55 |
| BoLA-3:00403 |  | 0.000108 | 55 |
| BoLA-3:05301 |  | 0.000108 | 55 |
| BoLA-gb1.7 |  | 0.000108 | 55 |
| BoLA-3:00201 |  | 0.000106 | 68 |
| BoLA-JSP.1 |  | 0.000106 | 68 |
| BoLA-2:00501 |  | 0.000103 | 76 |
| BoLA-2:04401 |  | 7.9e-05 | 82 |
| BoLA-2:07101 |  | 7.5e-05 | 63 |
| BoLA-3:05101 |  | 6.9e-05 | 60 |
| BoLA-3:06801 |  | 6.6e-05 | 67 |
| BoLA-1:06701 |  | 6.2e-05 | 76 |
| BoLA-2:04501 |  | 5.5e-05 | 71 |
| BoLA-3:06501 |  | 5.3e-05 | 83 |
| BoLA-2:02601 |  | 4.7e-05 | 63 |
| BoLA-2:02602 |  | 4.7e-05 | 63 |
| BoLA-2:02603 |  | 4.7e-05 | 63 |
| BoLA-3:00102 |  | 4.7e-05 | 49 |
| BoLA-2:06201 |  | 4.2e-05 | 79 |
| BoLA-3:01101 |  | 4.2e-05 | 63 |
| BoLA-3:06602 |  | 4.2e-05 | 78 |
| BoLA-2:03001 |  | 4.1e-05 | 51 |
| BoLA-2:04301 |  | 3.6e-05 | 73 |
| BoLA-2:01201 |  | 3.4e-05 | 74 |
| BoLA-3:03601 |  | 3.4e-05 | 76 |
| BoLA-T2a |  | 3.4e-05 | 74 |
| BoLA-3:06601 |  | 3.3e-05 | 77 |
| BoLA-5:07201 |  | 3.3e-05 | 63 |
| BoLA-2:06001 |  | 3.2e-05 | 71 |
| BoLA-2:00601 |  | 3.1e-05 | 75 |
| BoLA-2:01602 |  | 3.1e-05 | 75 |
| BoLA-3:01001 |  | 3.1e-05 | 65 |
| BoLA-3:03801 |  | 3.1e-05 | 78 |
| BoLA-2:02501 |  | 3e-05 | 66 |
| BoLA-1:00902 |  | 2.9e-05 | 63 |
| BoLA-T5 |  | 2.9e-05 | 63 |
| BoLA-2:05601 |  | 2.8e-05 | 64 |
| BoLA-3:00103 |  | 2.5e-05 | 60 |
| BoLA-2:00602 |  | 2.2e-05 | 83 |
| BoLA-2:03202 |  | 2.2e-05 | 76 |
| BoLA-T7 |  | 2e-05 | 72 |
| BoLA-1:02001 |  | 1.9e-05 | 73 |
| BoLA-1:03102 |  | 1.9e-05 | 86 |
| BoLA-6:01401 |  | 1.9e-05 | 75 |
| BoLA-1:02301 |  | 1.8e-05 | 75 |
| BoLA-1:04901 |  | 1.8e-05 | 74 |
| BoLA-D18.4 |  | 1.8e-05 | 75 |
| BoLA-amani.1 |  | 1.6e-05 | 71 |
| BoLA-1:01901 |  | 1.4e-05 | 67 |
| BoLA-2:05701 |  | 1.3e-05 | 88 |
| BoLA-6:01402 |  | 1.2e-05 | 86 |
| BoLA-1:04201 |  | 1.1e-05 | 83 |
| BoLA-2:04402 |  | 1.1e-05 | 82 |
| BoLA-2:05501 |  | 1.1e-05 | 86 |
| BoLA-2:07001 |  | 1.1e-05 | 73 |
| BoLA-3:03501 |  | 1.1e-05 | 51 |
| BoLA-4:02402 |  | 1.1e-05 | 81 |
| BoLA-5:03901 |  | 1.1e-05 | 80 |
| BoLA-6:03401 |  | 1.1e-05 | 61 |
| BoLA-1:03101 |  | 9e-06 | 91 |
| BoLA-2:01801 |  | 9e-06 | 81 |
| BoLA-2:01802 |  | 9e-06 | 81 |
| BoLA-2:01601 |  | 8e-06 | 85 |
| BoLA-1:00901 |  | 7e-06 | 85 |
| BoLA-1:02101 |  | 7e-06 | 53 |
| BoLA-6:01301 |  | 7e-06 | 67 |
| BoLA-HD6 |  | 7e-06 | 67 |
| BoLA-1:02901 |  | 6e-06 | 79 |
| BoLA-3:07301 |  | 6e-06 | 69 |
| BoLA-2:04801 |  | 5e-06 | 86 |
| BoLA-6:01501 |  | 4e-06 | 78 |
| BoLA-2:05401 |  | 3e-06 | 67 |
| BoLA-2:06901 |  | 3e-06 | 84 |
| BoLA-3:05201 |  | 3e-06 | 89 |
| BoLA-5:06401 |  | 3e-06 | 75 |
| BoLA-6:01502 |  | 3e-06 | 75 |
| BoLA-T2c |  | 3e-06 | 92 |
| BoLA-1:02801 |  | 2e-06 | 83 |
| BoLA-1:07401 |  | 2e-06 | 83 |
| BoLA-4:02401 |  | 2e-06 | 87 |
| BoLA-4:06301 |  | 2e-06 | 80 |
| BoLA-6:04101 |  | 2e-06 | 87 |
| BoLA-T2b |  | 2e-06 | 87 |
| BoLA-2:00801 |  | 1e-06 | 94 |
| BoLA-2:00802 |  | 1e-06 | 95 |
| BoLA-2:02201 |  | 1e-06 | 97 |
| BoLA-6:01302 |  | 1e-06 | 86 |

| Alleles | Peptide | Score | Percentile rank |
| --- | --- | --- | --- |
| BoLA-1:04901 | GKHHDDDSD | 0.000304 | 38 |
| BoLA-6:01402 |  | 0.000194 | 47 |
| BoLA-6:04001 |  | 6.4e-05 | 72 |
| BoLA-1:02301 |  | 5e-05 | 63 |
| BoLA-D18.4 |  | 5e-05 | 63 |
| BoLA-2:02601 |  | 4.1e-05 | 65 |
| BoLA-2:02602 |  | 4.1e-05 | 65 |
| BoLA-2:02603 |  | 4.1e-05 | 65 |
| BoLA-1:00902 |  | 3.5e-05 | 61 |
| BoLA-T5 |  | 3.5e-05 | 61 |
| BoLA-3:03701 |  | 3e-05 | 94 |
| BoLA-1:04201 |  | 2.8e-05 | 72 |
| BoLA-3:05901 |  | 2.5e-05 | 81 |
| BoLA-1:02001 |  | 2.1e-05 | 72 |
| BoLA-2:00601 |  | 2.1e-05 | 80 |
| BoLA-2:01602 |  | 2.1e-05 | 80 |
| BoLA-3:05001 |  | 2.1e-05 | 89 |
| BoLA-3:02701 |  | 1.8e-05 | 80 |
| BoLA-3:02702 |  | 1.8e-05 | 80 |
| BoLA-2:00501 |  | 1.7e-05 | 92 |
| BoLA-3:06501 |  | 1.6e-05 | 92 |
| BoLA-2:05501 |  | 1.5e-05 | 83 |
| BoLA-2:06001 |  | 1.4e-05 | 82 |
| BoLA-2:06201 |  | 1.4e-05 | 89 |
| BoLA-1:03102 |  | 1.3e-05 | 90 |
| BoLA-1:03101 |  | 1.2e-05 | 89 |
| BoLA-6:03401 |  | 1.1e-05 | 61 |
| BoLA-3:01703 |  | 1e-05 | 93 |
| BoLA-5:00301 |  | 1e-05 | 97 |
| BoLA-2:00602 |  | 9e-06 | 91 |
| BoLA-2:01601 |  | 9e-06 | 84 |
| BoLA-2:04701 |  | 8e-06 | 93 |
| BoLA-2:04801 |  | 8e-06 | 80 |
| BoLA-1:00901 |  | 7e-06 | 85 |
| BoLA-1:01901 |  | 7e-06 | 77 |
| BoLA-2:04401 |  | 7e-06 | 97 |
| BoLA-3:05801 |  | 7e-06 | 96 |
| BoLA-6:01401 |  | 7e-06 | 86 |
| BoLA-1:06101 |  | 6e-06 | 88 |
| BoLA-3:05002 |  | 6e-06 | 93 |
| BoLA-2:02501 |  | 4e-06 | 91 |
| BoLA-2:04301 |  | 4e-06 | 94 |
| BoLA-3:01001 |  | 4e-06 | 90 |
| BoLA-3:01702 |  | 4e-06 | 95 |
| BoLA-3:00401 |  | 3e-06 | 95 |
| BoLA-3:00402 |  | 3e-06 | 95 |
| BoLA-3:00403 |  | 3e-06 | 95 |
| BoLA-3:05301 |  | 3e-06 | 95 |
| BoLA-gb1.7 |  | 3e-06 | 95 |
| BoLA-1:02101 |  | 2e-06 | 73 |
| BoLA-1:02901 |  | 2e-06 | 91 |
| BoLA-1:06701 |  | 2e-06 | 98 |
| BoLA-2:01801 |  | 2e-06 | 95 |
| BoLA-2:01802 |  | 2e-06 | 95 |
| BoLA-2:04501 |  | 2e-06 | 98 |
| BoLA-2:05601 |  | 2e-06 | 91 |
| BoLA-3:01101 |  | 2e-06 | 94 |
| BoLA-3:05201 |  | 2e-06 | 92 |
| BoLA-3:06601 |  | 2e-06 | 97 |
| BoLA-3:06602 |  | 2e-06 | 99 |
| BoLA-4:02402 |  | 2e-06 | 96 |
| BoLA-5:07201 |  | 2e-06 | 94 |
| BoLA-1:07401 |  | 1e-06 | 92 |
| BoLA-2:02201 |  | 1e-06 | 97 |
| BoLA-2:03001 |  | 1e-06 | 96 |
| BoLA-2:04402 |  | 1e-06 | 99 |
| BoLA-2:04601 |  | 1e-06 | 100 |
| BoLA-2:06901 |  | 1e-06 | 95 |
| BoLA-2:07101 |  | 1e-06 | 99 |
| BoLA-3:00201 |  | 1e-06 | 100 |
| BoLA-3:01701 |  | 1e-06 | 100 |
| BoLA-3:03601 |  | 1e-06 | 99 |
| BoLA-3:03801 |  | 1e-06 | 100 |
| BoLA-3:05101 |  | 1e-06 | 99 |
| BoLA-3:06801 |  | 1e-06 | 99 |
| BoLA-4:02401 |  | 1e-06 | 95 |
| BoLA-6:01301 |  | 1e-06 | 92 |
| BoLA-6:04101 |  | 1e-06 | 95 |
| BoLA-amani.1 |  | 1e-06 | 98 |
| BoLA-HD6 |  | 1e-06 | 92 |
| BoLA-JSP.1 |  | 1e-06 | 100 |
| BoLA-T2b |  | 1e-06 | 95 |
| BoLA-1:02801 |  | 0.0 | 100 |
| BoLA-2:00801 |  | 0.0 | 100 |
| BoLA-2:00802 |  | 0.0 | 100 |
| BoLA-2:01201 |  | 0.0 | 100 |
| BoLA-2:03202 |  | 0.0 | 100 |
| BoLA-2:05401 |  | 0.0 | 100 |
| BoLA-2:05701 |  | 0.0 | 100 |
| BoLA-2:07001 |  | 0.0 | 100 |
| BoLA-3:00101 |  | 0.0 | 100 |
| BoLA-3:00102 |  | 0.0 | 100 |
| BoLA-3:00103 |  | 0.0 | 100 |
| BoLA-3:03501 |  | 0.0 | 100 |
| BoLA-3:07301 |  | 0.0 | 100 |
| BoLA-4:06301 |  | 0.0 | 100 |
| BoLA-5:03901 |  | 0.0 | 100 |
| BoLA-5:06401 |  | 0.0 | 100 |
| BoLA-6:01302 |  | 0.0 | 100 |
| BoLA-6:01501 |  | 0.0 | 100 |
| BoLA-6:01502 |  | 0.0 | 100 |
| BoLA-AW10 |  | 0.0 | 100 |
| BoLA-T2a |  | 0.0 | 100 |
| BoLA-T2c |  | 0.0 | 100 |
| BoLA-T7 |  | 0.0 | 100 |

| Alleles | Peptide | Score | Percentile rank |
| --- | --- | --- | --- |
| BoLA-3:03701 | CHGKHHDDD | 4.6e-05 | 92 |
| BoLA-2:04801 |  | 3.6e-05 | 60 |
| BoLA-2:02201 |  | 1.8e-05 | 60 |
| BoLA-3:05001 |  | 1.3e-05 | 93 |
| BoLA-3:06501 |  | 1.3e-05 | 94 |
| BoLA-2:01601 |  | 1.2e-05 | 80 |
| BoLA-3:05801 |  | 1.1e-05 | 94 |
| BoLA-5:00301 |  | 9e-06 | 97 |
| BoLA-2:00601 |  | 8e-06 | 89 |
| BoLA-2:01602 |  | 8e-06 | 89 |
| BoLA-2:00602 |  | 7e-06 | 93 |
| BoLA-1:03102 |  | 6e-06 | 95 |
| BoLA-3:05002 |  | 5e-06 | 94 |
| BoLA-3:05901 |  | 5e-06 | 95 |
| BoLA-2:01801 |  | 4e-06 | 90 |
| BoLA-2:01802 |  | 4e-06 | 90 |
| BoLA-2:06001 |  | 4e-06 | 95 |
| BoLA-3:01101 |  | 4e-06 | 89 |
| BoLA-6:04001 |  | 4e-06 | 98 |
| BoLA-1:03101 |  | 3e-06 | 97 |
| BoLA-1:04201 |  | 3e-06 | 94 |
| BoLA-2:02501 |  | 3e-06 | 94 |
| BoLA-3:00401 |  | 3e-06 | 95 |
| BoLA-3:00402 |  | 3e-06 | 95 |
| BoLA-3:00403 |  | 3e-06 | 95 |
| BoLA-3:02701 |  | 3e-06 | 97 |
| BoLA-3:02702 |  | 3e-06 | 97 |
| BoLA-3:05301 |  | 3e-06 | 95 |
| BoLA-gb1.7 |  | 3e-06 | 95 |
| BoLA-1:02001 |  | 2e-06 | 96 |
| BoLA-1:06701 |  | 2e-06 | 98 |
| BoLA-2:02601 |  | 2e-06 | 97 |
| BoLA-2:02602 |  | 2e-06 | 97 |
| BoLA-2:02603 |  | 2e-06 | 97 |
| BoLA-2:03001 |  | 2e-06 | 89 |
| BoLA-2:04701 |  | 2e-06 | 99 |
| BoLA-3:01703 |  | 2e-06 | 99 |
| BoLA-6:01402 |  | 2e-06 | 98 |
| BoLA-1:00902 |  | 1e-06 | 97 |
| BoLA-1:02301 |  | 1e-06 | 99 |
| BoLA-1:02901 |  | 1e-06 | 97 |
| BoLA-1:04901 |  | 1e-06 | 99 |
| BoLA-1:07401 |  | 1e-06 | 92 |
| BoLA-2:00501 |  | 1e-06 | 100 |
| BoLA-2:04301 |  | 1e-06 | 100 |
| BoLA-2:04601 |  | 1e-06 | 100 |
| BoLA-2:05501 |  | 1e-06 | 100 |
| BoLA-2:06201 |  | 1e-06 | 100 |
| BoLA-2:06901 |  | 1e-06 | 95 |
| BoLA-3:00201 |  | 1e-06 | 100 |
| BoLA-3:01701 |  | 1e-06 | 100 |
| BoLA-3:01702 |  | 1e-06 | 100 |
| BoLA-3:03801 |  | 1e-06 | 100 |
| BoLA-3:05101 |  | 1e-06 | 99 |
| BoLA-3:06602 |  | 1e-06 | 100 |
| BoLA-4:02402 |  | 1e-06 | 99 |
| BoLA-5:03901 |  | 1e-06 | 99 |
| BoLA-6:01401 |  | 1e-06 | 99 |
| BoLA-D18.4 |  | 1e-06 | 99 |
| BoLA-JSP.1 |  | 1e-06 | 100 |
| BoLA-T5 |  | 1e-06 | 97 |
| BoLA-1:00901 |  | 0.0 | 100 |
| BoLA-1:01901 |  | 0.0 | 100 |
| BoLA-1:02101 |  | 0.0 | 100 |
| BoLA-1:02801 |  | 0.0 | 100 |
| BoLA-1:06101 |  | 0.0 | 100 |
| BoLA-2:00801 |  | 0.0 | 100 |
| BoLA-2:00802 |  | 0.0 | 100 |
| BoLA-2:01201 |  | 0.0 | 100 |
| BoLA-2:03202 |  | 0.0 | 100 |
| BoLA-2:04401 |  | 0.0 | 100 |
| BoLA-2:04402 |  | 0.0 | 100 |
| BoLA-2:04501 |  | 0.0 | 100 |
| BoLA-2:05401 |  | 0.0 | 100 |
| BoLA-2:05601 |  | 0.0 | 100 |
| BoLA-2:05701 |  | 0.0 | 100 |
| BoLA-2:07001 |  | 0.0 | 100 |
| BoLA-2:07101 |  | 0.0 | 100 |
| BoLA-3:00101 |  | 0.0 | 100 |
| BoLA-3:00102 |  | 0.0 | 100 |
| BoLA-3:00103 |  | 0.0 | 100 |
| BoLA-3:01001 |  | 0.0 | 100 |
| BoLA-3:03501 |  | 0.0 | 100 |
| BoLA-3:03601 |  | 0.0 | 100 |
| BoLA-3:05201 |  | 0.0 | 100 |
| BoLA-3:06601 |  | 0.0 | 100 |
| BoLA-3:06801 |  | 0.0 | 100 |
| BoLA-3:07301 |  | 0.0 | 100 |
| BoLA-4:02401 |  | 0.0 | 100 |
| BoLA-4:06301 |  | 0.0 | 100 |
| BoLA-5:06401 |  | 0.0 | 100 |
| BoLA-5:07201 |  | 0.0 | 100 |
| BoLA-6:01301 |  | 0.0 | 100 |
| BoLA-6:01302 |  | 0.0 | 100 |
| BoLA-6:01501 |  | 0.0 | 100 |
| BoLA-6:01502 |  | 0.0 | 100 |
| BoLA-6:03401 |  | 0.0 | 100 |
| BoLA-6:04101 |  | 0.0 | 100 |
| BoLA-amani.1 |  | 0.0 | 100 |
| BoLA-AW10 |  | 0.0 | 100 |
| BoLA-HD6 |  | 0.0 | 100 |
| BoLA-T2a |  | 0.0 | 100 |
| BoLA-T2b |  | 0.0 | 100 |
| BoLA-T2c |  | 0.0 | 100 |
| BoLA-T7 |  | 0.0 | 100 |

| Alleles | Peptide | Score | Percentilerank |
| --- | --- | --- | --- |
| BoLA-2:04801 | KHHDDDSDG | 0.003144 | 13 |
| BoLA-5:00301 |  | 0.001081 | 53 |
| BoLA-3:05001 |  | 0.000847 | 42 |
| BoLA-2:00602 |  | 0.000559 | 39 |
| BoLA-2:00601 |  | 0.000419 | 40 |
| BoLA-2:01602 |  | 0.000419 | 40 |
| BoLA-3:06501 |  | 0.000364 | 60 |
| BoLA-6:01402 |  | 0.000362 | 38 |
| BoLA-3:05002 |  | 0.000293 | 50 |
| BoLA-3:03701 |  | 0.000278 | 75 |
| BoLA-1:06101 |  | 0.000264 | 47 |
| BoLA-3:05801 |  | 0.000258 | 63 |
| BoLA-2:04701 |  | 0.000192 | 60 |
| BoLA-3:01101 |  | 0.00018 | 44 |
| BoLA-3:01703 |  | 0.00015 | 66 |
| BoLA-3:01001 |  | 0.000111 | 48 |
| BoLA-2:01601 |  | 9.5e-05 | 52 |
| BoLA-3:01702 |  | 8.6e-05 | 64 |
| BoLA-1:03102 |  | 8.5e-05 | 68 |
| BoLA-1:02001 |  | 7.6e-05 | 53 |
| BoLA-2:04401 |  | 7.6e-05 | 82 |
| BoLA-3:05901 |  | 6.6e-05 | 68 |
| BoLA-1:03101 |  | 6.4e-05 | 71 |
| BoLA-1:04901 |  | 6.2e-05 | 58 |
| BoLA-3:01701 |  | 6.2e-05 | 65 |
| BoLA-1:04201 |  | 5.6e-05 | 63 |
| BoLA-1:06701 |  | 5.3e-05 | 78 |
| BoLA-2:02601 |  | 5.2e-05 | 62 |
| BoLA-2:02602 |  | 5.2e-05 | 62 |
| BoLA-2:02603 |  | 5.2e-05 | 62 |
| BoLA-2:04301 |  | 5.2e-05 | 68 |
| BoLA-2:06001 |  | 5.2e-05 | 64 |
| BoLA-2:02201 |  | 5e-05 | 45 |
| BoLA-6:01401 |  | 4.7e-05 | 63 |
| BoLA-1:01901 |  | 4.6e-05 | 49 |
| BoLA-1:02301 |  | 4.3e-05 | 64 |
| BoLA-D18.4 |  | 4.3e-05 | 64 |
| BoLA-2:04601 |  | 4.1e-05 | 75 |
| BoLA-6:04001 |  | 4.1e-05 | 78 |
| BoLA-3:06601 |  | 4e-05 | 74 |
| BoLA-3:06602 |  | 3.9e-05 | 79 |
| BoLA-3:02701 |  | 3.8e-05 | 70 |
| BoLA-3:02702 |  | 3.8e-05 | 70 |
| BoLA-1:02101 |  | 2.8e-05 | 33 |
| BoLA-2:06201 |  | 2.6e-05 | 84 |
| BoLA-3:05101 |  | 2.6e-05 | 73 |
| BoLA-4:02402 |  | 2.4e-05 | 71 |
| BoLA-5:07201 |  | 2.4e-05 | 67 |
| BoLA-1:02901 |  | 2.2e-05 | 63 |
| BoLA-2:04501 |  | 2.2e-05 | 82 |
| BoLA-2:02501 |  | 2.1e-05 | 71 |
| BoLA-2:05601 |  | 1.9e-05 | 68 |
| BoLA-4:02401 |  | 1.9e-05 | 55 |
| BoLA-2:01801 |  | 1.8e-05 | 72 |
| BoLA-2:01802 |  | 1.8e-05 | 72 |
| BoLA-6:04101 |  | 1.8e-05 | 55 |
| BoLA-T2b |  | 1.8e-05 | 55 |
| BoLA-2:00501 |  | 1.7e-05 | 92 |
| BoLA-2:03001 |  | 1.6e-05 | 63 |
| BoLA-2:06901 |  | 1.6e-05 | 64 |
| BoLA-6:03401 |  | 1.6e-05 | 56 |
| BoLA-1:00901 |  | 1.5e-05 | 77 |
| BoLA-2:05501 |  | 1.5e-05 | 83 |
| BoLA-3:00401 |  | 1.5e-05 | 81 |
| BoLA-3:00402 |  | 1.5e-05 | 81 |
| BoLA-3:00403 |  | 1.5e-05 | 81 |
| BoLA-3:05301 |  | 1.5e-05 | 81 |
| BoLA-gb1.7 |  | 1.5e-05 | 81 |
| BoLA-3:03801 |  | 1.3e-05 | 87 |
| BoLA-2:04402 |  | 1.1e-05 | 82 |
| BoLA-3:00201 |  | 1e-05 | 95 |
| BoLA-JSP.1 |  | 1e-05 | 95 |
| BoLA-3:00101 |  | 9e-06 | 80 |
| BoLA-3:03601 |  | 9e-06 | 88 |
| BoLA-AW10 |  | 9e-06 | 80 |
| BoLA-2:07001 |  | 8e-06 | 76 |
| BoLA-3:05201 |  | 8e-06 | 80 |
| BoLA-amani.1 |  | 8e-06 | 80 |
| BoLA-1:07401 |  | 7e-06 | 67 |
| BoLA-1:02801 |  | 6e-06 | 70 |
| BoLA-2:01201 |  | 5e-06 | 93 |
| BoLA-3:00102 |  | 5e-06 | 83 |
| BoLA-T2a |  | 5e-06 | 93 |
| BoLA-T7 |  | 5e-06 | 87 |
| BoLA-2:05701 |  | 4e-06 | 95 |
| BoLA-2:07101 |  | 4e-06 | 93 |
| BoLA-5:03901 |  | 4e-06 | 90 |
| BoLA-5:06401 |  | 4e-06 | 71 |
| BoLA-6:01501 |  | 4e-06 | 78 |
| BoLA-2:00802 |  | 3e-06 | 82 |
| BoLA-2:03202 |  | 3e-06 | 94 |
| BoLA-3:00103 |  | 3e-06 | 88 |
| BoLA-6:01502 |  | 3e-06 | 75 |
| BoLA-2:00801 |  | 2e-06 | 86 |
| BoLA-3:07301 |  | 2e-06 | 82 |
| BoLA-4:06301 |  | 2e-06 | 80 |
| BoLA-2:05401 |  | 1e-06 | 85 |
| BoLA-6:01301 |  | 1e-06 | 92 |
| BoLA-6:01302 |  | 1e-06 | 86 |
| BoLA-HD6 |  | 1e-06 | 92 |
| BoLA-T2c |  | 1e-06 | 98 |
| BoLA-3:03501 |  | 0.0 | 100 |

| Alleles | Peptide | Score | Percentile rank |
| --- | --- | --- | --- |
| BoLA-2:00601 | GDGAAPCHG | 0.000688 | 34 |
| BoLA-2:01602 |  | 0.000688 | 34 |
| BoLA-6:01402 |  | 0.000339 | 39 |
| BoLA-2:00602 |  | 0.000153 | 58 |
| BoLA-2:00501 |  | 0.000146 | 72 |
| BoLA-6:01401 |  | 9e-05 | 54 |
| BoLA-3:06501 |  | 8.1e-05 | 78 |
| BoLA-3:03701 |  | 7.2e-05 | 88 |
| BoLA-2:01601 |  | 6.1e-05 | 58 |
| BoLA-3:05801 |  | 5.9e-05 | 81 |
| BoLA-3:00401 |  | 5.1e-05 | 65 |
| BoLA-3:00402 |  | 5.1e-05 | 65 |
| BoLA-3:00403 |  | 5.1e-05 | 65 |
| BoLA-3:05301 |  | 5.1e-05 | 65 |
| BoLA-6:03401 |  | 5.1e-05 | 41 |
| BoLA-gb1.7 |  | 5.1e-05 | 65 |
| BoLA-1:03102 |  | 4.6e-05 | 76 |
| BoLA-3:00102 |  | 4.1e-05 | 52 |
| BoLA-3:05002 |  | 4e-05 | 75 |
| BoLA-3:05001 |  | 3.6e-05 | 84 |
| BoLA-2:04701 |  | 3.3e-05 | 81 |
| BoLA-3:01703 |  | 3.3e-05 | 83 |
| BoLA-1:01901 |  | 3.2e-05 | 54 |
| BoLA-1:02001 |  | 2.9e-05 | 67 |
| BoLA-3:01702 |  | 2.9e-05 | 77 |
| BoLA-2:06201 |  | 2.8e-05 | 83 |
| BoLA-1:03101 |  | 2.7e-05 | 82 |
| BoLA-1:04201 |  | 2.6e-05 | 73 |
| BoLA-2:06001 |  | 2.3e-05 | 76 |
| BoLA-5:00301 |  | 2.3e-05 | 94 |
| BoLA-2:04301 |  | 2.1e-05 | 79 |
| BoLA-3:02701 |  | 2.1e-05 | 78 |
| BoLA-3:02702 |  | 2.1e-05 | 78 |
| BoLA-3:05901 |  | 2.1e-05 | 83 |
| BoLA-2:05501 |  | 2e-05 | 80 |
| BoLA-3:06801 |  | 2e-05 | 80 |
| BoLA-3:05101 |  | 1.9e-05 | 76 |
| BoLA-1:02901 |  | 1.8e-05 | 66 |
| BoLA-2:04601 |  | 1.8e-05 | 84 |
| BoLA-3:01101 |  | 1.8e-05 | 73 |
| BoLA-3:00101 |  | 1.6e-05 | 71 |
| BoLA-3:03801 |  | 1.6e-05 | 85 |
| BoLA-AW10 |  | 1.6e-05 | 71 |
| BoLA-2:04401 |  | 1.5e-05 | 94 |
| BoLA-1:06101 |  | 1.4e-05 | 81 |
| BoLA-1:06701 |  | 1.4e-05 | 89 |
| BoLA-2:04402 |  | 1.2e-05 | 81 |
| BoLA-3:00103 |  | 1.2e-05 | 70 |
| BoLA-3:01701 |  | 1e-05 | 88 |
| BoLA-3:06601 |  | 1e-05 | 88 |
| BoLA-3:06602 |  | 1e-05 | 91 |
| BoLA-5:07201 |  | 8e-06 | 81 |
| BoLA-1:00901 |  | 7e-06 | 85 |
| BoLA-1:07401 |  | 7e-06 | 67 |
| BoLA-2:01201 |  | 7e-06 | 91 |
| BoLA-2:07101 |  | 7e-06 | 89 |
| BoLA-T2a |  | 7e-06 | 91 |
| BoLA-2:01801 |  | 6e-06 | 86 |
| BoLA-2:01802 |  | 6e-06 | 86 |
| BoLA-2:07001 |  | 6e-06 | 80 |
| BoLA-6:04001 |  | 6e-06 | 96 |
| BoLA-1:02301 |  | 5e-06 | 89 |
| BoLA-2:00801 |  | 5e-06 | 73 |
| BoLA-2:02601 |  | 5e-06 | 90 |
| BoLA-2:02602 |  | 5e-06 | 90 |
| BoLA-2:02603 |  | 5e-06 | 90 |
| BoLA-2:03202 |  | 5e-06 | 90 |
| BoLA-D18.4 |  | 5e-06 | 89 |
| BoLA-1:04901 |  | 4e-06 | 90 |
| BoLA-2:04501 |  | 4e-06 | 95 |
| BoLA-2:05401 |  | 4e-06 | 63 |
| BoLA-3:00201 |  | 4e-06 | 99 |
| BoLA-3:03601 |  | 4e-06 | 94 |
| BoLA-3:05201 |  | 4e-06 | 86 |
| BoLA-6:01501 |  | 4e-06 | 78 |
| BoLA-JSP.1 |  | 4e-06 | 99 |
| BoLA-T2c |  | 4e-06 | 90 |
| BoLA-1:00902 |  | 3e-06 | 89 |
| BoLA-2:02501 |  | 3e-06 | 94 |
| BoLA-2:04801 |  | 3e-06 | 91 |
| BoLA-2:05601 |  | 3e-06 | 88 |
| BoLA-4:02402 |  | 3e-06 | 93 |
| BoLA-6:04101 |  | 3e-06 | 82 |
| BoLA-amani.1 |  | 3e-06 | 90 |
| BoLA-T2b |  | 3e-06 | 82 |
| BoLA-T5 |  | 3e-06 | 89 |
| BoLA-2:05701 |  | 2e-06 | 98 |
| BoLA-3:01001 |  | 2e-06 | 95 |
| BoLA-3:03501 |  | 2e-06 | 75 |
| BoLA-5:03901 |  | 2e-06 | 96 |
| BoLA-5:06401 |  | 2e-06 | 81 |
| BoLA-T7 |  | 2e-06 | 94 |
| BoLA-1:02101 |  | 1e-06 | 85 |
| BoLA-1:02801 |  | 1e-06 | 92 |
| BoLA-2:00802 |  | 1e-06 | 95 |
| BoLA-2:03001 |  | 1e-06 | 96 |
| BoLA-2:06901 |  | 1e-06 | 95 |
| BoLA-4:02401 |  | 1e-06 | 95 |
| BoLA-6:01502 |  | 1e-06 | 90 |
| BoLA-2:02201 |  | 0.0 | 100 |
| BoLA-3:07301 |  | 0.0 | 100 |
| BoLA-4:06301 |  | 0.0 | 100 |
| BoLA-6:01301 |  | 0.0 | 100 |
| BoLA-6:01302 |  | 0.0 | 100 |
| BoLA-HD6 |  | 0.0 | 100 |
