# Supplementary material for: Development of a multi-epitope chimeric vaccine in silico against Babesia bovis, Theileria annulata, and Anaplasma marginale using computational biology tools and reverse vaccinology approach
Source: PLoS One. 2025 Jan 24;20(1):e0312262. doi: 10.1371/journal.pone.0312262 (PMC11759392; doi:10.1371/journal.pone.0312262)
Supplement: S6 File — (DOCX) [file pone.0312262.s012.docx]

The tables of all the ten MHC I epitopes of Vir-B10 with their scores and percentile rank representing their affinities for different BOLA alleles. The peptide which has been selected for chimeric vaccine construction has been represented in bold letters. The BoLA alleles binding with the selected peptide possessing a percentile value >50 is highlighted as yellow. The BoLA allele that has bonded with the selected peptide with the lowest percentile rank is highlighted as green.

| Alleles | Peptide | Score | Percentile rank |
| --- | --- | --- | --- |
| BoLA-3:01703 | **DGGGQGTDS** | 0.00091 | 42 |
| BoLA-3:01701 |  | 0.000847 | 29 |
| BoLA-3:05001 |  | 0.000727 | 45 |
| BoLA-3:03701 |  | 0.000638 | 65 |
| BoLA-3:01702 |  | 0.000485 | 40 |
| BoLA-3:05002 |  | 0.000479 | 44 |
| BoLA-3:00401 |  | 0.000203 | 46 |
| BoLA-3:00402 |  | 0.000203 | 46 |
| BoLA-3:00403 |  | 0.000203 | 46 |
| BoLA-3:05301 |  | 0.000203 | 46 |
| BoLA-gb1.7 |  | 0.000203 | 46 |
| BoLA-3:02701 |  | 0.000149 | 50 |
| BoLA-3:02702 |  | 0.000149 | 50 |
| BoLA-1:06101 |  | 0.000111 | 58 |
| BoLA-3:05901 |  | 0.00011 | 60 |
| BoLA-2:00501 |  | 9e-05 | 78 |
| BoLA-5:00301 |  | 8.3e-05 | 84 |
| BoLA-2:06001 |  | 8.1e-05 | 57 |
| BoLA-3:05801 |  | 7.7e-05 | 78 |
| BoLA-2:00602 |  | 5.4e-05 | 72 |
| BoLA-2:04601 |  | 4.9e-05 | 73 |
| BoLA-3:05101 |  | 4.1e-05 | 67 |
| BoLA-2:00601 |  | 4e-05 | 72 |
| BoLA-2:01602 |  | 4e-05 | 72 |
| BoLA-2:04301 |  | 3.6e-05 | 73 |
| BoLA-2:04701 |  | 3.6e-05 | 80 |
| BoLA-3:01001 |  | 3.6e-05 | 63 |
| BoLA-3:00201 |  | 3e-05 | 86 |
| BoLA-JSP.1 |  | 3e-05 | 86 |
| BoLA-2:01801 |  | 2.8e-05 | 66 |
| BoLA-2:01802 |  | 2.8e-05 | 66 |
| BoLA-3:03801 |  | 2.6e-05 | 80 |
| BoLA-2:01601 |  | 2.5e-05 | 71 |
| BoLA-2:04401 |  | 2.4e-05 | 91 |
| BoLA-2:01201 |  | 2.2e-05 | 80 |
| BoLA-T2a |  | 2.2e-05 | 80 |
| BoLA-2:02501 |  | 2e-05 | 72 |
| BoLA-6:01402 |  | 1.9e-05 | 80 |
| BoLA-1:06701 |  | 1.8e-05 | 87 |
| BoLA-2:04501 |  | 1.7e-05 | 84 |
| BoLA-3:01101 |  | 1.6e-05 | 75 |
| BoLA-1:02001 |  | 1.5e-05 | 76 |
| BoLA-2:07101 |  | 1.5e-05 | 82 |
| BoLA-6:04001 |  | 1.4e-05 | 91 |
| BoLA-1:01901 |  | 1.3e-05 | 68 |
| BoLA-3:03601 |  | 1.3e-05 | 85 |
| BoLA-6:01401 |  | 1.3e-05 | 80 |
| BoLA-2:04402 |  | 1.2e-05 | 81 |
| BoLA-3:00101 |  | 1e-05 | 78 |
| BoLA-AW10 |  | 1e-05 | 78 |
| BoLA-2:03202 |  | 9e-06 | 85 |
| BoLA-2:05601 |  | 9e-06 | 77 |
| BoLA-2:06201 |  | 8e-06 | 93 |
| BoLA-3:06501 |  | 8e-06 | 96 |
| BoLA-3:06801 |  | 8e-06 | 88 |
| BoLA-2:07001 |  | 7e-06 | 78 |
| BoLA-3:00103 |  | 7e-06 | 78 |
| BoLA-1:07401 |  | 6e-06 | 69 |
| BoLA-2:03001 |  | 6e-06 | 76 |
| BoLA-3:06602 |  | 6e-06 | 94 |
| BoLA-1:02901 |  | 5e-06 | 82 |
| BoLA-1:04201 |  | 5e-06 | 91 |
| BoLA-3:00102 |  | 5e-06 | 83 |
| BoLA-5:03901 |  | 5e-06 | 88 |
| BoLA-T2c |  | 5e-06 | 89 |
| BoLA-1:00901 |  | 4e-06 | 90 |
| BoLA-1:00902 |  | 4e-06 | 86 |
| BoLA-1:02301 |  | 4e-06 | 91 |
| BoLA-1:03101 |  | 4e-06 | 96 |
| BoLA-1:03102 |  | 4e-06 | 97 |
| BoLA-2:05701 |  | 4e-06 | 95 |
| BoLA-3:06601 |  | 4e-06 | 94 |
| BoLA-4:02402 |  | 4e-06 | 91 |
| BoLA-5:07201 |  | 4e-06 | 88 |
| BoLA-6:03401 |  | 4e-06 | 75 |
| BoLA-D18.4 |  | 4e-06 | 91 |
| BoLA-T5 |  | 4e-06 | 86 |
| BoLA-2:02601 |  | 3e-06 | 94 |
| BoLA-2:02602 |  | 3e-06 | 94 |
| BoLA-2:02603 |  | 3e-06 | 94 |
| BoLA-2:05501 |  | 3e-06 | 96 |
| BoLA-T7 |  | 3e-06 | 92 |
| BoLA-1:04901 |  | 2e-06 | 95 |
| BoLA-2:04801 |  | 2e-06 | 94 |
| BoLA-2:06901 |  | 2e-06 | 89 |
| BoLA-3:03501 |  | 2e-06 | 75 |
| BoLA-amani.1 |  | 2e-06 | 93 |
| BoLA-1:02101 |  | 1e-06 | 85 |
| BoLA-2:00802 |  | 1e-06 | 95 |
| BoLA-2:02201 |  | 1e-06 | 97 |
| BoLA-2:05401 |  | 1e-06 | 85 |
| BoLA-3:05201 |  | 1e-06 | 97 |
| BoLA-4:02401 |  | 1e-06 | 95 |
| BoLA-4:06301 |  | 1e-06 | 89 |
| BoLA-5:06401 |  | 1e-06 | 90 |
| BoLA-6:04101 |  | 1e-06 | 95 |
| BoLA-T2b |  | 1e-06 | 95 |
| BoLA-1:02801 |  | 0.0 | 100 |
| BoLA-2:00801 |  | 0.0 | 100 |
| BoLA-3:07301 |  | 0.0 | 100 |
| BoLA-6:01301 |  | 0.0 | 100 |
| BoLA-6:01302 |  | 0.0 | 100 |
| BoLA-6:01501 |  | 0.0 | 100 |
| BoLA-6:01502 |  | 0.0 | 100 |
| BoLA-HD6 |  | 0.0 | 100 |

| Alleles | Peptide | Score | Percentile rank |
| --- | --- | --- | --- |
| BoLA-2:01201 | GGGQGTDSR | 0.018939 | 7.7 |
| BoLA-T2a |  | 0.018939 | 7.7 |
| BoLA-3:03701 |  | 0.016857 | 19 |
| BoLA-2:04401 |  | 0.010436 | 22 |
| BoLA-2:04701 |  | 0.009396 | 14 |
| BoLA-2:04601 |  | 0.005583 | 17 |
| BoLA-3:05101 |  | 0.005215 | 12 |
| BoLA-2:04501 |  | 0.004321 | 19 |
| BoLA-3:05801 |  | 0.002796 | 31 |
| BoLA-2:03202 |  | 0.002662 | 18 |
| BoLA-2:04402 |  | 0.001269 | 25 |
| BoLA-2:07101 |  | 0.001257 | 28 |
| BoLA-3:06501 |  | 0.001202 | 45 |
| BoLA-2:04301 |  | 0.001199 | 27 |
| BoLA-3:01703 |  | 0.001094 | 39 |
| BoLA-2:05501 |  | 0.000932 | 31 |
| BoLA-2:00501 |  | 0.000823 | 48 |
| BoLA-3:01702 |  | 0.000789 | 34 |
| BoLA-6:04001 |  | 0.000782 | 34 |
| BoLA-3:06801 |  | 0.000713 | 39 |
| BoLA-2:07001 |  | 0.000704 | 24 |
| BoLA-1:00901 |  | 0.000623 | 29 |
| BoLA-2:06201 |  | 0.000617 | 43 |
| BoLA-5:00301 |  | 0.00058 | 61 |
| BoLA-1:06701 |  | 0.000578 | 49 |
| BoLA-1:03102 |  | 0.000539 | 42 |
| BoLA-1:00902 |  | 0.000503 | 29 |
| BoLA-T5 |  | 0.000503 | 29 |
| BoLA-3:01701 |  | 0.000467 | 36 |
| BoLA-3:06602 |  | 0.000467 | 48 |
| BoLA-2:00602 |  | 0.000466 | 42 |
| BoLA-3:00201 |  | 0.000398 | 47 |
| BoLA-JSP.1 |  | 0.000398 | 47 |
| BoLA-3:06601 |  | 0.000383 | 46 |
| BoLA-3:03601 |  | 0.000335 | 48 |
| BoLA-1:02301 |  | 0.000295 | 40 |
| BoLA-D18.4 |  | 0.000295 | 40 |
| BoLA-1:03101 |  | 0.000291 | 51 |
| BoLA-amani.1 |  | 0.000275 | 34 |
| BoLA-3:05201 |  | 0.00027 | 38 |
| BoLA-5:07201 |  | 0.000252 | 38 |
| BoLA-3:03801 |  | 0.000248 | 52 |
| BoLA-1:02001 |  | 0.000244 | 38 |
| BoLA-3:00401 |  | 0.000232 | 44 |
| BoLA-3:00402 |  | 0.000232 | 44 |
| BoLA-3:00403 |  | 0.000232 | 44 |
| BoLA-3:05301 |  | 0.000232 | 44 |
| BoLA-gb1.7 |  | 0.000232 | 44 |
| BoLA-2:06901 |  | 0.000211 | 32 |
| BoLA-3:01101 |  | 0.000202 | 42 |
| BoLA-2:00601 |  | 0.000197 | 51 |
| BoLA-2:01602 |  | 0.000197 | 51 |
| BoLA-1:06101 |  | 0.000183 | 52 |
| BoLA-3:05001 |  | 0.000179 | 65 |
| BoLA-5:03901 |  | 0.000167 | 44 |
| BoLA-6:01402 |  | 0.000157 | 50 |
| BoLA-1:04201 |  | 0.000156 | 49 |
| BoLA-2:06001 |  | 0.000128 | 50 |
| BoLA-2:05701 |  | 0.000122 | 63 |
| BoLA-4:02402 |  | 0.000117 | 50 |
| BoLA-3:03501 |  | 0.000109 | 24 |
| BoLA-2:05601 |  | 9e-05 | 49 |
| BoLA-5:06401 |  | 9e-05 | 32 |
| BoLA-3:05901 |  | 8.6e-05 | 64 |
| BoLA-3:05002 |  | 8.2e-05 | 67 |
| BoLA-2:03001 |  | 7.4e-05 | 43 |
| BoLA-2:02501 |  | 7.1e-05 | 53 |
| BoLA-3:02701 |  | 6.6e-05 | 62 |
| BoLA-3:02702 |  | 6.6e-05 | 62 |
| BoLA-T2c |  | 6.4e-05 | 65 |
| BoLA-2:02201 |  | 5.7e-05 | 43 |
| BoLA-3:00101 |  | 5.5e-05 | 52 |
| BoLA-AW10 |  | 5.5e-05 | 52 |
| BoLA-2:04801 |  | 5.1e-05 | 55 |
| BoLA-6:01401 |  | 5.1e-05 | 62 |
| BoLA-2:01601 |  | 5e-05 | 61 |
| BoLA-4:02401 |  | 5e-05 | 42 |
| BoLA-T7 |  | 3.8e-05 | 64 |
| BoLA-2:00801 |  | 3.5e-05 | 44 |
| BoLA-3:00103 |  | 3.5e-05 | 54 |
| BoLA-3:00102 |  | 3.4e-05 | 54 |
| BoLA-1:02801 |  | 2.5e-05 | 52 |
| BoLA-1:02901 |  | 2.4e-05 | 62 |
| BoLA-3:01001 |  | 1.7e-05 | 74 |
| BoLA-6:01501 |  | 1.5e-05 | 61 |
| BoLA-2:00802 |  | 1.4e-05 | 59 |
| BoLA-2:01801 |  | 1.4e-05 | 76 |
| BoLA-2:01802 |  | 1.4e-05 | 76 |
| BoLA-3:07301 |  | 1.3e-05 | 59 |
| BoLA-1:07401 |  | 1.2e-05 | 59 |
| BoLA-2:05401 |  | 1.2e-05 | 47 |
| BoLA-1:01901 |  | 1.1e-05 | 71 |
| BoLA-1:04901 |  | 1.1e-05 | 80 |
| BoLA-2:02601 |  | 1e-05 | 83 |
| BoLA-2:02602 |  | 1e-05 | 83 |
| BoLA-2:02603 |  | 1e-05 | 83 |
| BoLA-4:06301 |  | 1e-05 | 60 |
| BoLA-6:03401 |  | 8e-06 | 66 |
| BoLA-1:02101 |  | 6e-06 | 55 |
| BoLA-6:01502 |  | 5e-06 | 68 |
| BoLA-6:04101 |  | 5e-06 | 74 |
| BoLA-T2b |  | 5e-06 | 74 |
| BoLA-6:01301 |  | 4e-06 | 74 |
| BoLA-6:01302 |  | 4e-06 | 63 |
| BoLA-HD6 |  | 4e-06 | 74 |

| Alleles | Peptide | Score | Percentile rank |
| --- | --- | --- | --- |
| BoLA-2:04401 | IVLGGGGDG | 0.000535 | 60 |
| BoLA-5:00301 |  | 0.000507 | 63 |
| BoLA-1:06101 |  | 0.000402 | 42 |
| BoLA-3:01703 |  | 0.000285 | 57 |
| BoLA-2:06201 |  | 0.000241 | 56 |
| BoLA-2:04402 |  | 0.000231 | 45 |
| BoLA-3:01702 |  | 0.000169 | 55 |
| BoLA-3:03701 |  | 0.000158 | 81 |
| BoLA-2:04501 |  | 0.000148 | 59 |
| BoLA-1:06701 |  | 0.000147 | 66 |
| BoLA-3:01701 |  | 0.000121 | 56 |
| BoLA-3:06601 |  | 0.00012 | 61 |
| BoLA-3:06801 |  | 9.9e-05 | 63 |
| BoLA-2:07001 |  | 9.4e-05 | 46 |
| BoLA-2:05701 |  | 8.8e-05 | 67 |
| BoLA-3:03601 |  | 8.5e-05 | 65 |
| BoLA-3:06602 |  | 8.4e-05 | 70 |
| BoLA-2:00501 |  | 7.4e-05 | 80 |
| BoLA-1:00901 |  | 7e-05 | 57 |
| BoLA-1:02301 |  | 6.7e-05 | 59 |
| BoLA-4:02402 |  | 6.7e-05 | 57 |
| BoLA-D18.4 |  | 6.7e-05 | 59 |
| BoLA-3:05001 |  | 5.5e-05 | 80 |
| BoLA-2:03202 |  | 5e-05 | 66 |
| BoLA-2:04701 |  | 4.8e-05 | 77 |
| BoLA-T7 |  | 4.8e-05 | 61 |
| BoLA-amani.1 |  | 4.7e-05 | 57 |
| BoLA-2:01201 |  | 4.5e-05 | 71 |
| BoLA-T2a |  | 4.5e-05 | 71 |
| BoLA-2:00602 |  | 4.2e-05 | 76 |
| BoLA-3:05801 |  | 4.2e-05 | 84 |
| BoLA-6:01402 |  | 3.9e-05 | 71 |
| BoLA-3:00401 |  | 3.7e-05 | 70 |
| BoLA-3:00402 |  | 3.7e-05 | 70 |
| BoLA-3:00403 |  | 3.7e-05 | 70 |
| BoLA-3:05301 |  | 3.7e-05 | 70 |
| BoLA-gb1.7 |  | 3.7e-05 | 70 |
| BoLA-2:07101 |  | 3.6e-05 | 72 |
| BoLA-6:01401 |  | 3.6e-05 | 67 |
| BoLA-6:04001 |  | 3.4e-05 | 81 |
| BoLA-2:01601 |  | 3.3e-05 | 67 |
| BoLA-2:00601 |  | 3.1e-05 | 75 |
| BoLA-2:01602 |  | 3.1e-05 | 75 |
| BoLA-3:05901 |  | 3.1e-05 | 78 |
| BoLA-1:03102 |  | 3e-05 | 81 |
| BoLA-1:03101 |  | 2.7e-05 | 82 |
| BoLA-2:04301 |  | 2.7e-05 | 77 |
| BoLA-1:00902 |  | 2.6e-05 | 65 |
| BoLA-T5 |  | 2.6e-05 | 65 |
| BoLA-2:04601 |  | 2.5e-05 | 80 |
| BoLA-3:00201 |  | 2.5e-05 | 88 |
| BoLA-4:02401 |  | 2.5e-05 | 51 |
| BoLA-JSP.1 |  | 2.5e-05 | 88 |
| BoLA-1:02801 |  | 2.3e-05 | 53 |
| BoLA-2:02501 |  | 2.3e-05 | 69 |
| BoLA-3:05002 |  | 2.2e-05 | 82 |
| BoLA-3:00103 |  | 2e-05 | 63 |
| BoLA-3:01001 |  | 2e-05 | 71 |
| BoLA-3:05101 |  | 2e-05 | 76 |
| BoLA-2:01801 |  | 1.9e-05 | 72 |
| BoLA-2:01802 |  | 1.9e-05 | 72 |
| BoLA-3:01101 |  | 1.8e-05 | 73 |
| BoLA-3:02701 |  | 1.7e-05 | 81 |
| BoLA-3:02702 |  | 1.7e-05 | 81 |
| BoLA-2:00802 |  | 1.6e-05 | 57 |
| BoLA-2:05501 |  | 1.6e-05 | 82 |
| BoLA-2:05601 |  | 1.6e-05 | 70 |
| BoLA-2:06001 |  | 1.6e-05 | 80 |
| BoLA-T2c |  | 1.5e-05 | 79 |
| BoLA-2:00801 |  | 1.4e-05 | 58 |
| BoLA-2:02601 |  | 1.3e-05 | 80 |
| BoLA-2:02602 |  | 1.3e-05 | 80 |
| BoLA-2:02603 |  | 1.3e-05 | 80 |
| BoLA-3:06501 |  | 1.3e-05 | 94 |
| BoLA-5:06401 |  | 1.3e-05 | 55 |
| BoLA-2:06901 |  | 1.2e-05 | 68 |
| BoLA-1:04901 |  | 1e-05 | 81 |
| BoLA-3:05201 |  | 1e-05 | 77 |
| BoLA-5:03901 |  | 1e-05 | 81 |
| BoLA-1:02101 |  | 9e-06 | 49 |
| BoLA-3:00102 |  | 9e-06 | 75 |
| BoLA-4:06301 |  | 8e-06 | 62 |
| BoLA-2:03001 |  | 7e-06 | 74 |
| BoLA-5:07201 |  | 7e-06 | 82 |
| BoLA-1:02001 |  | 6e-06 | 87 |
| BoLA-1:04201 |  | 6e-06 | 89 |
| BoLA-2:04801 |  | 6e-06 | 84 |
| BoLA-3:00101 |  | 6e-06 | 85 |
| BoLA-AW10 |  | 6e-06 | 85 |
| BoLA-3:03801 |  | 5e-06 | 94 |
| BoLA-6:01301 |  | 5e-06 | 71 |
| BoLA-HD6 |  | 5e-06 | 71 |
| BoLA-1:01901 |  | 4e-06 | 85 |
| BoLA-6:03401 |  | 4e-06 | 75 |
| BoLA-1:02901 |  | 2e-06 | 91 |
| BoLA-2:05401 |  | 2e-06 | 74 |
| BoLA-3:03501 |  | 2e-06 | 75 |
| BoLA-3:07301 |  | 2e-06 | 82 |
| BoLA-6:01501 |  | 2e-06 | 86 |
| BoLA-6:04101 |  | 2e-06 | 87 |
| BoLA-T2b |  | 2e-06 | 87 |
| BoLA-1:07401 |  | 1e-06 | 92 |
| BoLA-2:02201 |  | 1e-06 | 97 |
| BoLA-6:01302 |  | 1e-06 | 86 |
| BoLA-6:01502 |  | 1e-06 | 90 |

| Alleles | Peptide | Score | Percentile rank |
| --- | --- | --- | --- |
| BoLA-5:00301 | ELGRNGSAG | 0.00074 | 58 |
| BoLA-2:00501 |  | 0.000633 | 52 |
| BoLA-1:06101 |  | 0.000522 | 39 |
| BoLA-T2c |  | 0.000478 | 44 |
| BoLA-2:06201 |  | 0.000448 | 47 |
| BoLA-2:05701 |  | 0.000406 | 48 |
| BoLA-3:00102 |  | 0.000405 | 23 |
| BoLA-3:05001 |  | 0.000373 | 54 |
| BoLA-2:00602 |  | 0.00036 | 46 |
| BoLA-2:00601 |  | 0.000287 | 46 |
| BoLA-2:01602 |  | 0.000287 | 46 |
| BoLA-3:01703 |  | 0.000274 | 58 |
| BoLA-T7 |  | 0.000258 | 41 |
| BoLA-3:03701 |  | 0.000242 | 77 |
| BoLA-6:01401 |  | 0.000161 | 47 |
| BoLA-2:04401 |  | 0.000158 | 74 |
| BoLA-3:03601 |  | 0.00014 | 59 |
| BoLA-6:01402 |  | 0.000133 | 53 |
| BoLA-3:05801 |  | 0.000131 | 72 |
| BoLA-3:05002 |  | 0.000122 | 62 |
| BoLA-3:06801 |  | 0.000116 | 61 |
| BoLA-2:01601 |  | 0.000113 | 49 |
| BoLA-3:01702 |  | 0.00011 | 60 |
| BoLA-1:06701 |  | 0.000107 | 70 |
| BoLA-2:01801 |  | 0.000101 | 48 |
| BoLA-2:01802 |  | 0.000101 | 48 |
| BoLA-1:02001 |  | 9e-05 | 51 |
| BoLA-2:06001 |  | 8.5e-05 | 56 |
| BoLA-1:03102 |  | 8.4e-05 | 68 |
| BoLA-6:04001 |  | 8.3e-05 | 67 |
| BoLA-3:01701 |  | 7.7e-05 | 62 |
| BoLA-3:05901 |  | 7.6e-05 | 65 |
| BoLA-3:00201 |  | 6.7e-05 | 75 |
| BoLA-6:01501 |  | 6.7e-05 | 42 |
| BoLA-JSP.1 |  | 6.7e-05 | 75 |
| BoLA-2:02501 |  | 6.6e-05 | 54 |
| BoLA-2:04501 |  | 6.6e-05 | 69 |
| BoLA-3:03801 |  | 6.6e-05 | 69 |
| BoLA-3:01001 |  | 6.2e-05 | 56 |
| BoLA-5:07201 |  | 6.2e-05 | 55 |
| BoLA-6:04101 |  | 6.2e-05 | 39 |
| BoLA-T2b |  | 6.2e-05 | 39 |
| BoLA-1:00901 |  | 6e-05 | 59 |
| BoLA-3:00103 |  | 4.6e-05 | 50 |
| BoLA-2:04701 |  | 4.5e-05 | 78 |
| BoLA-1:03101 |  | 4.4e-05 | 76 |
| BoLA-2:04601 |  | 4.4e-05 | 74 |
| BoLA-3:00401 |  | 4.3e-05 | 68 |
| BoLA-3:00402 |  | 4.3e-05 | 68 |
| BoLA-3:00403 |  | 4.3e-05 | 68 |
| BoLA-3:05301 |  | 4.3e-05 | 68 |
| BoLA-gb1.7 |  | 4.3e-05 | 68 |
| BoLA-2:03001 |  | 4.2e-05 | 50 |
| BoLA-1:01901 |  | 4.1e-05 | 51 |
| BoLA-2:04402 |  | 4.1e-05 | 67 |
| BoLA-4:02402 |  | 4.1e-05 | 64 |
| BoLA-1:02301 |  | 3.9e-05 | 66 |
| BoLA-D18.4 |  | 3.9e-05 | 66 |
| BoLA-3:06501 |  | 3.8e-05 | 86 |
| BoLA-3:02701 |  | 3.2e-05 | 73 |
| BoLA-3:02702 |  | 3.2e-05 | 73 |
| BoLA-6:03401 |  | 3.2e-05 | 47 |
| BoLA-5:03901 |  | 3.1e-05 | 67 |
| BoLA-3:00101 |  | 3e-05 | 62 |
| BoLA-AW10 |  | 3e-05 | 62 |
| BoLA-6:01502 |  | 2.7e-05 | 45 |
| BoLA-2:05501 |  | 2.6e-05 | 77 |
| BoLA-3:07301 |  | 2.4e-05 | 52 |
| BoLA-1:00902 |  | 2.2e-05 | 67 |
| BoLA-2:00802 |  | 2.2e-05 | 52 |
| BoLA-T5 |  | 2.2e-05 | 67 |
| BoLA-1:02901 |  | 2.1e-05 | 64 |
| BoLA-1:04201 |  | 2e-05 | 76 |
| BoLA-3:00102 |  | 2e-05 | 63 |
| BoLA-3:01101 |  | 2e-05 | 72 |
| BoLA-1:02101 |  | 1.9e-05 | 39 |
| BoLA-2:02601 |  | 1.9e-05 | 76 |
| BoLA-2:02602 |  | 1.9e-05 | 76 |
| BoLA-2:02603 |  | 1.9e-05 | 76 |
| BoLA-6:01301 |  | 1.9e-05 | 54 |
| BoLA-HD6 |  | 1.9e-05 | 54 |
| BoLA-3:05101 |  | 1.8e-05 | 77 |
| BoLA-2:04301 |  | 1.7e-05 | 82 |
| BoLA-1:07401 |  | 1.6e-05 | 56 |
| BoLA-2:00801 |  | 1.6e-05 | 56 |
| BoLA-4:06301 |  | 1.6e-05 | 54 |
| BoLA-3:06602 |  | 1.5e-05 | 88 |
| BoLA-2:07101 |  | 1.4e-05 | 83 |
| BoLA-2:01201 |  | 1.3e-05 | 85 |
| BoLA-T2a |  | 1.3e-05 | 85 |
| BoLA-3:06601 |  | 1.2e-05 | 87 |
| BoLA-4:02401 |  | 1e-05 | 65 |
| BoLA-amani.1 |  | 9e-06 | 79 |
| BoLA-1:02801 |  | 8e-06 | 67 |
| BoLA-1:04901 |  | 8e-06 | 83 |
| BoLA-2:05401 |  | 8e-06 | 53 |
| BoLA-2:07001 |  | 8e-06 | 76 |
| BoLA-6:01302 |  | 8e-06 | 53 |
| BoLA-2:05601 |  | 7e-06 | 79 |
| BoLA-2:04801 |  | 6e-06 | 84 |
| BoLA-2:06901 |  | 6e-06 | 76 |
| BoLA-2:03202 |  | 5e-06 | 90 |
| BoLA-3:05201 |  | 5e-06 | 84 |
| BoLA-5:06401 |  | 3e-06 | 75 |
| BoLA-3:03501 |  | 2e-06 | 75 |
| BoLA-2:02201 |  | 1e-06 | 97 |

| Alleles | Peptide | Score | Percentile rank |
| --- | --- | --- | --- |
| BoLA-3:03701 | SGTTETSEE | 0.012816 | 23 |
| BoLA-3:01703 |  | 0.012014 | 14 |
| BoLA-3:01702 |  | 0.009101 | 12 |
| BoLA-3:01701 |  | 0.005715 | 12 |
| BoLA-3:05801 |  | 0.003805 | 27 |
| BoLA-2:04701 |  | 0.003637 | 23 |
| BoLA-2:04601 |  | 0.002864 | 23 |
| BoLA-2:04301 |  | 0.002246 | 20 |
| BoLA-3:05101 |  | 0.001961 | 20 |
| BoLA-2:01201 |  | 0.001728 | 23 |
| BoLA-T2a |  | 0.001728 | 23 |
| BoLA-2:00501 |  | 0.001514 | 39 |
| BoLA-3:00201 |  | 0.001495 | 30 |
| BoLA-JSP.1 |  | 0.001495 | 30 |
| BoLA-3:05001 |  | 0.001393 | 36 |
| BoLA-5:03901 |  | 0.001329 | 21 |
| BoLA-2:00602 |  | 0.001312 | 29 |
| BoLA-2:04401 |  | 0.001238 | 48 |
| BoLA-2:04501 |  | 0.000927 | 35 |
| BoLA-3:05002 |  | 0.000925 | 36 |
| BoLA-3:00101 |  | 0.000864 | 19 |
| BoLA-AW10 |  | 0.000864 | 19 |
| BoLA-1:06701 |  | 0.000857 | 44 |
| BoLA-2:01801 |  | 0.000842 | 22 |
| BoLA-2:01802 |  | 0.000842 | 22 |
| BoLA-3:00401 |  | 0.000775 | 29 |
| BoLA-3:00402 |  | 0.000775 | 29 |
| BoLA-3:00403 |  | 0.000775 | 29 |
| BoLA-3:05301 |  | 0.000775 | 29 |
| BoLA-gb1.7 |  | 0.000775 | 29 |
| BoLA-2:05601 |  | 0.000737 | 25 |
| BoLA-2:06201 |  | 0.000691 | 42 |
| BoLA-3:01101 |  | 0.000607 | 30 |
| BoLA-3:06602 |  | 0.000605 | 44 |
| BoLA-3:06601 |  | 0.000591 | 40 |
| BoLA-2:00601 |  | 0.000562 | 37 |
| BoLA-2:01602 |  | 0.000562 | 37 |
| BoLA-2:04402 |  | 0.00044 | 37 |
| BoLA-5:07201 |  | 0.000401 | 33 |
| BoLA-2:03202 |  | 0.000391 | 39 |
| BoLA-6:04001 |  | 0.00039 | 43 |
| BoLA-5:00301 |  | 0.000363 | 67 |
| BoLA-3:05201 |  | 0.000351 | 35 |
| BoLA-3:06501 |  | 0.000344 | 61 |
| BoLA-3:03601 |  | 0.000337 | 48 |
| BoLA-3:05901 |  | 0.00033 | 43 |
| BoLA-2:07001 |  | 0.000308 | 32 |
| BoLA-1:06101 |  | 0.000263 | 47 |
| BoLA-3:02701 |  | 0.000247 | 42 |
| BoLA-3:02702 |  | 0.000247 | 42 |
| BoLA-2:07101 |  | 0.00024 | 47 |
| BoLA-1:00902 |  | 0.000218 | 38 |
| BoLA-T5 |  | 0.000218 | 38 |
| BoLA-3:00102 |  | 0.000212 | 30 |
| BoLA-3:03801 |  | 0.000206 | 54 |
| BoLA-1:03101 |  | 0.000192 | 56 |
| BoLA-2:02501 |  | 0.000184 | 39 |
| BoLA-1:00901 |  | 0.000174 | 44 |
| BoLA-2:03001 |  | 0.000174 | 32 |
| BoLA-2:06901 |  | 0.000166 | 35 |
| BoLA-1:03102 |  | 0.000165 | 58 |
| BoLA-1:02301 |  | 0.000134 | 50 |
| BoLA-D18.4 |  | 0.000134 | 50 |
| BoLA-3:06801 |  | 0.000129 | 60 |
| BoLA-3:03501 |  | 0.000124 | 22 |
| BoLA-2:05501 |  | 0.00012 | 57 |
| BoLA-2:06001 |  | 0.000116 | 51 |
| BoLA-4:06301 |  | 0.000107 | 33 |
| BoLA-2:05701 |  | 0.000105 | 65 |
| BoLA-amani.1 |  | 0.000104 | 46 |
| BoLA-6:01402 |  | 0.000103 | 57 |
| BoLA-2:04801 |  | 9e-05 | 47 |
| BoLA-T2c |  | 8.8e-05 | 61 |
| BoLA-1:02001 |  | 8.7e-05 | 51 |
| BoLA-1:02901 |  | 8.3e-05 | 46 |
| BoLA-2:01601 |  | 7.8e-05 | 55 |
| BoLA-3:00103 |  | 7.7e-05 | 43 |
| BoLA-4:02402 |  | 6.9e-05 | 57 |
| BoLA-6:01401 |  | 6.2e-05 | 60 |
| BoLA-5:06401 |  | 5e-05 | 39 |
| BoLA-T7 |  | 3.8e-05 | 64 |
| BoLA-1:04201 |  | 3.2e-05 | 71 |
| BoLA-3:01001 |  | 2.9e-05 | 66 |
| BoLA-1:04901 |  | 2.2e-05 | 71 |
| BoLA-1:02801 |  | 2.1e-05 | 54 |
| BoLA-2:02201 |  | 1.9e-05 | 59 |
| BoLA-2:02601 |  | 1.9e-05 | 76 |
| BoLA-2:02602 |  | 1.9e-05 | 76 |
| BoLA-2:02603 |  | 1.9e-05 | 76 |
| BoLA-2:00802 |  | 1.7e-05 | 56 |
| BoLA-6:03401 |  | 1.5e-05 | 57 |
| BoLA-1:01901 |  | 1.4e-05 | 67 |
| BoLA-2:00801 |  | 1.4e-05 | 58 |
| BoLA-1:07401 |  | 1.2e-05 | 59 |
| BoLA-4:02401 |  | 1.2e-05 | 62 |
| BoLA-2:05401 |  | 1.1e-05 | 48 |
| BoLA-3:07301 |  | 1e-05 | 62 |
| BoLA-6:01501 |  | 9e-06 | 68 |
| BoLA-1:02101 |  | 4e-06 | 62 |
| BoLA-6:01502 |  | 4e-06 | 71 |
| BoLA-6:04101 |  | 4e-06 | 78 |
| BoLA-T2b |  | 4e-06 | 78 |
| BoLA-6:01301 |  | 2e-06 | 83 |
| BoLA-6:01302 |  | 2e-06 | 74 |
| BoLA-HD6 |  | 2e-06 | 83 |

| Alleles | Peptide | Score | Percentile rank |
| --- | --- | --- | --- |
| BoLA-2:01201 | GSGTTETSE | 0.001971 | 22 |
| BoLA-T2a |  | 0.001971 | 22 |
| BoLA-2:04601 |  | 0.001283 | 32 |
| BoLA-2:03202 |  | 0.001138 | 27 |
| BoLA-5:00301 |  | 0.001125 | 52 |
| BoLA-1:06701 |  | 0.000992 | 42 |
| BoLA-2:04501 |  | 0.000979 | 35 |
| BoLA-2:04401 |  | 0.000956 | 52 |
| BoLA-2:04701 |  | 0.000805 | 40 |
| BoLA-3:01703 |  | 0.000778 | 44 |
| BoLA-2:07001 |  | 0.000705 | 24 |
| BoLA-2:00501 |  | 0.000656 | 51 |
| BoLA-3:03701 |  | 0.000652 | 65 |
| BoLA-2:06201 |  | 0.000558 | 44 |
| BoLA-2:05601 |  | 0.000437 | 30 |
| BoLA-1:06101 |  | 0.00041 | 42 |
| BoLA-2:07101 |  | 0.000403 | 41 |
| BoLA-3:01702 |  | 0.000389 | 43 |
| BoLA-3:05801 |  | 0.000369 | 58 |
| BoLA-3:06602 |  | 0.000334 | 52 |
| BoLA-3:05101 |  | 0.000318 | 40 |
| BoLA-3:06601 |  | 0.000277 | 50 |
| BoLA-1:00901 |  | 0.00027 | 39 |
| BoLA-3:01701 |  | 0.00022 | 47 |
| BoLA-3:05201 |  | 0.00019 | 42 |
| BoLA-2:04402 |  | 0.000187 | 47 |
| BoLA-1:03102 |  | 0.000167 | 58 |
| BoLA-5:03901 |  | 0.000167 | 44 |
| BoLA-3:03601 |  | 0.000163 | 57 |
| BoLA-amani.1 |  | 0.000161 | 41 |
| BoLA-1:04201 |  | 0.000157 | 49 |
| BoLA-2:06001 |  | 0.00015 | 48 |
| BoLA-3:00201 |  | 0.000144 | 64 |
| BoLA-3:06801 |  | 0.000144 | 58 |
| BoLA-JSP.1 |  | 0.000144 | 64 |
| BoLA-2:05701 |  | 0.00014 | 62 |
| BoLA-3:05001 |  | 0.00014 | 69 |
| BoLA-6:01402 |  | 0.000139 | 52 |
| BoLA-1:00902 |  | 0.000127 | 44 |
| BoLA-T5 |  | 0.000127 | 44 |
| BoLA-1:03101 |  | 0.000118 | 63 |
| BoLA-2:01601 |  | 0.000113 | 49 |
| BoLA-3:02701 |  | 0.000113 | 54 |
| BoLA-3:00401 |  | 0.000112 | 55 |
| BoLA-3:00402 |  | 0.000112 | 55 |
| BoLA-3:00403 |  | 0.000112 | 55 |
| BoLA-3:05301 |  | 0.000112 | 55 |
| BoLA-gb1.7 |  | 0.000112 | 55 |
| BoLA-3:05002 |  | 0.000109 | 63 |
| BoLA-3:01101 |  | 0.000108 | 50 |
| BoLA-5:07201 |  | 0.000101 | 49 |
| BoLA-2:00602 |  | 9.9e-05 | 64 |
| BoLA-2:05501 |  | 9.6e-05 | 60 |
| BoLA-2:02501 |  | 9.2e-05 | 49 |
| BoLA-3:06501 |  | 8.2e-05 | 78 |
| BoLA-2:00601 |  | 8.1e-05 | 63 |
| BoLA-2:01602 |  | 8.1e-05 | 63 |
| BoLA-1:02301 |  | 8e-05 | 56 |
| BoLA-D18.4 |  | 8e-05 | 56 |
| BoLA-1:02001 |  | 7.7e-05 | 53 |
| BoLA-2:06901 |  | 6.1e-05 | 47 |
| BoLA-6:04001 |  | 6e-05 | 73 |
| BoLA-2:04301 |  | 5.8e-05 | 67 |
| BoLA-2:03001 |  | 4.8e-05 | 48 |
| BoLA-T2c |  | 4.6e-05 | 68 |
| BoLA-3:05901 |  | 3.9e-05 | 75 |
| BoLA-3:07301 |  | 3.8e-05 | 47 |
| BoLA-4:02402 |  | 3.8e-05 | 65 |
| BoLA-3:02701 |  | 3.5e-05 | 71 |
| BoLA-3:02702 |  | 3.5e-05 | 71 |
| BoLA-3:03801 |  | 3.3e-05 | 78 |
| BoLA-1:02901 |  | 3.1e-05 | 58 |
| BoLA-5:06401 |  | 3.1e-05 | 44 |
| BoLA-2:04801 |  | 3e-05 | 62 |
| BoLA-3:00101 |  | 3e-05 | 62 |
| BoLA-AW10 |  | 3e-05 | 62 |
| BoLA-T7 |  | 2.5e-05 | 69 |
| BoLA-3:01001 |  | 2.4e-05 | 69 |
| BoLA-2:02601 |  | 2.1e-05 | 74 |
| BoLA-2:02602 |  | 2.1e-05 | 74 |
| BoLA-2:02603 |  | 2.1e-05 | 74 |
| BoLA-3:00103 |  | 2.1e-05 | 62 |
| BoLA-6:01401 |  | 2.1e-05 | 74 |
| BoLA-2:05401 |  | 1.7e-05 | 42 |
| BoLA-3:00102 |  | 1.7e-05 | 65 |
| BoLA-2:01801 |  | 1.6e-05 | 74 |
| BoLA-2:01802 |  | 1.6e-05 | 74 |
| BoLA-3:03501 |  | 1.6e-05 | 45 |
| BoLA-2:00801 |  | 1.4e-05 | 58 |
| BoLA-1:02101 |  | 1.2e-05 | 45 |
| BoLA-1:04901 |  | 1.2e-05 | 79 |
| BoLA-4:06301 |  | 1.2e-05 | 58 |
| BoLA-1:02801 |  | 1.1e-05 | 62 |
| BoLA-1:01901 |  | 1e-05 | 72 |
| BoLA-6:03401 |  | 1e-05 | 63 |
| BoLA-2:00802 |  | 6e-06 | 72 |
| BoLA-2:02201 |  | 6e-06 | 76 |
| BoLA-6:01501 |  | 6e-06 | 73 |
| BoLA-4:02401 |  | 5e-06 | 75 |
| BoLA-1:07401 |  | 4e-06 | 74 |
| BoLA-6:01502 |  | 3e-06 | 75 |
| BoLA-6:01301 |  | 2e-06 | 83 |
| BoLA-6:04101 |  | 2e-06 | 87 |
| BoLA-HD6 |  | 2e-06 | 83 |
| BoLA-T2b |  | 2e-06 | 87 |
| BoLA-6:01302 |  | 1e-06 | 86 |

| Alleles | Peptide | Score | Percentile rank |
| --- | --- | --- | --- |
| BoLA-2:00501 | MIVLGGGGD | 0.000152 | 71 |
| BoLA-3:03601 |  | 7.1e-05 | 67 |
| BoLA-T2c |  | 6.1e-05 | 65 |
| BoLA-2:04401 |  | 5.7e-05 | 85 |
| BoLA-2:06201 |  | 4.7e-05 | 78 |
| BoLA-5:00301 |  | 4.2e-05 | 90 |
| BoLA-3:06602 |  | 3.8e-05 | 79 |
| BoLA-3:03701 |  | 2.8e-05 | 94 |
| BoLA-2:00801 |  | 2.7e-05 | 48 |
| BoLA-1:06101 |  | 2.2e-05 | 76 |
| BoLA-3:06601 |  | 2.2e-05 | 81 |
| BoLA-1:00901 |  | 2.1e-05 | 73 |
| BoLA-3:05001 |  | 2e-05 | 90 |
| BoLA-3:01703 |  | 1.5e-05 | 90 |
| BoLA-T7 |  | 1.3e-05 | 77 |
| BoLA-1:03101 |  | 1.2e-05 | 89 |
| BoLA-1:03102 |  | 1.2e-05 | 90 |
| BoLA-1:06701 |  | 1.2e-05 | 90 |
| BoLA-2:01801 |  | 1.2e-05 | 78 |
| BoLA-2:01802 |  | 1.2e-05 | 78 |
| BoLA-2:04501 |  | 1.2e-05 | 87 |
| BoLA-2:05701 |  | 1.2e-05 | 88 |
| BoLA-3:00201 |  | 1.1e-05 | 94 |
| BoLA-JSP.1 |  | 1.1e-05 | 94 |
| BoLA-2:00802 |  | 1e-05 | 64 |
| BoLA-2:04402 |  | 1e-05 | 83 |
| BoLA-3:01702 |  | 1e-05 | 88 |
| BoLA-2:01601 |  | 9e-06 | 84 |
| BoLA-3:01701 |  | 9e-06 | 89 |
| BoLA-6:01401 |  | 9e-06 | 84 |
| BoLA-6:04001 |  | 9e-06 | 94 |
| BoLA-2:07001 |  | 8e-06 | 76 |
| BoLA-2:07101 |  | 8e-06 | 88 |
| BoLA-1:02301 |  | 7e-06 | 86 |
| BoLA-2:00602 |  | 7e-06 | 93 |
| BoLA-2:04601 |  | 7e-06 | 91 |
| BoLA-D18.4 |  | 7e-06 | 86 |
| BoLA-1:00902 |  | 6e-06 | 82 |
| BoLA-2:01201 |  | 6e-06 | 92 |
| BoLA-2:04701 |  | 6e-06 | 94 |
| BoLA-3:06801 |  | 6e-06 | 90 |
| BoLA-T2a |  | 6e-06 | 92 |
| BoLA-T5 |  | 6e-06 | 82 |
| BoLA-2:06001 |  | 5e-06 | 93 |
| BoLA-3:01001 |  | 5e-06 | 88 |
| BoLA-3:05002 |  | 5e-06 | 94 |
| BoLA-3:05101 |  | 5e-06 | 89 |
| BoLA-4:02402 |  | 5e-06 | 89 |
| BoLA-amani.1 |  | 5e-06 | 85 |
| BoLA-2:02501 |  | 4e-06 | 91 |
| BoLA-2:03202 |  | 4e-06 | 92 |
| BoLA-3:00401 |  | 4e-06 | 93 |
| BoLA-3:00402 |  | 4e-06 | 93 |
| BoLA-3:00403 |  | 4e-06 | 93 |
| BoLA-3:05301 |  | 4e-06 | 93 |
| BoLA-3:05901 |  | 4e-06 | 96 |
| BoLA-3:06501 |  | 4e-06 | 98 |
| BoLA-5:03901 |  | 4e-06 | 90 |
| BoLA-6:01402 |  | 4e-06 | 95 |
| BoLA-gb1.7 |  | 4e-06 | 93 |
| BoLA-1:01901 |  | 3e-06 | 88 |
| BoLA-1:02801 |  | 3e-06 | 79 |
| BoLA-2:00601 |  | 3e-06 | 96 |
| BoLA-2:01602 |  | 3e-06 | 96 |
| BoLA-2:04301 |  | 3e-06 | 96 |
| BoLA-3:03801 |  | 3e-06 | 97 |
| BoLA-3:05801 |  | 3e-06 | 99 |
| BoLA-4:02401 |  | 3e-06 | 82 |
| BoLA-5:07201 |  | 3e-06 | 91 |
| BoLA-1:02001 |  | 2e-06 | 96 |
| BoLA-2:02601 |  | 2e-06 | 97 |
| BoLA-2:02602 |  | 2e-06 | 97 |
| BoLA-2:02603 |  | 2e-06 | 97 |
| BoLA-2:05501 |  | 2e-06 | 98 |
| BoLA-3:02701 |  | 2e-06 | 98 |
| BoLA-3:02702 |  | 2e-06 | 98 |
| BoLA-3:05201 |  | 2e-06 | 92 |
| BoLA-1:02101 |  | 1e-06 | 85 |
| BoLA-1:02901 |  | 1e-06 | 97 |
| BoLA-1:04201 |  | 1e-06 | 99 |
| BoLA-1:04901 |  | 1e-06 | 99 |
| BoLA-1:07401 |  | 1e-06 | 92 |
| BoLA-2:02201 |  | 1e-06 | 97 |
| BoLA-2:03001 |  | 1e-06 | 96 |
| BoLA-2:04801 |  | 1e-06 | 98 |
| BoLA-2:05601 |  | 1e-06 | 96 |
| BoLA-2:06901 |  | 1e-06 | 95 |
| BoLA-3:00101 |  | 1e-06 | 99 |
| BoLA-3:00102 |  | 1e-06 | 98 |
| BoLA-3:00103 |  | 1e-06 | 97 |
| BoLA-3:01101 |  | 1e-06 | 98 |
| BoLA-3:07301 |  | 1e-06 | 90 |
| BoLA-5:06401 |  | 1e-06 | 90 |
| BoLA-6:01301 |  | 1e-06 | 92 |
| BoLA-6:03401 |  | 1e-06 | 93 |
| BoLA-6:04101 |  | 1e-06 | 95 |
| BoLA-AW10 |  | 1e-06 | 99 |
| BoLA-HD6 |  | 1e-06 | 92 |
| BoLA-T2b |  | 1e-06 | 95 |
| BoLA-2:05401 |  | 0.0 | 100 |
| BoLA-3:03501 |  | 0.0 | 100 |
| BoLA-4:06301 |  | 0.0 | 100 |
| BoLA-6:01302 |  | 0.0 | 100 |
| BoLA-6:01501 |  | 0.0 | 100 |
| BoLA-6:01502 |  | 0.0 | 100 |

| Alleles | Peptide | Score | Percentile rank |
| --- | --- | --- | --- |
| BoLA-1:06701 | GTDELGRNG | 0.019395 | 12 |
| BoLA-2:07001 |  | 0.006934 | 7.8 |
| BoLA-3:03701 |  | 0.006613 | 32 |
| BoLA-2:04401 |  | 0.006589 | 28 |
| BoLA-5:00301 |  | 0.005945 | 31 |
| BoLA-3:01703 |  | 0.005802 | 21 |
| BoLA-1:06101 |  | 0.005546 | 17 |
| BoLA-3:01702 |  | 0.004884 | 17 |
| BoLA-6:01402 |  | 0.004646 | 12 |
| BoLA-3:06801 |  | 0.003419 | 24 |
| BoLA-3:06602 |  | 0.00295 | 26 |
| BoLA-2:05601 |  | 0.002919 | 14 |
| BoLA-3:03601 |  | 0.002868 | 24 |
| BoLA-3:05201 |  | 0.002814 | 17 |
| BoLA-2:05401 |  | 0.002433 | 4.1 |
| BoLA-2:04402 |  | 0.002207 | 20 |
| BoLA-2:00501 |  | 0.00207 | 35 |
| BoLA-2:04501 |  | 0.001833 | 28 |
| BoLA-3:01701 |  | 0.001831 | 21 |
| BoLA-2:05701 |  | 0.00175 | 30 |
| BoLA-3:06601 |  | 0.001698 | 28 |
| BoLA-2:01601 |  | 0.001696 | 16 |
| BoLA-2:07101 |  | 0.00167 | 25 |
| BoLA-6:01401 |  | 0.001667 | 20 |
| BoLA-2:04701 |  | 0.001486 | 33 |
| BoLA-2:00601 |  | 0.001391 | 26 |
| BoLA-2:01602 |  | 0.001391 | 26 |
| BoLA-3:05801 |  | 0.001225 | 41 |
| BoLA-2:06201 |  | 0.0012 | 34 |
| BoLA-5:07201 |  | 0.001185 | 23 |
| BoLA-2:00602 |  | 0.00117 | 30 |
| BoLA-3:06501 |  | 0.001125 | 46 |
| BoLA-1:02901 |  | 0.000956 | 21 |
| BoLA-2:01201 |  | 0.000955 | 29 |
| BoLA-T2a |  | 0.000955 | 29 |
| BoLA-3:01101 |  | 0.000939 | 26 |
| BoLA-amani.1 |  | 0.000911 | 22 |
| BoLA-3:05001 |  | 0.000779 | 44 |
| BoLA-3:05002 |  | 0.000756 | 38 |
| BoLA-3:00401 |  | 0.000718 | 30 |
| BoLA-3:00402 |  | 0.000718 | 30 |
| BoLA-3:00403 |  | 0.000718 | 30 |
| BoLA-3:05301 |  | 0.000718 | 30 |
| BoLA-gb1.7 |  | 0.000718 | 30 |
| BoLA-2:03202 |  | 0.000655 | 33 |
| BoLA-T7 |  | 0.000638 | 32 |
| BoLA-3:00103 |  | 0.000548 | 21 |
| BoLA-5:03901 |  | 0.000522 | 30 |
| BoLA-3:05901 |  | 0.000506 | 38 |
| BoLA-2:02601 |  | 0.000501 | 32 |
| BoLA-2:02602 |  | 0.000501 | 32 |
| BoLA-2:02603 |  | 0.000501 | 32 |
| BoLA-2:06001 |  | 0.000472 | 32 |
| BoLA-2:04601 |  | 0.000416 | 46 |
| BoLA-2:04301 |  | 0.000415 | 40 |
| BoLA-T2c |  | 0.000372 | 47 |
| BoLA-2:02501 |  | 0.000323 | 32 |
| BoLA-1:02101 |  | 0.000318 | 11 |
| BoLA-2:00801 |  | 0.000305 | 20 |
| BoLA-1:02001 |  | 0.000298 | 35 |
| BoLA-3:00201 |  | 0.000264 | 54 |
| BoLA-JSP.1 |  | 0.000264 | 54 |
| BoLA-2:00802 |  | 0.000259 | 22 |
| BoLA-1:01901 |  | 0.000242 | 27 |
| BoLA-2:01801 |  | 0.000242 | 35 |
| BoLA-2:01802 |  | 0.000242 | 35 |
| BoLA-1:03101 |  | 0.000236 | 54 |
| BoLA-3:07301 |  | 0.000233 | 29 |
| BoLA-1:00901 |  | 0.000228 | 41 |
| BoLA-5:06401 |  | 0.000222 | 24 |
| BoLA-3:03801 |  | 0.000208 | 54 |
| BoLA-3:05101 |  | 0.000195 | 46 |
| BoLA-4:06301 |  | 0.000195 | 28 |
| BoLA-3:00102 |  | 0.000189 | 31 |
| BoLA-3:00101 |  | 0.000176 | 36 |
| BoLA-AW10 |  | 0.000176 | 36 |
| BoLA-2:03001 |  | 0.000175 | 32 |
| BoLA-3:02701 |  | 0.000169 | 48 |
| BoLA-3:02702 |  | 0.000169 | 48 |
| BoLA-1:03102 |  | 0.000168 | 58 |
| BoLA-2:05501 |  | 0.000163 | 53 |
| BoLA-1:02301 |  | 0.000154 | 48 |
| BoLA-D18.4 |  | 0.000154 | 48 |
| BoLA-6:03401 |  | 0.000151 | 29 |
| BoLA-1:04201 |  | 0.000138 | 51 |
| BoLA-1:04901 |  | 0.000132 | 48 |
| BoLA-4:02402 |  | 0.000126 | 49 |
| BoLA-2:04801 |  | 0.000124 | 43 |
| BoLA-3:03501 |  | 0.000124 | 22 |
| BoLA-1:00902 |  | 0.00012 | 45 |
| BoLA-2:06901 |  | 0.00012 | 39 |
| BoLA-T5 |  | 0.00012 | 45 |
| BoLA-1:07401 |  | 0.000112 | 32 |
| BoLA-1:02801 |  | 0.000104 | 35 |
| BoLA-6:04001 |  | 9.7e-05 | 65 |
| BoLA-6:04101 |  | 7.7e-05 | 36 |
| BoLA-T2b |  | 7.7e-05 | 36 |
| BoLA-3:01001 |  | 7.3e-05 | 53 |
| BoLA-6:01501 |  | 7.3e-05 | 41 |
| BoLA-4:02401 |  | 3e-05 | 49 |
| BoLA-6:01301 |  | 2.4e-05 | 51 |
| BoLA-HD6 |  | 2.4e-05 | 51 |
| BoLA-6:01502 |  | 2e-05 | 49 |
| BoLA-2:01201 |  | 2e-05 | 81 |
| BoLA-2:02201 |  | 9e-06 | 70 |
| BoLA-6:01302 |  | 6e-06 | 57 |

| Alleles | Peptide | Score | Percentile rank |
| --- | --- | --- | --- |
| BoLA-1:06701 | GTTETSEEP | 0.028132 | 9.4 |
| BoLA-1:06101 |  | 0.022435 | 6.9 |
| BoLA-3:01703 |  | 0.01348 | 13 |
| BoLA-6:01402 |  | 0.013157 | 7.1 |
| BoLA-3:01702 |  | 0.010945 | 11 |
| BoLA-5:00301 |  | 0.009473 | 25 |
| BoLA-2:00501 |  | 0.008824 | 18 |
| BoLA-2:04401 |  | 0.008328 | 25 |
| BoLA-3:03601 |  | 0.007008 | 16 |
| BoLA-3:03701 |  | 0.004946 | 36 |
| BoLA-2:07001 |  | 0.004096 | 11 |
| BoLA-3:05002 |  | 0.004095 | 20 |
| BoLA-3:06801 |  | 0.004038 | 22 |
| BoLA-3:01101 |  | 0.003965 | 15 |
| BoLA-2:04402 |  | 0.003962 | 15 |
| BoLA-5:07201 |  | 0.003904 | 15 |
| BoLA-1:02901 |  | 0.003563 | 13 |
| BoLA-2:00601 |  | 0.003307 | 18 |
| BoLA-2:01602 |  | 0.003307 | 18 |
| BoLA-2:05601 |  | 0.003286 | 13 |
| BoLA-T2c |  | 0.003156 | 28 |
| BoLA-6:01401 |  | 0.002987 | 15 |
| BoLA-3:01701 |  | 0.002925 | 17 |
| BoLA-2:04501 |  | 0.002868 | 23 |
| BoLA-3:05201 |  | 0.002679 | 18 |
| BoLA-3:06602 |  | 0.002669 | 27 |
| BoLA-2:00602 |  | 0.002494 | 22 |
| BoLA-2:06201 |  | 0.002445 | 26 |
| BoLA-3:05001 |  | 0.002271 | 29 |
| BoLA-3:05801 |  | 0.002061 | 35 |
| BoLA-3:06601 |  | 0.001527 | 29 |
| BoLA-3:06501 |  | 0.001467 | 43 |
| BoLA-2:04701 |  | 0.00145 | 33 |
| BoLA-2:03202 |  | 0.001428 | 24 |
| BoLA-amani.1 |  | 0.001304 | 19 |
| BoLA-2:05701 |  | 0.00123 | 34 |
| BoLA-1:01901 |  | 0.001219 | 13 |
| BoLA-1:00901 |  | 0.001215 | 22 |
| BoLA-3:07301 |  | 0.001215 | 17 |
| BoLA-2:01601 |  | 0.001156 | 20 |
| BoLA-2:04301 |  | 0.00108 | 28 |
| BoLA-2:01201 |  | 0.000919 | 30 |
| BoLA-T2a |  | 0.000919 | 30 |
| BoLA-2:05401 |  | 0.000738 | 8.7 |
| BoLA-3:00401 |  | 0.000731 | 30 |
| BoLA-3:00402 |  | 0.000731 | 30 |
| BoLA-3:00403 |  | 0.000731 | 30 |
| BoLA-3:05301 |  | 0.000731 | 30 |
| BoLA-gb1.7 |  | 0.000731 | 30 |
| BoLA-5:06401 |  | 0.000693 | 16 |
| BoLA-4:06301 |  | 0.000624 | 19 |
| BoLA-2:01801 |  | 0.000606 | 25 |
| BoLA-2:01802 |  | 0.000606 | 25 |
| BoLA-2:06001 |  | 0.000597 | 29 |
| BoLA-1:02101 |  | 0.000579 | 7.7 |
| BoLA-3:00201 |  | 0.000551 | 43 |
| BoLA-JSP.1 |  | 0.000551 | 43 |
| BoLA-1:04201 |  | 0.000498 | 35 |
| BoLA-T7 |  | 0.000488 | 35 |
| BoLA-2:02601 |  | 0.000434 | 34 |
| BoLA-2:02602 |  | 0.000434 | 34 |
| BoLA-2:02603 |  | 0.000434 | 34 |
| BoLA-1:07401 |  | 0.000425 | 20 |
| BoLA-1:02001 |  | 0.00041 | 31 |
| BoLA-2:07101 |  | 0.000402 | 41 |
| BoLA-3:05901 |  | 0.000395 | 41 |
| BoLA-2:00801 |  | 0.000392 | 18 |
| BoLA-2:04801 |  | 0.000376 | 30 |
| BoLA-2:02501 |  | 0.000352 | 31 |
| BoLA-3:00103 |  | 0.000345 | 25 |
| BoLA-1:02801 |  | 0.000284 | 25 |
| BoLA-3:05101 |  | 0.000275 | 42 |
| BoLA-1:03101 |  | 0.000271 | 52 |
| BoLA-6:01502 |  | 0.000263 | 21 |
| BoLA-3:03801 |  | 0.000246 | 52 |
| BoLA-2:04601 |  | 0.000242 | 53 |
| BoLA-1:00902 |  | 0.000227 | 37 |
| BoLA-T5 |  | 0.000227 | 37 |
| BoLA-2:03001 |  | 0.000218 | 30 |
| BoLA-3:02701 |  | 0.000214 | 44 |
| BoLA-3:02702 |  | 0.000214 | 44 |
| BoLA-5:03901 |  | 0.000213 | 41 |
| BoLA-4:02402 |  | 0.000212 | 42 |
| BoLA-1:03102 |  | 0.000209 | 55 |
| BoLA-3:01001 |  | 0.000198 | 40 |
| BoLA-6:03401 |  | 0.000196 | 26 |
| BoLA-6:01501 |  | 0.000177 | 31 |
| BoLA-2:05501 |  | 0.000174 | 52 |
| BoLA-1:02301 |  | 0.000172 | 47 |
| BoLA-D18.4 |  | 0.000172 | 47 |
| BoLA-6:04001 |  | 0.000165 | 56 |
| BoLA-2:00802 |  | 0.000163 | 26 |
| BoLA-3:00102 |  | 0.000162 | 32 |
| BoLA-2:06901 |  | 0.000158 | 36 |
| BoLA-3:00101 |  | 0.000151 | 38 |
| BoLA-AW10 |  | 0.000151 | 38 |
| BoLA-1:04901 |  | 0.000115 | 50 |
| BoLA-6:04101 |  | 8.1e-05 | 35 |
| BoLA-T2b |  | 8.1e-05 | 35 |
| BoLA-3:03501 |  | 5.9e-05 | 30 |
| BoLA-4:02401 |  | 4.1e-05 | 44 |
| BoLA-6:01301 |  | 3.1e-05 | 48 |
| BoLA-HD6 |  | 3.1e-05 | 48 |
| BoLA-2:02201 |  | 1.6e-05 | 62 |
| BoLA-6:01302 |  | 1.1e-05 | 49 |

| Alleles | Peptide | Score | Percentile rank |
| --- | --- | --- | --- |
| BoLA-6:04001 | TGMAYYMFF | 0.226722 | 0.39 |
| BoLA-3:06501 |  | 0.196531 | 2.0 |
| BoLA-2:04401 |  | 0.190493 | 1.4 |
| BoLA-3:03701 |  | 0.189139 | 1.2 |
| BoLA-2:04301 |  | 0.18766 | 0.15 |
| BoLA-3:05801 |  | 0.180707 | 0.62 |
| BoLA-3:06801 |  | 0.158698 | 1.1 |
| BoLA-3:05101 |  | 0.149571 | 0.27 |
| BoLA-2:04601 |  | 0.137738 | 0.57 |
| BoLA-T2c |  | 0.095429 | 5.9 |
| BoLA-2:04701 |  | 0.086398 | 1.7 |
| BoLA-3:00201 |  | 0.073843 | 2.9 |
| BoLA-JSP.1 |  | 0.073843 | 2.9 |
| BoLA-3:06602 |  | 0.068411 | 3.8 |
| BoLA-3:01701 |  | 0.06263 | 1.8 |
| BoLA-3:03801 |  | 0.062299 | 1.7 |
| BoLA-3:06601 |  | 0.059363 | 3.6 |
| BoLA-4:02402 |  | 0.054998 | 3.0 |
| BoLA-3:02701 |  | 0.054767 | 2.0 |
| BoLA-3:02702 |  | 0.054767 | 2.0 |
| BoLA-2:04402 |  | 0.053939 | 2.0 |
| BoLA-3:01101 |  | 0.052635 | 3.3 |
| BoLA-3:05901 |  | 0.046504 | 2.1 |
| BoLA-3:05002 |  | 0.042871 | 4.2 |
| BoLA-2:07101 |  | 0.037924 | 3.1 |
| BoLA-2:05701 |  | 0.036527 | 6.0 |
| BoLA-3:01702 |  | 0.036208 | 4.5 |
| BoLA-3:01703 |  | 0.035737 | 6.7 |
| BoLA-3:00401 |  | 0.035404 | 2.7 |
| BoLA-3:00402 |  | 0.035404 | 2.7 |
| BoLA-3:00403 |  | 0.035404 | 2.7 |
| BoLA-3:05301 |  | 0.035404 | 2.7 |
| BoLA-gb1.7 |  | 0.035404 | 2.7 |
| BoLA-2:04501 |  | 0.034416 | 4.9 |
| BoLA-4:06301 |  | 0.033597 | 1.8 |
| BoLA-1:06701 |  | 0.032734 | 8.4 |
| BoLA-amani.1 |  | 0.030578 | 3.2 |
| BoLA-5:03901 |  | 0.03037 | 2.9 |
| BoLA-3:05201 |  | 0.029306 | 4.2 |
| BoLA-5:00301 |  | 0.02784 | 13 |
| BoLA-2:06901 |  | 0.027756 | 2.3 |
| BoLA-5:07201 |  | 0.025301 | 4.3 |
| BoLA-2:06201 |  | 0.024713 | 6.8 |
| BoLA-2:05601 |  | 0.022747 | 2.9 |
| BoLA-2:03202 |  | 0.021796 | 4.9 |
| BoLA-2:07001 |  | 0.020785 | 3.3 |
| BoLA-3:03601 |  | 0.020355 | 8.3 |
| BoLA-3:05001 |  | 0.018002 | 9.5 |
| BoLA-3:00101 |  | 0.017549 | 2.1 |
| BoLA-AW10 |  | 0.017549 | 2.1 |
| BoLA-2:01201 |  | 0.015231 | 8.6 |
| BoLA-T2a |  | 0.015231 | 8.6 |
| BoLA-2:02501 |  | 0.013193 | 4.5 |
| BoLA-2:00601 |  | 0.011667 | 7.7 |
| BoLA-2:01602 |  | 0.011667 | 7.7 |
| BoLA-3:00102 |  | 0.011432 | 2.5 |
| BoLA-4:02401 |  | 0.011415 | 3.4 |
| BoLA-2:00602 |  | 0.01135 | 9.1 |
| BoLA-T7 |  | 0.011 | 11 |
| BoLA-2:00501 |  | 0.010954 | 15 |
| BoLA-3:03501 |  | 0.010839 | 1.2 |
| BoLA-2:04801 |  | 0.010459 | 6.7 |
| BoLA-2:00802 |  | 0.009672 | 3.7 |
| BoLA-3:00103 |  | 0.009657 | 3.7 |
| BoLA-1:02301 |  | 0.007844 | 12 |
| BoLA-D18.4 |  | 0.007844 | 12 |
| BoLA-1:04901 |  | 0.007091 | 12 |
| BoLA-2:06001 |  | 0.006987 | 7.9 |
| BoLA-1:03101 |  | 0.006907 | 15 |
| BoLA-2:02201 |  | 0.006285 | 5.2 |
| BoLA-2:00801 |  | 0.005932 | 4.7 |
| BoLA-2:02601 |  | 0.00588 | 12 |
| BoLA-2:02602 |  | 0.00588 | 12 |
| BoLA-2:02603 |  | 0.00588 | 12 |
| BoLA-2:05501 |  | 0.005415 | 14 |
| BoLA-5:06401 |  | 0.004711 | 5.9 |
| BoLA-1:00902 |  | 0.004055 | 12 |
| BoLA-T5 |  | 0.004055 | 12 |
| BoLA-1:00901 |  | 0.003991 | 13 |
| BoLA-1:02001 |  | 0.003545 | 13 |
| BoLA-2:03001 |  | 0.003021 | 9.5 |
| BoLA-2:01601 |  | 0.002633 | 13 |
| BoLA-1:03102 |  | 0.002542 | 23 |
| BoLA-3:01001 |  | 0.002441 | 16 |
| BoLA-1:06101 |  | 0.00189 | 26 |
| BoLA-3:07301 |  | 0.001681 | 15 |
| BoLA-2:01801 |  | 0.001398 | 18 |
| BoLA-2:01802 |  | 0.001398 | 18 |
| BoLA-2:05401 |  | 0.001243 | 6.4 |
| BoLA-6:01301 |  | 0.001033 | 18 |
| BoLA-HD6 |  | 0.001033 | 18 |
| BoLA-1:04201 |  | 0.001006 | 27 |
| BoLA-6:01302 |  | 0.000946 | 11 |
| BoLA-1:02901 |  | 0.000819 | 22 |
| BoLA-6:01402 |  | 0.000698 | 29 |
| BoLA-6:03401 |  | 0.000566 | 18 |
| BoLA-1:07401 |  | 0.000523 | 19 |
| BoLA-6:04101 |  | 0.000482 | 19 |
| BoLA-T2b |  | 0.000482 | 19 |
| BoLA-6:01501 |  | 0.000371 | 23 |
| BoLA-1:02801 |  | 0.000245 | 26 |
| BoLA-6:01502 |  | 0.000107 | 29 |
| BoLA-6:01401 |  | 0.000106 | 52 |
| BoLA-1:01901 |  | 4.1e-05 | 51 |
| BoLA-1:02101 |  | 1.5e-05 | 42 |
