# Supplementary material for: Development of a multi-epitope chimeric vaccine in silico against Babesia bovis, Theileria annulata, and Anaplasma marginale using computational biology tools and reverse vaccinology approach
Source: PLoS One. 2025 Jan 24;20(1):e0312262. doi: 10.1371/journal.pone.0312262 (PMC11759392; doi:10.1371/journal.pone.0312262)
Supplement: S7 File — (DOCX) [file pone.0312262.s013.docx]

The tables of all the ten MHC I epitopes of OMP-1 with their scores and percentile rank representing their affinities for different BOLA alleles. The peptide which has been selected for chimeric vaccine construction has been represented in bold letters. The BoLA alleles binding with the selected peptide possessing a percentile value >50 is highlighted as yellow. The BoLA allele that has bonded with the selected peptide with the lowest percentile rank is highlighted as green.

| Alleles | Peptide | Score | Percentile rank |
| --- | --- | --- | --- |
| BoLA-2:01201 | **ASGGSFEGK** | 0.682319 | 0.18 |
| BoLA-T2a |  | 0.682319 | 0.18 |
| BoLA-2:03202 |  | 0.146197 | 0.54 |
| BoLA-2:04501 |  | 0.13388 | 1.1 |
| BoLA-2:04401 |  | 0.107446 | 3.4 |
| BoLA-2:07101 |  | 0.072304 | 1.5 |
| BoLA-2:04701 |  | 0.062683 | 2.7 |
| BoLA-3:03701 |  | 0.035413 | 11 |
| BoLA-2:07001 |  | 0.032974 | 2.2 |
| BoLA-2:04601 |  | 0.025558 | 5.6 |
| BoLA-2:04402 |  | 0.021504 | 4.9 |
| BoLA-3:06601 |  | 0.012762 | 12 |
| BoLA-3:06602 |  | 0.012055 | 14 |
| BoLA-2:05501 |  | 0.010954 | 8.6 |
| BoLA-3:05101 |  | 0.010748 | 7.3 |
| BoLA-amani.1 |  | 0.010653 | 6.2 |
| BoLA-2:06201 |  | 0.009409 | 13 |
| BoLA-3:06501 |  | 0.007712 | 25 |
| BoLA-3:03601 |  | 0.006781 | 16 |
| BoLA-3:05801 |  | 0.006637 | 21 |
| BoLA-1:03102 |  | 0.005254 | 16 |
| BoLA-3:06801 |  | 0.004374 | 22 |
| BoLA-5:03901 |  | 0.003891 | 13 |
| BoLA-3:05201 |  | 0.003768 | 15 |
| BoLA-2:05601 |  | 0.003295 | 13 |
| BoLA-5:07201 |  | 0.002494 | 18 |
| BoLA-5:00301 |  | 0.002462 | 42 |
| BoLA-1:06701 |  | 0.002415 | 31 |
| BoLA-1:03101 |  | 0.002098 | 26 |
| BoLA-2:04301 |  | 0.002029 | 21 |
| BoLA-1:00902 |  | 0.001997 | 17 |
| BoLA-T5 |  | 0.001997 | 17 |
| BoLA-2:00501 |  | 0.001835 | 37 |
| BoLA-3:01703 |  | 0.001746 | 33 |
| BoLA-3:03801 |  | 0.001718 | 29 |
| BoLA-2:05701 |  | 0.001332 | 33 |
| BoLA-1:00901 |  | 0.001305 | 21 |
| BoLA-3:01101 |  | 0.001152 | 24 |
| BoLA-4:02402 |  | 0.001008 | 24 |
| BoLA-3:01702 |  | 0.000976 | 32 |
| BoLA-2:00801 |  | 0.000972 | 12 |
| BoLA-2:00602 |  | 0.000858 | 34 |
| BoLA-1:04201 |  | 0.000851 | 28 |
| BoLA-1:02001 |  | 0.000786 | 24 |
| BoLA-3:00201 |  | 0.000765 | 38 |
| BoLA-JSP.1 |  | 0.000765 | 38 |
| BoLA-3:01701 |  | 0.000724 | 31 |
| BoLA-6:01402 |  | 0.000705 | 29 |
| BoLA-3:05001 |  | 0.000563 | 48 |
| BoLA-6:04001 |  | 0.000541 | 38 |
| BoLA-2:00601 |  | 0.000527 | 37 |
| BoLA-2:01602 |  | 0.000527 | 37 |
| BoLA-2:06901 |  | 0.000509 | 24 |
| BoLA-1:02301 |  | 0.000495 | 34 |
| BoLA-D18.4 |  | 0.000495 | 34 |
| BoLA-4:06301 |  | 0.000468 | 21 |
| BoLA-5:06401 |  | 0.000446 | 19 |
| BoLA-T2c |  | 0.000431 | 45 |
| BoLA-3:03501 |  | 0.000393 | 14 |
| BoLA-2:04801 |  | 0.000386 | 30 |
| BoLA-1:06101 |  | 0.000375 | 43 |
| BoLA-3:00401 |  | 0.000323 | 40 |
| BoLA-3:00402 |  | 0.000323 | 40 |
| BoLA-3:00403 |  | 0.000323 | 40 |
| BoLA-3:05301 |  | 0.000323 | 40 |
| BoLA-gb1.7 |  | 0.000323 | 40 |
| BoLA-1:02901 |  | 0.000253 | 33 |
| BoLA-3:05901 |  | 0.000236 | 48 |
| BoLA-3:05002 |  | 0.000235 | 53 |
| BoLA-2:00802 |  | 0.00023 | 23 |
| BoLA-T7 |  | 0.000206 | 44 |
| BoLA-2:01601 |  | 0.000204 | 40 |
| BoLA-2:06001 |  | 0.000203 | 43 |
| BoLA-2:05401 |  | 0.000197 | 17 |
| BoLA-2:02601 |  | 0.000148 | 47 |
| BoLA-2:02602 |  | 0.000148 | 47 |
| BoLA-2:02603 |  | 0.000148 | 47 |
| BoLA-3:02701 |  | 0.000142 | 50 |
| BoLA-3:02702 |  | 0.000142 | 50 |
| BoLA-6:01501 |  | 0.000125 | 34 |
| BoLA-3:07301 |  | 0.00012 | 35 |
| BoLA-2:02501 |  | 0.000111 | 46 |
| BoLA-4:02401 |  | 0.000106 | 32 |
| BoLA-3:00101 |  | 0.0001 | 44 |
| BoLA-6:01401 |  | 0.0001 | 53 |
| BoLA-AW10 |  | 0.0001 | 44 |
| BoLA-2:02201 |  | 8.9e-05 | 37 |
| BoLA-3:01001 |  | 8.5e-05 | 51 |
| BoLA-6:01502 |  | 7.4e-05 | 33 |
| BoLA-1:04901 |  | 6.4e-05 | 57 |
| BoLA-3:00103 |  | 6.3e-05 | 46 |
| BoLA-2:03001 |  | 6.1e-05 | 45 |
| BoLA-2:01801 |  | 5.9e-05 | 56 |
| BoLA-2:01802 |  | 5.9e-05 | 56 |
| BoLA-3:00102 |  | 4.9e-05 | 49 |
| BoLA-1:02801 |  | 4.6e-05 | 44 |
| BoLA-1:07401 |  | 2.7e-05 | 49 |
| BoLA-1:01901 |  | 2.3e-05 | 60 |
| BoLA-6:03401 |  | 1.7e-05 | 55 |
| BoLA-1:02101 |  | 1.4e-05 | 43 |
| BoLA-6:01302 |  | 1.2e-05 | 48 |
| BoLA-6:04101 |  | 1.2e-05 | 61 |
| BoLA-T2b |  | 1.2e-05 | 61 |
| BoLA-6:01301 |  | 1e-05 | 62 |
| BoLA-HD6 |  | 1e-05 | 62 |

| Alleles | Peptide | Score | Percentile rank |
| --- | --- | --- | --- |
| BoLA-2:04601 | SGGSFEGKY | 0.300751 | 0.08 |
| BoLA-3:05101 |  | 0.282303 | 0.05 |
| BoLA-3:03701 |  | 0.278499 | 0.42 |
| BoLA-2:04401 |  | 0.241809 | 0.78 |
| BoLA-3:05801 |  | 0.241158 | 0.29 |
| BoLA-2:04701 |  | 0.188722 | 0.33 |
| BoLA-2:04301 |  | 0.187523 | 0.15 |
| BoLA-2:06201 |  | 0.182212 | 0.87 |
| BoLA-3:06801 |  | 0.118102 | 1.7 |
| BoLA-2:04402 |  | 0.086902 | 0.96 |
| BoLA-2:04501 |  | 0.068436 | 2.5 |
| BoLA-3:01703 |  | 0.060883 | 4.2 |
| BoLA-2:07101 |  | 0.056498 | 2.0 |
| BoLA-2:06901 |  | 0.054633 | 1.0 |
| BoLA-4:02402 |  | 0.052895 | 3.1 |
| BoLA-2:01201 |  | 0.051497 | 4.4 |
| BoLA-T2a |  | 0.051497 | 4.4 |
| BoLA-2:07001 |  | 0.051084 | 1.3 |
| BoLA-3:01702 |  | 0.04914 | 3.4 |
| BoLA-amani.1 |  | 0.043739 | 2.5 |
| BoLA-3:01701 |  | 0.04313 | 2.6 |
| BoLA-2:04801 |  | 0.036304 | 2.9 |
| BoLA-1:03101 |  | 0.036041 | 4.6 |
| BoLA-3:06501 |  | 0.034176 | 12 |
| BoLA-1:00902 |  | 0.031905 | 3.3 |
| BoLA-T5 |  | 0.031905 | 3.3 |
| BoLA-1:00901 |  | 0.027389 | 4.1 |
| BoLA-1:02301 |  | 0.026412 | 6.1 |
| BoLA-D18.4 |  | 0.026412 | 6.1 |
| BoLA-3:01101 |  | 0.025719 | 5.5 |
| BoLA-2:03202 |  | 0.025481 | 4.3 |
| BoLA-2:05501 |  | 0.024745 | 4.3 |
| BoLA-1:03102 |  | 0.018517 | 6.8 |
| BoLA-3:05901 |  | 0.017705 | 5.4 |
| BoLA-3:03601 |  | 0.016729 | 9.5 |
| BoLA-3:05002 |  | 0.016419 | 9.1 |
| BoLA-3:06602 |  | 0.016374 | 12 |
| BoLA-3:06601 |  | 0.014891 | 11 |
| BoLA-2:05601 |  | 0.014164 | 4.5 |
| BoLA-2:05701 |  | 0.012796 | 13 |
| BoLA-1:06701 |  | 0.012673 | 16 |
| BoLA-4:06301 |  | 0.011724 | 4.4 |
| BoLA-4:02401 |  | 0.011256 | 3.5 |
| BoLA-3:05001 |  | 0.009938 | 14 |
| BoLA-3:02701 |  | 0.008407 | 8.5 |
| BoLA-3:02702 |  | 0.008407 | 8.5 |
| BoLA-2:00802 |  | 0.008047 | 4.1 |
| BoLA-3:00401 |  | 0.008027 | 9.1 |
| BoLA-3:00402 |  | 0.008027 | 9.1 |
| BoLA-3:00403 |  | 0.008027 | 9.1 |
| BoLA-3:05301 |  | 0.008027 | 9.1 |
| BoLA-gb1.7 |  | 0.008027 | 9.1 |
| BoLA-5:00301 |  | 0.007783 | 27 |
| BoLA-3:00201 |  | 0.007709 | 14 |
| BoLA-JSP.1 |  | 0.007709 | 14 |
| BoLA-2:00501 |  | 0.007208 | 20 |
| BoLA-6:04001 |  | 0.006853 | 14 |
| BoLA-T7 |  | 0.006703 | 14 |
| BoLA-2:00602 |  | 0.006661 | 13 |
| BoLA-2:00801 |  | 0.006441 | 4.5 |
| BoLA-2:02201 |  | 0.005689 | 5.6 |
| BoLA-1:04201 |  | 0.005584 | 12 |
| BoLA-3:05201 |  | 0.004794 | 14 |
| BoLA-3:03801 |  | 0.003714 | 21 |
| BoLA-1:02001 |  | 0.00367 | 13 |
| BoLA-2:00601 |  | 0.003032 | 18 |
| BoLA-2:01602 |  | 0.003032 | 18 |
| BoLA-T2c |  | 0.003015 | 28 |
| BoLA-5:07201 |  | 0.002934 | 17 |
| BoLA-5:03901 |  | 0.002672 | 16 |
| BoLA-3:00101 |  | 0.002329 | 11 |
| BoLA-AW10 |  | 0.002329 | 11 |
| BoLA-2:03001 |  | 0.00227 | 11 |
| BoLA-2:06001 |  | 0.002262 | 16 |
| BoLA-2:02501 |  | 0.002261 | 14 |
| BoLA-1:04901 |  | 0.001715 | 22 |
| BoLA-5:06401 |  | 0.001613 | 11 |
| BoLA-3:00103 |  | 0.001351 | 14 |
| BoLA-2:05401 |  | 0.001288 | 6.3 |
| BoLA-2:01601 |  | 0.001248 | 19 |
| BoLA-1:06101 |  | 0.000996 | 33 |
| BoLA-6:03401 |  | 0.000989 | 14 |
| BoLA-1:02901 |  | 0.000952 | 21 |
| BoLA-3:00102 |  | 0.000921 | 16 |
| BoLA-3:07301 |  | 0.000858 | 19 |
| BoLA-2:02601 |  | 0.000784 | 27 |
| BoLA-2:02602 |  | 0.000784 | 27 |
| BoLA-2:02603 |  | 0.000784 | 27 |
| BoLA-2:01801 |  | 0.000706 | 24 |
| BoLA-2:01802 |  | 0.000706 | 24 |
| BoLA-3:03501 |  | 0.000628 | 11 |
| BoLA-6:01402 |  | 0.000585 | 32 |
| BoLA-6:01501 |  | 0.000532 | 20 |
| BoLA-1:02801 |  | 0.00047 | 21 |
| BoLA-3:01001 |  | 0.000441 | 31 |
| BoLA-6:01401 |  | 0.000206 | 43 |
| BoLA-1:07401 |  | 9e-05 | 35 |
| BoLA-6:01502 |  | 7.5e-05 | 33 |
| BoLA-6:04101 |  | 3.8e-05 | 45 |
| BoLA-T2b |  | 3.8e-05 | 45 |
| BoLA-1:01901 |  | 3.7e-05 | 52 |
| BoLA-6:01302 |  | 3e-05 | 37 |
| BoLA-1:02101 |  | 2.2e-05 | 37 |
| BoLA-6:01301 |  | 2.2e-05 | 52 |
| BoLA-HD6 |  | 2.2e-05 | 52 |

| Alleles | Peptide | Score | Percentile rank |
| --- | --- | --- | --- |
| BoLA-2:06201 | GGKLPGLLY | 0.404193 | 0.14 |
| BoLA-2:04601 |  | 0.353191 | 0.04 |
| BoLA-3:05101 |  | 0.291353 | 0.04 |
| BoLA-4:02402 |  | 0.286599 | 0.58 |
| BoLA-2:04401 |  | 0.281803 | 0.52 |
| BoLA-3:03701 |  | 0.232919 | 0.7 |
| BoLA-2:04701 |  | 0.228621 | 0.18 |
| BoLA-2:04301 |  | 0.222535 | 0.09 |
| BoLA-1:00901 |  | 0.212775 | 0.81 |
| BoLA-2:04501 |  | 0.210637 | 0.45 |
| BoLA-2:01201 |  | 0.198472 | 1.6 |
| BoLA-T2a |  | 0.198472 | 1.6 |
| BoLA-3:05801 |  | 0.195851 | 0.51 |
| BoLA-1:00902 |  | 0.143585 | 0.77 |
| BoLA-T5 |  | 0.143585 | 0.77 |
| BoLA-1:02301 |  | 0.14201 | 1.8 |
| BoLA-D18.4 |  | 0.14201 | 1.8 |
| BoLA-1:03101 |  | 0.137316 | 1.0 |
| BoLA-2:05501 |  | 0.135532 | 0.46 |
| BoLA-amani.1 |  | 0.134059 | 0.98 |
| BoLA-2:04402 |  | 0.131898 | 0.48 |
| BoLA-2:07101 |  | 0.126503 | 0.57 |
| BoLA-2:03202 |  | 0.126171 | 0.69 |
| BoLA-2:07001 |  | 0.093494 | 0.56 |
| BoLA-1:03102 |  | 0.091615 | 1.1 |
| BoLA-2:06901 |  | 0.087479 | 0.45 |
| BoLA-4:02401 |  | 0.083476 | 0.85 |
| BoLA-3:06801 |  | 0.069772 | 3.4 |
| BoLA-2:04801 |  | 0.050787 | 2.2 |
| BoLA-3:01701 |  | 0.045351 | 2.4 |
| BoLA-3:01703 |  | 0.043294 | 5.7 |
| BoLA-2:05701 |  | 0.04293 | 5.2 |
| BoLA-2:00802 |  | 0.039125 | 1.4 |
| BoLA-2:00801 |  | 0.037891 | 1.6 |
| BoLA-3:05002 |  | 0.032613 | 5.5 |
| BoLA-3:05001 |  | 0.031642 | 6.1 |
| BoLA-1:04201 |  | 0.030829 | 3.6 |
| BoLA-3:06601 |  | 0.029223 | 6.7 |
| BoLA-3:01702 |  | 0.029133 | 5.4 |
| BoLA-3:06602 |  | 0.026433 | 8.5 |
| BoLA-3:03601 |  | 0.024531 | 7.2 |
| BoLA-3:01101 |  | 0.022263 | 6.0 |
| BoLA-2:02201 |  | 0.019966 | 2.1 |
| BoLA-6:04001 |  | 0.01906 | 7.2 |
| BoLA-1:04901 |  | 0.018252 | 7.0 |
| BoLA-3:06501 |  | 0.017828 | 18 |
| BoLA-2:00602 |  | 0.017272 | 6.5 |
| BoLA-2:02601 |  | 0.015712 | 6.5 |
| BoLA-2:02602 |  | 0.015712 | 6.5 |
| BoLA-2:02603 |  | 0.015712 | 6.5 |
| BoLA-2:00601 |  | 0.015351 | 6.2 |
| BoLA-2:01602 |  | 0.015351 | 6.2 |
| BoLA-3:05901 |  | 0.014462 | 6.4 |
| BoLA-1:06701 |  | 0.01348 | 15 |
| BoLA-2:02501 |  | 0.012131 | 4.8 |
| BoLA-3:00401 |  | 0.011972 | 6.9 |
| BoLA-3:00402 |  | 0.011972 | 6.9 |
| BoLA-3:00403 |  | 0.011972 | 6.9 |
| BoLA-3:05301 |  | 0.011972 | 6.9 |
| BoLA-gb1.7 |  | 0.011972 | 6.9 |
| BoLA-2:06001 |  | 0.011462 | 5.4 |
| BoLA-1:02001 |  | 0.010167 | 7.0 |
| BoLA-3:02701 |  | 0.009761 | 7.7 |
| BoLA-3:02702 |  | 0.009761 | 7.7 |
| BoLA-2:00501 |  | 0.009697 | 17 |
| BoLA-5:00301 |  | 0.009327 | 25 |
| BoLA-T2c |  | 0.007373 | 21 |
| BoLA-3:05201 |  | 0.006971 | 12 |
| BoLA-1:06101 |  | 0.006464 | 16 |
| BoLA-4:06301 |  | 0.005661 | 7.2 |
| BoLA-1:02801 |  | 0.00561 | 6.3 |
| BoLA-3:00201 |  | 0.005091 | 18 |
| BoLA-JSP.1 |  | 0.005091 | 18 |
| BoLA-2:05601 |  | 0.004901 | 9.9 |
| BoLA-2:01601 |  | 0.004855 | 8.6 |
| BoLA-6:01402 |  | 0.004802 | 12 |
| BoLA-3:03801 |  | 0.004555 | 19 |
| BoLA-2:03001 |  | 0.00443 | 7.6 |
| BoLA-5:07201 |  | 0.004346 | 14 |
| BoLA-5:06401 |  | 0.003614 | 6.9 |
| BoLA-T7 |  | 0.003518 | 18 |
| BoLA-5:03901 |  | 0.003264 | 15 |
| BoLA-3:01001 |  | 0.002974 | 14 |
| BoLA-6:01302 |  | 0.002708 | 6.3 |
| BoLA-3:07301 |  | 0.002583 | 13 |
| BoLA-1:02901 |  | 0.00242 | 15 |
| BoLA-3:00101 |  | 0.001939 | 12 |
| BoLA-AW10 |  | 0.001939 | 12 |
| BoLA-2:01801 |  | 0.001864 | 16 |
| BoLA-2:01802 |  | 0.001864 | 16 |
| BoLA-6:01501 |  | 0.001796 | 12 |
| BoLA-2:05401 |  | 0.001627 | 5.4 |
| BoLA-6:01301 |  | 0.001573 | 16 |
| BoLA-HD6 |  | 0.001573 | 16 |
| BoLA-3:00103 |  | 0.0015 | 13 |
| BoLA-6:01401 |  | 0.001439 | 21 |
| BoLA-3:00102 |  | 0.001214 | 13 |
| BoLA-6:03401 |  | 0.000825 | 15 |
| BoLA-1:07401 |  | 0.000742 | 17 |
| BoLA-1:02101 |  | 0.000649 | 7.2 |
| BoLA-3:03501 |  | 0.000547 | 11 |
| BoLA-6:01502 |  | 0.000442 | 17 |
| BoLA-6:04101 |  | 0.000381 | 20 |
| BoLA-T2b |  | 0.000381 | 20 |
| BoLA-1:01901 |  | 0.00028 | 25 |

| Alleles | Peptide | Score | Percentile rank |
| --- | --- | --- | --- |
| BoLA-1:02301 | GKLPGLLYP | 0.065334 | 3.3 |
| BoLA-D18.4 |  | 0.065334 | 3.3 |
| BoLA-1:04901 |  | 0.049675 | 3.5 |
| BoLA-2:02601 |  | 0.043577 | 3.4 |
| BoLA-2:02602 |  | 0.043577 | 3.4 |
| BoLA-2:02603 |  | 0.043577 | 3.4 |
| BoLA-6:01402 |  | 0.036218 | 4.1 |
| BoLA-1:00902 |  | 0.033595 | 3.1 |
| BoLA-T5 |  | 0.033595 | 3.1 |
| BoLA-3:01703 |  | 0.030921 | 7.5 |
| BoLA-2:05501 |  | 0.030894 | 3.5 |
| BoLA-1:06101 |  | 0.025069 | 6.3 |
| BoLA-3:06501 |  | 0.019017 | 17 |
| BoLA-1:03101 |  | 0.018568 | 7.8 |
| BoLA-1:03102 |  | 0.018403 | 6.8 |
| BoLA-1:04201 |  | 0.017249 | 5.8 |
| BoLA-3:05801 |  | 0.016264 | 13 |
| BoLA-1:02001 |  | 0.013343 | 5.9 |
| BoLA-3:01702 |  | 0.012089 | 10 |
| BoLA-2:06201 |  | 0.010345 | 13 |
| BoLA-2:04701 |  | 0.009768 | 14 |
| BoLA-3:03701 |  | 0.009645 | 27 |
| BoLA-3:01101 |  | 0.009026 | 9.9 |
| BoLA-1:01901 |  | 0.008508 | 5.2 |
| BoLA-1:00901 |  | 0.008366 | 8.2 |
| BoLA-2:01601 |  | 0.008155 | 6.1 |
| BoLA-3:05001 |  | 0.007831 | 16 |
| BoLA-3:05002 |  | 0.007824 | 15 |
| BoLA-3:05901 |  | 0.00745 | 11 |
| BoLA-2:06001 |  | 0.006819 | 8.0 |
| BoLA-1:06701 |  | 0.006739 | 21 |
| BoLA-3:02701 |  | 0.006617 | 9.8 |
| BoLA-3:02702 |  | 0.006617 | 9.8 |
| BoLA-6:01401 |  | 0.006541 | 9.3 |
| BoLA-5:00301 |  | 0.006281 | 30 |
| BoLA-1:02801 |  | 0.006021 | 6.0 |
| BoLA-2:00602 |  | 0.005385 | 15 |
| BoLA-2:04401 |  | 0.005342 | 30 |
| BoLA-2:00601 |  | 0.005331 | 14 |
| BoLA-2:01602 |  | 0.005331 | 14 |
| BoLA-3:01701 |  | 0.005034 | 13 |
| BoLA-3:05101 |  | 0.004783 | 13 |
| BoLA-2:02501 |  | 0.004207 | 9.6 |
| BoLA-2:04801 |  | 0.004066 | 12 |
| BoLA-2:04601 |  | 0.004007 | 20 |
| BoLA-3:06602 |  | 0.003432 | 25 |
| BoLA-2:05701 |  | 0.00334 | 24 |
| BoLA-1:02901 |  | 0.002821 | 14 |
| BoLA-2:04301 |  | 0.002311 | 20 |
| BoLA-2:03202 |  | 0.002257 | 20 |
| BoLA-3:06601 |  | 0.001648 | 29 |
| BoLA-2:02201 |  | 0.001555 | 12 |
| BoLA-6:04001 |  | 0.001515 | 26 |
| BoLA-4:02402 |  | 0.001473 | 21 |
| BoLA-2:07101 |  | 0.001377 | 27 |
| BoLA-2:00501 |  | 0.00132 | 41 |
| BoLA-2:04501 |  | 0.001285 | 31 |
| BoLA-2:01201 |  | 0.001276 | 26 |
| BoLA-T2a |  | 0.001276 | 26 |
| BoLA-2:06901 |  | 0.001255 | 16 |
| BoLA-3:06801 |  | 0.001252 | 33 |
| BoLA-6:03401 |  | 0.001028 | 14 |
| BoLA-1:02101 |  | 0.00101 | 5.5 |
| BoLA-3:00401 |  | 0.000941 | 27 |
| BoLA-3:00402 |  | 0.000941 | 27 |
| BoLA-3:00403 |  | 0.000941 | 27 |
| BoLA-3:05301 |  | 0.000941 | 27 |
| BoLA-gb1.7 |  | 0.000941 | 27 |
| BoLA-3:01001 |  | 0.000851 | 24 |
| BoLA-6:01301 |  | 0.00081 | 19 |
| BoLA-HD6 |  | 0.00081 | 19 |
| BoLA-3:03601 |  | 0.000542 | 42 |
| BoLA-T7 |  | 0.000541 | 34 |
| BoLA-2:04402 |  | 0.000509 | 35 |
| BoLA-5:07201 |  | 0.0005 | 31 |
| BoLA-2:05601 |  | 0.000482 | 29 |
| BoLA-3:00201 |  | 0.000478 | 45 |
| BoLA-JSP.1 |  | 0.000478 | 45 |
| BoLA-4:02401 |  | 0.000475 | 17 |
| BoLA-1:07401 |  | 0.000473 | 20 |
| BoLA-3:03501 |  | 0.000458 | 13 |
| BoLA-6:04101 |  | 0.00042 | 20 |
| BoLA-T2b |  | 0.00042 | 20 |
| BoLA-3:05201 |  | 0.00041 | 33 |

| Alleles | Peptide | Score | Percentile rank |
| --- | --- | --- | --- |
| BoLA-2:01801 | LPGLLYPQA | 0.089075 | 1.6 |
| BoLA-2:01802 |  | 0.089075 | 1.6 |
| BoLA-2:00501 |  | 0.088842 | 1.9 |
| BoLA-3:01701 |  | 0.051515 | 2.1 |
| BoLA-3:01702 |  | 0.043167 | 3.9 |
| BoLA-3:05001 |  | 0.039708 | 5.0 |
| BoLA-3:01703 |  | 0.03677 | 6.6 |
| BoLA-3:05002 |  | 0.030377 | 5.8 |
| BoLA-3:01001 |  | 0.018111 | 4.1 |
| BoLA-T2c |  | 0.016203 | 16 |
| BoLA-1:06701 |  | 0.011228 | 16 |
| BoLA-2:03001 |  | 0.011064 | 4.0 |
| BoLA-2:00601 |  | 0.008735 | 9.6 |
| BoLA-2:01602 |  | 0.008735 | 9.6 |
| BoLA-2:00602 |  | 0.00786 | 12 |
| BoLA-3:03701 |  | 0.007366 | 31 |
| BoLA-5:00301 |  | 0.006301 | 30 |
| BoLA-3:03801 |  | 0.004099 | 20 |
| BoLA-3:00401 |  | 0.003909 | 14 |
| BoLA-3:00402 |  | 0.003909 | 14 |
| BoLA-3:00403 |  | 0.003909 | 14 |
| BoLA-3:05301 |  | 0.003909 | 14 |
| BoLA-gb1.7 |  | 0.003909 | 14 |
| BoLA-3:06501 |  | 0.003596 | 33 |
| BoLA-6:01401 |  | 0.003388 | 14 |
| BoLA-3:05801 |  | 0.003233 | 29 |
| BoLA-1:06101 |  | 0.002925 | 22 |
| BoLA-3:06801 |  | 0.002797 | 26 |
| BoLA-6:01402 |  | 0.002734 | 16 |
| BoLA-2:05701 |  | 0.002732 | 26 |
| BoLA-1:01901 |  | 0.002714 | 8.9 |
| BoLA-3:05901 |  | 0.002007 | 21 |
| BoLA-2:06201 |  | 0.001974 | 28 |
| BoLA-2:04601 |  | 0.00175 | 28 |
| BoLA-3:00201 |  | 0.001601 | 29 |
| BoLA-JSP.1 |  | 0.001601 | 29 |
| BoLA-2:06001 |  | 0.001579 | 19 |
| BoLA-2:04401 |  | 0.001465 | 46 |
| BoLA-3:05101 |  | 0.001434 | 23 |
| BoLA-3:03601 |  | 0.00127 | 32 |
| BoLA-1:02001 |  | 0.001135 | 21 |
| BoLA-3:02701 |  | 0.001051 | 24 |
| BoLA-3:02702 |  | 0.001051 | 24 |
| BoLA-6:03401 |  | 0.001048 | 14 |
| BoLA-2:04301 |  | 0.001032 | 28 |
| BoLA-2:05601 |  | 0.001008 | 22 |
| BoLA-1:04901 |  | 0.001 | 26 |
| BoLA-2:02501 |  | 0.000973 | 20 |
| BoLA-2:03202 |  | 0.000957 | 28 |
| BoLA-2:01601 |  | 0.000862 | 23 |
| BoLA-2:02601 |  | 0.000827 | 27 |
| BoLA-2:02602 |  | 0.000827 | 27 |
| BoLA-2:02603 |  | 0.000827 | 27 |
| BoLA-2:04701 |  | 0.000771 | 41 |
| BoLA-5:07201 |  | 0.000713 | 28 |
| BoLA-5:03901 |  | 0.00071 | 27 |
| BoLA-3:01101 |  | 0.000625 | 30 |
| BoLA-2:07001 |  | 0.000548 | 26 |
| BoLA-2:04801 |  | 0.000513 | 27 |
| BoLA-3:00101 |  | 0.000491 | 24 |
| BoLA-AW10 |  | 0.000491 | 24 |
| BoLA-6:04001 |  | 0.000472 | 40 |
| BoLA-1:07401 |  | 0.000432 | 20 |
| BoLA-4:06301 |  | 0.000425 | 22 |
| BoLA-2:04501 |  | 0.000388 | 46 |
| BoLA-2:01201 |  | 0.000372 | 41 |
| BoLA-T2a |  | 0.000372 | 41 |
| BoLA-2:07101 |  | 0.000371 | 42 |
| BoLA-3:00103 |  | 0.000366 | 25 |
| BoLA-3:07301 |  | 0.000346 | 25 |
| BoLA-T7 |  | 0.000336 | 38 |
| BoLA-1:04201 |  | 0.000332 | 39 |
| BoLA-4:02402 |  | 0.000307 | 37 |
| BoLA-3:06602 |  | 0.000299 | 54 |
| BoLA-3:05201 |  | 0.000295 | 37 |
| BoLA-2:06901 |  | 0.000286 | 29 |
| BoLA-2:05501 |  | 0.000248 | 47 |
| BoLA-1:02901 |  | 0.000234 | 34 |
| BoLA-3:06601 |  | 0.000219 | 53 |
| BoLA-3:00102 |  | 0.000206 | 30 |
| BoLA-1:03101 |  | 0.000192 | 56 |
| BoLA-1:02101 |  | 0.000178 | 15 |
| BoLA-1:03102 |  | 0.000178 | 57 |
| BoLA-4:02401 |  | 0.000178 | 26 |
| BoLA-6:01501 |  | 0.000167 | 31 |
| BoLA-2:04402 |  | 0.000166 | 49 |
| BoLA-1:02301 |  | 0.000138 | 50 |
| BoLA-D18.4 |  | 0.000138 | 50 |
| BoLA-6:04101 |  | 0.000135 | 30 |
| BoLA-T2b |  | 0.000135 | 30 |
| BoLA-1:00901 |  | 0.000121 | 49 |
| BoLA-amani.1 |  | 7.5e-05 | 50 |
| BoLA-1:00902 |  | 7.1e-05 | 52 |
| BoLA-T5 |  | 7.1e-05 | 52 |
| BoLA-2:02201 |  | 5.9e-05 | 43 |
| BoLA-5:06401 |  | 4.6e-05 | 40 |
| BoLA-1:02801 |  | 4.4e-05 | 45 |
| BoLA-3:03501 |  | 4.4e-05 | 33 |
| BoLA-6:01502 |  | 3.8e-05 | 40 |
| BoLA-6:01301 |  | 2.2e-05 | 52 |
| BoLA-HD6 |  | 2.2e-05 | 52 |
| BoLA-6:01302 |  | 1.3e-05 | 47 |
| BoLA-2:05401 |  | 9e-06 | 51 |
| BoLA-2:00801 |  | 8e-06 | 66 |
| BoLA-2:00802 |  | 8e-06 | 68 |

| Alleles | Peptide | Score | Percentile rank |
| --- | --- | --- | --- |
| BoLA-6:04001 | AGGKLPGLL | 0.46444 | 0.08 |
| BoLA-3:06501 |  | 0.294495 | 0.81 |
| BoLA-3:00201 |  | 0.282112 | 0.48 |
| BoLA-JSP.1 |  | 0.282112 | 0.48 |
| BoLA-3:01101 |  | 0.252595 | 0.59 |
| BoLA-3:01703 |  | 0.239499 | 0.62 |
| BoLA-3:01702 |  | 0.22038 | 0.44 |
| BoLA-3:06602 |  | 0.157904 | 1.3 |
| BoLA-3:01701 |  | 0.15606 | 0.64 |
| BoLA-3:05801 |  | 0.127877 | 1.4 |
| BoLA-5:00301 |  | 0.123598 | 2.7 |
| BoLA-3:06601 |  | 0.122442 | 1.6 |
| BoLA-3:06801 |  | 0.103809 | 2.0 |
| BoLA-1:06701 |  | 0.087215 | 3.5 |
| BoLA-2:04601 |  | 0.079536 | 1.6 |
| BoLA-2:04701 |  | 0.076831 | 2.0 |
| BoLA-3:05101 |  | 0.067634 | 1.2 |
| BoLA-3:00101 |  | 0.061167 | 0.37 |
| BoLA-AW10 |  | 0.061167 | 0.37 |
| BoLA-3:03701 |  | 0.059399 | 6.3 |
| BoLA-5:07201 |  | 0.055552 | 1.8 |
| BoLA-3:05201 |  | 0.053735 | 2.4 |
| BoLA-3:00102 |  | 0.04586 | 0.39 |
| BoLA-3:00401 |  | 0.041129 | 2.3 |
| BoLA-3:00402 |  | 0.041129 | 2.3 |
| BoLA-3:00403 |  | 0.041129 | 2.3 |
| BoLA-3:05301 |  | 0.041129 | 2.3 |
| BoLA-gb1.7 |  | 0.041129 | 2.3 |
| BoLA-3:03801 |  | 0.040537 | 3.1 |
| BoLA-2:04401 |  | 0.034834 | 11 |
| BoLA-3:03601 |  | 0.032382 | 5.8 |
| BoLA-3:00103 |  | 0.03207 | 1.1 |
| BoLA-5:03901 |  | 0.028028 | 3.1 |
| BoLA-2:00501 |  | 0.027746 | 7.1 |
| BoLA-4:06301 |  | 0.026549 | 2.3 |
| BoLA-3:03501 |  | 0.026319 | 0.4 |
| BoLA-3:07301 |  | 0.026082 | 3.2 |
| BoLA-3:05002 |  | 0.024499 | 6.9 |
| BoLA-T2c |  | 0.024242 | 13 |
| BoLA-3:05901 |  | 0.023813 | 4.2 |
| BoLA-T7 |  | 0.021639 | 6.6 |
| BoLA-2:05701 |  | 0.019978 | 9.3 |
| BoLA-3:02701 |  | 0.018029 | 5.2 |
| BoLA-3:02702 |  | 0.018029 | 5.2 |
| BoLA-2:05601 |  | 0.016999 | 3.8 |
| BoLA-2:04301 |  | 0.01632 | 5.8 |
| BoLA-2:04402 |  | 0.015951 | 6.3 |
| BoLA-6:01501 |  | 0.014904 | 2.7 |
| BoLA-2:06201 |  | 0.014484 | 9.8 |
| BoLA-2:04801 |  | 0.014135 | 5.6 |
| BoLA-5:06401 |  | 0.013976 | 2.6 |
| BoLA-6:01301 |  | 0.013036 | 6.3 |
| BoLA-HD6 |  | 0.013036 | 6.3 |
| BoLA-3:05001 |  | 0.012851 | 12 |
| BoLA-2:01801 |  | 0.011181 | 6.5 |
| BoLA-2:01802 |  | 0.011181 | 6.5 |
| BoLA-1:02901 |  | 0.011175 | 6.6 |
| BoLA-2:07101 |  | 0.009066 | 11 |
| BoLA-1:06101 |  | 0.00833 | 14 |
| BoLA-2:07001 |  | 0.00786 | 7.2 |
| BoLA-1:03102 |  | 0.007309 | 13 |
| BoLA-2:02601 |  | 0.007286 | 10 |
| BoLA-2:02602 |  | 0.007286 | 10 |
| BoLA-2:02603 |  | 0.007286 | 10 |
| BoLA-1:02301 |  | 0.007183 | 12 |
| BoLA-D18.4 |  | 0.007183 | 12 |
| BoLA-2:03001 |  | 0.006816 | 5.7 |
| BoLA-1:03101 |  | 0.006621 | 15 |
| BoLA-2:06901 |  | 0.006603 | 7.1 |
| BoLA-2:00601 |  | 0.006555 | 12 |
| BoLA-2:01602 |  | 0.006555 | 12 |
| BoLA-2:00602 |  | 0.006493 | 14 |
| BoLA-1:02801 |  | 0.006183 | 5.9 |
| BoLA-1:04901 |  | 0.005325 | 14 |
| BoLA-2:04501 |  | 0.005106 | 18 |
| BoLA-2:01201 |  | 0.004984 | 15 |
| BoLA-T2a |  | 0.004984 | 15 |
| BoLA-4:02402 |  | 0.004913 | 13 |
| BoLA-2:06001 |  | 0.004864 | 10 |
| BoLA-2:03202 |  | 0.004527 | 14 |
| BoLA-amani.1 |  | 0.004392 | 11 |
| BoLA-2:05501 |  | 0.004384 | 16 |
| BoLA-2:02501 |  | 0.00432 | 9.4 |
| BoLA-2:05401 |  | 0.004225 | 2.7 |
| BoLA-3:01001 |  | 0.004221 | 12 |
| BoLA-6:01502 |  | 0.003838 | 5.0 |
| BoLA-1:07401 |  | 0.002396 | 9.5 |
| BoLA-6:01302 |  | 0.002125 | 7.1 |
| BoLA-1:00902 |  | 0.00195 | 17 |
| BoLA-T5 |  | 0.00195 | 17 |
| BoLA-1:00901 |  | 0.00149 | 20 |
| BoLA-6:04101 |  | 0.001245 | 12 |
| BoLA-T2b |  | 0.001245 | 12 |
| BoLA-2:02201 |  | 0.001142 | 14 |
| BoLA-2:01601 |  | 0.00108 | 20 |
| BoLA-6:01401 |  | 0.001018 | 24 |
| BoLA-4:02401 |  | 0.000965 | 13 |
| BoLA-2:00801 |  | 0.000938 | 12 |
| BoLA-6:03401 |  | 0.000864 | 15 |
| BoLA-1:04201 |  | 0.000768 | 30 |
| BoLA-2:00802 |  | 0.000664 | 15 |
| BoLA-6:01402 |  | 0.000409 | 36 |
| BoLA-1:02001 |  | 0.000267 | 36 |
| BoLA-1:01901 |  | 0.000128 | 34 |
| BoLA-1:02101 |  | 6e-05 | 25 |

| Alleles | Peptide | Score | Percentila rank |
| --- | --- | --- | --- |
| BoLA-3:01703 | GSFEGKYSP | 0.168339 | 1.3 |
| BoLA-1:06101 |  | 0.12839 | 1.1 |
| BoLA-3:01702 |  | 0.123084 | 1.2 |
| BoLA-1:06701 |  | 0.093907 | 3.3 |
| BoLA-5:00301 |  | 0.083298 | 4.7 |
| BoLA-6:01402 |  | 0.06648 | 2.8 |
| BoLA-3:05001 |  | 0.066127 | 3.1 |
| BoLA-3:05002 |  | 0.056325 | 3.1 |
| BoLA-3:03701 |  | 0.055199 | 6.8 |
| BoLA-3:06801 |  | 0.04998 | 4.9 |
| BoLA-2:00501 |  | 0.049725 | 4.0 |
| BoLA-3:01701 |  | 0.049009 | 2.2 |
| BoLA-2:04401 |  | 0.044381 | 8.5 |
| BoLA-3:06602 |  | 0.042062 | 6.0 |
| BoLA-3:06501 |  | 0.041448 | 11 |
| BoLA-3:01101 |  | 0.035497 | 4.4 |
| BoLA-3:06601 |  | 0.032725 | 6.1 |
| BoLA-6:01401 |  | 0.032559 | 3.1 |
| BoLA-1:00901 |  | 0.030655 | 3.8 |
| BoLA-2:03202 |  | 0.030573 | 3.7 |
| BoLA-2:04501 |  | 0.029951 | 5.6 |
| BoLA-T2c |  | 0.028962 | 12 |
| BoLA-2:05701 |  | 0.028848 | 7.2 |
| BoLA-2:05701 |  | 0.028848 | 7.2 |
| BoLA-2:07001 |  | 0.028281 | 2.5 |
| BoLA-1:02301 |  | 0.027425 | 5.9 |
| BoLA-D18.4 |  | 0.027425 | 5.9 |
| BoLA-2:06201 |  | 0.027284 | 6.3 |
| BoLA-3:05801 |  | 0.027188 | 8.4 |
| BoLA-3:03601 |  | 0.024353 | 7.3 |
| BoLA-5:07201 |  | 0.023208 | 4.6 |
| BoLA-amani.1 |  | 0.023149 | 3.9 |
| BoLA-3:05201 |  | 0.021538 | 5.4 |
| BoLA-2:05601 |  | 0.021265 | 3.1 |
| BoLA-2:04402 |  | 0.020026 | 5.2 |
| BoLA-2:04701 |  | 0.018455 | 8.8 |
| BoLA-2:01201 |  | 0.018184 | 7.9 |
| BoLA-T2a |  | 0.018184 | 7.9 |
| BoLA-2:07101 |  | 0.018183 | 6.3 |
| BoLA-1:00902 |  | 0.014946 | 5.5 |
| BoLA-T5 |  | 0.014946 | 5.5 |
| BoLA-1:01901 |  | 0.014259 | 4.0 |
| BoLA-3:00401 |  | 0.013814 | 6.2 |
| BoLA-3:00402 |  | 0.013814 | 6.2 |
| BoLA-3:00403 |  | 0.013814 | 6.2 |
| BoLA-3:05301 |  | 0.013814 | 6.2 |
| BoLA-gb1.7 |  | 0.013814 | 6.2 |
| BoLA-3:05901 |  | 0.012372 | 7.2 |
| BoLA-3:07301 |  | 0.012219 | 5.5 |
| BoLA-2:00601 |  | 0.0116 | 7.8 |
| BoLA-2:01602 |  | 0.0116 | 7.8 |
| BoLA-5:03901 |  | 0.011476 | 6.8 |
| BoLA-2:00602 |  | 0.009491 | 11 |
| BoLA-2:04601 |  | 0.008258 | 13 |
| BoLA-1:02901 |  | 0.007835 | 8.1 |
| BoLA-2:04301 |  | 0.007345 | 11 |
| BoLA-1:02001 |  | 0.007333 | 8.4 |
| BoLA-2:02601 |  | 0.007237 | 10 |
| BoLA-2:02602 |  | 0.007237 | 10 |
| BoLA-2:02603 |  | 0.007237 | 10 |
| BoLA-3:05101 |  | 0.00723 | 9.6 |
| BoLA-1:04201 |  | 0.007076 | 11 |
| BoLA-1:03101 |  | 0.00645 | 15 |
| BoLA-2:06001 |  | 0.006022 | 8.7 |
| BoLA-2:01601 |  | 0.005965 | 7.5 |
| BoLA-1:03102 |  | 0.005462 | 16 |
| BoLA-1:04901 |  | 0.005455 | 14 |
| BoLA-2:02501 |  | 0.005422 | 8.3 |
| BoLA-3:02701 |  | 0.005273 | 11 |
| BoLA-3:02702 |  | 0.005273 | 11 |
| BoLA-2:05501 |  | 0.004876 | 15 |
| BoLA-3:00101 |  | 0.004857 | 6.5 |
| BoLA-AW10 |  | 0.004857 | 6.5 |
| BoLA-4:06301 |  | 0.004603 | 8.0 |
| BoLA-3:00102 |  | 0.004601 | 5.5 |
| BoLA-3:00201 |  | 0.004544 | 19 |
| BoLA-JSP.1 |  | 0.004544 | 19 |
| BoLA-4:02402 |  | 0.004168 | 14 |
| BoLA-2:05401 |  | 0.003972 | 2.9 |
| BoLA-1:02801 |  | 0.003905 | 7.8 |
| BoLA-2:06901 |  | 0.003751 | 9.6 |
| BoLA-6:01501 |  | 0.003388 | 7.9 |
| BoLA-6:01301 |  | 0.003323 | 12 |
| BoLA-HD6 |  | 0.003323 | 12 |
| BoLA-3:03801 |  | 0.002831 | 23 |
| BoLA-2:04801 |  | 0.002791 | 14 |
| BoLA-2:01801 |  | 0.00262 | 14 |
| BoLA-2:01802 |  | 0.00262 | 14 |
| BoLA-3:01001 |  | 0.002531 | 15 |
| BoLA-2:03001 |  | 0.0024 | 11 |
| BoLA-T7 |  | 0.002386 | 21 |
| BoLA-3:00103 |  | 0.002279 | 11 |
| BoLA-3:03501 |  | 0.002263 | 4.8 |
| BoLA-6:04001 |  | 0.002148 | 23 |
| BoLA-6:01502 |  | 0.002046 | 7.5 |
| BoLA-4:02401 |  | 0.001564 | 10 |
| BoLA-1:07401 |  | 0.001504 | 12 |
| BoLA-2:00801 |  | 0.001488 | 9.6 |
| BoLA-1:02101 |  | 0.001484 | 4.3 |
| BoLA-5:06401 |  | 0.00144 | 12 |
| BoLA-2:02201 |  | 0.001196 | 14 |
| BoLA-6:04101 |  | 0.00106 | 13 |
| BoLA-T2b |  | 0.00106 | 13 |
| BoLA-6:03401 |  | 0.000912 | 14 |
| BoLA-2:00802 |  | 0.000858 | 13 |
| BoLA-6:01302 |  | 0.000433 | 15 |

| Alleles | Peptide | Score | Percentile rank |
| --- | --- | --- | --- |
| BoLA-6:01402 | AQAAGGKLP | 0.279075 | 0.98 |
| BoLA-1:06101 |  | 0.236566 | 0.34 |
| BoLA-6:01401 |  | 0.128695 | 0.86 |
| BoLA-1:02301 |  | 0.047977 | 4.1 |
| BoLA-D18.4 |  | 0.047977 | 4.1 |
| BoLA-1:00901 |  | 0.04107 | 3.1 |
| BoLA-3:01703 |  | 0.038181 | 6.3 |
| BoLA-1:02901 |  | 0.034608 | 2.9 |
| BoLA-5:00301 |  | 0.03285 | 12 |
| BoLA-2:01601 |  | 0.03251 | 1.8 |
| BoLA-1:00902 |  | 0.030736 | 3.4 |
| BoLA-T5 |  | 0.030736 | 3.4 |
| BoLA-2:00602 |  | 0.027525 | 4.3 |
| BoLA-1:02801 |  | 0.027014 | 2.0 |
| BoLA-1:01901 |  | 0.026521 | 2.9 |
| BoLA-1:03101 |  | 0.025984 | 6.1 |
| BoLA-1:02101 |  | 0.024437 | 0.39 |
| BoLA-2:00601 |  | 0.024374 | 4.0 |
| BoLA-2:01602 |  | 0.024374 | 4.0 |
| BoLA-2:06201 |  | 0.023376 | 7.0 |
| BoLA-3:01702 |  | 0.021722 | 6.8 |
| BoLA-1:03102 |  | 0.021677 | 6.0 |
| BoLA-1:04201 |  | 0.02111 | 5.0 |
| BoLA-2:04401 |  | 0.020126 | 16 |
| BoLA-6:01501 |  | 0.018503 | 2.2 |
| BoLA-1:02001 |  | 0.015216 | 5.4 |
| BoLA-1:06701 |  | 0.014883 | 14 |
| BoLA-3:06501 |  | 0.013224 | 20 |
| BoLA-1:07401 |  | 0.013221 | 3.3 |
| BoLA-6:03401 |  | 0.012367 | 3.4 |
| BoLA-2:04301 |  | 0.011944 | 7.3 |
| BoLA-2:06001 |  | 0.011074 | 5.6 |
| BoLA-1:04901 |  | 0.010686 | 9.5 |
| BoLA-2:05501 |  | 0.010147 | 9.1 |
| BoLA-2:05701 |  | 0.009019 | 15 |
| BoLA-2:02601 |  | 0.008879 | 8.9 |
| BoLA-2:02602 |  | 0.008879 | 8.9 |
| BoLA-2:02603 |  | 0.008879 | 8.9 |
| BoLA-6:01301 |  | 0.008617 | 7.6 |
| BoLA-HD6 |  | 0.008617 | 7.6 |
| BoLA-2:04701 |  | 0.008445 | 15 |
| BoLA-3:06602 |  | 0.008415 | 17 |
| BoLA-3:01101 |  | 0.007965 | 11 |
| BoLA-6:01502 |  | 0.00741 | 3.1 |
| BoLA-3:05001 |  | 0.007102 | 17 |
| BoLA-3:01701 |  | 0.006579 | 11 |
| BoLA-3:03701 |  | 0.006524 | 32 |
| BoLA-3:05002 |  | 0.006502 | 16 |
| BoLA-2:00501 |  | 0.006019 | 22 |
| BoLA-6:04101 |  | 0.005839 | 5.3 |
| BoLA-T2b |  | 0.005839 | 5.3 |
| BoLA-2:02501 |  | 0.005639 | 8.1 |
| BoLA-5:07201 |  | 0.004753 | 13 |
| BoLA-3:06601 |  | 0.004577 | 19 |
| BoLA-2:04601 |  | 0.003951 | 20 |
| BoLA-3:05801 |  | 0.003805 | 27 |
| BoLA-4:02402 |  | 0.003334 | 15 |
| BoLA-2:04402 |  | 0.002742 | 18 |
| BoLA-2:04501 |  | 0.002384 | 25 |
| BoLA-5:06401 |  | 0.001963 | 9.6 |
| BoLA-3:06801 |  | 0.00195 | 29 |
| BoLA-3:05901 |  | 0.001791 | 22 |
| BoLA-3:03601 |  | 0.001777 | 28 |
| BoLA-3:05101 |  | 0.001723 | 21 |
| BoLA-2:01801 |  | 0.001546 | 18 |
| BoLA-2:01802 |  | 0.001546 | 18 |
| BoLA-2:04801 |  | 0.00148 | 18 |
| BoLA-3:02701 |  | 0.001388 | 22 |
| BoLA-3:02702 |  | 0.001388 | 22 |
| BoLA-3:07301 |  | 0.001291 | 16 |
| BoLA-6:04001 |  | 0.001272 | 28 |
| BoLA-2:03001 |  | 0.001124 | 16 |
| BoLA-3:00401 |  | 0.001109 | 25 |
| BoLA-3:00402 |  | 0.001109 | 25 |
| BoLA-3:00403 |  | 0.001109 | 25 |
| BoLA-3:05301 |  | 0.001109 | 25 |
| BoLA-gb1.7 |  | 0.001109 | 25 |
| BoLA-T2c |  | 0.001087 | 37 |
| BoLA-3:05201 |  | 0.001075 | 25 |
| BoLA-2:05401 |  | 0.000939 | 7.5 |
| BoLA-2:07101 |  | 0.000881 | 32 |
| BoLA-2:03202 |  | 0.000847 | 30 |
| BoLA-3:00201 |  | 0.000845 | 37 |
| BoLA-JSP.1 |  | 0.000845 | 37 |
| BoLA-3:01001 |  | 0.0008 | 25 |
| BoLA-2:06901 |  | 0.000779 | 20 |
| BoLA-2:00802 |  | 0.000662 | 15 |
| BoLA-6:01302 |  | 0.000616 | 13 |
| BoLA-2:00801 |  | 0.00053 | 16 |
| BoLA-2:07001 |  | 0.000527 | 27 |
| BoLA-3:00102 |  | 0.000521 | 20 |
| BoLA-2:05601 |  | 0.000514 | 29 |
| BoLA-3:03801 |  | 0.000496 | 43 |
| BoLA-5:03901 |  | 0.000447 | 32 |
| BoLA-2:02201 |  | 0.000431 | 21 |
| BoLA-4:02401 |  | 0.000424 | 18 |
| BoLA-2:01201 |  | 0.000345 | 42 |
| BoLA-T2a |  | 0.000345 | 42 |
| BoLA-3:00103 |  | 0.000333 | 26 |
| BoLA-amani.1 |  | 0.000331 | 32 |
| BoLA-T7 |  | 0.000279 | 40 |
| BoLA-4:06301 |  | 0.000235 | 26 |
| BoLA-3:00101 |  | 0.000221 | 33 |
| BoLA-AW10 |  | 0.000221 | 33 |
| BoLA-3:03501 |  | 0.000145 | 21 |

| Alleles | Peptide | Score | Percentile rank |
| --- | --- | --- | --- |
| BoLA-6:01402 | AEAPPAKGP | 0.952347 | 0.01 |
| BoLA-1:01901 |  | 0.637873 | 0.13 |
| BoLA-6:01401 |  | 0.505514 | 0.04 |
| BoLA-1:02001 |  | 0.283764 | 0.24 |
| BoLA-1:04201 |  | 0.127329 | 0.72 |
| BoLA-2:01601 |  | 0.120684 | 0.29 |
| BoLA-1:02901 |  | 0.09515 | 0.98 |
| BoLA-6:03401 |  | 0.08915 | 0.61 |
| BoLA-1:06101 |  | 0.071023 | 2.4 |
| BoLA-2:00601 |  | 0.067316 | 1.1 |
| BoLA-2:01602 |  | 0.067316 | 1.1 |
| BoLA-1:07401 |  | 0.065503 | 0.7 |
| BoLA-1:02101 |  | 0.063044 | 0.13 |
| BoLA-2:00602 |  | 0.059418 | 1.8 |
| BoLA-2:06001 |  | 0.042894 | 1.3 |
| BoLA-1:02801 |  | 0.023304 | 2.3 |
| BoLA-1:03101 |  | 0.021978 | 6.9 |
| BoLA-6:04101 |  | 0.021195 | 2.3 |
| BoLA-T2b |  | 0.021195 | 2.3 |
| BoLA-1:03102 |  | 0.018421 | 6.8 |
| BoLA-3:01703 |  | 0.018099 | 11 |
| BoLA-1:04901 |  | 0.013052 | 8.5 |
| BoLA-2:04701 |  | 0.011979 | 12 |
| BoLA-3:05001 |  | 0.011939 | 13 |
| BoLA-3:01702 |  | 0.011281 | 11 |
| BoLA-3:03701 |  | 0.011041 | 25 |
| BoLA-2:06201 |  | 0.010567 | 12 |
| BoLA-6:01501 |  | 0.010291 | 3.6 |
| BoLA-2:05501 |  | 0.009985 | 9.2 |
| BoLA-3:06501 |  | 0.009354 | 23 |
| BoLA-1:02301 |  | 0.009223 | 11 |
| BoLA-D18.4 |  | 0.009223 | 11 |
| BoLA-2:04401 |  | 0.009003 | 24 |
| BoLA-3:05002 |  | 0.008921 | 14 |
| BoLA-3:06602 |  | 0.00871 | 17 |
| BoLA-1:06701 |  | 0.00868 | 19 |
| BoLA-3:01101 |  | 0.008136 | 11 |
| BoLA-3:01701 |  | 0.00709 | 11 |
| BoLA-2:04301 |  | 0.006679 | 11 |
| BoLA-1:00901 |  | 0.006025 | 9.7 |
| BoLA-3:06801 |  | 0.005998 | 19 |
| BoLA-6:01502 |  | 0.005781 | 3.8 |
| BoLA-3:06601 |  | 0.005617 | 18 |
| BoLA-1:00902 |  | 0.005548 | 9.7 |
| BoLA-T5 |  | 0.005548 | 9.7 |
| BoLA-2:02501 |  | 0.005426 | 8.3 |
| BoLA-2:02601 |  | 0.005095 | 12 |
| BoLA-2:02602 |  | 0.005095 | 12 |
| BoLA-2:02603 |  | 0.005095 | 12 |
| BoLA-5:00301 |  | 0.004928 | 33 |
| BoLA-2:04402 |  | 0.004926 | 14 |
| BoLA-3:05801 |  | 0.004226 | 26 |
| BoLA-3:03801 |  | 0.004116 | 20 |
| BoLA-2:00501 |  | 0.003667 | 28 |
| BoLA-3:05901 |  | 0.003647 | 16 |
| BoLA-2:05701 |  | 0.003528 | 23 |
| BoLA-2:01801 |  | 0.00343 | 13 |
| BoLA-2:01802 |  | 0.00343 | 13 |
| BoLA-4:02402 |  | 0.002399 | 17 |
| BoLA-3:01001 |  | 0.002336 | 16 |
| BoLA-3:05201 |  | 0.002287 | 19 |
| BoLA-3:02701 |  | 0.002069 | 18 |
| BoLA-3:02702 |  | 0.002069 | 18 |
| BoLA-2:04601 |  | 0.00206 | 26 |
| BoLA-2:03001 |  | 0.00186 | 13 |
| BoLA-3:07301 |  | 0.001774 | 15 |
| BoLA-2:04801 |  | 0.001711 | 17 |
| BoLA-2:04501 |  | 0.001606 | 29 |
| BoLA-2:02201 |  | 0.001505 | 12 |
| BoLA-3:03601 |  | 0.001301 | 32 |
| BoLA-2:07101 |  | 0.001263 | 28 |
| BoLA-2:00802 |  | 0.001103 | 12 |
| BoLA-3:00401 |  | 0.001036 | 26 |
| BoLA-3:00402 |  | 0.001036 | 26 |
| BoLA-3:00403 |  | 0.001036 | 26 |
| BoLA-3:05301 |  | 0.001036 | 26 |
| BoLA-gb1.7 |  | 0.001036 | 26 |
| BoLA-T2c |  | 0.000833 | 39 |
| BoLA-6:01301 |  | 0.000809 | 19 |
| BoLA-HD6 |  | 0.000809 | 19 |
| BoLA-3:05101 |  | 0.000571 | 33 |
| BoLA-2:06901 |  | 0.000545 | 23 |
| BoLA-2:05401 |  | 0.000514 | 11 |
| BoLA-4:02401 |  | 0.000509 | 17 |
| BoLA-6:04001 |  | 0.000477 | 40 |
| BoLA-5:07201 |  | 0.000475 | 31 |
| BoLA-2:07001 |  | 0.000458 | 28 |
| BoLA-2:05601 |  | 0.000455 | 30 |
| BoLA-6:01302 |  | 0.000448 | 15 |
| BoLA-2:03202 |  | 0.000401 | 39 |
| BoLA-5:03901 |  | 0.000392 | 34 |
| BoLA-2:01201 |  | 0.00028 | 44 |
| BoLA-amani.1 |  | 0.00028 | 34 |
| BoLA-T2a |  | 0.00028 | 44 |
| BoLA-T7 |  | 0.000271 | 41 |
| BoLA-5:06401 |  | 0.00021 | 25 |
| BoLA-2:00801 |  | 0.000183 | 25 |
| BoLA-3:00102 |  | 0.000153 | 33 |
| BoLA-3:00101 |  | 0.00013 | 40 |
| BoLA-AW10 |  | 0.00013 | 40 |
| BoLA-3:03501 |  | 0.00012 | 23 |
| BoLA-3:00201 |  | 0.000113 | 68 |
| BoLA-JSP.1 |  | 0.000113 | 68 |
| BoLA-4:06301 |  | 6.6e-05 | 38 |
| BoLA-3:00103 |  | 4.4e-05 | 51 |

| Alleles | Peptide | Score | Percentile rank |
| --- | --- | --- | --- |
| BoLA-T2c | FFASVQYKL | 0.647047 | 0.46 |
| BoLA-3:06501 |  | 0.410757 | 0.29 |
| BoLA-1:06701 |  | 0.363843 | 0.27 |
| BoLA-5:00301 |  | 0.259274 | 0.65 |
| BoLA-3:06801 |  | 0.184304 | 0.77 |
| BoLA-3:03701 |  | 0.140665 | 2.0 |
| BoLA-4:02402 |  | 0.135303 | 1.4 |
| BoLA-3:05801 |  | 0.095239 | 2.2 |
| BoLA-2:04401 |  | 0.090086 | 4.2 |
| BoLA-3:03801 |  | 0.088685 | 0.89 |
| BoLA-2:00501 |  | 0.085422 | 2.0 |
| BoLA-4:02401 |  | 0.083333 | 0.85 |
| BoLA-3:06602 |  | 0.081292 | 3.1 |
| BoLA-3:03601 |  | 0.079475 | 2.2 |
| BoLA-T7 |  | 0.076883 | 1.9 |
| BoLA-3:05201 |  | 0.075606 | 1.6 |
| BoLA-3:06601 |  | 0.071003 | 3.0 |
| BoLA-3:07301 |  | 0.070462 | 1.3 |
| BoLA-2:05701 |  | 0.069809 | 3.2 |
| BoLA-3:00201 |  | 0.059842 | 3.6 |
| BoLA-JSP.1 |  | 0.059842 | 3.6 |
| BoLA-1:03101 |  | 0.054125 | 3.2 |
| BoLA-4:06301 |  | 0.050144 | 1.2 |
| BoLA-5:07201 |  | 0.047184 | 2.2 |
| BoLA-6:04001 |  | 0.044758 | 3.5 |
| BoLA-3:00103 |  | 0.043189 | 0.72 |
| BoLA-2:04301 |  | 0.042737 | 2.3 |
| BoLA-3:01001 |  | 0.042085 | 1.8 |
| BoLA-2:04601 |  | 0.039376 | 3.7 |
| BoLA-2:04801 |  | 0.039163 | 2.7 |
| BoLA-3:01703 |  | 0.037257 | 6.5 |
| BoLA-3:05101 |  | 0.036393 | 2.6 |
| BoLA-3:01702 |  | 0.035477 | 4.6 |
| BoLA-1:02801 |  | 0.032242 | 1.7 |
| BoLA-2:04701 |  | 0.032181 | 5.5 |
| BoLA-2:04402 |  | 0.03185 | 3.5 |
| BoLA-2:00602 |  | 0.03073 | 3.9 |
| BoLA-2:06901 |  | 0.029884 | 2.1 |
| BoLA-3:01101 |  | 0.029426 | 5.1 |
| BoLA-1:02301 |  | 0.028592 | 5.8 |
| BoLA-D18.4 |  | 0.028592 | 5.8 |
| BoLA-1:03102 |  | 0.026896 | 4.9 |
| BoLA-1:06101 |  | 0.026604 | 6.0 |
| BoLA-3:05002 |  | 0.025638 | 6.6 |
| BoLA-3:01701 |  | 0.024774 | 4.2 |
| BoLA-2:06201 |  | 0.024468 | 6.8 |
| BoLA-3:00101 |  | 0.022572 | 1.6 |
| BoLA-AW10 |  | 0.022572 | 1.6 |
| BoLA-2:00601 |  | 0.021744 | 4.5 |
| BoLA-2:01602 |  | 0.021744 | 4.5 |
| BoLA-2:02201 |  | 0.021186 | 2.0 |
| BoLA-3:00102 |  | 0.020807 | 1.3 |
| BoLA-5:03901 |  | 0.02044 | 4.3 |
| BoLA-3:05001 |  | 0.018618 | 9.3 |
| BoLA-2:06001 |  | 0.017409 | 3.7 |
| BoLA-6:01301 |  | 0.016907 | 5.6 |
| BoLA-HD6 |  | 0.016907 | 5.6 |
| BoLA-2:01801 |  | 0.015419 | 5.4 |
| BoLA-2:01802 |  | 0.015419 | 5.4 |
| BoLA-1:04901 |  | 0.015408 | 7.7 |
| BoLA-2:02501 |  | 0.014856 | 4.1 |
| BoLA-1:02901 |  | 0.013931 | 5.7 |
| BoLA-2:04501 |  | 0.013578 | 10 |
| BoLA-1:07401 |  | 0.01351 | 3.3 |
| BoLA-2:00802 |  | 0.01308 | 3.0 |
| BoLA-2:05601 |  | 0.012641 | 5.0 |
| BoLA-1:04201 |  | 0.012179 | 7.5 |
| BoLA-2:03001 |  | 0.011553 | 3.9 |
| BoLA-2:07001 |  | 0.010582 | 5.9 |
| BoLA-1:00901 |  | 0.009619 | 7.6 |
| BoLA-amani.1 |  | 0.008872 | 6.9 |
| BoLA-2:05501 |  | 0.00874 | 11 |
| BoLA-2:02601 |  | 0.008339 | 9.3 |
| BoLA-2:02602 |  | 0.008339 | 9.3 |
| BoLA-2:02603 |  | 0.008339 | 9.3 |
| BoLA-2:01601 |  | 0.008125 | 6.1 |
| BoLA-2:07101 |  | 0.007881 | 12 |
| BoLA-6:01302 |  | 0.007132 | 3.7 |
| BoLA-6:03401 |  | 0.006873 | 5.0 |
| BoLA-2:00801 |  | 0.006763 | 4.4 |
| BoLA-1:02001 |  | 0.006601 | 8.9 |
| BoLA-3:02701 |  | 0.006073 | 11 |
| BoLA-3:02702 |  | 0.006073 | 11 |
| BoLA-3:00401 |  | 0.005917 | 12 |
| BoLA-3:00402 |  | 0.005917 | 12 |
| BoLA-3:00403 |  | 0.005917 | 12 |
| BoLA-3:05301 |  | 0.005917 | 12 |
| BoLA-gb1.7 |  | 0.005917 | 12 |
| BoLA-3:05901 |  | 0.005522 | 13 |
| BoLA-5:06401 |  | 0.005327 | 5.5 |
| BoLA-2:03202 |  | 0.004955 | 14 |
| BoLA-1:00902 |  | 0.00449 | 11 |
| BoLA-T5 |  | 0.00449 | 11 |
| BoLA-6:01501 |  | 0.003339 | 8.0 |
| BoLA-6:04101 |  | 0.003114 | 7.6 |
| BoLA-T2b |  | 0.003114 | 7.6 |
| BoLA-6:01402 |  | 0.003106 | 15 |
| BoLA-1:01901 |  | 0.001556 | 12 |
| BoLA-6:01401 |  | 0.001493 | 21 |
| BoLA-6:01502 |  | 0.001457 | 9.1 |
| BoLA-2:01201 |  | 0.001435 | 25 |
| BoLA-T2a |  | 0.001435 | 25 |
| BoLA-2:05401 |  | 0.000729 | 8.7 |
| BoLA-3:03501 |  | 0.000665 | 10 |
| BoLA-1:02101 |  | 0.000654 | 7.1 |
