# Supplementary material for: Development of a multi-epitope chimeric vaccine in silico against Babesia bovis, Theileria annulata, and Anaplasma marginale using computational biology tools and reverse vaccinology approach
Source: PLoS One. 2025 Jan 24;20(1):e0312262. doi: 10.1371/journal.pone.0312262 (PMC11759392; doi:10.1371/journal.pone.0312262)
Supplement: S10 File — (DOCX) [file pone.0312262.s016.docx]

The tables of all the ten MHC II epitopes of SPAG-1 with their scores and percentile rank representing their affinities for different BOLA alleles. The peptide which has been selected for chimeric vaccine construction has been represented in bold letters. The BoLA alleles binding with the selected peptide possessing a percentile value >50 is highlighted as yellow. The BoLA allele that has bonded with the selected peptide with the lowest percentile rank is highlighted as green.

| Alleles | Core Sequence | Peptide | Score | Percentile rank |
| --- | --- | --- | --- | --- |
| HLA-DRB3*02:01 | TTTGPGGNG | **TDGTTTGPGGNGEGG** | 0.0004 | 93 |
| HLA-DRB1*04:01 | TTTGPGGNG |  | 0.0003 | 96 |
| HLA-DRB3*01:01 | TTTGPGGNG |  | 0.0002 | 97 |
| HLA-DRB1*08:01 | TTGPGGNGE |  | 0.0010 | 97 |
| HLA-DRB1*11:01 | TTTGPGGNG |  | 0.0002 | 98 |
| HLA-DRB1*03:01 | TTTGPGGNG |  | 0.0002 | 98 |
| HLA-DRB1*14:01 | TTTGPGGNG |  | 0.0006 | 98 |
| HLA-DRB1*13:01 | TTTGPGGNG |  | 0.0002 | 100 |

| Alleles | Core Sequence | Peptide | Score | Percentile rank |
| --- | --- | --- | --- | --- |
| HLA-DRB1*04:01 | GKGQGSGLQ | AKAGKGQGSGLQGPG | 0.0022 | 73 |
| HLA-DRB3*02:01 | GKGQGSGLQ |  | 0.0009 | 87 |
| HLA-DRB1*13:01 | GKGQGSGLQ |  | 0.0016 | 88 |
| HLA-DRB1*11:01 | GKGQGSGLQ |  | 0.0007 | 89 |
| HLA-DRB1*08:01 | GKGQGSGLQ |  | 0.0021 | 92 |
| HLA-DRB1*03:01 | GKGQGSGLQ |  | 0.0003 | 95 |
| HLA-DRB1*14:01 | GKGQGSGLQ |  | 0.0008 | 97 |
| HLA-DRB3*01:01 | GKGQGSGLQ |  | 0.0001 | 98 |

| Alleles | Core sequence | Peptide | Score | Percentile rank |
| --- | --- | --- | --- | --- |
| HLA-DRB1*04:01 | EEEEEDDKS | DDDDEEEEEDDKSTS | 0.0012 | 83 |
| HLA-DRB3*01:01 | EEEEEDDKS |  | 0.0005 | 84 |
| HLA-DRB1*11:01 | EEEEEDDKS |  | 0.0004 | 94 |
| HLA-DRB3*02:01 | DEEEEEDDK |  | 0.0004 | 94 |
| HLA-DRB1*14:01 | EEEDDKSTS |  | 0.0006 | 98 |
| HLA-DRB1*08:01 | EEEEEDDKS |  | 0.0007 | 98 |
| HLA-DRB1*13:01 | EEEDDKSTS |  | 0.0003 | 99 |

| Alleles | Core Sequence | Peptide | Score | Percentile rank |
| --- | --- | --- | --- | --- |
| HLA-DRB3*02:01 | GSGLQGPGG | GKGQGSGLQGPGGVG | 0.0009 | 87 |
| HLA-DRB1*04:01 | GSGLQGPGG |  | 0.0005 | 93 |
| HLA-DRB1*11:01 | GSGLQGPGG |  | 0.0002 | 97 |
| HLA-DRB1*08:01 | QGSGLQGPG |  | 0.0009 | 97 |
| HLA-DRB3*01:01 | GSGLQGPGG |  | 0.0001 | 98 |
| HLA-DRB1*14:01 | GSGLQGPGG |  | 0.0006 | 98 |
| HLA-DRB1*03:01 | QGSGLQGPG |  | 0.0001 | 99 |
| HLA-DRB1*13:01 | GSGLQGPGG |  | 0.0003 | 99 |

| Alleles | Core Sequence | Peptide | Score | Percentile rank |
| --- | --- | --- | --- | --- |
| HLA-DRB1*04:01 | GKGQGSGLQ | KAGKGQGSGLQGPGG | 0.0006 | 91 |
| HLA-DRB3*02:01 | GKGQGSGLQ |  | 0.0004 | 93 |
| HLA-DRB1*11:01 | GKGQGSGLQ |  | 0.0003 | 95 |
| HLA-DRB1*13:01 | GKGQGSGLQ |  | 0.0007 | 96 |
| HLA-DRB1*08:01 | GKGQGSGLQ |  | 0.0011 | 96 |
| HLA-DRB1*03:01 | GKGQGSGLQ |  | 0.0002 | 98 |
| HLA-DRB3*01:01 | KGQGSGLQG |  | 0.0001 | 99 |
| HLA-DRB1*14:01 | GKGQGSGLQ |  | 0.0005 | 99 |

| Alleles | Core Sequence | Peptide | Score | Percentile rank |
| --- | --- | --- | --- | --- |
| HLA-DRB3*02:01 | GSGLQGPGG | GKAGKGQGSVSPGGG | 0.0009 | 87 |
| HLA-DRB1*04:01 | GSGLQGPGG |  | 0.0005 | 93 |
| HLA-DRB1*11:01 | GSGLQGPGG |  | 0.0002 | 97 |
| HLA-DRB1*08:01 | QGSGLQGPG |  | 0.0009 | 97 |
| HLA-DRB3*01:01 | GSGLQGPGG |  | 0.0001 | 98 |
| HLA-DRB1*14:01 | GSGLQGPGG |  | 0.0006 | 98 |
| HLA-DRB1*03:01 | QGSGLQGPG |  | 0.0001 | 99 |
| HLA-DRB1*13:01 | GSGLQGPGG |  | 0.0003 | 99 |

| Alleles | Core sequence | Peptide | Score | Percentile rank |
| --- | --- | --- | --- | --- |
| HLA-DRB3*02:01 | GSGLQGPGG | KGQGSGLQGPGGVGV | 0.0013 | 81 |
| HLA-DRB1*04:01 | GSGLQGPGG |  | 0.0007 | 89 |
| HLA-DRB3*01:01 | LQGPGGVGV |  | 0.0003 | 94 |
| HLA-DRB1*11:01 | GSGLQGPGG |  | 0.0002 | 97 |
| HLA-DRB1*13:01 | GSGLQGPGG |  | 0.0004 | 98 |
| HLA-DRB1*14:01 | GSGLQGPGG |  | 0.0006 | 98 |
| HLA-DRB1*08:01 | GSGLQGPGG |  | 0.0008 | 98 |
| HLA-DRB1*03:01 | GSGLQGPGG |  | 0.0001 | 99 |

| Alleles | Core Sequence | Peptide | Score | Percentile rank |
| --- | --- | --- | --- | --- |
| HLA-DRB3*02:01 | GSGLQGPGG | AGKGQGSGLQGPGGV | 0.0003 | 95 |
| HLA-DRB1*04:01 | GSGLQGPGG |  | 0.0002 | 97 |
| HLA-DRB3*01:01 | GSGLQGPGG |  | 0.0001 | 98 |
| HLA-DRB1*11:01 | GQGSGLQGP |  | 0.0002 | 98 |
| HLA-DRB1*08:01 | QGSGLQGPG |  | 0.0007 | 98 |
| HLA-DRB1*03:01 | QGSGLQGPG |  | 0.0001 | 99 |
| HLA-DRB1*13:01 | GQGSGLQGP |  | 0.0003 | 99 |
| HLA-DRB1*14:01 | GQGSGLQGP |  | 0.0004 | 99 |

| Alleles | Core Sequence | Peptide | Score | Percentile rank |
| --- | --- | --- | --- | --- |
| HLA-DRB1*04:01 | SSASPTSPT | SSSTSSASPTSPTTT | 0.0014 | 81 |
| HLA-DRB3*02:01 | SSASPTSPT |  | 0.0010 | 85 |
| HLA-DRB1*11:01 | SSASPTSPT |  | 0.0003 | 95 |
| HLA-DRB1*14:01 | SSASPTSPT |  | 0.0010 | 96 |
| HLA-DRB1*08:01 | SSASPTSPT |  | 0.0009 | 97 |
| HLA-DRB3*01:01 | SSASPTSPT |  | 0.0001 | 98 |
| HLA-DRB1*13:01 | SSASPTSPT |  | 0.0004 | 98 |
| HLA-DRB1*03:01 | SSASPTSPT |  | 0.0001 | 99 |

| Alleles | Core Sequence | Peptide | Score | Percentile rank |
| --- | --- | --- | --- | --- |
| HLA-DRB1*14:01 | GVGVPGVGV | DSISGPIPSPGDPRA | 0.0061 | 75 |
| HLA-DRB3*02:01 | GVPGVGVPG |  | 0.0013 | 82 |
| HLA-DRB1*04:01 | GVGVPGVGV |  | 0.0010 | 86 |
| HLA-DRB1*13:01 | GVGVPGVGV |  | 0.0018 | 86 |
| HLA-DRB3*01:01 | GVGVPGVGV |  | 0.0003 | 90 |
| HLA-DRB1*11:01 | GVGVPGVGV |  | 0.0005 | 92 |
| HLA-DRB1*03:01 | VGVPGVGVP |  | 0.0005 | 93 |
| HLA-DRB1*08:01 | GVGVPGVGV |  | 0.0017 | 94 |
