# Supplementary material for: Development of a multi-epitope chimeric vaccine in silico against Babesia bovis, Theileria annulata, and Anaplasma marginale using computational biology tools and reverse vaccinology approach
Source: PLoS One. 2025 Jan 24;20(1):e0312262. doi: 10.1371/journal.pone.0312262 (PMC11759392; doi:10.1371/journal.pone.0312262)
Supplement: S14 File — (DOCX) [file pone.0312262.s020.docx]

| BoLA Alleles | Protein sequence of BoLA allele | Amino acid length | Pseudo sequence similarity to HLA-allele |
| --- | --- | --- | --- |
| BoLA-DRB3*016:01 | MVCLYFSGGSWMAALIVMLMVLCPPLAWAREIQPHFLEYTKKECHFFNGTERVRFLDRYFHNGEEFVRFDSDWGEYRAVTELGRPDAKYWNSQKDFLEEKRAAVDTYCRHNYGVGESFTVQRRVEPIVTVYPAKTQPLQHHNLLVCSVNGFYPGNIEVRWFRNGHEEEAGVISTGLIQNGDWTFQTMVMLETVPQSGEVYTCQVEHPSQTSPITVEWRARSDSAQSKMMSGVGGFVLGLFFLAVGLFIYFRNQKGRPTLQPTGLLS | 266 | HLA-DRB1*1101 |
| BoLA-DRB3*4101 | HFLEYTKKECHFFNGTERVRFLDRYFYNGEEYVRFDSDWSEYRAVTELGRPDAEYWNSQEILERARAAVDTYCRHNYGVGESFTVQR | 87 | HLA-DRB1*0401 |
| BoLA-DRB3*7001 | HFLEYSKSECHFFNGTERVRFLDRYFHNGEENVRFDSDWGEFRAVTELGRPAAEYWNSQKDILERERAYVDTYCRHNYGVVESFTVQR | 88 | HLA-DRB1*1301 |
| BoLA-DRB3*2101 | IVMLMVLCPPLAWAREIQPHFLEYSTGECHFFNGTERVRFLDRYFYNGEEYVRFDSDWGEYRAVTELGRQDAEQWNSQKDFLEERRAEVDTYCRHNYGVGESFTVQRR | 108 | HLA-DRB1*0801 |
| BoLA-DRB3*3301 | IVMLMVLCPPLAWAREIQPHFLEYSTGECHFFNGTERVRFLDRYFYNGEEYVRFDSDWGEYRAVTELGRQDAEQWNSQKDFLEERRAEVDTYCRHNYGVGESFTVQRR | 108 | HLA-DRB1*1401 |
| BoLA-DRB3*3501 | HFLEYSKSECHFFNGTERVRYLDRYYTNGEENVRFDSDWGEFRAVTELGRPDAEYWNSQKDFLERKRANVDTYCRHNYGVFESFTVQRR | 89 | HLA-DRB1*0301 |
| BoLA-DRB3*1301 | HFLEYLKSECHFFNGTERVRFLERYFYNGEEYVRFDSDWGEYRAVTELGRPDAKYWNSQKDLLERKRANVDTYCRHNYGVVESFTVQWR | 89 | HLA-DRB3*0201 |
| BoLA-DRB3*4301 | HFLEYRKSECHFFNGTERVRYLDRSFYNGEEFVRFDSDWGEYRAVTELGRRVAEQWNSQKDLLERKRANVDTYCRHNYGVGESFTVQRR | 89 | HLA-DRB3*0101 |
