# Supplementary material for: Development of a multi-epitope chimeric vaccine in silico against Babesia bovis, Theileria annulata, and Anaplasma marginale using computational biology tools and reverse vaccinology approach
Source: PLoS One. 2025 Jan 24;20(1):e0312262. doi: 10.1371/journal.pone.0312262 (PMC11759392; doi:10.1371/journal.pone.0312262)
Supplement: S15 File — (DOCX) [file pone.0312262.s021.docx]

**Supplementary File 2**: Conserved regions of each protein retrieved from Clustal Omega with their antigenic scores and transmembrane topology screening.

| **Name of Microorganisms** | **Name of Protein** | **Conserved regions** | **VaxiJen score**  **(antigenic score)** | **TMHMM** | |
| --- | --- | --- | --- | --- | --- |
| ***Babesia bovis*** | **MSA-2c** | LEKNFEAVGMEATSA | **0.5764**( Probable **ANTIGEN** ). | outside | |
|  |  | YLSGQSNEELLKLLI | **1.0209**( Probable **ANTIGEN** ). | outside | |
|  |  | FINPSSTSEAETPSP | **0.6119**( Probable **ANTIGEN** ). | outside | |
|  | **AMA-1** | VPVILSSFFAEDALAS | **1.1921**( Probable **ANTIGEN** ). | outside | |
|  |  | NHGSGIYVDLGGYES | **0.4537**( Probable **ANTIGEN** ). | outside | |
|  |  | DPSYRGLAFPETAVDSNIPTQPKTRGSSS | **1.0578**( Probable **ANTIGEN** ). | outside | |
|  |  | GGSCVAIAPAFQEYA | **0.6101**( Probable **ANTIGEN** ). | outside | |
|  |  | SKVANAIFSPLSNVA | **0.4640** ( Probable **ANTIGEN** ) | outside | |
|  |  | SIALTAIGSPLEYDAV | **0.4538**( Probable **ANTIGEN** ). | outside | |
|  |  | LCSDVKPNWFIRFLH | **1.5180**( Probable **ANTIGEN** ). | outside | |
|  |  | YLSNYDYDTTLDADN | **1.0065**( Probable **ANTIGEN** ) | outside | |
| ***Theileria annulata*** | **SPAG-1** | IFVSGADKMPAGESS | **0.4196**( Probable **ANTIGEN** ). | outside | |
|  |  | RTSKPSPLVTLESAV | **0.4087**( Probable **ANTIGEN** ). | outside | |
|  |  | SFQEPVSQELEFQSD | **1.0739** ( Probable **ANTIGEN** ) | outside | |
|  |  | TEINESGSGSDEDED | **2.3507**( Probable **ANTIGEN** ). | outside | |
|  |  | DDDDEEEEEDDKSTS | **1.7988** ( Probable **ANTIGEN** ). | outside | |
|  |  | SSSTSSASPTSPTTT | **1.1213**( Probable **ANTIGEN** ). | outside | |
|  |  | LSQTGLGPSGSHAQQ | **1.0821**( Probable **ANTIGEN** ) | outside | |
|  |  | DPGVGVPGVGVPGVG | **0.9688**( Probable **ANTIGEN** ). | outside | |
|  |  | VPGVGVPGVGVPGVG | **0.9586**( Probable **ANTIGEN** ). | outside | |
|  |  | ADSSGLPGSGGLGAG | **1.5042**( Probable **ANTIGEN** ). | outside | |
|  |  | AKAGKGQGSGLQGPGGVGVVPGVG | **0.7450**( Probable **ANTIGEN** ). | outside | |
|  |  | AASSSSPGKPPGVGA | **0.9024**( Probable **ANTIGEN** ). | outside | |
|  |  | GVMPGVGVRAQGGVI | **0.5392**( Probable **ANTIGEN** ). | outside | |
|  |  | IGAPGVAGVPGGKPG | **0.9340**( Probable **ANTIGEN** ). | outside | |
|  |  | TDGTTTGPGGNGEGG | **2.9595 ( Probable ANTIGEN ).** | outside |  |
|  |  | IKNKLLGSGFEVASI | **1.1196**( Probable **ANTIGEN** ). | outside |  |
|  | **TASP** | DRQLNPIDFDPNDNQ | **0.5199**( Probable **ANTIGEN** ). | outside |  |
|  |  | QPTQQEPIEPEQPTQP | **0.9722**( Probable **ANTIGEN** ). | outside |  |
|  |  | AEPEELEPETVTVEV | **0.8752**( Probable **ANTIGEN** ). | outside |  |
|  |  | PEPIRSEEPTTTDQT | **0.7926**( Probable **ANTIGEN )** | outside |  |
|  |  | QQPVVEPPVQPTEST | **0.7607**( Probable **ANTIGEN** ). | outside |  |
|  |  | PTKASSSGDGAAPCHGKHHDDDSDG | **1.6960**( Probable **ANTIGEN** ) | outside |  |
| ***Anaplasma marginale*** | **Vir-B10** | LVVCAITGMAYYMFFRGSGTTETSEEPQ | **0.5833 ( Probable ANTIGEN ).** | outside |  |
|  |  | KRGTPMIVLGGGGDG | **0.9000**( Probable **ANTIGEN** ). | outside |  |
|  |  | GPSEDGGGQGTDSRF | **2.1276**( Probable **ANTIGEN** ) | outside |  |
|  |  | MIDAVLETAINSDIP | **1.2461**( Probable **ANTIGEN** ). | outside |  |
|  |  | LPHGIDIQINSAGTDELGRNGSAGF | **0.6103**( Probable **ANTIGEN** ). | outside |  |
|  | **OMP1** | FFASVQYKLAVPHFR | **0.7227**( Probable **ANTIGEN** ). | outside |  |
|  |  | DFIVEDKGKALNTFAMKEKQQGGTAKAA | **0.6214**( Probable **ANTIGEN** ). | outside |  |
|  |  | AEAPPAKGPDLASGGSFEGKYSP | **0.8176**( Probable **ANTIGEN** ). | outside |  |
|  |  | AGNVRLEAEGMYQKFPVDTKKY | **0.7700**( Probable **ANTIGEN** ). | outside |  |
|  |  | AQAAGGKLPGLLYPQASLG | **0.7983**( Probable **ANTIGEN** ). | outside |  |
