# Supplementary material for: Development of a multi-epitope chimeric vaccine in silico against Babesia bovis, Theileria annulata, and Anaplasma marginale using computational biology tools and reverse vaccinology approach
Source: PLoS One. 2025 Jan 24;20(1):e0312262. doi: 10.1371/journal.pone.0312262 (PMC11759392; doi:10.1371/journal.pone.0312262)
Supplement: S16 File — (DOCX) [file pone.0312262.s022.docx]

**Table 2(a): Antigenicity prediction, screening of transmembrane topology, allergenicity, conservancy along with toxicity assessment of the 10 best major histocompatibility complex class I epitopes of AMA-1.**

| **Epitopes** | **Start** | **End** | **Length** | **No. of BOLAs***  **binding epitopes** | **Antigenicity score** | **Allergenicity** | **Toxicity** | **Conservancy** |
| --- | --- | --- | --- | --- | --- | --- | --- | --- |
| VILSSFFAE | 3 | 11 | 9 | 98 | 2.168 | Probable non-allergen | Non-toxin | 100.00% |
| VPVILSSFF | 1 | 9 | 9 | 98 | 1.6785 | Probable non-allergen | Non-toxin | 100.00% |
| PVILSSFFA | 2 | 10 | 9 | 98 | 1.6759 | Probable non-allergen | Non-toxin | 100.00% |
| ILSSFFAED | 4 | 12 | 9 | 98 | 1.6578 | Probable non-allergen | Non-toxin | 100.00% |
| PNWFIRFLH | 7 | 15 | 9 | 98 | 1.6375 | Probable non-allergen | Non-toxin | 100.00% |
| PETAVDSNI | 10 | 18 | 9 | 98 | 1.6315 | Probable non-allergen | Non-toxin | 100.00% |
| ETAVDSNIP | 11 | 19 | 9 | 98 | 1.6019 | Probable non-allergen | Non-toxin | 100.00% |
| HGSGIYVDL | 2 | 10 | 9 | 98 | 1.3376 | Probable non-allergen | Non-toxin | 100.00% |
| LTAIGSPLE | 4 | 12 | 9 | 98 | 1.2928 | Probable non-allergen | Non-toxin | 100.00% |
| TAIGSPLEY | 5 | 13 | 9 | 98 | 1.2746 | Probable non-allergen | Non-toxin | 100.00% |

*BOLA- Bovine Leukocyte antigen
