# Supplementary material for: Development of a multi-epitope chimeric vaccine in silico against Babesia bovis, Theileria annulata, and Anaplasma marginale using computational biology tools and reverse vaccinology approach
Source: PLoS One. 2025 Jan 24;20(1):e0312262. doi: 10.1371/journal.pone.0312262 (PMC11759392; doi:10.1371/journal.pone.0312262)
Supplement: S17 File — (DOCX) [file pone.0312262.s023.docx]

**Table 2 (b): Antigenicity prediction, screening of transmembrane topology, allergenicity, conservancy along with toxicity assessment of the 10 best major histocompatibility complex class I epitopes of MSA-2c.**

| **Epitopes** | **Start** | **End** | **Length** | **No. of BOLAs***  **binding epitopes** | **Antigenicity score** | **Allergenicity** | **Toxicity** | **Conservancy** |
| --- | --- | --- | --- | --- | --- | --- | --- | --- |
| YLSGQSNEE | 1 | 9 | 9 | 294 | 1.5966 | Probable non-allergen | Non-toxin | 100.00% |
| LSGQSNEEL | 2 | 10 | 9 | 294 | 1.5215 | Probable non-allergen | Non-toxin | 100.00% |
| SGQSNEELL | 3 | 11 | 9 | 294 | 1.2708 | Probable non-allergen | Non-toxin | 100.00% |
| LEKNFEAVG | 1 | 9 | 9 | 294 | 0.9730 | Probable non-allergen | Non-toxin | 100.00% |
| NEELLKLLI | 7 | 15 | 9 | 294 | 0.9499 | Probable non-allergen | Non-toxin | 100.00% |
| TSEAETPSP | 7 | 15 | 9 | 294 | 0.9129 | Probable non-allergen | Non-toxin | 100.00% |
| STSEAETPS | 6 | 14 | 9 | 294 | 0.8743 | Probable non-allergen | Non-toxin | 100.00% |
| NPSSTSEAE | 3 | 11 | 9 | 294 | 0.8722 | Probable non-allergen | Non-toxin | 100.00% |
| PSSTSEAET | 4 | 12 | 9 | 294 | 0.8456 | Probable non-allergen | Non-toxin | 100.00% |
| GQSNEELLK | 4 | 12 | 9 | 294 | 0.7307 | Probable non-allergen | Non-toxin | 100.00% |

*BOLA- Bovine Leukocyte antigen
