# Supplementary material for: Development of a multi-epitope chimeric vaccine in silico against Babesia bovis, Theileria annulata, and Anaplasma marginale using computational biology tools and reverse vaccinology approach
Source: PLoS One. 2025 Jan 24;20(1):e0312262. doi: 10.1371/journal.pone.0312262 (PMC11759392; doi:10.1371/journal.pone.0312262)
Supplement: S18 File — (DOCX) [file pone.0312262.s024.docx]

**Table 3 (b): Antigenicity prediction, screening of transmembrane topology, allergenicity, conservancy along with toxicity assessment of the 10 best major histocompatibility complex class Iepitopes of SPAG-1.**

| **Epitopes** | **Start** | **End** | **Length** | **No. of BOLAs***  **binding epitopes** | **Antigenicity score** | **Allergenicity** | **Toxicity** | **Conservancy** |
| --- | --- | --- | --- | --- | --- | --- | --- | --- |
| GPGGNGEGG | 7 | 15 | 9 | 98 | 4.0758 | Probable non-allergen | Non-toxin | 100.00% |
| TGPGGNGEG | 6 | 14 | 9 | 98 | 3.6991 | Probable non-allergen | Non-toxin | 100.00% |
| TTTGPGGNG | 4 | 12 | 9 | 98 | 3.4672 | Probable non-allergen | Non-toxin | 100.00% |
| TTGPGGNGE | 5 | 13 | 9 | 98 | 3.4051 | Probable non-allergen | Non-toxin | 100.00% |
| GSGSDEDED | 7 | 15 | 9 | 98 | 3.2926 | Probable non-allergen | Non-toxin | 100.00% |
| KGQGSGLQG | 5 | 13 | 9 | 98 | 2.3075 | Probable non-allergen | Non-toxin | 100.00% |
| KPSGGGVPG | 5 | 13 | 9 | 98 | 2.2203 | Probable non-allergen | Non-toxin | 100.00% |
| NESGSSSEG | 1 | 9 | 9 | 98 | 2.1038 | Probable non-allergen | Non-toxin | 100.00% |
| SSGLPGSGG | 3 | 11 | 9 | 98 | 2.0990 | Probable non-allergen | Non-toxin | 100.00% |
| DSSGLPGSG | 2 | 10 | 9 | 98 | 2.0658 | Probable non-allergen | Non-toxin | 100.00% |

*BOLA- Bovine Leukocyte antigen
