# Supplementary material for: Development of a multi-epitope chimeric vaccine in silico against Babesia bovis, Theileria annulata, and Anaplasma marginale using computational biology tools and reverse vaccinology approach
Source: PLoS One. 2025 Jan 24;20(1):e0312262. doi: 10.1371/journal.pone.0312262 (PMC11759392; doi:10.1371/journal.pone.0312262)
Supplement: S19 File — (DOCX) [file pone.0312262.s025.docx]

**Table 3 (a): Antigenicity prediction, screening of transmembrane topology, allergenicity, conservancy along with toxicity assessment of the 10 best major histocompatibility complex class I epitopes of TASP.**

| **Epitopes** | **Start** | **End** | **Length** | **No. of BOLAs***  **binding epitopes** | **Antigenicity score** | **Allergenicity** | **Toxicity** | **Conservancy** |
| --- | --- | --- | --- | --- | --- | --- | --- | --- |
| TKASSSGDG | 2 | 10 | 9 | 98 | 2.6063 | Probable non-allergen | Non-toxin | 100.00% |
| ASSSGDGAA | 4 | 12 | 9 | 98 | 1.9999 | Probable non-allergen | Non-toxin | 100.00% |
| SGDGAAPCH | 7 | 15 | 9 | 98 | 1.9439 | Probable non-allergen | Non-toxin | 100.00% |
| PTKASSSGD | 1 | 9 | 9 | 98 | 1.9411 | Probable non-allergen | Non-toxin | 100.00% |
| SSSGDGAAP | 5 | 13 | 9 | 98 | 1.8073 | Probable non-allergen | Non-toxin | 100.00% |
| HGKHHDDDS | 15 | 23 | 9 | 98 | 1.7343     \|  \| \| --- \| | Probable non-allergen | Non-toxin | 100.00% |
| GKHHDDDSD | 16 | 24 | 9 | 98 | 1.6899 | Probable non-allergen | Non-toxin | 100.00% |
| CHGKHHDDD | 14 | 22 | 9 | 98 | 1.6090     \|  \| \| --- \| | Probable non-allergen | Non-toxin | 100.00% |
| KHHDDDSDG | 17 | 25 | 9 | 98 | 1.5899 | Probable non-allergen | Non-toxin | 100.00% |
| GDGAAPCHG | 8 | 16 | 9 | 98 | 1.4758     \|  \| \| --- \| | Probable non-allergen | Non-toxin | 100.00% |

*BOLA- Bovine Leukocyte antigen
