# Supplementary material for: Development of a multi-epitope chimeric vaccine in silico against Babesia bovis, Theileria annulata, and Anaplasma marginale using computational biology tools and reverse vaccinology approach
Source: PLoS One. 2025 Jan 24;20(1):e0312262. doi: 10.1371/journal.pone.0312262 (PMC11759392; doi:10.1371/journal.pone.0312262)
Supplement: S20 File — (DOCX) [file pone.0312262.s026.docx]

**Table 4 (a): Antigenicity prediction, screening of transmembrane topology, allergenicity, conservancy along with toxicity assessment of the 10 best Major Histocompatibility complex class I epitopes of Vir-B10.**

| **Epitopes** | **Start** | **End** | **Length** | **No. of BOLAs***  **binding epitopes** | **Antigenicity score** | **Allergenicity** | **Toxicity** | **Conservancy** |
| --- | --- | --- | --- | --- | --- | --- | --- | --- |
| DGGGQGTDS | 5 | 13 | 9 | 98 | 2.6052 | Probable non-allergen | Non-toxin | 100.00% |
| GGGQGTDSR | 6 | 14 | 9 | 98 | 2.4728 | Probable non-allergen | Non-toxin | 100.00% |
| IVLGGGGDG | 7 | 15 | 9 | 98 | 2.4524 | Probable non-allergen | Non-toxin | 100.00% |
| ELGRNGSAG | 16 | 24 | 9 | 98 | 1.9989 | Probable non-allergen | Non-toxin | 100.00% |
| SGTTETSEE | 18 | 26 | 9 | 98 | 1.3129 | Probable non-allergen | Non-toxin | 100.00% |
| GSGTTETSE | 17 | 25 | 9 | 98 | 1.3077 | Probable non-allergen | Non-toxin | 100.00% |
| MIVLGGGGD | 6 | 14 | 9 | 98 | 1.1384 | Probable non-allergen | Non-toxin | 100.00% |
| GTDELGRNG | 13 | 21 | 9 | 98 | 0.8048 | Probable non-allergen | Non-toxin | 100.00% |
| GTTETSEEP | 19 | 27 | 9 | 98 | 0.7532 | Probable non-allergen | Non-toxin | 100.00% |
| TGMAYYMFF | 7 | 15 | 9 | 98 | 0.5073 | Probable non-allergen | Non-toxin | 100.00% |

*BOLA- Bovine Leukocyte antigen
