# Supplementary material for: Development of a multi-epitope chimeric vaccine in silico against Babesia bovis, Theileria annulata, and Anaplasma marginale using computational biology tools and reverse vaccinology approach
Source: PLoS One. 2025 Jan 24;20(1):e0312262. doi: 10.1371/journal.pone.0312262 (PMC11759392; doi:10.1371/journal.pone.0312262)
Supplement: S21 File — (DOCX) [file pone.0312262.s027.docx]

**Table 4 (b): Antigenicity prediction, screening of transmembrane topology, allergenicity, conservancy along with toxicity assessment of the 10 best major histocompatibility complex class 1 epitopes of OMP-1.**

| **Epitopes** | **Start** | **End** | **Length** | **No. of BOLAs***  **binding epitopes.** | **Antigenicity score** | **Allergenicity** | **Toxicity** | **Conservancy** |
| --- | --- | --- | --- | --- | --- | --- | --- | --- |
| ASGGSFEGK | 12 | 20 | 9 | 98 | 2.2830 | Probable non-allergen | Non-toxin | 100.00% |
| SGGSFEGKY | 13 | 21 | 9 | 98 | 1.9582 | Probable non-allergen | Non-toxin | 100.00% |
| GGKLPGLLY | 5 | 13 | 9 | 98 | 1.4426 | Probable non-allergen | Non-toxin | 100.00% |
| GKLPGLLYP | 6 | 14 | 9 | 98 | 1.4157 | Probable non-allergen | Non-toxin | 100.00% |
| LPGLLYPQA | 8 | 16 | 9 | 98 | 1.3012 | Probable non-allergen | Non-toxin | 100.00% |
| AGGKLPGLL | 4 | 12 | 9 | 98 | 1.1130     \|  \| \| --- \| | Probable non-allergen | Non-toxin | 100.00% |
| GSFEGKYSP | 15 | 23 | 9 | 98 | 0.9138 | Probable non-allergen | Non-toxin | 100.00% |
| AQAAGGKLP | 1 | 9 | 9 | 98 | 0.8607 | Probable non-allergen | Non-toxin | 100.00% |
| AEAPPAKGP | 1 | 9 | 9 | 98 | 0.7576 | Probable non-allergen | Non-toxin | 100.00% |
| FFASVQYKL | 1 | 9 | 9 | 98 | 0.6819 | Probable non-allergen | Non-toxin | 100.00% |

*BOLA- Bovine Leukocyte antigen
