# Supplementary material for: Development of a multi-epitope chimeric vaccine in silico against Babesia bovis, Theileria annulata, and Anaplasma marginale using computational biology tools and reverse vaccinology approach
Source: PLoS One. 2025 Jan 24;20(1):e0312262. doi: 10.1371/journal.pone.0312262 (PMC11759392; doi:10.1371/journal.pone.0312262)
Supplement: S22 File — (DOCX) [file pone.0312262.s028.docx]

**Table 5 (a): Antigenicity prediction, screening of transmembrane topology, allergenicity, conservancy along with toxicity assessment of the 10 best major histocompatibility complex class II epitopes of AMA-1.**

| **Epitopes** | **Start** | **End** | **Length** | **No. of BOLAs***  **binding epitopes** | **Antigenicity score** | **Allergenicity** | **Toxicity** | **Conservancy** |
| --- | --- | --- | --- | --- | --- | --- | --- | --- |
| PVILSSFFAEDALAS | 2 | 16 | 15 | 8 | 1.2864 | Probable non-allergen | Non-toxin | 100.00% |
| VPVILSSFFAEDALA | 1 | 15 | 15 | 8 | 1.0845 | Probable non-allergen | Non-toxin | 100.00% |
| YLSNYDYDTTLDADN | 1 | 15 | 15 | 8 | 1.0065 | Probable non-allergen | Non-toxin | 100.00% |
| AFPETAVDSNIPTQP | 8 | 22 | 15 | 8 | 0.9931 | Probable non-allergen | Non-toxin | 100.00% |
| SIALTAIGSPLEYDA | 1 | 15 | 15 | 8 | 0.8029 | Probable non-allergen | Non-toxin | 100.00% |
| GLAFPETAVDSNIPT | 6 | 20 | 15 | 8 | 0.7954 | Probable non-allergen | Non-toxin | 100.00% |
| YRGLAFPETAVDSNI | 4 | 18 | 15 | 8 | 0.6374 | Probable non-allergen | Non-toxin | 100.00% |
| IALTAIGSPLEYDAV | 2 | 16 | 15 | 8 | 0.4934 | Probable non-allergen | Non-toxin | 100.00% |
| SKVANAIFSPLSNVA | 1 | 15 | 15 | 8 | 0.4640 | Probable non-allergen | Non-toxin | 100.00% |
| NHGSGIYVDLGGYES | 1 | 15 | 15 | 8 | 0.4537 | Probable non-allergen | Non-toxin | 100.00% |

*BOLA- Bovine Leukocyte antigen
