# Supplementary material for: Development of a multi-epitope chimeric vaccine in silico against Babesia bovis, Theileria annulata, and Anaplasma marginale using computational biology tools and reverse vaccinology approach
Source: PLoS One. 2025 Jan 24;20(1):e0312262. doi: 10.1371/journal.pone.0312262 (PMC11759392; doi:10.1371/journal.pone.0312262)
Supplement: S23 File — (Only one MHC II epitope was found for MSA-2c). (DOCX) [file pone.0312262.s029.docx]

**Table 5 (b): Antigenicity prediction, screening of transmembrane topology, allergenicity, conservancy along with toxicity assessment of the best major histocompatibility complex class IIepitope of MSA-2c. (Only one MHC II epitope was found for MSA-2c)**

| **Epitopes** | **Start** | **End** | **Length** | **No. of BOLAs*binding epitopes** | **Antigenicity score** | **Allergenicity** | **Toxicity** | **Conservancy** |
| --- | --- | --- | --- | --- | --- | --- | --- | --- |
| LEKNFEAVGMEATSA | 1 | 15 | 15 | 8 | 0.5764 | Probable non-allergen | Non-toxin | 100.00% |

*BOLA- Bovine Leukocyte antigen
