# Supplementary material for: Development of a multi-epitope chimeric vaccine in silico against Babesia bovis, Theileria annulata, and Anaplasma marginale using computational biology tools and reverse vaccinology approach
Source: PLoS One. 2025 Jan 24;20(1):e0312262. doi: 10.1371/journal.pone.0312262 (PMC11759392; doi:10.1371/journal.pone.0312262)
Supplement: S24 File — (DOCX) [file pone.0312262.s030.docx]

**Table 6(b): Antigenicity prediction, screening of transmembrane topology, allergenicity, conservancy along with toxicity assessment of the 10 best major histocompatibility complex class II epitope of SPAG-1.**

| **Epitopes** | **Start** | **End** | **Length** | **No. of BOLAs*binding epitopes** | **Antigenicity score** | **Allergenicity** | **Toxicity** | **Conservancy** |
| --- | --- | --- | --- | --- | --- | --- | --- | --- |
| TDGTTTGPGGNGEGG | 1 | 15 | 15 | 8 | 2.9595 | Probable non-allergen | Non-toxin | 100.00% |
| AKAGKGQGSGLQGPG | 1 | 15 | 15 | 8 | 1.8564 | Probable non-allergen | Non-toxin | 100.00% |
| DDDDEEEEEDDKSTS | 1 | 15 | 15 | 8 | 1.7988 | Probable non-allergen | Non-toxin | 100.00% |
| GKGQGSGLQGPGGVG | 4 | 18 | 15 | 8 | 1.7119 | Probable non-allergen | Non-toxin | 100.00% |
| KAGKGQGSGLQGPGG | 2 | 16 | 15 | 8 | 1.6931 | Probable non-allergen | Non-toxin | 100.00% |
| GKAGKGQGSVSPGGG | 1 | 15 | 15 | 8 | 1.5828 | Probable non-allergen | Non-toxin | 100.00% |
| KGQGSGLQGPGGVGV | 5 | 19 | 15 | 8 | 1.5115 | Probable non-allergen | Non-toxin | 100.00% |
| AGKGQGSGLQGPGGV | 3 | 17 | 15 | 8 | 1.3865 | Probable non-allergen | Non-toxin | 100.00% |
| SSSTSSASPTSPTTT | 1 | 15 | 15 | 8 | 1.1213 | Probable non-allergen | Non-toxin | 100.00% |
| DSISGPIPSPGDPRA | 2 | 16 | 15 | 8 | 1.1020 | Probable non-allergen | Non-toxin | 100.00% |

*BOLA- Bovine Leukocyte antigen
