# Supplementary material for: Development of a multi-epitope chimeric vaccine in silico against Babesia bovis, Theileria annulata, and Anaplasma marginale using computational biology tools and reverse vaccinology approach
Source: PLoS One. 2025 Jan 24;20(1):e0312262. doi: 10.1371/journal.pone.0312262 (PMC11759392; doi:10.1371/journal.pone.0312262)
Supplement: S25 File — (DOCX) [file pone.0312262.s031.docx]

**Table 6(a): Antigenicity prediction, screening of transmembrane topology, allergenicity, conservancy along with toxicity assessment of the 10 best major histocompatibility complex class II epitope of TASP.**

| **Epitopes** | **Start** | **End** | **Length** | **No. of BOLAs*binding epitopes** | **Antigenicity score** | **Allergenicity** | **Toxicity** | **Conservancy** |
| --- | --- | --- | --- | --- | --- | --- | --- | --- |
| PTKASSSGDGAAPCH | 1 | 15 | 15 | 8 | 1.8933 | Probable non-allergen | Non-toxin | 100.00% |
| SGDGAAPCHGKHHDD | 7 | 21 | 15 | 8 | 1.6264 | Probable non-allergen | Non-toxin | 100.00% |
| ASSSGDGAAPCHGKH | 4 | 18 | 15 | 8 | 1.6256 | Probable non-allergen | Non-toxin | 100.00% |
| SSSGDGAAPCHGKHH | 5 | 19 | 15 | 8 | 1.5494 | Probable non-allergen | Non-toxin | 100.00% |
| GDGAAPCHGKHHDDD | 8 | 22 | 15 | 8 | 1.5389 | Probable non-allergen | Non-toxin | 100.00% |
| DGAAPCHGKHHDDDS | 9 | 23 | 15 | 8 | 1.4793 | Probable non-allergen | Non-toxin | 100.00% |
| SSGDGAAPCHGKHHD | 6 | 20 | 15 | 8 | 1.4703 | Probable non-allergen | Non-toxin | 100.00% |
| PTQQEPIEPEQPTQP | 2 | 16 | 15 | 8 | 0.9230 | Probable non-allergen | Non-toxin | 100.00% |
| PEPIRSEEPTTTDQT | 1 | 15 | 15 | 8 | 0.7926 | Probable non-allergen | Non-toxin | 100.00% |
| QQPVVEPPVQPTEST | 1 | 15 | 15 | 8 | 0.7607 | Probable non-allergen | Non-toxin | 100.00% |

*BOLA- Bovine Leukocyte antigen
