# Supplementary material for: Development of a multi-epitope chimeric vaccine in silico against Babesia bovis, Theileria annulata, and Anaplasma marginale using computational biology tools and reverse vaccinology approach
Source: PLoS One. 2025 Jan 24;20(1):e0312262. doi: 10.1371/journal.pone.0312262 (PMC11759392; doi:10.1371/journal.pone.0312262)
Supplement: S26 File — (DOCX) [file pone.0312262.s032.docx]

**Table 7(b): Antigenicity prediction, screening of transmembrane topology, allergenicity, conservancy along with toxicity assessment of the 10 best major histocompatibility complex class II epitope of OMP-1.**

| **Epitopes** | **Start** | **End** | **Length** | **No. of BOLAs***  **binding epitopes** | **Antigenicity score** | **Allergenicity** | **Toxicity** | **Conservancy** |
| --- | --- | --- | --- | --- | --- | --- | --- | --- |
| AQAAGGKLPGLLYPQ | 1 | 15 | 15 | 8 | 1.2848 | Probable non-allergen | Non-toxin | 100.00% |
| QAAGGKLPGLLYPQA | 2 | 16 | 15 | 8 | 1.1154 | Probable non-allergen | Non-toxin | 100.00% |
| AGGKLPGLLYPQASL | 4 | 18 | 15 | 8 | 0.9219 | Probable non-allergen | Non-toxin | 100.00% |
| PAKGPDLASGGSFEG | 5 | 19 | 15 | 8 | 0.8893 | Probable non-allergen | Non-toxin | 100.00% |
| AAGGKLPGLLYPQAS | 3 | 17 | 15 | 8 | 0.8667 | Probable non-allergen | Non-toxin | 100.00% |
| PDLASGGSFEGKYSP | 9 | 23 | 15 | 8 | 0.8659     \|  \| \| --- \| | Probable non-allergen | Non-toxin | 100.00% |
| GGKLPGLLYPQASLG | 5 | 19 | 15 | 8 | 0.8578 | Probable non-allergen | Non-toxin | 100.00% |
| FFASVQYKLAVPHFR | 1 | 15 | 15 | 8 | 0.7227 | Probable non-allergen | Non-toxin | 100.00% |
| PPAKGPDLASGGSFE | 4 | 18 | 15 | 8 | 0.5378 | Probable non-allergen | Non-toxin | 100.00% |
| APPAKGPDLASGGSF | 3 | 17 | 15 | 8 | 0.5259 | Probable non-allergen | Non-toxin | 100.00% |

*BOLA- Bovine Leukocyte antigen
