# Supplementary material for: Development of a multi-epitope chimeric vaccine in silico against Babesia bovis, Theileria annulata, and Anaplasma marginale using computational biology tools and reverse vaccinology approach
Source: PLoS One. 2025 Jan 24;20(1):e0312262. doi: 10.1371/journal.pone.0312262 (PMC11759392; doi:10.1371/journal.pone.0312262)
Supplement: S27 File — (DOCX) [file pone.0312262.s033.docx]

**Table 7(a): Antigenicity prediction, screening of transmembrane topology, allergenicity, conservancy along with toxicity assessment of the 10 best major histocompatibility complex class II epitope of Vir-B10.**

| **Epitopes** | **Start** | **End** | **Length** | **No. of BOLAs***  **binding epitopes** | **Antigenicity score** | **Allergenicity** | **Toxicity** | **Conservancy** |
| --- | --- | --- | --- | --- | --- | --- | --- | --- |
| GPSEDGGGQGTDSRF | 1 | 15 | 15 | 8 | 2.1276 | Probable non-allergen | Non-toxin | 100.00% |
| MIDAVLETAINSDIP | 1 | 15 | 15 | 8 | 1.2461 | Probable non-allergen | Non-toxin | 100.00% |
| NSAGTDELGRNGSAG | 10 | 24 | 15 | 8 | 1.0128 | Probable non-allergen | Non-toxin | 100.00% |
| KRGTPMIVLGGGGDG | 1 | 15 | 15 | 8 | 0.9000 | Probable non-allergen | Non-toxin | 100.00% |
| LVVCAITGMAYYMFF | 1 | 15 | 15 | 8 | 0.7723 | Probable non-allergen | Non-toxin | 100.00% |
| SAGTDELGRNGSAGF | 11 | 25 | 15 | 8 | 0.7347 | Probable non-allergen | Non-toxin | 100.00% |
| PHGIDIQINSAGTDE | 2 | 16 | 15 | 8 | 0.6693 | Probable non-allergen | Non-toxin | 100.00% |
| LPHGIDIQINSAGTD | 1 | 15 | 15 | 8 | 0.6472     \|  \| \| --- \| | Probable non-allergen | Non-toxin | 100.00% |
| INSAGTDELGRNGSA | 9 | 23 | 15 | 8 | 0.5296     \|  \| \| --- \| | Probable non-allergen | Non-toxin | 100.00% |
| CAITGMAYYMFFRGS | 4 | 18 | 15 | 8 | 0.4432 | Probable non-allergen | Non-toxin | 100.00% |

*BOLA- Bovine Leukocyte antigen
