# Supplementary material for: Development of a multi-epitope chimeric vaccine in silico against Babesia bovis, Theileria annulata, and Anaplasma marginale using computational biology tools and reverse vaccinology approach
Source: PLoS One. 2025 Jan 24;20(1):e0312262. doi: 10.1371/journal.pone.0312262 (PMC11759392; doi:10.1371/journal.pone.0312262)
Supplement: S28 File — (DOCX) [file pone.0312262.s034.docx]

| Position of B-cell epitopes | Peptides selected for chimeric vaccine construction | Residues in peptides | Score |
| --- | --- | --- | --- |
| Bepipred linear epitope prediction 2.0 of MSA-2c (Threshold value- 0.500) | | | |
| MSA-2c  34-42 | PSSTSEAET | P | 0.501 |
|  |  | S | 0.535 |
|  |  | S | 0.559 |
|  |  | T | 0.6 |
|  |  | S | 0.611 |
|  |  | E | 0.612 |
|  |  | A | 0.604 |
|  |  | E | 0.583 |
|  |  | T | 0.512 |
| Kolaskar and Tongaonkar prediction of MSA-2c (Threshold value- 1.000) | | | |
| MSA-2c  14-19 | SAYLSG | S | 1.044 |
|  |  | A | 1.067 |
|  |  | Y | 1.04 |
|  |  | L | 1.055 |
|  |  | S | 1.055 |
|  |  | G | 1.014 |
| Kolaskar and Tongaonkar prediction of Ama-1 (Threshold value- 1.044) | | | |
| AMA-1  118-125 | RFLHYLSN | R | 1.065 |
|  |  | F | 1.103 |
|  |  | L | 1.126 |
|  |  | H | 1.106 |
|  |  | Y | 1.092 |
|  |  | L | 1.102 |
|  |  | S | 1.047 |
|  |  | N | 1.055 |
| AMA-1  21-26 | GIYVDL | G | 1.08 |
|  |  | I | 1.046 |
|  |  | Y | 1.1 |
|  |  | V | 1.08 |
|  |  | D | 1.08 |
|  |  | L | 1.081 |
| Emini surface accessibility prediction of SPAG-1 (Threshold value- 1.000) | | | |
| SPAG-1  415-424 | LSDPSSTDGT | L | 1.052 |
|  |  | S | 1.776 |
|  |  | D | 2.295 |
|  |  | P | 3.873 |
|  |  | S | 4.826 |
|  |  | S | 4.826 |
|  |  | T | 4.654 |
|  |  | D | 4.654 |
|  |  | G | 4.654 |
|  |  | T | 1.052 |
| Bepipred linear epitope prediction 2.0 of SPAG-1 (Threshold value- 0.500) | | | |
| SPAG-1  106-137 | DPGVGVPGVGVPGVGVPGVGVPGVGVPGVGAD | D | 0.634 |
|  |  | P | 0.623 |
|  |  | G | 0.628 |
|  |  | V | 0.615 |
|  |  | G | 0.601 |
|  |  | V | 0.611 |
|  |  | P | 0.599 |
|  |  | G | 0.603 |
|  |  | V | 0.596 |
|  |  | G | 0.608 |
|  |  | V | 0.615 |
|  |  | P | 0.62 |
|  |  | G | 0.639 |
|  |  | V | 0.638 |
|  |  | G | 0.635 |
|  |  | V | 0.629 |
|  |  | P | 0.62 |
|  |  | G | 0.631 |
|  |  | V | 0.618 |
|  |  | G | 0.621 |
|  |  | V | 0.623 |
|  |  | P | 0.602 |
|  |  | G | 0.616 |
|  |  | V | 0.613 |
|  |  | G | 0.617 |
|  |  | V | 0.619 |
|  |  | P | 0.612 |
|  |  | G | 0.623 |
|  |  | V | 0.619 |
|  |  | G | 0.622 |
|  |  | A | 0.638 |
|  |  | D | 0.634 |
| Kolaskar and Tongaonkar prediction of SPAG-1 (Threshold value- 1.008) | | | |
| SPAG-1  200-213 | QGGVIIGAPGVAGV | Q | 1.067 |
|  |  | G | 1.034 |
|  |  | G | 1.073 |
|  |  | V | 1.046 |
|  |  | I | 1.053 |
|  |  | I | 1.08 |
|  |  | G | 1.08 |
|  |  | A | 1.08 |
|  |  | P | 1.068 |
|  |  | G | 1.028 |
|  |  | V | 1.101 |
|  |  | A | 1.101 |
|  |  | G | 1.074 |
|  |  | V | 1.074 |
| Bepipred linear epitope prediction 2.0 of SPAG-1 (Threshold value- 0.500) | | | |
| SPAG-1  267-275 | SSGSHAQQS | S | 0.661 |
|  |  | S | 0.645 |
|  |  | G | 0.647 |
|  |  | S | 0.645 |
|  |  | H | 0.635 |
|  |  | A | 0.644 |
|  |  | Q | 0.645 |
|  |  | Q | 0.65 |
|  |  | S | 0.634 |
| Kolaskar and Tongaonkar prediction of SPAG-1 (Threshold value- 1.008) | | | |
| SPAG-1  290-308 | VPGVGVPGVGVPGVGVPGV | V | 1.119 |
|  |  | P | 1.119 |
|  |  | G | 1.119 |
|  |  | V | 1.146 |
|  |  | G | 1.074 |
|  |  | V | 1.119 |
|  |  | P | 1.119 |
|  |  | G | 1.119 |
|  |  | V | 1.146 |
|  |  | G | 1.074 |
|  |  | V | 1.119 |
|  |  | P | 1.119 |
|  |  | G | 1.119 |
|  |  | V | 1.146 |
|  |  | G | 1.074 |
|  |  | V | 1.119 |
|  |  | P | 1.119 |
|  |  | G | 1.074 |
|  |  | V | 1.073 |
| Kolaskar and Tongaonkar prediction of TASP (Threshold value- 1.002) | | | |
| TASP  62-73 | QQPVVEPPVQPT | Q | 1.038 |
|  |  | Q | 1.112 |
|  |  | P | 1.089 |
|  |  | V | 1.111 |
|  |  | V | 1.118 |
|  |  | E | 1.17 |
|  |  | P | 1.163 |
|  |  | P | 1.118 |
|  |  | V | 1.05 |
|  |  | Q | 1.05 |
|  |  | P | 1.043 |
|  |  | T | 1.02 |
| Bepipred linear epitope prediction 2.0 of Vir B-10 (Threshold value- 0.500) | | | |
| Vir B-10  17-32 | GSGTTETSEEPQKRGT | G | 0.522 |
|  |  | S | 0.555 |
|  |  | G | 0.58 |
|  |  | T | 0.617 |
|  |  | T | 0.643 |
|  |  | E | 0.659 |
|  |  | T | 0.664 |
|  |  | S | 0.682 |
|  |  | E | 0.683 |
|  |  | E | 0.683 |
|  |  | P | 0.666 |
|  |  | Q | 0.654 |
|  |  | K | 0.631 |
|  |  | R | 0.591 |
|  |  | G | 0.549 |
|  |  | T | 0.511 |
| Vir B-10  83-96 | NSAGTDELGRNGSA | N | 0.511 |
|  |  | S | 0.531 |
|  |  | A | 0.569 |
|  |  | G | 0.575 |
|  |  | T | 0.614 |
|  |  | D | 0.63 |
|  |  | E | 0.64 |
|  |  | L | 0.645 |
|  |  | G | 0.652 |
|  |  | R | 0.656 |
|  |  | N | 0.647 |
|  |  | G | 0.635 |
|  |  | S | 0.575 |
|  |  | A | 0.503 |
| Kolaskar and Tongaonkar prediction of Vir B-10 (Threshold value- 1.000) | | | |
| Vir B-10  33-39 | PMIVLGG | P | 1.012 |
|  |  | M | 1.065 |
|  |  | I | 1.065 |
|  |  | V | 1.06 |
|  |  | L | 1.033 |
|  |  | G | 1.04 |
|  |  | G | 0.999 |
| Emini surface accessibility prediction of Omp-1 (Threshold value- 1.000) | | | |
| OMP-1  61-66 | EGKYSP | E | 1.266 |
|  |  | G | 1.266 |
|  |  | K | 2.26 |
|  |  | Y | 1.318 |
|  |  | S | 1.318 |
|  |  | P | 1.06 |
