# Supplementary material for: Development of a multi-epitope chimeric vaccine in silico against Babesia bovis, Theileria annulata, and Anaplasma marginale using computational biology tools and reverse vaccinology approach
Source: PLoS One. 2025 Jan 24;20(1):e0312262. doi: 10.1371/journal.pone.0312262 (PMC11759392; doi:10.1371/journal.pone.0312262)
Supplement: S29 File — (DOCX) [file pone.0312262.s035.docx]

| Epitopes | Start | End | Length | Antigenicity score | TMHMM | Allergenicity |
| --- | --- | --- | --- | --- | --- | --- |
| Bepipred linear prediction method 2.0 | | | | | | |
| FEAVGMEATSAYLSGQSNEEL | 5 | 25 | 21 | 0.6625 ( Probable ANTIGEN ). | inside |  |
| PSSTSEAET | 34 | 42 | 9 | 0.8456 ( Probable ANTIGEN ). | outside | PROBABLE NON-ALLERGEN |
| Kolaskar and Tongaonkar prediction | | | | | | |
| LLKLLIFIN | 25 | 33 | 9 | 1.9355 ( Probable ANTIGEN ). | outside | PROBABLE NON-ALLERGEN |
|  |  |  |  |  |  |  |
|  |  |  |  |  |  |  |

**B-cell epitope prediction of MSA-2c**
