# Supplementary material for: Development of a multi-epitope chimeric vaccine in silico against Babesia bovis, Theileria annulata, and Anaplasma marginale using computational biology tools and reverse vaccinology approach
Source: PLoS One. 2025 Jan 24;20(1):e0312262. doi: 10.1371/journal.pone.0312262 (PMC11759392; doi:10.1371/journal.pone.0312262)
Supplement: S30 File — (DOCX) [file pone.0312262.s036.docx]

| Epitopes | Start | End | Length | Antigenicity score | TMHMM | Allergenicity |
| --- | --- | --- | --- | --- | --- | --- |
| Bepipred linear epitope prediction method 2.0 | | | | | |  |
| DALASNHGSG | 12 | 21 | 10 | 0.2880 ( Probable NON-ANTIGEN ). |  |  |
| GSPLEYDAVLC | 98 | 108 | 11 | 0.9207 ( Probable ANTIGEN ). | inside |  |
| NYDYDTTLD | 125 | 133 | 9 | 0.7766 ( Probable ANTIGEN ). | inside |  |
| Emini surface accessibility prediction method | | | | | |  |
| NIPTQPKTRGS | 48 | 58 | 11 | 1.1526 ( Probable ANTIGEN ). | inside |  |
| Kolaskar and Tongaonkar Prediction method | | | | | |  |
| GSCVAIAP | 62 | 69 | 8 | 1.0318 ( Probable ANTIGEN ). | outside | PROBABLE ALLERGEN |
| FSPLSNVASIALTAIG | 83 | 98 | 16 | 0.2514 ( Probable NON-ANTIGEN ).   \|  \| \| --- \| |  |  |
| YDAVLCSDV | 103 | 111 | 9 | 1.7447 ( Probable ANTIGEN ). | inside |  |
| RFLHYLSN | 118 | 125 | 8 | 0.4342 ( Probable ANTIGEN ). | outside | PROBABLE NON-ALLERGEN |

**B-cell epitope prediction of AMA-1.**
