# Supplementary material for: Development of a multi-epitope chimeric vaccine in silico against Babesia bovis, Theileria annulata, and Anaplasma marginale using computational biology tools and reverse vaccinology approach
Source: PLoS One. 2025 Jan 24;20(1):e0312262. doi: 10.1371/journal.pone.0312262 (PMC11759392; doi:10.1371/journal.pone.0312262)
Supplement: S31 File — (DOCX) [file pone.0312262.s037.docx]

| Epitopes | | Start | | End | | Length | | Antigenicity score | | TMHMM | Allergenicity |
| --- | --- | --- | --- | --- | --- | --- | --- | --- | --- | --- | --- |
| Emini surface accessibility prediction | | | | | | | | | | |  |
| GESSRTSKP | | 12 | | 20 | | 9 | |  | | inside |  |
| ELEFQSDTEI | | 39 | | 48 | | 10 | |  | | inside |  |
| ELELKSDTEI | | 225 | | 234 | | 10 | |  | | inside |  |
| SEGEDDDDEGK | | 241 | | 251 | | 11 | |  | | inside |  |
| HAQQSPQQDPAPSKP | | 271 | | 285 | | 15 | |  | | inside |  |
| LSDPSSTDGT | | 415 | | 424 | | 10 | | 0.4221 ( Probable ANTIGEN ). | | outside | PROBABLE NON-ALLERGEN |
| Kolaskar and Tongaonkar prediction method | | | | | | | | | | |  |
| PSPLVTLESAVSFQEPVSQELE | 20 | | 41 | | 22 | |  | | inside | |  |
| GVGVVPGVGAA | 166 | | 176 | | 11 | | -0.8429 ( Probable NON-ANTIGEN ).   \|  \| \| --- \| | | outside | |  |
| QGGVIIGAPGVAGV | 200 | | 213 | | 14 | | 0.7409 ( Probable ANTIGEN ). | | outside | | PROBABLE NON-ALLERGEN |
| GQPVSQELE | 219 | | 227 | | 9 | |  | | inside | |  |
| SSGSHAQQS | 267 | | 275 | | 9 | | 1.5257 ( Probable ANTIGEN ). | | outside | | PROBABLE NON-ALLERGEN |
| VPGVGVPGVGVPGVGVPGV | 290 | | 308 | | 19 | | 1.0606 ( Probable ANTIGEN ). | | outside | | PROBABLE NON-ALLERGEN |
| FAVQFLGDF | 336 | | 344 | | 9 | |  | | inside | |  |
| FEEVKSLVQTLINLKLAIANDF | 351 | | 372 | | 22 | |  | | inside | |  |
| SAFIVKISEI | 380 | | 389 | | 10 | |  | | inside | |  |

**B-cell epitope prediction of SPAG-1**
