# Supplementary material for: Development of a multi-epitope chimeric vaccine in silico against Babesia bovis, Theileria annulata, and Anaplasma marginale using computational biology tools and reverse vaccinology approach
Source: PLoS One. 2025 Jan 24;20(1):e0312262. doi: 10.1371/journal.pone.0312262 (PMC11759392; doi:10.1371/journal.pone.0312262)
Supplement: S32 File — (DOCX) [file pone.0312262.s038.docx]

| Epitopes | Start | End | Length | Antigenicity score | TMHMM | Allergenicity |
| --- | --- | --- | --- | --- | --- | --- |
| Emini surface accessibility prediction | | | | | |  |
| DPNDNQQPTQ | 10 | 19 | 10 | 1.2971 ( Probable ANTIGEN ). | inside |  |
| EQPTQPAE | 26 | 33 | 8 | 1.0117 ( Probable ANTIGEN ). | inside |  |
| EEPTTTDQT | 53 | 61 | 9 | 0.5471 ( Probable ANTIGEN ). | outside | PROBABLE ALLERGEN |
| Kolaskar and Tongaonkar prediction. | | | | | |  |
| PETVTVEVPEP | 39 | 49 | 11 | 0.6701 ( Probable ANTIGEN ). | outside | PROBABLE ALLERGEN |
| QQPVVEPPVQPT | 62 | 73 | 12 | 0.7532 ( Probable ANTIGEN ). | outside | PROBABLE NON-ALLERGEN |
|  |  |  |  |  |  |  |

**B-cell epitope prediction of TASP.**
