# Supplementary material for: Development of a multi-epitope chimeric vaccine in silico against Babesia bovis, Theileria annulata, and Anaplasma marginale using computational biology tools and reverse vaccinology approach
Source: PLoS One. 2025 Jan 24;20(1):e0312262. doi: 10.1371/journal.pone.0312262 (PMC11759392; doi:10.1371/journal.pone.0312262)
Supplement: S33 File — (DOCX) [file pone.0312262.s039.docx]

| Epitopes | Start | End | Length | Antigenicity score | TMHMM | Allergenicity |
| --- | --- | --- | --- | --- | --- | --- |
| Bepipred linear epitope prediction method 2.0 | | | | | |  |
| GSGTTETSEEPQKRGT | 17 | 32 | 16 | 1.2386 ( Probable ANTIGEN ). | outside | PROBABLE NON-ALLERGEN |
| GDGGPSEDGGGQGTDS | 41 | 56 | 16 | 2.6952 ( Probable ANTIGEN ). | inside |  |
| NSAGTDELGRNGSA | 83 | 96 | 14 | 0.6728 ( Probable ANTIGEN ). | outside | PROBABLE NON-ALLERGEN |
| Emini surface accessibility prediction. | | | | | |  |
| SGTTETSEEPQKRG | 18 | 31 | 14 | 1.3027 ( Probable ANTIGEN ). | inside |  |
| Kolaskar and Tongaonkar prediction method | | | | | |  |
| MIDAVLETA | 59 | 67 | 9 | 0.6212 ( Probable ANTIGEN ). | inside |  |
| NSDIPLPHGIDIQIN | 69 | 83 | 15 | 0.4065 ( Probable ANTIGEN ). | inside |  |

**B-cell epitope prediction of Vir-B10.**
