# Supplementary material for: Development of a multi-epitope chimeric vaccine in silico against Babesia bovis, Theileria annulata, and Anaplasma marginale using computational biology tools and reverse vaccinology approach
Source: PLoS One. 2025 Jan 24;20(1):e0312262. doi: 10.1371/journal.pone.0312262 (PMC11759392; doi:10.1371/journal.pone.0312262)
Supplement: S34 File — (DOCX) [file pone.0312262.s040.docx]

| Epitopes | Start | End | Length | Antigenicity score | TMHMM | Allergenicity |
| --- | --- | --- | --- | --- | --- | --- |
| Kolaskar and Tongaonkar prediction method | | | | | |  |
| SVQYKLAVPHFRDF | 4 | 17 | 14 | 0.4411 ( Probable ANTIGEN ). | inside |  |
|  |  |  |  |  |  |  |
|  |  |  |  |  |  |  |
|  |  |  |  |  |  |  |
|  |  |  |  |  |  |  |
|  |  |  |  |  |  |  |

**B-cell epitope prediction of OMP-1.**
