# Supplementary material for: Development of a multi-epitope chimeric vaccine in silico against Babesia bovis, Theileria annulata, and Anaplasma marginale using computational biology tools and reverse vaccinology approach
Source: PLoS One. 2025 Jan 24;20(1):e0312262. doi: 10.1371/journal.pone.0312262 (PMC11759392; doi:10.1371/journal.pone.0312262)
Supplement: S35 File — (DOCX) [file pone.0312262.s041.docx]

**Supplementary File 1: FASTA protein sequences of all the six proteins- MSA-2c AMA-1, SPAG-1, TASP, Vir B10 and OMP1.**

>tr|A0A0D6A0Y2|A0A0D6A0Y2_BABBO Merozoite surface antigen-2c OS=Babesia bovis OX=5865 GN=MSA-2c PE=4 SV=1

MVSFNIITVVLCSTLFNTTLASPQEEAVPTKQVNGSHLLFDDMKMLYDVMRSIDESMLKS

ILEKNFEAVGMEATSATKTHDALKAVKQLIKTDAPFNTSDFDTLDLEYLSGQSNEELLKL

LIEAIYGMEIIIEKTNSFVGESAKHSDKLDTDLRQYYWDNIYDDQSEYNKDKLTNLYKAF

ITDSGALGIASEELIKFETRKAQKDDYRFINPSSTSEAETPSPSSGENTAAQPPKPAETP

KPTGSSFTYGGLTVATLCYFVLSAF

>tr|T2HG14|T2HG14_BABBO Apical membrane antigen-1 OS=Babesia bovis OX=5865 GN=AMA-1 PE=3 SV=1

MQLHYKMQSTSLKYNYKRMLCMALVPVILSSFFAEDALASNSTLFAFHKEPNNRRLTRRS

SRGQLLNSRRGSDDASESSDRYPGRSGGAKNSSQSPWIKYMQKFDIPRNHGSGIYVDLGG

YESVGSKSYRMPVGKCPVVGKIIDLGNGADFLDPISSEDPSYRGLAFPETAVDSNIPTQP

KTRGSSSASAAKLSPVSAKDLRRWGYEGNDVANCSEYASNLIPASDRSTKYRYPFVFDSD

NQMCYILYSAIQYNQGNRYCDNDGSSEDGTSSLLCMKPYKSAEDAHLYYGSAKVDPDWEE

NCPMHPVRDAIFGKWSGGSCVAIAPAFQEYANSTEDCAAILFDNSATDLNIEAVNEDFNE

LKELTDGLKRLNMSKVANAIFSPLSNVAGTSRISRGVGMNWATYDKDSGMCALINETPNC

LILNAGSIALTAIGSPLEYDAVSYPCHIDTNGYVEPRAKNTNKYLDVPFEVTTALSMKTL

KCNAYVHTKYSDSCGTYFLCSDVKPNWFIRFLHMIGLYNTKRIVIFVCCTTTAIVLTIWI

WKRFIKAKKEPAPPSFDKYLSNYDYDTTLDADNETEQRLDSSAYSWGEAVQRPSDVTPVK

LSKIN

>tr|Q26675|Q26675_THEAN Sporozoite surface antigen OS=Theileria annulata OX=5874 GN=spag-1 PE=2 SV=1

MNIIHFLLTIPAIFVSGADKMPAGESSRTSKPSPLVTLESAVTQPSKDPFKTISALSKAT

KVWKSAVSVSGDSKTVPTPVSEPMITRSFQEPVSQELEFQSDTEINESGSGSDEDEDDDD

DEEEEEDDKSTSSKNGKGSPKAQPGVSSSSTSSASPTSPTTTLSQTGLGPSGSHAQQDPG

VGVPGVGVPGVGVPGVGVPGVGVPGVGVPGVGGVPGVGVAPGVGVPGVGVAPGVGVGADS

SGLPGSGGLGAGAKAGKGQGSGLQGPGGVGVVPGVGVAASSSSPGKPPGVGAGVMPGVGV

RAQGGVIIGAPGVAGVPGGKPGQPVSQELELKSDTEINESGSSSEGEDDDDEEEEEENKS

TSSKGAGGKAGKGQGSVSPGGGSSASQTSPTTTPQSGLASSGSHAQQSPQQDPAPSKPSG

GGVPGVGVPGVGVPGVGVPGVGVAPGVGVVPGVGGATTSSSSTTSTSTSTTTTTTTSSGK

PSDQGSHGTSPRNAVTRQTDSISGPIPSPGDPRAITGQMGEGERFAVQFLGDFKPKPRRY

EGQGTDAVKLKQFIFEEVKSLVQTLINLKLAIANDFVEISEKLKKKNQNYVPKLKLLKGE

QFDTKQKVANVLKGFNSLYFVFFMNLNLAKEVNKPEELAEFLWKLNTIPDKVGREFELAI

EKTKGSEKKKELEEAFNSIGLGFKIAQYATNDILSSITNSVYSLIKLKNFGDDFVTEVRK

SLQMVPHQKNLNGSAFIVKISEIINKKGTEDQDQTSGSGSKGTEGGSLRGQDLTEEEVLK

VLDELVKDVSEEHVGIGDLSDPSSRTPNAKPAELGPSLVIQNVPSDPSKVTPTQPSNLPQ

VPTTGPGNGTDGTTTGPGGNGEGGKDLKEGEKKEGLFQKIKNKLLGSGFEVASIIIPMTT

IIFSIVH

>tr|A7UAD3|A7UAD3_THEAN Surface protein (Fragment) OS=Theileria annulata OX=5874 PE=4 SV=1

DRQLNPIDFDPNDNQQPLEPDQPEQIDETQQPTQQEPIEPEQPTQPTAEPEELEPETVTV

EVPEPIRSEEPTTTDQTEEPTETQDCKHEMHTQQPVVEPPVQPTESTPTKASSSGDGAAP

CHGKHHDDDSDG

>tr|B9KHA8|B9KHA8_ANAMF VirB10 protein (VirB10) OS=Anaplasma marginale (strain Florida) OX=320483 GN=virB10 PE=4 SV=1

MSLGMSDETKDNNYGDGVEESVNVVGVHKSKKLFVVLVVCAITGMAYYMFFRGSGTTETS

EEPQPVIEKQDVDKLLKESEAPAQETAPRILTPPPKLPDLPPLVMPTAPELPTLARIAKK

KKEEPVVEETKEILPPAAESFFEPELQRRPMEDDGPPQHIPMPYRPGGGAIPEPVPSFLG

YDREKRGTPMIVLGGGGDGGPSEDGGGQGTDSRFSTWSTLDGTSSPSVKATRVGDPGYVI

LQGHMIDAVLETAINSDIPGVLRAIVSRDVYAEAGNMVMIPKGSRLIGSYFFDASGNNTR

VTVSWSRVILPHGIDIQINSAGTDELGRNGSAGFIDTKMGNVLTSTILLAGVSMGTAFVT

SKIPALQSEIKDTTEEKGEKKKEEKSSTLPVKIVSDAVKDFSESMKALIKKYVDTSKPTI

YVDQGTVMKVFVNQDIVFPREAVRR

>tr|Q2V9Q7|Q2V9Q7_ANAMF OMP1 OS=Anaplasma marginale (strain Florida) OX=320483 GN=omp1 PE=4 SV=1

MKKVYGLVYAALSLLFTPCGSFASPRPIDFSRGEGASGFFASVQYKLAVPHFRDFIVEDK

GKALNTFAMKEKQQGGTAKAAAGAATPPAAPSGAEAPPAKGPDLASGGSFEGKYSPEYLR

SAKAGSVSVGYSAGNVRLEAEGMYQKFPVDTKKYKDNPERAYRFAISAPDENSTTVATRP

QEPYHITAENKEVTTASLMANLCYDLLPESSQISPSACVGGGGSLVRFLGVTEVRWAYQA

KVGVQYFASRKAALFAYAYASRVHPEKFSNIPVVHHIKTESPKGSQGAAGSGGGESSAQA

AGGKLPGLLYPQASLGLDYFGFECGIRLVL
